# Transcriptome Analysis of Yamame (*Oncorhynchus masou*) in Normal Conditions after Heat Stress

**DOI:** 10.3390/biology8020021

**Published:** 2019-03-29

**Authors:** Waraporn Kraitavin, Kazutoshi Yoshitake, Yoji Igarashi, Susumu Mitsuyama, Shigeharu Kinoshita, Daisuke Kambayashi, Shugo Watabe, Shuichi Asakawa

**Affiliations:** 1 Graduate School of Agricultural and Life Sciences, The University of Tokyo, Bunkyo, Tokyo 113-8657, Japan; fofang881@gmail.com (W.K.); akyoshita@g.ecc.u-tokyo.ac.jp (K.Y.); aiga@mail.ecc.u-tokyo.ac.jp (Y.I.); a-mituya@mail.ecc.u-tokyo.ac.jp (S.M.); akino@mail.ecc.u-tokyo.ac.jp (S.K.); 2 Kobayashi Branch: Miyazaki Prefectural Fisheries Research Institute, Kobayashi, Miyazaki 886-0005, Japan; kambayashi-daisuke@pref.miyazaki.lg.jp; 3 School of Marine Biosciences, Kitasato University, Minami, Sagamihara, Kanagawa 252-0313, Japan; swatabe@kitasato-u.ac.jp

**Keywords:** high-temperature tolerance, yamame, selective breeding, differentially expressed genes, heat shock protein

## Abstract

Understanding the mechanism of high-temperature tolerance in cold-freshwater fish is crucial for predicting how certain species will cope with global warming. In this study, we investigated temperature tolerance in masu salmon (*Oncorhynchus masou*, known in Japan as ‘yamame’), an important aquaculture species. By selective breeding, we developed a group of yamame (F2) with high-temperature tolerance. This group was subjected to a high-temperature tolerance test and divided into two groups: High-temperature tolerant (HT) and non-high-temperature tolerant (NT). RNA was extracted from the gill and adipose fin tissues of each group, and the mRNA expression profiles were analyzed using RNA sequencing. A total of 2893 differentially expressed genes (DEGs) from the gill and 836 from the adipose fin were identified by comparing the HT and NT groups. Functional analyses were then performed to identify associated gene ontology (GO) terms and the Kyoto Encyclopedia of Genes and Genomes (KEGG) pathways. The HT group showed a high expression of heat shock protein 70 (HSP70) gene and enriched gene expression in the extracellular matrix (ECM), cell junction, and adhesion pathways in gill tissues compared to the NT group. The HT group also exhibited highly expressed genes in glycolysis and showed lower expression of the genes in the p53 signaling pathway in adipose fin tissues. Taken together, the difference of expression of some genes in the normal condition may be responsible for the difference in heat tolerance between the HT and NT yamame in the heat stress condition.

## 1. Introduction

Yamame (masu salmon; *Oncorhynchus masou*) is a member of the family Salmonidae and inhabits Japanese rivers. They are a non-migratory form of masu salmon, which live continuously in their natal rivers during their life cycles [1,2]. As temperatures in the world increase due to global warming, cold-freshwater fish such as rainbow trout and yamame will be affected [3]. High water temperature can affect the metabolism, protein degradation, and immune defense of fish and lead to higher risks of disease in fish. These factors, that occur during heat stress conditions, thus reduce their egg production and fertility [3,4]. Moreover, the adaptive responses after heat stress also play a crucial role in recovery [5,6]. According to Liu et al. [7], thermal-tolerant fish exhibit a shorter duration of heat stress response and earlier decline of HSP70 proteins when undergoing heat stress.

The Miyazaki Prefectural Fisheries Research Institute established high-temperature tolerant (HT) rainbow trout through selective breeding in 1996 [8]. In addition, the thermally selected rainbow trout showed highly expressed levels of heat shock protein (HSP) genes compared with the normal group without heat stress [9]. High temperatures cause cellular stresses and induce protein unfolding, which activates transcription factors, including heat shock factor 1 (HSF1), tumor protein p53 (p53), and nuclear factor-kappa B (NF-kB); such temperatures also allow HSF1 to produce HSPs (HSP70 and HSP90) in the cytoplasm [10,11]. HSPs are molecular chaperones involved in temperature tolerance [7,9], e.g., by preventing protein aggregation, assisting damaged proteins, and acting as primary genes, to cope with heat stress in the cell [12]. HSPs are associated with heat stress in tilapia [4], rainbow trout [9,13], killifish [14], and catfish [15], and heat stress induces tissue damage through apoptosis and necrosis, which increases the rate of cell proliferation and metabolism for maintaining cell activities [5,12,14,16]. p53 relates to the p53 signaling pathway, which is activated by the external environment, especially heat stress conditions [17].

Many studies have investigated the differentially expressed genes (DEGs) during the response to heat stress in fish; for instance, gene expression of Chinese minnow from the northern region showed greater thermal tolerance than the southern species [18], and the specific regulation patterns indicated a difference between imported rainbow trout and a local breeding strain [16,19]. The thermal-tolerant rainbow trout showed higher expression levels of HSPs than the low thermal-tolerant group under heat stress in fin and gill tissues [9,20]. In addition, HSPs were also highly expressed in other tissues, such as the brain, liver, muscle, and heart, in the thermally selected strains of rainbow trout [13]. HSPs were also up-regulated to promote the degradation of damaged proteins in the ER pathway during heat stress [21].

In a previous study, we examined rainbow trout in a high-temperature test, using the time to loss of equilibrium (TLE) method [8,9,20,22]. In this study, we performed a high-temperature tolerance test on yamame (F2) by observing TLE at 27.5 °C. A week after the test, we obtained gill and adipose fin tissue from the tested yamame to avoid a direct acute effect of heat stress, and we performed transcriptome analysis of the tissues. Gill and adipose fin tissues are the primary sites in direct contact with external factors such as temperature, salinity, and UV [7,23]. The gill is a complex tissue responsible for gas exchange, ion balance, and osmoregulation; it is also the first site for regulating plasma pH and excreting nitrogenous waste [16]. The gills were also shown to be an immune relevant organ in fish [24]. Quick sampling of fins is not lethal; it has been used in fisheries management for quite some time and shows gene expression response to external stimuli for some genes [25]. In our previous study of rainbow trout, we observed a higher expression of several genes, including HSP70b, in HT rainbow trout in the normal condition, indicating an association between the constitutive expression of some genes in the normal condition and HT phenotype [9].

In this study, we performed a comparative analysis between the HT and non-high-temperature tolerant (NT) yamame groups. Through this comparative transcriptomic investigation, we sought to understand the differences between the two groups in the normal constitutive condition. Finding DEGs in normal conditions enables us to select individuals with the HT trait without performing the heat stress test. In addition, the differential expression pattern between the HT and NT fish should help us to understand the molecular mechanism of high-temperature tolerance. Such understanding should also be important for further explorations into the heat tolerance of Salmonidae and other fish.

## 2. Materials and Methods

### 2.1. Fish Samples for Sequencing

Yamame, which originate from the Hitotsuse River in Miyazaki prefecture, Japan, are bred in Kobayashi Branch, Miyazaki Prefectural Fisheries Research Institute. Two generations of yamame were selected based on their tolerance to high temperature before our experiment. Then, these yamame were tested by exposure to 27.5 °C and were selected based on the TLE [20]. The fish showing more than 80 min of TLE were selected as having high-temperature resistance (F0); we then produced F1 by mating F0. In 2016, yamame (F1) were selected using the same method, and the selected F1 were mated to produce F2. In 2017, 3000 male and female fish (F2) were kept in a 1-T tank under a flow–throw condition at 17.5 °C with natural sunlight entering the room. About 50~70 fish were transferred to a 57-L tank at 27.5 °C, and we started an examination without acclimation. The fish showing a TLE of less than 20 min were identified as NT, and the fish showing no loss of equilibrium at 80 min were identified as HT. After the test, the fish were immediately returned and kept in a water tank for one week at a constant temperature of 17.5 °C. Gill and adipose fin tissues were collected and immediately put into 2 mL tubes with an RNAlater reagent to inhibit RNA degradation. The study was approved by the Subcommittee of Institutional Animal Care and the Graduate School of Agricultural and Life Sciences, University of Tokyo (permission #P14-952).

### 2.2. Constructing mRNA Libraries and Sequencing

Following the standard protocol, total RNAs were extracted from the gill and adipose fin tissues of the fish samples in the HT and NT groups using an RNeasy Mini Kit (QIAGEN, Valencia, CA, USA). RNA quality and integrity were assessed on an Agilent 2200 Tapestation (Agilent Technologies, Santa Clara, CA, USA) using RNA ScreenTape. RNA concentrations were measured by a Qubit^®^ 2.0 Fluorometer RNA assay kit (Life Technologies, Carlsbad, CA, USA). Approximately 2 µg of total RNA from each sample was used to construct complementary DNAs (cDNAs) with SuperScript II (Invitrogen, Carlsbad, CA, USA). We prepared cDNA libraries using a TruSeq Stranded mRNA Library Prep Kit (Illumina, San Diego, CA, USA). Index adapters were added to identify sequences for each sample in the final data. The quality of the libraries was measured on an Agilent 2200 Tapestation using High Sensitivity RNA ScreenTape, and qPCR was measured using a KAPA SYBR Fast qPCR Kit (NIPPON Genetics, Tokyo, Japan). Subsequently, the 40 libraries (10 biological replicates per group (HT and NT), per tissue (gill and fin)) were subjected to paired-end (2 × 100 bp) sequencing on the Illumina HiSeq 4000 Sequencing System (Illumina) at BGI, Japan, and all samples were sequenced in the same lane.

### 2.3. Data Analysis of mRNAs

Raw sequences were performed into clean reads after removing the adapter sequences and low-quality reads (Q < 20). The resulting clean reads were then de novo assembled using Trinity version 2.4.0 with Trimmomatic, default parameters [26] and pseudo-aligned to the Trinity assembled contigs as a reference using Kallisto version 0.43.1 [27]. The assembled contigs were annotated using Trinotate for a BLAST search against the Swiss–Prot. Following a previous authors’ protocol to use an adjusted *p* value of <0.05 [28], the DESeq2 R package was used for an analysis of the differential gene expression between the HT and NT in gill tissues and the HT and NT in adipose fin tissues with 10 biological replicates per condition. 

### 2.4. Functional Gene Enrichment Analysis of DEGs

The Gene Ontology (GO) enrichment analysis and Kyoto Encyclopedia of Genes and Genomes (KEGG) pathway annotation of all DEGs were generated using the KOBAS desktop application software version 3.0 [29], which uses the KEGG database (http://www.kegg.jp [30]) and statistical enrichment of DEGs. Set of genes of the *Danio rerio* (zebrafish) was used as a reference for the enrichment analysis. The significantly enriched KEGG pathways were determined by the hypergeometric test and Fisher’s exact test *p* value (<0.05). Thereafter, we implemented a ‘ggplot2’ R package to create scatter plots (Figure 3) [31] and a ‘gplots’ package to generate heatmap.2 (Figures 5 and 6) [32]. A statistical significance analysis t test was conducted to compare the gene expression levels between the HT and NT groups (Figure 4); a two-tailed *p* value of <0.05 was considered significant. 

### 2.5. Data Accessibility

The raw transcriptome reads obtained during this study were deposited at DNA Data Bank of Japan (DDBJ) Sequence Read Archive (DRA) under submission accession number DRA007715.

## 3. Results

### 3.1. De Novo Assembly of Gill and Adipose Fin Tissues Transcripts

We chose two tissues, gill and adipose fin, because these tissues suffer directly from temperature change. Adipose fin is also one of the easiest tissues on which to perform a biopsy, which is useful for diagnosis without killing or injuring the fish. To identify the DEGs, we created 10 libraries for individuals from the HT and NT groups for each tissue and obtained 40 libraries (HT_gill, NT_gill, HT_fin, and NT_fin) using the Illumina HiSeq 4000 Sequencing System. After removing adapter sequences and low-quality readings, 61,460,394 (HT_gill), 78,088,572 (NT_gill), 71,282,330 (HT_fin), and 59,749,778 (NT_fin), the average clean readings, remained in the respective libraries, with 10 replicates from the HT and NT groups (Table 1). Table 1 summarizes the major statistics, including the clean readings, Q20, and GC content. The Trinity assembler generated 409,318 transcript contigs (with 141,144 known sequences and 268,174 unknown sequences) in the gill tissues and 376,635 transcript contigs (with 132,179 known sequences and 244,456 unknown sequences) in the adipose fin tissues. The biological replicates and the significantly high similarity between the biological replicates for the gills and adipose fins were confirmed (Figures S1 and S2). Three outliers were found in Figure S2. The numbers of the reads for these samples in the range of those of other samples and the reasons were unknown.

### 3.2. Gene Expression Profiling

**Figure 1:**
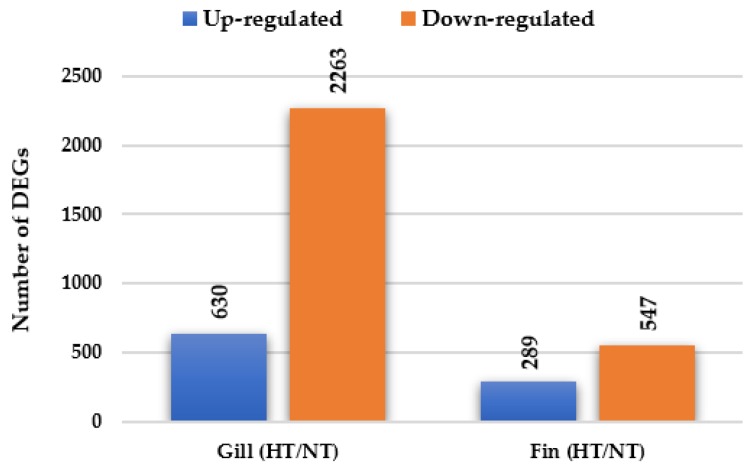
Differentially expressed genes (DEGs) were identified by comparing the HT and NT groups. A total of 2893 DEGs and 836 DEGs in gill and adipose fin were identified, including 630 up-regulated and 2263 down-regulated DEGs in the gills and 289 up-regulated and 547 down-regulated DEGs in the adipose fins.

A total of 3729 genes were significantly differentially expressed (including 2081 known and 1648 unknown genes) in the gill and adipose fin tissues in the normal condition in individuals from the HT groups compared with those from the NT groups (Figure 1). In the gill tissue, 2893 DEGs were identified, including 630 up-regulated DEGs and 2263 down-regulated DEGs. In the adipose fin tissue, 836 DEGs were detected, comprising 289 up-regulated DEGs and 547 down-regulated DEGs (Figure 1, Tables S1 and S2). Table 2 and Table 3 show the top 20 up- and down-regulated DEGs in the gill and adipose fin tissues. 

### 3.3. GO Analysis of DEGs in Gill and Adipose Fin

**Figure 2:**
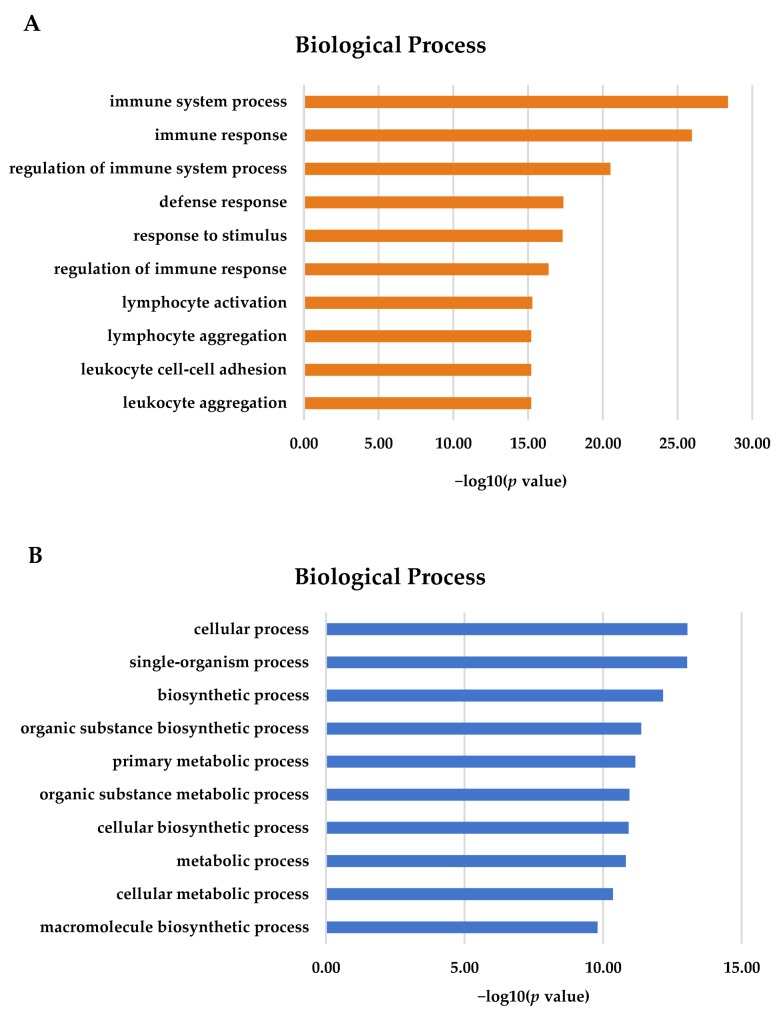
Gene Ontology (GO) terms for DEGs using KOBAS 3.0. (**A)** The majority of DEGs in the gill tissues were associated with immune and defense response (**B**) Most DEGs in the adipose fin tissues were involved in the cellular process, biosynthesis, and metabolism.

GO terms are associated with DEGs. For the gill, most of the 2893 DEGs were annotated in terms of biological processes (Figure 2A). The most important GO terms in the gill tissues were involved in immune system processes, immune responses, and the regulation of immune processes (*p* < 0.05). For the adipose fin, 836 DEGs were detected and showed significance (*p* < 0.05) in terms of biological processes. The most important GO terms included a cellular process, single-organism process, intracellular part, biosynthetic process, and metabolic process (Figure 2B). 

### 3.4. KEGG Pathway Enrichment Analysis of DEGs in Gill and Adipose Fin

**Figure 3:**
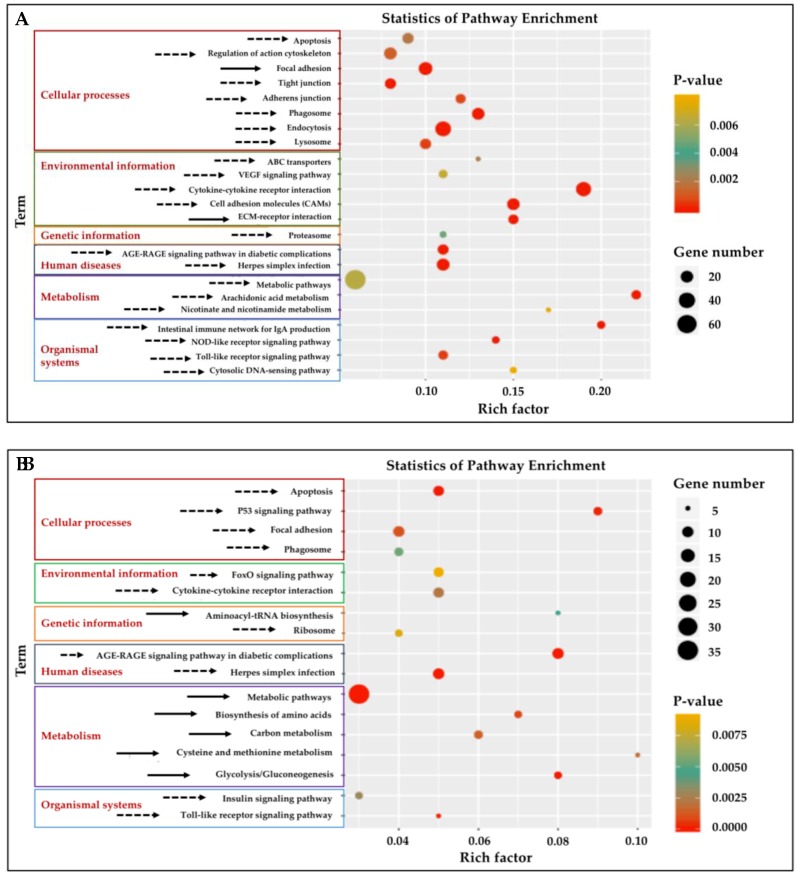
Kyoto Encyclopedia of Genes and Genomes (KEGG) pathway analysis of enrichment of the DEGs from the gill and adipose fin. (**A**) Twenty-three KEGG pathways were enriched (*p* < 0.01) in the gill tissues by comparing the HT and NT groups. (**B**) Seventeen KEGG pathways were enriched (*p* < 0.01) in adipose fin tissues. Rich factor is the ratio of the number of DEGs to the total number of genes in a certain pathway. The size and color of the dots represent the number of genes and the range of the *p* values, respectively. Solid arrows indicate up-regulated pathways, and dotted arrows indicate down-regulated pathways.

Twenty-three KEGG pathways were enriched (*p* < 0.01) (Figure 3A, Table S3) in the gill tissue and 17 pathways in adipose fin tissue (Figure 3B, Table S4). Significantly enriched pathways (cut-off of ≥5 DEGs, *p* < 0.01) (Table S3) were classified into six classes in both gill and adipose fin tissues: Cellular processes, environmental information processing, genetic information processing, human diseases, metabolism, and organismal systems. The cytokine–cytokine receptor interaction, cell adhesion molecules (CAMs), and the extracellular matrix (ECM)–receptor interaction showed highly significant pathways in the gill tissues (Figure 3A). Likewise, p53 signaling pathway and glycolysis showed as highly significant in the adipose fin tissues (Figure 3B).

### 3.5. Specific Transcriptomic Responses in Gill Tissues

**Figure 4:**
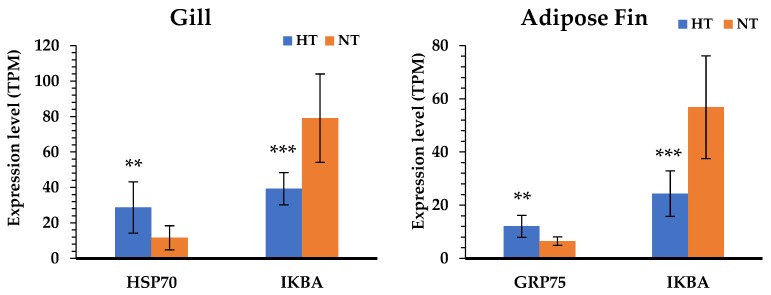
Comparison of the gene expression level between the HT and NT groups determined by normalized reads (TPM). The blue bars represent the gene expression of the HT group; the orange bars represent the gene expression of the NT group. Differences in statistically significant expression are marked (^(^*^)^ *p* < 0.05; ^(^**^)^ *p* < 0.01; ^(^***^)^ *p* < 0.001).

**Figure 5:**
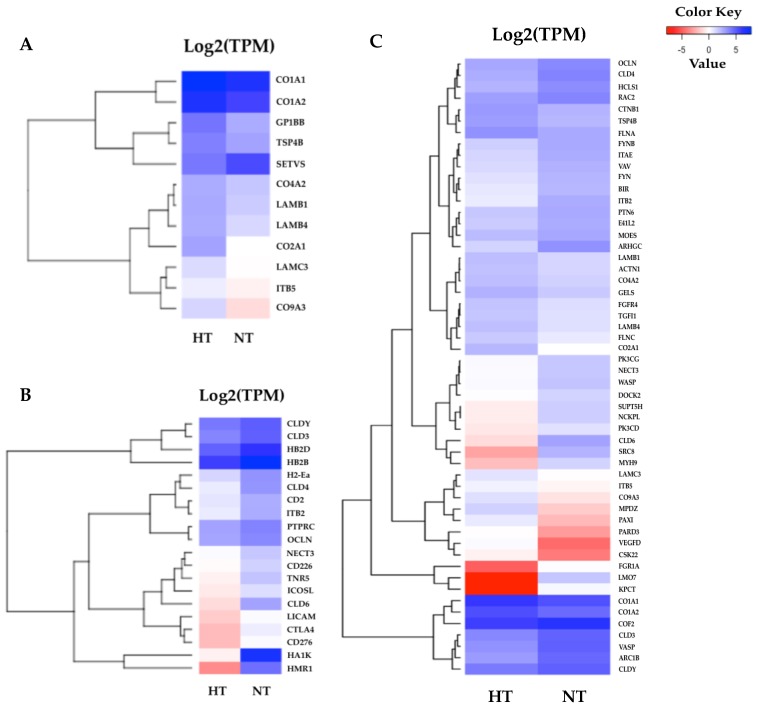
Heatmaps of specific enriched pathways in the gill tissues. (**A**) Twelve DEGs of the extracellular matrix (ECM)–receptor interaction were highly expressed in the HT group and were divided into two major clusters. (**B**) Twenty DEGs of cell adhesion molecules (CAMs). (**C**) 54 DEGs were identified in the gill tissues, most of which were associated with cell junction and the adhesion pathway. The red indicates less expressed DEGs, and the blue indicates highly expressed DEGs when comparing the HT and NT groups.

According to the results of the KEGG pathway annotations, the common pathways identified in both tissues—including cytokine–cytokine receptor interaction, the toll-like receptor signaling pathway, apoptosis, and focal adhesion—are crucial to heat response. We also found that the HSP genes were highly significant in the HT group gill and adipose fin tissues, and NF-kappa-B inhibitor alpha (IKBA) showed to be down-regulated in the HT compared to the NT groups (Figure 4). Furthermore, the common pathways showed that many genes were more highly expressed in the gill tissue than in the adipose fin tissue. The pathways annotated only in the gill tissue included the ECM–receptor interaction, CAMs, and pathways related to cell junction and adhesion, comprising the focal adhesion, tight junction, adherens junction, and the regulation of actin cytoskeleton (Figure 5; Tables S5, S6 and S7). 

### 3.6. Specific Transcriptomic Responses in Fin Tissues

**Figure 6:**
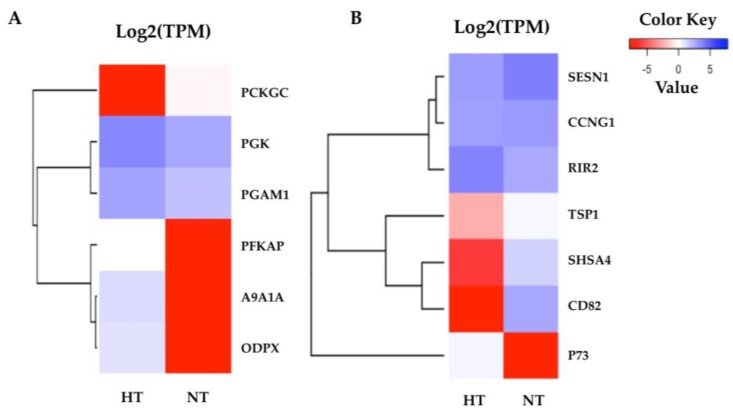
Heatmaps of the specific enriched pathway in adipose fin tissue. (**A**) Six DEGs of glycolysis were highly expressed in the HT group, but not phosphoenolpyruvate carboxykinase cytosolic (PCKGC). (**B**) The seven DEGs of the p53 signaling pathway show the difference between the HT and NT groups. The red indicates less expressed DEGs, and the blue indicates highly expressed DEGs when comparing the HT and NT groups.

The highly significant pathways were related to cellular processes and cell metabolism in the normal condition after heat stress. According to the KEGG pathway annotations, the highly enriched pathways in the adipose fin were annotated as glycolysis and the p53 signaling pathway (Figure 6). The most related genes in the glycolysis pathway, including phosphofructokinase, platelet (PFKAP), aldehyde dehydrogenase family 9 member A1-A (A9A1A), and pyruvate dehydrogenase protein X component (ODPX) were highly expressed in the fin. These are major enzymes involved in the glycolysis pathway (Tables S8 and S9). p73 was less expressed, and CD82 and Shisa family member 4 (SHSA4) were shown to have a lower expression in the p53 signaling pathway, which plays a major role in p53 negative feedback, reducing proteins in the p53 pathway. 

## 4. Discussion

The comparative gene expression profiles between individuals of yamame from the HT and NT groups are useful for understanding the difference between the two groups in the normal condition after heat stress; the candidate genes can also be exploited to develop high-temperature-tolerant yamame. Our study showed that yamame of the HT group induced changes in metabolism in the adipose fin tissue and regulated cell proliferation in the gill tissue. The gill is a primary tissue, which encounters the environment and intakes oxygen, regulates osmosis and ions, and exports nitrogenous waste in fish [15,33,34]. Therefore, the genes that are important for heat tolerance in fish might be expressed at these organs. In this study, we found the suppression of apoptotic and related immune pathways; taken together, the HSP genes were shown to be highly expressed in both gill and adipose fin tissues of the HT group. HSPs respond to numerous stressors, especially in heat stress, so that the induction of HSPs repress apoptosis through inhibition of p53, Akt, Bid, and Apaf-1 [35]. We found that HSP70 was highly expressed in the gill tissues, and the mitochondrial stress 70 protein (GRP75) was highly expressed in the adipose fin tissues; the mitochondrial stress 70 protein is a member of the HSP70 proteins that plays an important role in refolding the mitochondrial proteins and preventing glucose deprivation, ROS accumulation, and apoptosis induction [36,37]. Moreover, NF-kappa-B inhibitor alpha (IKBA) showed a lower expression in both the gill and adipose fin tissues of the HT group, which is an inhibitor of transcription factor NF-kappaB (NF-kB) and prevents inflammatory response [38]. Some studies reported that the overexpression of HSP70 inhibited the nucleus translocation of the p65 subunit of NF-kB in in vivo models, and it might prevent the phosphorylation and degradation of IKBA [39,40]. For that reason, the up-regulation of HSPs might be important genes for the thermal tolerant yamame and other salmonids.

Heat stress induces protein unfolding, which plays an important role in promoting protein refolding by stimulating HSFs to regulate HSPs, such as HSP70 and HSP90 [10]. Additionally, HSP70 and HSP90 are the primary sensors of misfolded proteins that facilitate protein folding and prevent protein aggregation during encounters with heat stress [7,18]. Pei et al. [41] reported that HSP proteins partially returned to a normal level after a nine-week recovery from heat stress in rabbit testis. However, other studies in fish reported that the expression of HSP mRNAs gradually decreased two or more hours after heat stress [7,14]. In our study, we examined fish one week after heat exposure, as this would be a sufficient time for the fish to return to a normal condition. We found that the HSP70 was highly expressed in the HT group when we examined yamame in normal conditions one week after heat exposure. Moreover, p53 is a transcription factor that activates various target genes in the p53 signaling pathway and plays an important role in cell arrest, apoptosis, and metabolism [17,42]. Other genes expressed by heat stress, such as activator protein 1 (AP-1), CCAAT enhancer binding protein beta (CEBP), IKBA, and NF-kB, exert major effects by inducing inflammation, apoptosis, and cell survival [43]. Several studies have reported that heat stress affects glucose and lipid metabolism, which may help to maintain energy homeostasis in fish [7,44].

Besides these results, genes involved in ECM and CAMs, as well as in cell junction and adhesion, were highly abundant in the gill tissue. In particular, a number of transcripts encoding collagen, such as collagen type I alpha 1 chain (CO1A1), collagen type I alpha 2 chain (CO1A2), collagen type II alpha 1 chain (CO2A1), and collagen type IX alpha 3 chain (CO9A3), were highly expressed in the HT group. In accordance with other studies, collagen-associated genes were shown to be up-regulated in a regional trout strain at 23 °C [19]. Collagen is the main structural protein in the connective tissues and a specific target of oxidative stress [5,19]. The induction of collagen synthesis was found after chemical stress [5] and thermal stress [19] in rainbow trout. Other related genes in the ECM components, such as integrin subunit beta 5 (ITB5), laminin subunit beta 1 (LAMB1), laminin subunit beta 4 (LAMB4), and laminin subunit gamma 3 (LAMC3), were also highly expressed in the HT compared to the NT group. The regulation of the ECM components has important roles in cellular proliferation, cell growth, and cell signaling and might be associated with an increased rate of cell turnover [5]. Moreover, transforming growth factor β-1 (TGFβ1), integrin beta subunits (ITGB), and catenin beta 1 (CTNB1) were shown to have a higher expression in the HT group, compared with the NT group. These proteins are involved in focal adhesion, which regulates cell proliferation and differentiation and is associated with the regulation of the cytoskeleton during thermal stress [19,45]. Heat stress damages tissues and induces osmotic shock in the gill tissue; therefore, newly generated cytoskeletons may replace damaged tissues and restore osmotic balance in fish [12,16,43]. Additionally, the adherens junction and tight junction also play critical roles in maintaining cell architecture, since the tight junction functions to regulate paracellular transport in the gill, up-regulation of occludin (OCLN), and claudin (CLDs), and it is important for maintaining membrane fluidity during heat stress [12,46]. On the contrary, OCLN and CLDs showed lower expression in the gills of the HT group compared to the NT group in the normal condition in our result. We found that several associated genes in the ECM and cell junction pathways were highly expressed in the HT group, so that may have caused the difference in heat tolerance ability between the HT and NT yamame in the thermal stress condition. 

In the adipose fin tissue, the highly significant GO terms were annotated for cellular, single-organism, intracellular, biosynthetic, and metabolic processes, which support the KEGG pathway enrichment results. The p53 signaling pathway is a primary heat stress response involved in cell cycle, DNA repair, and initiation of apoptosis [47], which showed significance in the HT group. Overexpression of p73, a homologue of p53 that is similar in structure and function to p53, activates and suppresses the target gene [42]. p73, which is a component in the direct negative feedback loop in the p53 signaling pathway [5,17], was more highly expressed in the HT group compared to the NT group, suggesting that it may have suppressed p53 activities by repression or competition of p53 transcriptional activation. Meanwhile, thrombospondin-1 (TSP1), which is important for interfacing with stress signals to inform stress responses in tissue [44,48], was less expressed in the HT group than in the NT group. The CD82 antigen, KAI, is a member of the tetraspanin protein family that regulates cell motility, morphology, signaling, and cell survival [49,50], and this antigen was also expressed at lower levels in the HT group. Moreover, protein shisa-4 (SHSA4), which is also regulated by p53 and plays a role in p53-dependent apoptosis [51], showed a lower expression in the HT group compared to the NT group. This result suggests that the p53 signaling pathway might be suppressed in the normal condition after heat stress in the HT group. Furthermore, we found that genes of ATP-generating enzymes were expressed at an extremely high level in the HT group compared to the NT group, including phosphoglycerate kinase (PGK), ATP-dependent 6-phosphofructo-kinase (PFKAP), pyruvate dehydrogenase protein X component (ODPX), aldehyde dehydrogenase family 9 member A1-A (A9A1A), and phosphoglycerate mutase 1 (PGAM1). These proteins are important enzymes in the glycolysis pathway and PFKAP is a second key enzyme of glycolysis [52,53]. In a similar way, ODPX was expressed at 23 °C (mild heat stress) in rainbow trout [19]. In addition, we found that phosphoenolpyruvate carboxykinase cytosolic (PCKGC) was expressed in lower levels in the HT group. This gene is the first key enzyme of the gluconeogenesis pathway [54]. During heat stress, regulation of metabolism might relate to the cellular energy needed to support responses to stress and repair mechanisms [12]. By contrast, our study found that the HT group had higher glycolysis metabolism than the NT group in the normal condition, which suggests that the high-energy production may related to the heat tolerance in the HT fish. In a similar way, if we can develop the HT yamame, the nutrient requirement of the HT group might not be equal to that of the normal group.

## 5. Conclusions

This paper reports the RNA-sequencing of gills and adipose fins isolated from yamame in a normal condition a week after heat stress. We detected 2893 DEGs of the gill and 836 DEGs of the adipose fin. Our study found that the HT group showed a high expression of HSP70 and GRP75 in gill and adipose fin tissues, respectively, and a lower expression of IKBA in both tissues compared with the NT group, which may play important roles in heat tolerance in fish. Moreover, the ECM genes and some genes associated with cell junction and adhesion in gill tissues were highly expressed in the HT group; these related genes might play significant roles in recovering damaged tissues. In the adipose fin tissue, glycolysis pathway genes were more highly expressed in the HT group compared to the NT group, which may be important in returning cell activities to their normal condition. In this study, we also identified the suppression of the p53 signaling pathway in the HT group in the normal condition, which might be associated with the p73 expression. A variety of genes were differentially expressed in the normal condition in gill and adipose fin tissues between the HT and NT groups and should be related to the difference of heat tolerance ability in the heat stress condition in yamame. These findings should be useful in understanding the mechanisms of heat tolerance of the HT group, which may help to develop a heat-tolerant strain of yamame and other fish. 

## Figures and Tables

**Table 1:** Generated readings expressed by Illumina for the gill and adipose fin tissues of individuals from the HT and NT groups.

Tissue Samples	Gill		Adipose Fin	
	HT	NT	HT	NT
Clean readings	61,460,394	78,088,572	71,282,330	59,749,778
Clean bases (Gb)	6.15	7.81	7.13	5.97
Q20 (%)	97.88	97.73	97.80	97.80
GC content (%)	48.39	48.06	48.92	48.84

HT = high-temperature tolerant; NT = non-high-temperature tolerant.

**Table 2:** Top 20 up- and down-regulated DEGs gill tissues.

Up-Regulated Genes in Gill		Down-Regulated Genes in Gill	
Gene Name	log2FC	Gene Name	log2FC
Beta-taxilin	7.82	Macrophage-expressed gene 1 protein	−25.52
Translocation protein SEC62	7.60	Unknown	−24.65
Tax1-binding protein 1 homolog B	7.57	M-protein, striated muscle	−24.06
Protein OS-9	7.55	Unknown	−23.70
Vacuolar protein sorting-associated protein 26A	7.43	Transmembrane protein 54	−23.24
DnaJ homolog subfamily A member 2	7.32	LINE-1 retrotransposable element ORF2 protein	−23.16
CD276 antigen	7.27	Nascent polypeptide-associated complex subunit alpha	−22.75
Interferon-induced very large GTPase 1	7.26	Carboxy-Terminal domain RNA polymerase II polypeptide A small phosphatase 2	−22.54
ADP-ribosylation factor 4	7.21	E3 ubiquitin-protein ligase TRIM39	−22.49
Protein Mdm4	7.15	C-C motif chemokine 4	−22.36
Pyruvate dehydrogenase protein X component	7.09	Major histocompatibility complex class I-related gene protein	−22.29
Transforming acidic coiled-coil-containing protein 3	7.05	LINE-1 retrotransposable element ORF2 protein	−22.20
Eukaryotic translation initiation factor 3 subunit B	6.95	LINE-1 retrotransposable element ORF2 protein	−22.20
Unknown	6.94	Complement C1q subcomponent subunit A	−20.66
Insulin-like growth factor 2 mRNA-binding protein 3	6.91	Unknown	−9.90
E3 ubiquitin-protein ligase AMFR	6.88	H-2 class I histocompatibility antigen, K-K alpha chain	−9.48
Ribosome-binding protein 1	6.84	Interferon-induced very large GTPase 1	−9.31
FYVE, RhoGEF and PH domain-containing protein 4	6.81	Ras-related protein Rab-25	−9.29
Cytochrome b-c1 complex subunit 1	6.78	H-2 class II histocompatibility antigen gamma chain	−9.09
Unknown	6.75	Cytochrome c1, heme protein, mitochondrial	−8.98

Log2FoldChange (Log2FC) indicates the differential gene expression between the HT and NT groups.

**Table 3:** Top 20 up- and down-regulated DEGs in adipose fin tissues.

Up-Regulated Genes in Adipose Fin		Down-Regulated Genes in Adipose Fin	
Gene Name	log2FC	Gene Name	log2FC
Aquaporin-3	23.19	Keratin, type I cytoskeletal 18	−23.47
Tumor protein p73	22.72	Unknown	−22.65
Nucleolin	8.54	Mitogen-activated protein kinase 3	−22.24
ICOS ligand	8.00	Unknown	−22.16
Proliferation marker protein Ki-67	7.73	Nascent polypeptide-associated complex subunit alpha	−12.00
Unknown	7.59	Unknown	−10.42
Unknown	7.47	Unknown	−10.06
E3 ubiquitin-protein ligase RNF13	7.46	Major histocompatibility complex class I-related gene protein	−9.97
Testin	7.39	Nascent polypeptide-associated complex subunit alpha	−9.87
ADP-ribosylation factor 4	7.37	Src substrate protein p85	−9.65
Olfactomedin-like protein 2B	7.35	Macrosialin	−9.06
Septin-2	7.16	A-kinase anchor protein 12	−8.93
Mitogen-activated protein kinase kinase kinase 5	7.10	EMILIN-2	−8.86
Aldehyde dehydrogenase family 9 member A1-A	7.08	Ras-related protein Rab-25	−8.82
Unknown	7.07	Myocilin	−8.77
Adaptin ear-binding coat-associated protein 2	7.04	Annexin A4	−8.63
Unknown	6.95	Annexin A2-A	−8.58
General transcription factor II-I repeat domain-containing protein 2	6.80	Nesprin-2	−8.44
Unknown	6.79	Unknown	−8.37
Aldehyde dehydrogenase family 9 member A1-A	6.77	Cytochrome c1, heme protein, mitochondrial	−8.36

Log2FoldChange (Log2FC) indicates the differential gene expression between the HT and NT groups.

## Supplementary Materials

The following are available online at https://www.mdpi.com/2079-7737/8/2/21/s1. Figure S1: Heatmap repeatability analysis of mRNA libraries between samples using the Pearson correlation. Figure S2: PCA analysis of mRNA libraries between samples. Tables S1 and S2: List of DEGs of the gill and fin tissues in yamame. Tables S3 and S4: Significantly enriched pathways involving DEGs from the gill and fin. Tables S5, S6, and S7: List of DEGs associated with the ECM, CAMs, and cell junction and adherens in the gill. Tables S8 and S9: List of DEGs associated with glycolysis, and p53 signaling pathway in the fin.

## References

[1] Wang C., Xu Q., Bai Q., Yin J., Jia Z. (2015). Comparison of Growth Performances, Nutritional Composition in Muscle of Diploid and Triploid Masu Salmon (Oncorhynchus masou B., 1856). Turk. J. Fish Aquat. Sci..

[2] Munakata A. (2012). Migratory Behaviors in Masu Salmon (Oncorhynchus masou) and the Influence of Endocrinological Factors. Aqua BioSci. Monogr..

[3] Lu Y., Wu Z., Song Z., Xiao P., Liu Y., Zhang P., You F. (2016). Insight into the heat resistance of fish via blood: Effects of heat stress on metabolism, oxidative stress and antioxidation response of olive flounder *Paralichthys olivaceus* and turbot *Scophthalmus maximus*. Fish Shellfish Immunol..

[4] Qiang J., Bao W.J., Tao F.Y., He J., Li X.H., Xu P., Sun L.Y. (2017). The expression profiles of miRNA-mRNA of early response in genetically improved farmed tilapia (*Oreochromis niloticus*) liver by acute heat stress. Sci. Rep..

[5] Webster T.U., Santos E.M. (2015). Global transcriptomic profiling demonstrates induction of oxidative stress and of compensatory cellular stress responses in brown trout exposed to glyphosate and Roundup. BMC Genom..

[6] Ruland J. (2011). Return to homeostasis: Downregulation of NF-kB responses. Nat. Immunol..

[7] Liu Y., Ma D., Zhao C., Xiao Z., Xu S., Xiao Y., Wang Y., Liu Q., Li J. (2017). The expression pattern of hsp70 plays a critical role in thermal tolerance of marine demersal fish: Multilevel responses of *Paralichthys olivaceus* and its hybrids (*P. olivaceus*
♀
*x P. dentatus*
♂) to chronic and acute stress. Mar. Environ. Res..

[8] Ineno T., Tsuchida S., Kanda M., Watabe S. (2005). Thermal tolerance of a rainbow trout *Oncorhyncus mykiss* strain selected by high-temperature breeding. Fish Sci..

[9] Tan E., Wongwarangkana C., Kinoshita S., Suzuki Y., Oshima K., Hattori M., Ineno T., Tamaki K., Kera A., Muto K. (2012). Global gene expression analysis of gill tissues from normal and thermally selected strains of rainbow trout. Fish Sci..

[10] Verbeke P., Fonager J., Clark B.F.C., Rattan S.I.S. (2001). Heat shock response and aging: Mechanisms and applications. Cell Biol. Int..

[11] Finkel T., Holbrook N.J. (2000). Oxidants, oxidative stress and the biology of aging. Nature.

[12] Buckley B.A., Gracey A.Y., Somero G.N. (2006). The cellular response to heat stress in the goby *Gillichthys mirabilis*: A cDNA microarray and protein-level analysis. J. Exp. Biol..

[13] Tan E., Kinoshita S., Suzuki Y., Ineno T., Tamaki K., Kera A., Muto K., Yada T., Kitamura S., Asakawa S. (2016). Different gene expression profiles between normal and thermally selected strains of rainbow trout, Oncorhyncus mykiss, as revealed by comprehensive transcriptome analysis. Gene.

[14] Healy T.M., Tymchuk W.E., Osborne E.J., Schulte P.M. (2010). Heat shock response of killifish (*Fundulus heteroclitus*): Candidate gene and heterologous microarray approaches. Physiol. Genom..

[15] Liu S., Wang X., Sun F., Zhang J., Feng J., Liu H., Rajendran K.V., Sun L., Zhang Y., Jiang Y. (2013). RNA-Seq reveals expression signatures of genes involved in oxygen transport, protein synthesis, folding, and degradation in response to heat stress in catfish. Physiol. Genom..

[16] Rebl A., Verleih M., Köbis J.M., Kühn C., Wimmers K., Köllner B., Goldammer T. (2013). Transcriptome profiling of gill tissue in regionally bred and globally farmed rainbow trout strains reveals different strategies for coping with thermal stress. Mar. Biotechnol..

[17] Harris S.L., Levine A.J. (2005). The p53 pathway: Positive and negative feedback loops. Oncogene.

[18] Yu D., Zhang Z., Shen Z., Zhang C., Liu H. (2018). Regional differences in thermal adaptation of a cold-water fish Rhynchocypris oxycephalus revealed by thermal tolerance and transcriptomic responses. Sci. Rep..

[19] Verleih M., Borchel A., Krasnov A., Rebl A., Korytář T., Kühn C., Goldammer T. (2015). Impact of thermal stress on kidney-specific gene expression in farmed regional and imported rainbow trout. Mar. Biotechnol..

[20] Ojima N., Mekuchi M., Ineno T., Tamaki K., Kera A., Kinoshita S., Asakawa S., Watabe S. (2012). Differential expression of heat-shock proteins in F2 offspring from F1 hybrids produced between thermally selected and normal rainbow trout strains. Fish Sci..

[21] Li Y., Huang J., Liu Z., Zhou Y., Xia B., Wang Y., Kang Y., Wang J. (2017). Transcriptome analysis provides insights into hepatic responses to moderate heat stress in the rainbow trout. Gene.

[22] Ineno T., Tamaki K., Yamada K., Kodama R., Tsuchida S., Tan E., Kinoshita S., Muto K., Yada T., Kitamura S. (2018). Thermal tolerance of a thermally selected strain of rainbow trout Oncorhynchus mykiss and the pedigrees of its F1 and F2 generations indicated by their critical thermal maxima. Fish Sci..

[23] Huth T.J., Place S.P. (2016). Transcriptome wide analyses reveal a sustained cellular stress response in the gill tissue of *Trematomus bernacchii* after acclimation to multiple stressors. BMC Genom..

[24] Koppang E.O., Fischer U., Moore L., Tranulis M.A., Dijkstra J.M., Köllner B., Aune L., Jirillo E., Hordvik I. (2010). Salmonid T cells assemble in the thymus, spleen and in novel interbranchial lymphoid tissue. J. Anat..

[25] Madeira C., Madeira D., Diniz M.S., Cabral H.N., Vinagre C. (2017). Comparing biomarker responses during thermal acclimation: A lethal vs non-lethal approach in a tropical reef clownfish. Comp. Biochem. Physiol. A Mol. Integr. Physiol..

[26] Haas B.J., Papanicolaou A., Yassour M., Grabherr M., Blood P.D., Bowden J., Couger M.B., Eccles D., Li B., Lieber M. (2013). De novo transcript sequence reconstruction from RNA-seq using the Trinity platform for reference generation and analysis. Nat. Protoc..

[27] Bray N.L., Pimentel H., Melsted P., Pachter L. (2016). Near-optimal probabilistic RNA-seq quantification. Nat. Biotechnol..

[28] Love M.I., Huber W., Anders S. (2014). Moderated estimation of fold change and dispersion for RNA-seq data with DESeq2. Genome Biol..

[29] Xie C., Mao X., Huang J., Ding Y., Wu J., Dong S., Kong L., Gao G., Li C.Y., Wei L. (2011). KOBAS 2.0: A web server for annotation and identification of enriched pathways and diseases. Nucleic Acids Res..

[30] Kanehisa M., Furumichi M., Tanabe M., Sato Y., Morishima K. (2017). KEGG: New perspectives on genomes, pathways, diseases and drugs. Nucleic Acids Res..

[31] Wickham H. (2016). ggplots2: Elegant Graphics for Data Analysis.

[32] Warnes G.R., Bolker B., Bonebakker L., Gentleman R., Liaw W.H.A., Lumley T., Maechler M., Magnusson A., Moeller S., Schwart M. gplots: Various R Programming Tools for Plotting Data. https://cran.r-project.org/web/packages/gplots/index.html.

[33] Logan C.A., Somero G.N. (2011). Effects of thermal acclimation on transcriptional responses to acute heat stress in the eurythermal fish *Gillichthys mirabilis* (Cooper). Am. J. Physiol. Regul. Integr. Comp. Physiol..

[34] Evans D.H., Piermarini P.M., Choe K.P. (2005). The multifunctional fish gill: Dominant site of gas exchange, osmoregulation, acid-base regulation, and excretion of nitrogenous waste. Physiol. Rev..

[35] Ikwegbue P.C., Masamba P., Oyinloye B.E., Kappo A.P. (2018). Roles of heat shock proteins in apoptosis, oxidative stress, human inflammatory diseases, and cancer. Pharmaceuticals.

[36] Jubran R., Kocsis J., Garam N., Maláti E., Gombos T., Barabás L., Gráf L., Prohászka Z., Fishelson Z. (2017). Circulating mitochondrial stress 70 protein/mortalin and cytosolic Hsp70 in blood: Risk indicators in colorectal cancer. Int. J. Cancer.

[37] Qiukai K., Liu X., Liu Y., Liu W., Zuo J. (2013). Over-expression of GRP75 inhibits liver injury induced by oxidative damage. Acta Biochim. Biophys. Sin..

[38] Kalmar B., Greensmith L. (2009). Activation of the heat shock response in a primary cellular model of motoneuron neurodegeneration-evidence for neuroprotective and neurotoxic effects. Cell. Mol. Biol. Lett..

[39] Tanaka T., Shibazaki A., Ono R., Kaisho T. (2014). HSP70 mediates degradation of the p65 subunit of nuclear factor κb to inhibit inflammatory signaling. Sci. Signal.

[40] Shi Y., Tu Z., Tang D., Zhang H., Liu M., Wang K., Calderwood S.K., Xiao X. (2006). The inhibition of LPS-induced production of inflammatory cytokines by HSP70 involves inactivation of the NF-κB pathway but not the MAPK pathways. Shock.

[41] Pei Y., Wu Y., Qin Y. (2012). Effects of chronic heat stress on the expressions of heat shock proteins 60, 70, 90, A2, and HSC70 in the rabbit testis. Cell Stress Chaperones.

[42] Harms K., Nozell S., Chen X. (2004). The common and distinct target genes of the p53 family transcription factors. Cell. Mol. Life Sci..

[43] Sonna L.A., Fujita J., Gaffin S.L., Lilly C.M. (2002). Invited review: Effects of heat and cold stress on mammalian gene expression. J. Appl. Physiol..

[44] Forgati M., Kasdalski P.K., Herrerias T., Zaleski T., Machado C., Souza M., Donatti L. (2017). Effect of heat stress on the renal and branchial carbohydrate metabolism and antioxidant system of Antarctic fish. J. Comp. Physiol. B.

[45] Aedo J.E., Maldonado J., Aballai V., Estrada J.M., Molina M.B., Meneses C., Escarate C.G., Silva H., Molina A., Valdés J.A. (2015). mRNA-seq reveals skeleton muscle atrophy in response to handling stress in a marine teleost, the red cusk-eel (*Genypterus chilensis*). BMC Genom..

[46] Dokladny K., Ye D., Kennedy J.C., Moseley P.L., Ma T.Y. (2008). Cellular and molecular mechanisms of heat stress-induced up-regulation of occluding protein expression. Am. J. Pathol..

[47] Bouchama A., Aziz M.A., Mahri S.A., Gabere M.N., Dlamy M.A., Mohamand S., Abbad M.A., Hussein M. (2017). A model of exposure to extreme environmental heat uncovers the human transcriptome to heat stress. Sci. Rep..

[48] Komarova E.A., Diatchenko L., Rokhlin O.W., Hill J.E., Wang Z.J., Krivokrysenko V.I., Feinstein E., Gudkov A.V. (1998). Stress-induced secretion of growth inhibitors: A novel tumor suppressor function of p53. Oncogen.

[49] Zhu J., Liang C., Hua Y., Miao C., Zhang J., Xu A., Zhao K., Liu S., Tian Y., Dong H. (2017). The metastasis suppressor CD82/KAI1 regulates cell migration and invasion via inhibiting TGF-β1/Smad signaling in renal cell carcinoma. Oncotarget.

[50] Sridhar S.C., Miranti C.K. (2006). Tetraspanin KAI1/CD82 suppresses invasion by inhibiting integrin-dependent crosstalk with c-Met receptor and Src kinases. Oncogene.

[51] Bourdon J.C., Renzing J., Robertson P.L., Fernandes K.N., Lane D.P. (2002). Scotin, a novel p53-inducible proapoptotic protein located in the ER and the nuclear membrane. J. Cell Biol..

[52] Han H.S., Kang G., Kim J.S., Choi B.H., Koo S.H. (2016). Regulation of glucose metabolism from a liver-centric perspective. Exp. Mol. Med..

[53] Li X.B., Gu J.D., Zhou Q.H. (2015). Review of aerobic glycolysis and its key enzymes-new targets for lung cancer therapy. Thorac. Cancer.

[54] Xiong Y., Lei Q.Y., Zhao S., Guan K.L. (2011). Regulation of glycolysis and gluconeogenesis by acetylation of PKM and PEPCK. Cold Spring Harb. Symp. Quant. Biol..

