# Supplementary material for: Transcriptome Analysis of Yamame (Oncorhynchus masou) in Normal Conditions after Heat Stress
Source: Biology (Basel). 2019 Mar 29;8(2):21. doi: 10.3390/biology8020021 (PMC6628215; doi:10.3390/biology8020021)
Supplement: Supplementary file 1 [file biology-08-00021-s001.pdf]

# Transcriptome analysis of yamame (*Oncorhynchus masou*) in normal conditions after heat stress

**Waraporn Kraitavin[1], Kazutoshi Yoshitake[1], Yoji Igarashi[1], Susumu Mitsuyama[1], Shigeharu Kinoshita[1], Daisuke Kambayashi[2], Shugo Watabe[3] and Shuichi Asakawa[1*]**

[1] Graduate School of Agricultural and Life Sciences, The University of Tokyo, Bunkyo, Tokyo 113-8657, Japan; asakawa@mail.ecc.u-tokyo.ac.jp (S.A.)
[2] Kobayashi Branch, Miyazaki Prefectural Fisheries Research Institute, Kobayashi, Miyazaki 886-0005, Japan
[3] School of Marine Biosciences, Kitasato University, Minami, Sagamihara, Kanagawa 252-0313, Japan; swatabe@kitasato-u.ac.jp
* Correspondence: asakawa@mail.ecc.u-tokyo.ac.jp (S.A.)

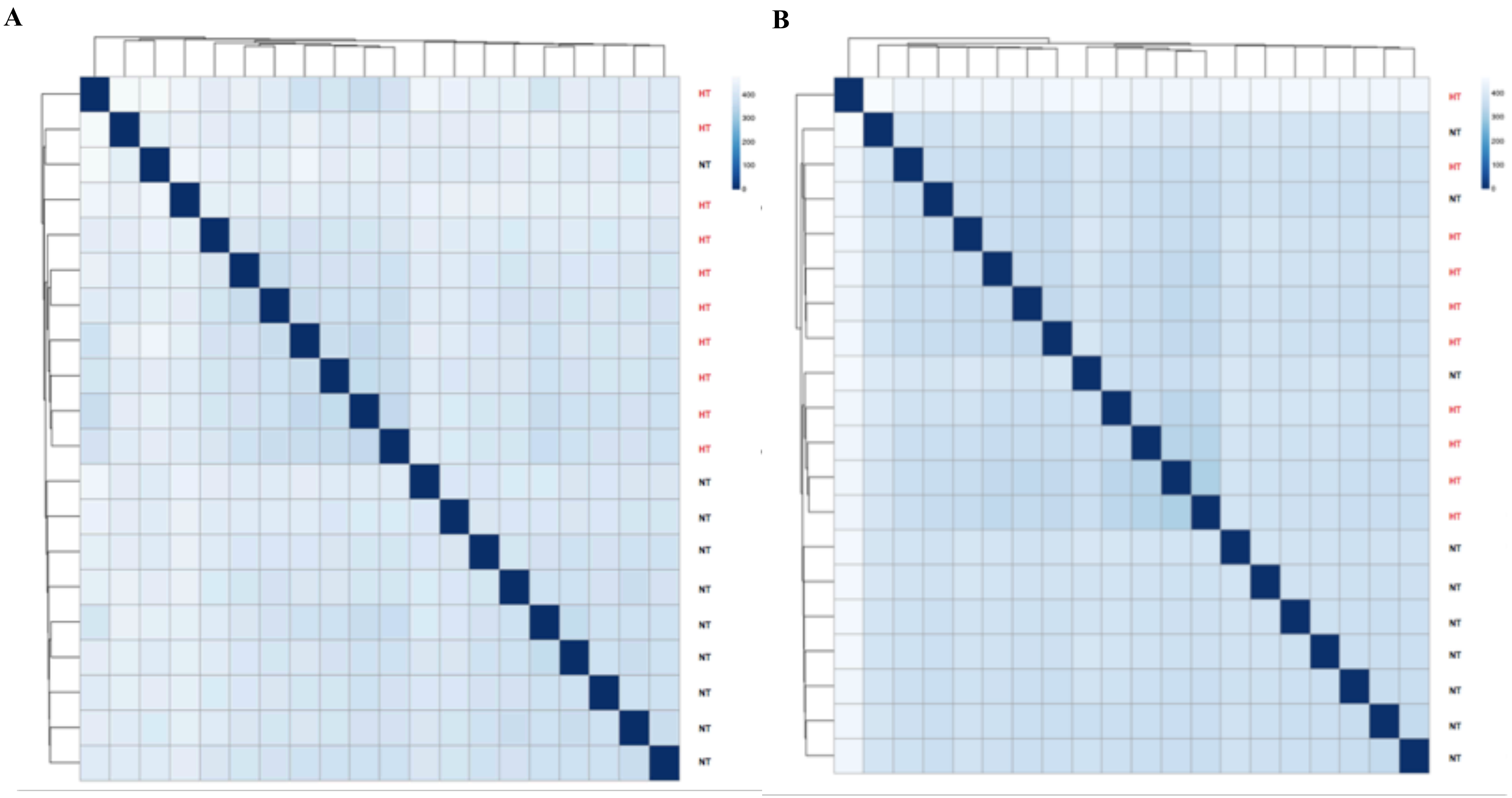


*HT represents high-temperature tolerant, NT represents non-high-temperature tolerant

**Figure S1.** The pheatmap repeatability analysis of mRNA libraries between samples using the Pearson correlation, (A) gill and (B) fin

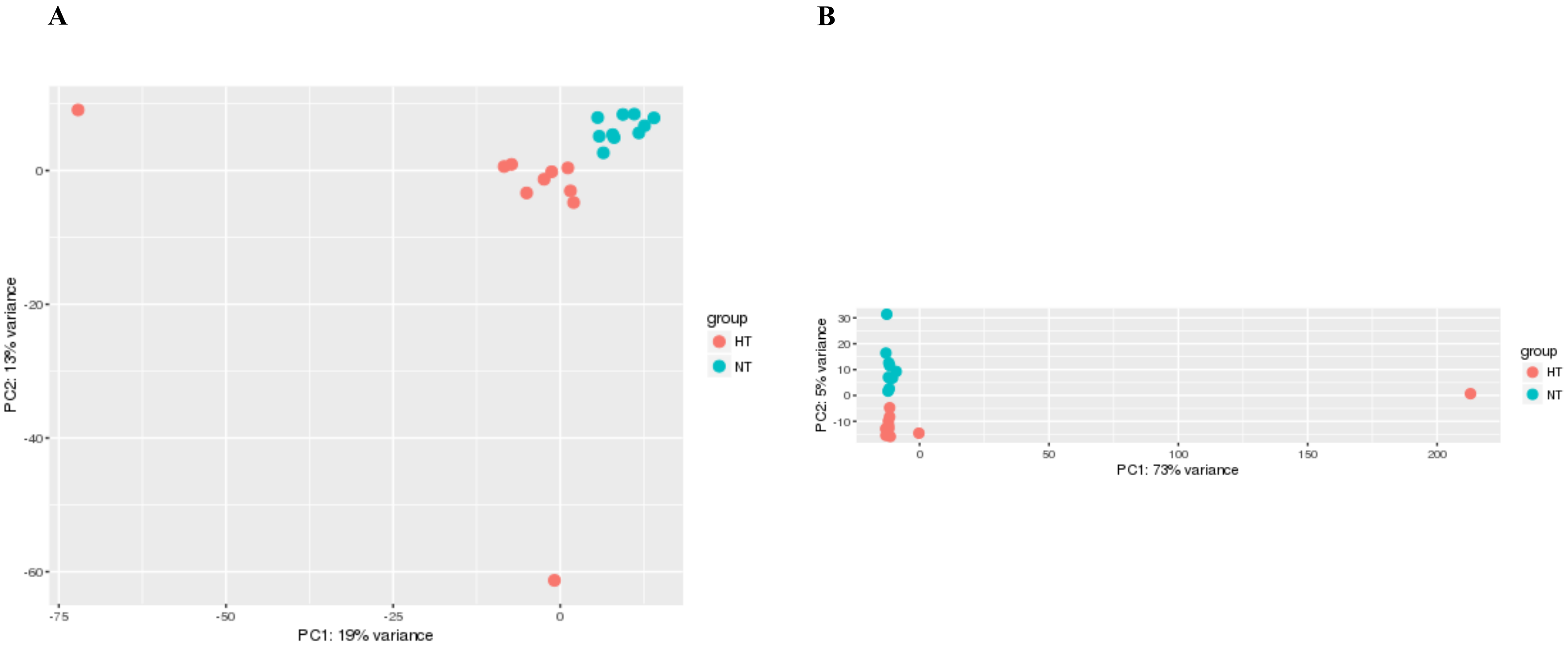


*HT represents high-temperature tolerant, NT represents non-high-temperature tolerant

**Figure S2.** The PCA analysis of mRNA libraries between samples, (A) gill and (B) fin.

**Table S1.** List of the differentially expressed genes of the gill in yamame

| Gene_id | Annotation | HT_TPM | NT_TPM | log2(FoldChange) | *P* - value |
|---|---|---|---|---|---|
| TRINITY_DN100000_c0_g1_i2 | VDAC2 | 27.57 | 17.15 | 0.71 | 0.00 |
| TRINITY_DN100006_c4_g1_i1 | | 0.65 | 2.69 | -1.93 | 0.00 |
| TRINITY_DN100021_c0_g1_i2 | CXL14 | 0.13 | 4.06 | -4.96 | 0.00 |
| TRINITY_DN100027_c4_g2_i1 | PEAK1 | 1.31 | 4.44 | -1.77 | 0.00 |
| TRINITY_DN100027_c4_g3_i1 | | 0.17 | 2.38 | -3.18 | 0.00 |
| TRINITY_DN100035_c6_g6_i2 | | 2.13 | 13.97 | -2.68 | 0.00 |
| TRINITY_DN100055_c1_g2_i1 | 3BHS | 0.22 | 1.25 | -2.46 | 0.00 |
| TRINITY_DN100058_c6_g2_i1 | OS9 | 1.73 | 0.00 | 7.55 | 0.00 |
| TRINITY_DN100064_c5_g3_i2 | | 5.39 | 15.59 | -1.53 | 0.00 |
| TRINITY_DN100065_c0_g1_i1 | | 0.25 | 2.38 | -3.38 | 0.00 |
| TRINITY_DN100077_c3_g2_i2 | | 15.42 | 30.05 | -1.01 | 0.00 |
| TRINITY_DN100087_c1_g1_i10 | CD83 | 0.20 | 2.87 | -3.57 | 0.00 |
| TRINITY_DN100087_c1_g1_i11 | CD83 | 1.59 | 12.45 | -2.98 | 0.00 |
| TRINITY_DN100087_c1_g1_i6 | CD83 | 2.19 | 7.21 | -1.75 | 0.00 |
| TRINITY_DN100087_c1_g3_i1 | | 0.18 | 1.25 | -2.70 | 0.00 |
| TRINITY_DN100103_c2_g1_i6 | | 0.00 | 2.71 | -6.39 | 0.00 |
| TRINITY_DN100111_c3_g7_i1 | | 1.64 | 7.10 | -2.08 | 0.00 |
| TRINITY_DN100114_c0_g2_i5 | SERPH | 44.88 | 24.13 | 0.88 | 0.00 |
| TRINITY_DN100121_c14_g1_i1 | | 0.17 | 7.23 | -4.32 | 0.00 |
| TRINITY_DN100121_c14_g1_i2 | | 2.76 | 20.16 | -2.78 | 0.00 |
| TRINITY_DN100126_c0_g1_i1 | | 0.02 | 2.24 | -5.23 | 0.00 |
| TRINITY_DN100131_c11_g2_i2 | | 0.88 | 3.60 | -2.06 | 0.00 |
| TRINITY_DN100132_c0_g1_i4 | TKT | 0.13 | 5.46 | -5.36 | 0.00 |
| TRINITY_DN100132_c0_g1_i6 | TKT | 1.35 | 18.77 | -3.76 | 0.00 |
| TRINITY_DN100148_c4_g1_i4 | SRGP3 | 0.87 | 3.96 | -2.27 | 0.00 |
| TRINITY_DN100150_c0_g2_i1 | TM238 | 9.67 | 24.53 | -1.36 | 0.00 |
| TRINITY_DN100155_c0_g1_i1 | TSP4B | 14.23 | 6.82 | 1.04 | 0.00 |
| TRINITY_DN100161_c3_g4_i6 | NECP2 | 2.21 | 0.01 | 6.35 | 0.00 |
| TRINITY_DN100165_c14_g2_i1 | DHI1L | 11.33 | 21.11 | -0.92 | 0.00 |
| TRINITY_DN100166_c1_g1_i9 | SH2D7 | 0.00 | 0.85 | -5.26 | 0.00 |
| TRINITY_DN100186_c4_g1_i1 | | 7.50 | 26.82 | -1.79 | 0.00 |
| TRINITY_DN100194_c0_g2_i4 | NFKB2 | 5.70 | 10.88 | -0.96 | 0.00 |
| TRINITY_DN100220_c3_g1_i2 | SEM4F | 0.85 | 1.84 | -1.12 | 0.00 |
| TRINITY_DN100226_c5_g1_i1 | TNR5 | 1.80 | 10.87 | -2.50 | 0.00 |
| TRINITY_DN100226_c5_g1_i2 | TNR5 | 1.67 | 6.18 | -1.89 | 0.00 |
| TRINITY_DN100228_c6_g1_i3 | BANK1 | 6.95 | 14.24 | -1.06 | 0.00 |
| TRINITY_DN100231_c6_g4_i8 | OCLN | 11.13 | 24.59 | -1.18 | 0.00 |
| TRINITY_DN100237_c5_g1_i1 | | 0.00 | 5.05 | -5.62 | 0.00 |
| TRINITY_DN100252_c1_g2_i6 | RGS14 | 0.39 | 1.56 | -2.03 | 0.00 |

| | | | | | |
|---|---|---|---|---|---|
| TRINITY_DN100260_c0_g1_i1 | EMIL2 | 0.01 | 4.31 | -8.77 | 0.00 |
| TRINITY_DN100281_c0_g1_i2 | | 6.13 | 23.91 | -1.98 | 0.00 |
| TRINITY_DN100304_c4_g1_i8 | LCK | 1.41 | 6.70 | -2.28 | 0.00 |
| TRINITY_DN100310_c4_g1_i1 | TOR4A | 0.23 | 2.03 | -3.03 | 0.00 |
| TRINITY_DN100320_c2_g1_i3 | | 4.54 | 13.74 | -1.67 | 0.00 |
| TRINITY_DN100333_c0_g1_i3 | UBP47 | 3.49 | 14.59 | -2.08 | 0.00 |
| TRINITY_DN100333_c0_g2_i1 | UB12B | 0.87 | 2.94 | -1.83 | 0.00 |
| TRINITY_DN100333_c0_g2_i2 | UBP5 | 0.93 | 5.42 | -2.53 | 0.00 |
| TRINITY_DN100342_c2_g1_i10 | UAP1 | 1.88 | 4.81 | -1.32 | 0.00 |
| TRINITY_DN100342_c2_g1_i13 | UAP1 | 0.44 | 0.21 | 4.68 | 0.00 |
| TRINITY_DN100344_c2_g1_i2 | | 0.11 | 3.74 | -3.98 | 0.00 |
| TRINITY_DN100380_c0_g1_i2 | | 0.00 | 11.76 | -7.84 | 0.00 |
| TRINITY_DN100380_c3_g2_i5 | | 5.63 | 2.10 | 1.42 | 0.00 |
| TRINITY_DN100403_c5_g1_i4 | | 7.50 | 33.42 | -2.09 | 0.00 |
| TRINITY_DN100403_c6_g1_i1 | LECA | 2.25 | 17.02 | -2.93 | 0.00 |
| TRINITY_DN100405_c4_g1_i1 | | 97.66 | 40.97 | 1.25 | 0.00 |
| TRINITY_DN100408_c3_g1_i2 | C1QB | 20.75 | 47.59 | -1.22 | 0.00 |
| TRINITY_DN100409_c3_g1_i2 | AGRG5 | 0.00 | 1.97 | -6.10 | 0.00 |
| TRINITY_DN100418_c2_g1_i1 | | 0.64 | 3.93 | -2.60 | 0.00 |
| TRINITY_DN100423_c0_g1_i1 | SFRP1 | 9.76 | 4.07 | 1.26 | 0.00 |
| TRINITY_DN100424_c0_g4_i2 | | 1.44 | 5.99 | -2.04 | 0.00 |
| TRINITY_DN100429_c5_g1_i2 | HMX3 | 0.02 | 2.00 | -5.79 | 0.00 |
| TRINITY_DN100429_c5_g1_i5 | HMX3 | 0.36 | 2.36 | -2.83 | 0.00 |
| TRINITY_DN100429_c5_g3_i1 | HMX3 | 0.54 | 9.88 | -3.85 | 0.00 |
| TRINITY_DN100433_c6_g1_i1 | | 3.96 | 121.43 | -4.92 | 0.00 |
| TRINITY_DN100437_c1_g2_i1 | | 15.46 | 39.59 | -1.42 | 0.00 |
| TRINITY_DN100447_c0_g1_i3 | SELO | 0.00 | 0.87 | -5.22 | 0.00 |
| TRINITY_DN100458_c0_g1_i1 | PIM1 | 6.00 | 12.61 | -1.08 | 0.00 |
| TRINITY_DN100458_c0_g1_i2 | PIM1 | 9.30 | 15.06 | -0.71 | 0.00 |
| TRINITY_DN100460_c3_g1_i2 | | 1.10 | 13.87 | -3.35 | 0.00 |
| TRINITY_DN100462_c3_g1_i2 | | 0.24 | 4.19 | -4.05 | 0.00 |
| TRINITY_DN100466_c3_g1_i14 | | 0.36 | 24.42 | -5.05 | 0.00 |
| TRINITY_DN100466_c3_g1_i15 | CUZD1 | 0.01 | 1.42 | -5.66 | 0.00 |
| TRINITY_DN100486_c3_g1_i2 | | 9.08 | 5.77 | 0.66 | 0.00 |
| TRINITY_DN100486_c4_g3_i2 | SNX9 | 0.00 | 0.73 | -3.69 | 0.00 |
| TRINITY_DN100496_c4_g2_i1 | IFI44 | 6.87 | 55.25 | -2.95 | 0.00 |
| TRINITY_DN100496_c4_g4_i1 | | 4.41 | 35.20 | -2.96 | 0.00 |
| TRINITY_DN100497_c6_g3_i1 | GIMA4 | 0.17 | 1.62 | -3.42 | 0.00 |
| TRINITY_DN100500_c0_g1_i15 | BTNLA | 7.76 | 0.31 | 4.67 | 0.00 |
| TRINITY_DN100500_c0_g1_i5 | MOG | 0.00 | 4.85 | -5.59 | 0.00 |
| TRINITY_DN100501_c5_g3_i4 | | 0.21 | 16.52 | -6.12 | 0.00 |
| TRINITY_DN100518_c3_g2_i6 | SWP70 | 2.18 | 5.73 | -1.44 | 0.00 |

| | | | | | |
|---|---|---|---|---|---|
| TRINITY_DN100519_c4_g1_i1 | | 1.05 | 5.26 | -2.42 | 0.00 |
| TRINITY_DN100526_c0_g2_i1 | MB212 | 5.14 | 2.92 | 0.82 | 0.00 |
| TRINITY_DN100532_c9_g1_i2 | K1C18 | 1.80 | 7.64 | -2.08 | 0.00 |
| TRINITY_DN100532_c9_g1_i4 | K1C18 | 0.00 | 0.92 | -5.03 | 0.00 |
| TRINITY_DN100537_c1_g2_i10 | S4A7 | 0.04 | 0.57 | -3.72 | 0.00 |
| TRINITY_DN100537_c1_g2_i5 | S4A10 | 1.76 | 3.55 | -1.03 | 0.00 |
| TRINITY_DN100541_c0_g4_i10 | FYN | 2.36 | 7.51 | -1.65 | 0.00 |
| TRINITY_DN100560_c3_g4_i1 | FUCL4 | 2.74 | 26.50 | -3.28 | 0.00 |
| TRINITY_DN100562_c1_g1_i3 | | 0.00 | 2.17 | -4.37 | 0.00 |
| TRINITY_DN100562_c1_g1_i4 | | 0.89 | 10.55 | -3.46 | 0.00 |
| TRINITY_DN100562_c1_g1_i5 | | 0.00 | 5.43 | -7.00 | 0.00 |
| TRINITY_DN100569_c1_g1_i2 | LY75 | 0.26 | 2.79 | -3.35 | 0.00 |
| TRINITY_DN100569_c1_g1_i5 | LY75 | 0.11 | 2.18 | -4.00 | 0.00 |
| TRINITY_DN100580_c9_g1_i1 | | 1.00 | 2.19 | -1.11 | 0.00 |
| TRINITY_DN100581_c0_g3_i1 | CLD6 | 0.37 | 12.10 | -5.11 | 0.00 |
| TRINITY_DN100602_c1_g1_i1 | | 3.98 | 2.45 | 0.70 | 0.00 |
| TRINITY_DN100605_c8_g1_i4 | TVA2 | 0.00 | 0.63 | -4.09 | 0.00 |
| TRINITY_DN100612_c1_g1_i7 | FBX28 | 0.00 | 1.63 | -6.68 | 0.00 |
| TRINITY_DN100613_c0_g1_i7 | KMT2C | 0.01 | 0.74 | -5.19 | 0.00 |
| TRINITY_DN100615_c0_g1_i1 | TSN8 | 92.43 | 148.41 | -0.70 | 0.00 |
| TRINITY_DN100618_c1_g1_i10 | HBA4 | 513.75 | 50.62 | 3.75 | 0.00 |
| TRINITY_DN100620_c1_g2_i10 | | 0.43 | 9.35 | -3.91 | 0.00 |
| TRINITY_DN100620_c1_g2_i4 | | 0.00 | 6.34 | -4.52 | 0.00 |
| TRINITY_DN100624_c1_g1_i1 | | 0.04 | 3.18 | -4.50 | 0.00 |
| TRINITY_DN100624_c1_g1_i10 | ITAE | 3.12 | 9.92 | -1.74 | 0.00 |
| TRINITY_DN100624_c1_g1_i7 | | 0.05 | 2.10 | -5.25 | 0.00 |
| TRINITY_DN100635_c2_g2_i1 | | 0.74 | 2.75 | -1.94 | 0.00 |
| TRINITY_DN100639_c4_g1_i4 | FNDC1 | 5.39 | 2.48 | 1.13 | 0.00 |
| TRINITY_DN100640_c1_g1_i12 | ST3A1 | 3.65 | 0.02 | 6.70 | 0.00 |
| TRINITY_DN100641_c2_g1_i3 | KGUA | 3.97 | 8.01 | -1.05 | 0.00 |
| TRINITY_DN100644_c3_g1_i2 | CD3E | 4.89 | 27.77 | -2.52 | 0.00 |
| TRINITY_DN100644_c3_g1_i5 | | 0.20 | 2.05 | -3.35 | 0.00 |
| TRINITY_DN100644_c3_g4_i1 | | 9.78 | 46.36 | -2.24 | 0.00 |
| TRINITY_DN100660_c0_g2_i4 | RLF | 0.08 | 0.75 | -2.93 | 0.00 |
| TRINITY_DN100705_c4_g1_i2 | | 4.27 | 1.05 | 1.97 | 0.00 |
| TRINITY_DN100705_c5_g1_i10 | | 0.00 | 5.76 | -6.81 | 0.00 |
| TRINITY_DN100705_c5_g1_i7 | | 0.00 | 1.52 | -4.89 | 0.00 |
| TRINITY_DN100716_c7_g2_i1 | ZCCHV | 2.41 | 15.61 | -2.68 | 0.00 |
| TRINITY_DN100716_c8_g1_i1 | | 15.55 | 8.40 | 0.86 | 0.00 |
| TRINITY_DN100730_c0_g2_i6 | MTHSD | 0.00 | 0.52 | -4.40 | 0.00 |
| TRINITY_DN100732_c2_g1_i4 | TNR5 | 0.15 | 1.03 | -2.81 | 0.00 |
| TRINITY_DN100751_c12_g1_i1 | | 0.04 | 0.87 | -4.33 | 0.00 |

| | | | | | |
|---|---|---|---|---|---|
| TRINITY_DN100754_c0_g1_i1 | AVIL | 0.93 | 7.46 | -2.99 | 0.00 |
| TRINITY_DN100754_c0_g1_i3 | AVIL | 0.90 | 6.12 | -2.75 | 0.00 |
| TRINITY_DN100754_c0_g1_i4 | AVIL | 1.31 | 8.87 | -2.81 | 0.00 |
| TRINITY_DN100754_c0_g1_i6 | AVIL | 0.05 | 2.12 | -5.30 | 0.00 |
| TRINITY_DN100755_c0_g1_i2 | BT2A2 | 0.00 | 1.20 | -4.49 | 0.00 |
| TRINITY_DN100758_c5_g1_i1 | | 50.16 | 27.30 | 0.91 | 0.00 |
| TRINITY_DN100758_c5_g1_i2 | | 47.53 | 32.35 | 0.59 | 0.00 |
| TRINITY_DN100772_c4_g3_i4 | CAPG | 0.00 | 1.59 | -4.39 | 0.00 |
| TRINITY_DN100772_c4_g3_i6 | CAPG | 7.31 | 18.03 | -1.28 | 0.00 |
| TRINITY_DN100773_c0_g2_i13 | | 0.07 | 1.42 | -3.19 | 0.00 |
| TRINITY_DN100784_c2_g1_i5 | HMR1 | 0.00 | 7.38 | -22.29 | 0.00 |
| TRINITY_DN100785_c1_g1_i3 | | 0.21 | 4.54 | -4.20 | 0.00 |
| TRINITY_DN100787_c0_g1_i6 | HTR1B | 19.96 | 5.69 | 1.06 | 0.00 |
| TRINITY_DN100796_c0_g4_i1 | | 11.88 | 3.21 | 1.93 | 0.00 |
| TRINITY_DN100809_c0_g3_i1 | | 2.05 | 24.10 | -3.60 | 0.00 |
| TRINITY_DN100826_c12_g1_i2 | | 5.44 | 13.42 | -1.31 | 0.00 |
| TRINITY_DN100851_c1_g3_i1 | | 1.53 | 4.56 | -1.63 | 0.00 |
| TRINITY_DN100851_c1_g5_i1 | | 0.00 | 5.48 | -4.12 | 0.00 |
| TRINITY_DN100859_c2_g7_i2 | 5NT3 | 8.82 | 4.22 | 1.07 | 0.00 |
| TRINITY_DN100862_c1_g1_i10 | | 0.31 | 3.50 | -3.59 | 0.00 |
| TRINITY_DN100862_c1_g1_i11 | AGRG3 | 0.13 | 2.02 | -4.05 | 0.00 |
| TRINITY_DN100862_c1_g1_i12 | | 0.06 | 0.87 | -3.43 | 0.00 |
| TRINITY_DN100862_c1_g1_i20 | AGRG3 | 0.00 | 2.80 | -7.87 | 0.00 |
| TRINITY_DN100862_c1_g1_i21 | AGRG4 | 0.00 | 0.69 | -5.68 | 0.00 |
| TRINITY_DN100862_c1_g1_i5 | AGRG3 | 0.10 | 1.36 | -3.92 | 0.00 |
| TRINITY_DN100875_c8_g5_i2 | | 1.10 | 10.19 | -3.19 | 0.00 |
| TRINITY_DN100887_c8_g1_i1 | | 3.65 | 16.12 | -2.18 | 0.00 |
| TRINITY_DN100899_c0_g1_i3 | ARRD2 | 14.51 | 24.68 | -0.77 | 0.00 |
| TRINITY_DN100899_c0_g1_i7 | ARRD2 | 21.99 | 45.76 | -1.07 | 0.00 |
| TRINITY_DN100905_c1_g1_i1 | BPI | 14.21 | 38.28 | -1.44 | 0.00 |
| TRINITY_DN100933_c1_g1_i3 | VWDE | 0.67 | 0.06 | 3.48 | 0.00 |
| TRINITY_DN100936_c0_g1_i3 | M4K3 | 5.65 | 24.41 | -2.13 | 0.00 |
| TRINITY_DN100939_c0_g1_i4 | | 25.89 | 2.68 | 1.65 | 0.00 |
| TRINITY_DN100947_c11_g1_i11 | VWA7 | 0.30 | 3.81 | -3.65 | 0.00 |
| TRINITY_DN100947_c11_g1_i6 | VWA7 | 0.00 | 0.75 | -4.66 | 0.00 |
| TRINITY_DN100950_c0_g1_i6 | STA13 | 0.00 | 0.62 | -4.33 | 0.00 |
| TRINITY_DN100961_c0_g1_i7 | FGFP2 | 2.96 | 0.54 | 2.45 | 0.00 |
| TRINITY_DN100972_c0_g1_i9 | VSI10 | 0.00 | 1.24 | -6.12 | 0.00 |
| TRINITY_DN100984_c3_g1_i2 | CNN2 | 8.25 | 4.42 | 0.88 | 0.00 |
| TRINITY_DN100991_c2_g1_i2 | | 4.19 | 11.38 | -1.41 | 0.00 |
| TRINITY_DN100991_c3_g2_i4 | IN80C | 0.00 | 1.27 | -5.00 | 0.00 |
| TRINITY_DN101001_c6_g1_i1 | | 0.58 | 6.48 | -3.32 | 0.00 |

| | | | | | |
|---|---|---|---|---|---|
| TRINITY_DN101001_c6_g2_i1 | TREF1 | 0.28 | 1.20 | -2.07 | 0.00 |
| TRINITY_DN101019_c0_g1_i12 | SSFA2 | 0.02 | 0.62 | -5.09 | 0.00 |
| TRINITY_DN101021_c5_g1_i1 | | 0.83 | 3.37 | -2.02 | 0.00 |
| TRINITY_DN101022_c0_g1_i1 | RIN3 | 0.28 | 1.94 | -2.79 | 0.00 |
| TRINITY_DN101029_c0_g1_i17 | F177A | 0.08 | 3.11 | -5.14 | 0.00 |
| TRINITY_DN101036_c1_g1_i3 | RL4B | 12.52 | 1.36 | 3.39 | 0.00 |
| TRINITY_DN101036_c1_g1_i4 | RL4B | 0.00 | 5.52 | -7.21 | 0.00 |
| TRINITY_DN101039_c0_g1_i11 | TXLNB | 0.09 | 3.00 | -5.17 | 0.00 |
| TRINITY_DN101039_c0_g1_i3 | TXLNB | 4.48 | 0.02 | 7.82 | 0.00 |
| TRINITY_DN101064_c3_g1_i3 | ODPX | 2.29 | 0.00 | 7.09 | 0.00 |
| TRINITY_DN101082_c6_g1_i1 | RASF5 | 1.26 | 3.19 | -1.33 | 0.00 |
| TRINITY_DN101082_c6_g2_i2 | RASF5 | 0.89 | 2.68 | -1.58 | 0.00 |
| TRINITY_DN101097_c0_g1_i7 | PKHG3 | 0.00 | 0.64 | -4.63 | 0.00 |
| TRINITY_DN101100_c2_g3_i1 | | 0.23 | 3.86 | -3.73 | 0.00 |
| TRINITY_DN101103_c5_g1_i1 | RN223 | 0.35 | 1.86 | -2.47 | 0.00 |
| TRINITY_DN101103_c8_g2_i2 | | 2.09 | 0.08 | 4.07 | 0.00 |
| TRINITY_DN101105_c2_g2_i12 | | 0.23 | 3.57 | -3.81 | 0.00 |
| TRINITY_DN101105_c2_g2_i6 | | 1.43 | 6.54 | -2.21 | 0.00 |
| TRINITY_DN101110_c5_g1_i3 | TRI25 | 1.89 | 15.57 | -3.07 | 0.00 |
| TRINITY_DN101113_c1_g1_i1 | ANKL2 | 0.00 | 7.81 | -4.25 | 0.00 |
| TRINITY_DN101114_c3_g1_i1 | | 0.10 | 1.60 | -3.35 | 0.00 |
| TRINITY_DN101116_c1_g1_i7 | I13R2 | 2.42 | 4.68 | -0.91 | 0.00 |
| TRINITY_DN101127_c4_g2_i2 | | 2.02 | 10.43 | -2.38 | 0.00 |
| TRINITY_DN101129_c0_g1_i1 | C1GLT | 0.10 | 3.56 | -4.35 | 0.00 |
| TRINITY_DN101129_c0_g1_i2 | C1GLT | 1.25 | 14.70 | -3.59 | 0.00 |
| TRINITY_DN101129_c0_g3_i1 | | 0.83 | 7.81 | -3.30 | 0.00 |
| TRINITY_DN101135_c4_g1_i14 | LIPO | 444.07 | 144.53 | 1.69 | 0.00 |
| TRINITY_DN101135_c4_g1_i16 | LIPO | 41.73 | 3.11 | 3.99 | 0.00 |
| TRINITY_DN101135_c4_g1_i18 | | 5.01 | 6.38 | -3.45 | 0.00 |
| TRINITY_DN101137_c5_g1_i2 | GSTA | 97.43 | 54.20 | 0.82 | 0.00 |
| TRINITY_DN101149_c0_g1_i1 | GPR34 | 0.62 | 2.26 | -1.89 | 0.00 |
| TRINITY_DN101149_c0_g2_i1 | GPR34 | 0.29 | 1.50 | -2.32 | 0.00 |
| TRINITY_DN101151_c2_g1_i2 | | 0.00 | 4.31 | -7.12 | 0.00 |
| TRINITY_DN101167_c8_g1_i1 | LYSC2 | 118.31 | 490.99 | -2.09 | 0.00 |
| TRINITY_DN101167_c8_g1_i2 | LYSC2 | 8.55 | 39.04 | -2.21 | 0.00 |
| TRINITY_DN101184_c11_g1_i5 | SLAF8 | 0.17 | 1.69 | -3.21 | 0.00 |
| TRINITY_DN101193_c11_g2_i2 | | 0.00 | 19.11 | -8.37 | 0.00 |
| TRINITY_DN101193_c11_g2_i3 | | 0.20 | 8.81 | -5.38 | 0.00 |
| TRINITY_DN101193_c11_g3_i1 | | 0.05 | 8.88 | -6.81 | 0.00 |
| TRINITY_DN101195_c2_g2_i1 | | 0.99 | 4.76 | -2.39 | 0.00 |
| TRINITY_DN101196_c1_g1_i1 | | 0.05 | 1.28 | -4.78 | 0.00 |
| TRINITY_DN101196_c1_g1_i10 | GPSM2 | 10.44 | 20.41 | -1.00 | 0.00 |

| | | | | | |
|---|---|---|---|---|---|
| TRINITY_DN101196_c1_g1_i5 | GPSM1 | 8.42 | 21.64 | -1.41 | 0.00 |
| TRINITY_DN101196_c1_g2_i1 | | 8.94 | 21.48 | -1.29 | 0.00 |
| TRINITY_DN101210_c7_g1_i2 | | 2.43 | 0.05 | 3.89 | 0.00 |
| TRINITY_DN101211_c0_g1_i1 | IRF3 | 1.97 | 5.22 | -1.47 | 0.00 |
| TRINITY_DN101215_c1_g2_i2 | NACA | 0.02 | 6.64 | -22.75 | 0.00 |
| TRINITY_DN101230_c2_g1_i8 | | 10.46 | 28.41 | -1.46 | 0.00 |
| TRINITY_DN101230_c2_g1_i9 | IAN1 | 13.05 | 49.42 | -1.91 | 0.00 |
| TRINITY_DN101230_c2_g4_i1 | | 8.10 | 43.45 | -2.45 | 0.00 |
| TRINITY_DN101230_c2_g4_i2 | | 0.18 | 1.75 | -3.19 | 0.00 |
| TRINITY_DN101230_c2_g5_i1 | | 2.24 | 5.80 | -1.41 | 0.00 |
| TRINITY_DN101232_c0_g1_i1 | | 2.11 | 13.85 | -2.68 | 0.00 |
| TRINITY_DN101236_c0_g1_i3 | GMIP | 0.49 | 3.13 | -2.69 | 0.00 |
| TRINITY_DN101240_c3_g4_i1 | PNPH | 0.99 | 2.88 | -1.56 | 0.00 |
| TRINITY_DN101242_c5_g1_i2 | CY1 | 0.00 | 18.70 | -8.98 | 0.00 |
| TRINITY_DN101242_c5_g1_i6 | CY1 | 84.36 | 44.63 | 0.93 | 0.00 |
| TRINITY_DN101267_c4_g1_i11 | AGRG3 | 0.16 | 1.81 | -3.38 | 0.00 |
| TRINITY_DN101267_c4_g1_i13 | AGRG3 | 0.06 | 4.21 | -6.29 | 0.00 |
| TRINITY_DN101267_c4_g1_i15 | | 1.62 | 11.60 | -2.75 | 0.00 |
| TRINITY_DN101267_c4_g1_i7 | AGRG3 | 0.15 | 3.23 | -4.47 | 0.00 |
| TRINITY_DN101272_c2_g1_i1 | PR38B | 0.00 | 1.51 | -6.49 | 0.00 |
| TRINITY_DN101283_c11_g1_i5 | F177A | 0.78 | 2.20 | -1.51 | 0.00 |
| TRINITY_DN101294_c1_g3_i1 | | 0.78 | 4.22 | -2.45 | 0.00 |
| TRINITY_DN101294_c1_g5_i3 | TCA | 8.91 | 44.96 | -2.36 | 0.00 |
| TRINITY_DN101327_c1_g1_i4 | PGH2 | 0.70 | 2.53 | -1.90 | 0.00 |
| TRINITY_DN101345_c6_g1_i1 | TM234 | 0.00 | 1.55 | -5.44 | 0.00 |
| TRINITY_DN101346_c1_g1_i9 | IGHM | 0.03 | 1.30 | -4.98 | 0.00 |
| TRINITY_DN101359_c3_g3_i4 | RHG12 | 0.00 | 3.30 | -6.23 | 0.00 |
| TRINITY_DN101368_c2_g1_i7 | SH3K1 | 4.12 | 9.03 | -1.15 | 0.00 |
| TRINITY_DN101371_c1_g1_i1 | | 1.42 | 10.03 | -2.81 | 0.00 |
| TRINITY_DN101371_c1_g1_i2 | | 0.69 | 13.94 | -3.99 | 0.00 |
| TRINITY_DN101375_c6_g2_i1 | U119A | 0.77 | 2.63 | -1.80 | 0.00 |
| TRINITY_DN101377_c5_g1_i2 | | 0.00 | 8.47 | -4.39 | 0.00 |
| TRINITY_DN101383_c2_g2_i1 | | 0.68 | 2.57 | -1.89 | 0.00 |
| TRINITY_DN101389_c7_g1_i1 | CDO1 | 2.77 | 100.09 | -5.07 | 0.00 |
| TRINITY_DN101389_c7_g1_i2 | CDO1 | 0.05 | 1.30 | -4.33 | 0.00 |
| TRINITY_DN101389_c7_g1_i4 | CDO1 | 0.07 | 3.94 | -5.81 | 0.00 |
| TRINITY_DN101397_c4_g4_i1 | RHOG | 6.21 | 15.75 | -1.37 | 0.00 |
| TRINITY_DN101399_c2_g2_i2 | ZBED4 | 0.00 | 2.53 | -6.48 | 0.00 |
| TRINITY_DN101409_c4_g7_i10 | PXDN | 0.78 | 7.13 | -3.22 | 0.00 |
| TRINITY_DN101409_c4_g7_i3 | | 2.31 | 14.80 | -2.76 | 0.00 |
| TRINITY_DN101438_c1_g1_i4 | AB1IP | 0.36 | 1.20 | -1.70 | 0.00 |
| TRINITY_DN101464_c9_g1_i4 | TCB2 | 0.50 | 4.04 | -2.91 | 0.00 |

| | | | | | |
|---|---|---|---|---|---|
| TRINITY_DN101476_c0_g1_i1 | WASP | 3.46 | 9.23 | -1.41 | 0.00 |
| TRINITY_DN101478_c0_g1_i9 | 3BP2 | 0.33 | 1.24 | -1.94 | 0.00 |
| TRINITY_DN101481_c2_g1_i4 | SYSC | 11.20 | 21.47 | -0.92 | 0.00 |
| TRINITY_DN101486_c2_g1_i11 | | 1.24 | 0.23 | 2.36 | 0.00 |
| TRINITY_DN101486_c2_g2_i1 | | 110.83 | 18.37 | 2.48 | 0.00 |
| TRINITY_DN101488_c1_g1_i3 | PTPRC | 11.90 | 28.64 | -1.30 | 0.00 |
| TRINITY_DN101489_c1_g1_i6 | PLCL1 | 0.43 | 0.00 | 5.89 | 0.00 |
| TRINITY_DN101496_c4_g3_i1 | | 1.16 | 3.73 | -1.69 | 0.00 |
| TRINITY_DN101530_c1_g1_i8 | VMP1 | 0.01 | 0.85 | -4.26 | 0.00 |
| TRINITY_DN101545_c10_g2_i5 | | 0.00 | 0.58 | -4.99 | 0.00 |
| TRINITY_DN101546_c0_g1_i2 | ALS | 0.21 | 1.30 | -2.54 | 0.00 |
| TRINITY_DN101554_c1_g4_i1 | SELN | 7.74 | 4.92 | 0.62 | 0.00 |
| TRINITY_DN101571_c2_g5_i1 | HELZ2 | 0.22 | 1.83 | -3.01 | 0.00 |
| TRINITY_DN101580_c0_g1_i6 | MGT4A | 0.11 | 0.96 | -3.10 | 0.00 |
| TRINITY_DN101590_c2_g1_i7 | CSK22 | 0.66 | 0.03 | 4.53 | 0.00 |
| TRINITY_DN101593_c0_g1_i2 | LAYN | 0.98 | 0.05 | 4.24 | 0.00 |
| TRINITY_DN101596_c1_g2_i1 | IL17 | 0.34 | 3.09 | -3.15 | 0.00 |
| TRINITY_DN101599_c4_g1_i3 | URGCP | 0.00 | 0.54 | -4.85 | 0.00 |
| TRINITY_DN101608_c1_g2_i1 | FCGR4 | 0.23 | 1.93 | -2.88 | 0.00 |
| TRINITY_DN101610_c5_g5_i1 | | 0.14 | 6.10 | -4.40 | 0.00 |
| TRINITY_DN101617_c7_g1_i1 | AEBP1 | 11.25 | 5.64 | 0.99 | 0.00 |
| TRINITY_DN101659_c1_g1_i2 | PRDM1 | 0.89 | 4.88 | -2.45 | 0.00 |
| TRINITY_DN101660_c5_g2_i1 | | 36.43 | 189.00 | -2.46 | 0.00 |
| TRINITY_DN101661_c6_g1_i5 | | 0.19 | 5.06 | -4.90 | 0.00 |
| TRINITY_DN101681_c1_g1_i1 | LXN | 9.96 | 4.55 | 1.11 | 0.00 |
| TRINITY_DN101693_c3_g2_i1 | IL6RB | 0.14 | 1.96 | -3.85 | 0.00 |
| TRINITY_DN101693_c5_g1_i1 | | 18.54 | 6.27 | 1.58 | 0.00 |
| TRINITY_DN101694_c0_g1_i10 | SEC62 | 0.48 | 0.00 | 5.24 | 0.00 |
| TRINITY_DN101694_c0_g1_i12 | SEC62 | 2.01 | 0.00 | 7.60 | 0.00 |
| TRINITY_DN101700_c14_g1_i1 | | 1.43 | 37.42 | -4.24 | 0.00 |
| TRINITY_DN101704_c3_g1_i3 | CD28 | 1.39 | 6.83 | -2.29 | 0.00 |
| TRINITY_DN101704_c3_g1_i5 | CD28 | 0.98 | 3.65 | -1.93 | 0.00 |
| TRINITY_DN101704_c3_g1_i9 | CD28 | 0.81 | 3.50 | -2.09 | 0.00 |
| TRINITY_DN101706_c2_g1_i1 | | 0.12 | 13.05 | -6.24 | 0.00 |
| TRINITY_DN101714_c0_g1_i2 | | 102.77 | 354.12 | -1.76 | 0.00 |
| TRINITY_DN101714_c0_g2_i1 | | 3.92 | 14.03 | -1.89 | 0.00 |
| TRINITY_DN101726_c0_g3_i1 | RDH7 | 18.05 | 8.85 | 1.04 | 0.00 |
| TRINITY_DN101726_c0_g3_i3 | RDH2 | 4.48 | 0.93 | 2.24 | 0.00 |
| TRINITY_DN101735_c1_g4_i1 | | 45.62 | 175.66 | -1.91 | 0.00 |
| TRINITY_DN101741_c0_g1_i1 | URP2 | 0.68 | 3.58 | -2.35 | 0.00 |
| TRINITY_DN101741_c1_g2_i3 | TRPT1 | 0.33 | 3.18 | -3.30 | 0.00 |
| TRINITY_DN101746_c6_g1_i1 | | 0.09 | 3.12 | -3.79 | 0.00 |

| | | | | | |
|---|---|---|---|---|---|
| TRINITY_DN101747_c0_g2_i5 | PARP8 | 0.10 | 1.05 | -3.59 | 0.00 |
| TRINITY_DN101758_c3_g3_i1 | SHIP1 | 2.25 | 8.03 | -1.76 | 0.00 |
| TRINITY_DN101825_c1_g1_i2 | | 2.58 | 5.27 | -1.02 | 0.00 |
| TRINITY_DN101852_c0_g1_i4 | FGOP2 | 0.77 | 0.03 | 4.59 | 0.00 |
| TRINITY_DN101854_c5_g4_i2 | | 0.16 | 1.02 | -2.56 | 0.00 |
| TRINITY_DN101862_c0_g3_i9 | | 0.58 | 5.19 | -3.16 | 0.00 |
| TRINITY_DN101899_c8_g4_i1 | | 12.85 | 0.26 | 4.57 | 0.00 |
| TRINITY_DN101901_c0_g1_i3 | CNTN1 | 23.72 | 56.33 | -1.27 | 0.00 |
| TRINITY_DN101901_c0_g2_i1 | | 306.47 | 630.06 | -1.07 | 0.00 |
| TRINITY_DN101903_c1_g1_i15 | LYN | 2.43 | 6.23 | -1.40 | 0.00 |
| TRINITY_DN101909_c3_g4_i2 | | 0.85 | 14.22 | -4.06 | 0.00 |
| TRINITY_DN101912_c5_g1_i1 | TRFR | 0.13 | 0.78 | -2.49 | 0.00 |
| TRINITY_DN101918_c1_g1_i2 | PRRX1 | 9.52 | 2.12 | 0.99 | 0.00 |
| TRINITY_DN101923_c3_g1_i2 | PSD4 | 0.78 | 2.62 | -1.75 | 0.00 |
| TRINITY_DN101928_c1_g3_i1 | ZFAN6 | 0.73 | 7.85 | -3.68 | 0.00 |
| TRINITY_DN101932_c4_g3_i1 | | 0.72 | 2.07 | -1.52 | 0.00 |
| TRINITY_DN101933_c0_g3_i1 | | 12.31 | 34.27 | -1.50 | 0.00 |
| TRINITY_DN101943_c1_g1_i1 | | 6.79 | 0.43 | 4.21 | 0.00 |
| TRINITY_DN101947_c0_g3_i3 | RRBP1 | 0.99 | 0.00 | 6.84 | 0.00 |
| TRINITY_DN101955_c0_g1_i11 | ELF1 | 0.00 | 2.63 | -7.58 | 0.00 |
| TRINITY_DN101955_c0_g1_i3 | ELF1 | 0.00 | 0.47 | -5.18 | 0.00 |
| TRINITY_DN101959_c5_g1_i3 | | 0.05 | 2.26 | -5.41 | 0.00 |
| TRINITY_DN101966_c3_g4_i1 | P2RX1 | 2.49 | 15.59 | -2.59 | 0.00 |
| TRINITY_DN101966_c3_g9_i1 | BTLA | 0.20 | 1.25 | -3.03 | 0.00 |
| TRINITY_DN101974_c1_g1_i15 | | 3.71 | 12.08 | -1.73 | 0.00 |
| TRINITY_DN101974_c1_g1_i5 | | 2.70 | 14.72 | -2.42 | 0.00 |
| TRINITY_DN101974_c1_g1_i8 | | 1.23 | 0.01 | 4.59 | 0.00 |
| TRINITY_DN101974_c1_g2_i2 | | 1.33 | 36.33 | -4.78 | 0.00 |
| TRINITY_DN101982_c6_g1_i1 | | 5.12 | 2.78 | 0.88 | 0.00 |
| TRINITY_DN101987_c2_g1_i2 | | 0.11 | 1.78 | -3.94 | 0.00 |
| TRINITY_DN101999_c2_g1_i14 | | 0.05 | 3.16 | -5.66 | 0.00 |
| TRINITY_DN101999_c2_g1_i8 | | 0.06 | 1.56 | -5.11 | 0.00 |
| TRINITY_DN102001_c1_g1_i1 | | 9.22 | 51.54 | -2.43 | 0.00 |
| TRINITY_DN102004_c7_g1_i3 | TLR13 | 1.94 | 3.22 | -0.74 | 0.00 |
| TRINITY_DN102017_c1_g2_i2 | TBCD1 | 0.08 | 1.25 | -3.90 | 0.00 |
| TRINITY_DN102031_c5_g1_i11 | RABX5 | 0.01 | 1.83 | -6.24 | 0.00 |
| TRINITY_DN102039_c0_g1_i11 | HAUS6 | 0.06 | 0.95 | -3.90 | 0.00 |
| TRINITY_DN102042_c5_g2_i5 | CD2AP | 0.19 | 5.61 | -4.84 | 0.00 |
| TRINITY_DN102045_c4_g1_i2 | FER | 0.01 | 1.20 | -6.52 | 0.00 |
| TRINITY_DN102073_c4_g1_i3 | | 1.95 | 0.13 | 3.73 | 0.00 |
| TRINITY_DN102073_c4_g1_i4 | TBFG | 34.23 | 7.93 | 1.19 | 0.00 |
| TRINITY_DN102081_c4_g1_i1 | RHG30 | 0.86 | 3.89 | -2.26 | 0.00 |

| | | | | | |
|---|---|---|---|---|---|
| TRINITY_DN102081_c4_g2_i1 | RHG30 | 1.20 | 4.16 | -1.81 | 0.00 |
| TRINITY_DN102082_c1_g3_i4 | SH3L3 | 10.73 | 21.11 | -0.97 | 0.00 |
| TRINITY_DN102083_c4_g1_i12 | RHG32 | 0.15 | 1.89 | -3.83 | 0.00 |
| TRINITY_DN102091_c0_g1_i1 | MUC5B | 0.40 | 16.91 | -5.24 | 0.00 |
| TRINITY_DN102091_c0_g1_i10 | | 1.73 | 70.26 | -5.38 | 0.00 |
| TRINITY_DN102091_c0_g1_i11 | | 2.70 | 131.16 | -5.53 | 0.00 |
| TRINITY_DN102091_c0_g1_i13 | | 0.34 | 21.35 | -5.72 | 0.00 |
| TRINITY_DN102091_c0_g1_i15 | | 1.00 | 46.20 | -5.37 | 0.00 |
| TRINITY_DN102091_c0_g1_i16 | | 1.08 | 19.66 | -4.15 | 0.00 |
| TRINITY_DN102091_c0_g1_i18 | | 0.00 | 37.26 | -6.35 | 0.00 |
| TRINITY_DN102091_c0_g1_i20 | MUC5B | 0.18 | 16.03 | -6.36 | 0.00 |
| TRINITY_DN102091_c0_g1_i24 | | 0.15 | 26.79 | -7.14 | 0.00 |
| TRINITY_DN102091_c0_g1_i27 | | 2.51 | 105.44 | -5.24 | 0.00 |
| TRINITY_DN102091_c0_g1_i3 | | 0.00 | 29.61 | -5.66 | 0.00 |
| TRINITY_DN102091_c0_g1_i4 | | 0.00 | 34.92 | -6.11 | 0.00 |
| TRINITY_DN102091_c0_g1_i5 | | 0.39 | 14.45 | -3.93 | 0.00 |
| TRINITY_DN102091_c0_g1_i6 | | 0.00 | 2.23 | -4.61 | 0.00 |
| TRINITY_DN102091_c0_g1_i7 | | 1.72 | 51.64 | -4.79 | 0.00 |
| TRINITY_DN102091_c0_g1_i8 | | 1.02 | 64.30 | -6.07 | 0.00 |
| TRINITY_DN102106_c0_g4_i1 | | 29.56 | 85.38 | -1.61 | 0.00 |
| TRINITY_DN102107_c3_g1_i3 | | 4.05 | 17.10 | -1.99 | 0.00 |
| TRINITY_DN102120_c2_g1_i6 | | 6.22 | 17.26 | -1.46 | 0.00 |
| TRINITY_DN102129_c0_g4_i1 | | 2.98 | 13.29 | -2.09 | 0.00 |
| TRINITY_DN102132_c1_g3_i1 | | 6.17 | 2.76 | 1.12 | 0.00 |
| TRINITY_DN102136_c0_g2_i3 | | 32.55 | 89.86 | -1.49 | 0.00 |
| TRINITY_DN102147_c3_g1_i1 | NUB1 | 1.69 | 14.72 | -3.12 | 0.00 |
| TRINITY_DN102155_c0_g1_i4 | PPTC7 | 0.04 | 0.82 | -4.14 | 0.00 |
| TRINITY_DN102161_c3_g1_i1 | TNF14 | 0.54 | 4.90 | -3.15 | 0.00 |
| TRINITY_DN102161_c3_g1_i3 | TNF14 | 0.24 | 1.81 | -2.77 | 0.00 |
| TRINITY_DN102161_c3_g1_i4 | TNF14 | 1.06 | 4.98 | -2.23 | 0.00 |
| TRINITY_DN102161_c3_g3_i1 | | 0.38 | 12.51 | -4.27 | 0.00 |
| TRINITY_DN102170_c0_g1_i4 | DNS2A | 7.74 | 15.07 | -0.97 | 0.00 |
| TRINITY_DN102170_c0_g1_i5 | DNS2A | 7.02 | 16.43 | -1.26 | 0.00 |
| TRINITY_DN102170_c0_g3_i1 | DNS2A | 9.42 | 23.88 | -1.34 | 0.00 |
| TRINITY_DN102171_c0_g1_i1 | TRI69 | 0.88 | 0.00 | 5.75 | 0.00 |
| TRINITY_DN102184_c4_g3_i3 | C1GLT | 2.11 | 17.08 | -3.07 | 0.00 |
| TRINITY_DN102184_c4_g3_i5 | | 0.00 | 4.68 | -4.87 | 0.00 |
| TRINITY_DN102187_c10_g1_i1 | | 7.03 | 27.58 | -1.91 | 0.00 |
| TRINITY_DN102198_c6_g3_i1 | | 0.07 | 1.86 | -3.88 | 0.00 |
| TRINITY_DN102203_c2_g1_i1 | SPT13 | 2.87 | 0.22 | 3.71 | 0.00 |
| TRINITY_DN102203_c2_g1_i8 | SPT13 | 0.00 | 1.61 | -6.66 | 0.00 |
| TRINITY_DN102205_c0_g2_i11 | WASP | 0.22 | 4.31 | -4.38 | 0.00 |

| | | | | | |
|---|---|---|---|---|---|
| TRINITY_DN102205_c0_g2_i6 | WASP | 0.04 | 1.66 | -5.26 | 0.00 |
| TRINITY_DN102205_c0_g2_i7 | WASL | 0.28 | 3.20 | -3.55 | 0.00 |
| TRINITY_DN102208_c3_g1_i1 | ESPN | 0.60 | 1.87 | -1.62 | 0.00 |
| TRINITY_DN102225_c3_g1_i8 | | 0.00 | 2.81 | -6.59 | 0.00 |
| TRINITY_DN102239_c7_g1_i5 | FIS1 | 0.00 | 3.56 | -6.38 | 0.00 |
| TRINITY_DN102241_c0_g1_i15 | HCFC1 | 0.10 | 1.82 | -4.07 | 0.00 |
| TRINITY_DN102242_c0_g1_i4 | | 0.24 | 2.98 | -3.30 | 0.00 |
| TRINITY_DN102242_c0_g4_i1 | | 0.84 | 20.57 | -4.66 | 0.00 |
| TRINITY_DN102242_c0_g6_i1 | | 0.00 | 3.68 | -6.11 | 0.00 |
| TRINITY_DN102265_c1_g1_i1 | | 0.77 | 14.34 | -4.09 | 0.00 |
| TRINITY_DN102280_c4_g1_i7 | | 0.00 | 2.04 | -7.39 | 0.00 |
| TRINITY_DN102317_c12_g1_i1 | | 0.88 | 4.36 | -2.26 | 0.00 |
| TRINITY_DN102318_c3_g5_i5 | | 0.11 | 3.35 | -4.87 | 0.00 |
| TRINITY_DN102319_c6_g1_i1 | LAMC3 | 2.17 | 0.94 | 1.19 | 0.00 |
| TRINITY_DN102324_c6_g1_i3 | SAMD9 | 2.25 | 5.14 | -1.19 | 0.00 |
| TRINITY_DN102336_c4_g4_i1 | | 0.26 | 1.14 | -1.95 | 0.00 |
| TRINITY_DN102347_c1_g1_i1 | | 0.05 | 9.14 | -7.33 | 0.00 |
| TRINITY_DN102355_c4_g2_i3 | LCAP | 1.69 | 4.44 | -1.40 | 0.00 |
| TRINITY_DN102360_c8_g3_i2 | TPM4 | 42.82 | 26.36 | 0.73 | 0.00 |
| TRINITY_DN102362_c1_g3_i3 | | 0.32 | 6.52 | -4.14 | 0.00 |
| TRINITY_DN102362_c1_g5_i3 | OST4 | 0.00 | 14.41 | -7.90 | 0.00 |
| TRINITY_DN102372_c3_g3_i1 | TMC7 | 0.91 | 4.02 | -2.18 | 0.00 |
| TRINITY_DN102376_c2_g1_i1 | | 0.73 | 0.03 | 4.36 | 0.00 |
| TRINITY_DN102376_c2_g3_i1 | | 5.03 | 11.30 | -1.21 | 0.00 |
| TRINITY_DN102376_c2_g5_i1 | | 1.08 | 15.27 | -3.78 | 0.00 |
| TRINITY_DN102379_c1_g2_i1 | T106B | 7.90 | 3.76 | 1.08 | 0.00 |
| TRINITY_DN102385_c9_g1_i4 | C4S2A | 19.20 | 33.85 | -0.83 | 0.00 |
| TRINITY_DN102389_c3_g5_i1 | | 1.33 | 3.69 | -1.47 | 0.00 |
| TRINITY_DN102389_c3_g5_i2 | | 52.03 | 148.23 | -1.44 | 0.00 |
| TRINITY_DN102391_c4_g1_i2 | MORC3 | 0.12 | 8.22 | -6.11 | 0.00 |
| TRINITY_DN102413_c1_g2_i7 | POLY | 0.08 | 0.81 | -3.45 | 0.00 |
| TRINITY_DN102420_c0_g1_i7 | ANO8 | 0.12 | 1.69 | -3.80 | 0.00 |
| TRINITY_DN102427_c0_g1_i6 | FXC1A | 3.24 | 5.61 | -0.80 | 0.00 |
| TRINITY_DN102440_c1_g6_i10 | ABCB9 | 6.22 | 15.96 | -1.40 | 0.00 |
| TRINITY_DN102456_c6_g1_i2 | | 0.22 | 2.76 | -3.44 | 0.00 |
| TRINITY_DN102456_c6_g2_i10 | AGRG7 | 0.00 | 1.66 | -4.84 | 0.00 |
| TRINITY_DN102458_c4_g1_i17 | DEN1B | 0.71 | 3.26 | -2.18 | 0.00 |
| TRINITY_DN102458_c4_g1_i18 | DEN1B | 0.25 | 1.22 | -2.38 | 0.00 |
| TRINITY_DN102468_c0_g3_i13 | RIPK2 | 3.38 | 6.89 | -1.04 | 0.00 |
| TRINITY_DN102485_c2_g1_i2 | CD3Z | 7.74 | 20.37 | -1.43 | 0.00 |
| TRINITY_DN102496_c0_g2_i2 | DCSTP | 0.08 | 0.79 | -3.07 | 0.00 |
| TRINITY_DN102499_c0_g1_i5 | | 0.00 | 0.96 | -5.95 | 0.00 |

| | | | | | |
|---|---|---|---|---|---|
| TRINITY_DN102501_c1_g1_i5 | UB2G1 | 0.54 | 0.00 | 4.94 | 0.00 |
| TRINITY_DN102502_c2_g1_i7 | LEG9 | 5.24 | 14.49 | -1.50 | 0.00 |
| TRINITY_DN102505_c7_g1_i11 | LCP2 | 1.56 | 4.67 | -1.61 | 0.00 |
| TRINITY_DN102507_c0_g1_i1 | | 1.73 | 64.25 | -4.95 | 0.00 |
| TRINITY_DN102512_c1_g2_i5 | PA24A | 0.00 | 0.57 | -5.01 | 0.00 |
| TRINITY_DN102515_c4_g2_i13 | PDLI7 | 0.00 | 1.81 | -5.12 | 0.00 |
| TRINITY_DN102527_c1_g1_i10 | | 0.72 | 2.95 | -1.99 | 0.00 |
| TRINITY_DN102528_c2_g1_i2 | KANL1 | 0.26 | 1.87 | -2.76 | 0.00 |
| TRINITY_DN102536_c4_g1_i1 | | 22.84 | 7.97 | 1.52 | 0.00 |
| TRINITY_DN102542_c12_g1_i2 | CYGB1 | 10.23 | 5.52 | 0.87 | 0.00 |
| TRINITY_DN102546_c5_g1_i1 | | 17.45 | 9.87 | 0.86 | 0.00 |
| TRINITY_DN102555_c0_g1_i1 | | 23.74 | 11.28 | 1.07 | 0.00 |
| TRINITY_DN102556_c0_g5_i3 | | 1.51 | 4.44 | -1.57 | 0.00 |
| TRINITY_DN102559_c3_g3_i1 | | 1.25 | 17.48 | -3.74 | 0.00 |
| TRINITY_DN102570_c2_g1_i11 | S39A7 | 0.65 | 1.58 | -8.01 | 0.00 |
| TRINITY_DN102590_c0_g1_i1 | | 2.79 | 6.30 | -1.16 | 0.00 |
| TRINITY_DN102596_c5_g1_i2 | TDX | 58.98 | 94.29 | -0.69 | 0.00 |
| TRINITY_DN102598_c2_g1_i1 | | 0.53 | 2.01 | -1.93 | 0.00 |
| TRINITY_DN102610_c0_g2_i2 | DAB2 | 1.63 | 0.11 | 3.85 | 0.00 |
| TRINITY_DN102610_c0_g3_i1 | DAB2 | 4.75 | 2.02 | 1.26 | 0.00 |
| TRINITY_DN102616_c5_g3_i1 | | 9.98 | 21.66 | -1.15 | 0.00 |
| TRINITY_DN102636_c0_g1_i1 | | 0.24 | 3.25 | -3.49 | 0.00 |
| TRINITY_DN102642_c2_g3_i10 | IL2RB | 0.11 | 2.33 | -4.36 | 0.00 |
| TRINITY_DN102642_c2_g3_i8 | IL2RB | 0.23 | 2.01 | -3.00 | 0.00 |
| TRINITY_DN102667_c0_g1_i2 | | 11.55 | 23.32 | -1.01 | 0.00 |
| TRINITY_DN102669_c5_g1_i12 | IKBZ | 1.67 | 11.97 | -2.68 | 0.00 |
| TRINITY_DN102669_c5_g1_i13 | IKBZ | 0.76 | 4.21 | -2.44 | 0.00 |
| TRINITY_DN102669_c5_g1_i15 | IKBZ | 0.13 | 0.81 | -2.74 | 0.00 |
| TRINITY_DN102669_c5_g1_i19 | IKBZ | 1.10 | 7.31 | -2.83 | 0.00 |
| TRINITY_DN102669_c5_g1_i7 | IKBZ | 0.00 | 2.12 | -5.03 | 0.00 |
| TRINITY_DN102675_c5_g1_i4 | | 0.37 | 6.19 | -3.59 | 0.00 |
| TRINITY_DN102678_c1_g1_i3 | | 2.48 | 4.86 | -0.98 | 0.00 |
| TRINITY_DN102692_c1_g2_i2 | | 0.74 | 0.00 | 5.67 | 0.00 |
| TRINITY_DN102704_c3_g2_i1 | | 0.00 | 2.46 | -4.54 | 0.00 |
| TRINITY_DN102710_c6_g3_i1 | | 1.46 | 10.23 | -2.79 | 0.00 |
| TRINITY_DN102717_c11_g1_i1 | CR1 | 21.26 | 10.54 | 1.05 | 0.00 |
| TRINITY_DN102724_c0_g1_i12 | IN35 | 6.21 | 12.45 | -1.03 | 0.00 |
| TRINITY_DN102724_c0_g1_i6 | IN35 | 2.01 | 6.17 | -1.59 | 0.00 |
| TRINITY_DN102730_c1_g1_i1 | | 0.66 | 9.47 | -3.55 | 0.00 |
| TRINITY_DN102752_c4_g1_i3 | | 1.20 | 19.63 | -3.96 | 0.00 |
| TRINITY_DN102762_c2_g2_i8 | PP4R1 | 0.02 | 1.26 | -5.90 | 0.00 |
| TRINITY_DN102816_c0_g2_i2 | LUM | 60.00 | 31.46 | 0.94 | 0.00 |

| | | | | | |
|---|---|---|---|---|---|
| TRINITY_DN102819_c5_g1_i14 | TRI39 | 57.53 | 104.98 | -0.92 | 0.00 |
| TRINITY_DN102819_c5_g1_i16 | TRI39 | 0.00 | 12.44 | -22.49 | 0.00 |
| TRINITY_DN102819_c5_g1_i6 | | 0.10 | 1.41 | -3.56 | 0.00 |
| TRINITY_DN102822_c3_g1_i4 | ADRM1 | 0.00 | 0.70 | -4.52 | 0.00 |
| TRINITY_DN102842_c2_g1_i12 | MX | 0.00 | 0.52 | -4.90 | 0.00 |
| TRINITY_DN102842_c2_g1_i17 | MX2 | 0.31 | 4.14 | -3.71 | 0.00 |
| TRINITY_DN102842_c2_g1_i19 | MX2 | 0.00 | 4.94 | -7.67 | 0.00 |
| TRINITY_DN102842_c2_g1_i5 | MX2 | 0.06 | 3.33 | -4.40 | 0.00 |
| TRINITY_DN102842_c2_g1_i6 | MX2 | 0.14 | 2.50 | -4.28 | 0.00 |
| TRINITY_DN102842_c2_g1_i8 | MX | 0.02 | 0.89 | -5.00 | 0.00 |
| TRINITY_DN102852_c1_g3_i1 | | 0.22 | 7.71 | -4.08 | 0.00 |
| TRINITY_DN102863_c3_g1_i4 | | 13.90 | 41.17 | -1.56 | 0.00 |
| TRINITY_DN102863_c3_g1_i6 | LITAF | 13.31 | 27.16 | -1.07 | 0.00 |
| TRINITY_DN102864_c0_g1_i4 | ITB1A | 15.38 | 42.01 | -1.48 | 0.00 |
| TRINITY_DN102864_c0_g2_i6 | ITB5 | 1.47 | 0.73 | 1.04 | 0.00 |
| TRINITY_DN102866_c3_g3_i6 | FRIH | 1989.08 | 1266.13 | 0.71 | 0.00 |
| TRINITY_DN102907_c0_g1_i4 | | 0.00 | 1.74 | -4.54 | 0.00 |
| TRINITY_DN102921_c3_g1_i11 | COR1A | 12.53 | 36.23 | -1.51 | 0.00 |
| TRINITY_DN102921_c3_g1_i2 | COR1A | 10.34 | 38.97 | -1.92 | 0.00 |
| TRINITY_DN102921_c3_g1_i4 | COR1A | 7.04 | 15.56 | -1.12 | 0.00 |
| TRINITY_DN102937_c5_g2_i1 | | 2.41 | 0.20 | 3.74 | 0.00 |
| TRINITY_DN102954_c2_g1_i2 | SC65 | 3.70 | 1.72 | 1.12 | 0.00 |
| TRINITY_DN102965_c8_g1_i1 | | 3.10 | 7.68 | -1.35 | 0.00 |
| TRINITY_DN102965_c8_g2_i1 | | 1.48 | 3.96 | -1.45 | 0.00 |
| TRINITY_DN102971_c1_g3_i1 | | 2.43 | 7.28 | -1.60 | 0.00 |
| TRINITY_DN102974_c0_g1_i1 | AOXC | 0.15 | 2.66 | -3.66 | 0.00 |
| TRINITY_DN102992_c2_g1_i1 | TP8L2 | 1.46 | 4.41 | -1.58 | 0.00 |
| TRINITY_DN102992_c2_g1_i4 | TP8L2 | 2.09 | 4.15 | -1.01 | 0.00 |
| TRINITY_DN103000_c4_g1_i1 | PSA2 | 63.74 | 110.96 | -0.80 | 0.00 |
| TRINITY_DN103025_c1_g1_i3 | | 0.68 | 2.88 | -2.17 | 0.00 |
| TRINITY_DN103038_c0_g2_i2 | CHD2 | 0.33 | 2.85 | -3.16 | 0.00 |
| TRINITY_DN103048_c3_g1_i2 | | 13.01 | 32.65 | -1.32 | 0.00 |
| TRINITY_DN103057_c1_g1_i3 | | 1.16 | 10.23 | -3.13 | 0.00 |
| TRINITY_DN103060_c0_g1_i2 | CR3L2 | 0.00 | 0.68 | -5.53 | 0.00 |
| TRINITY_DN103065_c7_g1_i1 | F213A | 2.84 | 0.76 | 1.99 | 0.00 |
| TRINITY_DN103098_c0_g1_i1 | DHE3 | 15.71 | 8.02 | 0.95 | 0.00 |
| TRINITY_DN103098_c0_g2_i2 | | 0.00 | 10.30 | -23.70 | 0.00 |
| TRINITY_DN103099_c8_g1_i2 | | 0.86 | 9.48 | -3.17 | 0.00 |
| TRINITY_DN103102_c5_g1_i2 | | 4.40 | 20.03 | -2.11 | 0.00 |
| TRINITY_DN103110_c4_g1_i5 | | 0.60 | 7.93 | -3.55 | 0.00 |
| TRINITY_DN103113_c1_g1_i11 | RN121 | 3.54 | 0.61 | 2.60 | 0.00 |
| TRINITY_DN103113_c1_g1_i14 | RN121 | 0.03 | 0.51 | -3.98 | 0.00 |

| | | | | | |
|---|---|---|---|---|---|
| TRINITY_DN103113_c1_g1_i15 | RN121 | 0.04 | 0.57 | -3.71 | 0.00 |
| TRINITY_DN103116_c0_g1_i1 | NEMF | 0.00 | 1.30 | -6.69 | 0.00 |
| TRINITY_DN103118_c3_g1_i4 | SAT1 | 0.92 | 7.65 | -2.95 | 0.00 |
| TRINITY_DN103118_c3_g3_i1 | | 88.75 | 145.34 | -0.71 | 0.00 |
| TRINITY_DN103126_c4_g1_i1 | | 8.93 | 44.15 | -2.35 | 0.00 |
| TRINITY_DN103126_c4_g6_i1 | | 34.52 | 16.78 | 1.04 | 0.00 |
| TRINITY_DN103175_c6_g1_i3 | P2RY8 | 0.47 | 1.50 | -1.62 | 0.00 |
| TRINITY_DN103186_c6_g2_i1 | | 5.79 | 16.12 | -1.48 | 0.00 |
| TRINITY_DN103192_c2_g1_i1 | | 0.29 | 2.90 | -3.67 | 0.00 |
| TRINITY_DN103193_c2_g1_i2 | | 1.59 | 5.44 | -1.77 | 0.00 |
| TRINITY_DN103203_c1_g1_i7 | NFS1 | 31.57 | 22.13 | 0.50 | 0.00 |
| TRINITY_DN103207_c5_g1_i2 | EIF3B | 5.72 | 0.00 | 6.95 | 0.00 |
| TRINITY_DN103212_c0_g1_i9 | FLT3 | 0.28 | 3.59 | -3.69 | 0.00 |
| TRINITY_DN103219_c0_g1_i1 | FKB10 | 8.19 | 2.74 | 1.58 | 0.00 |
| TRINITY_DN103219_c0_g1_i2 | FKB10 | 4.10 | 1.58 | 1.39 | 0.00 |
| TRINITY_DN103219_c0_g1_i3 | FKB10 | 23.08 | 15.23 | 0.58 | 0.00 |
| TRINITY_DN103233_c4_g1_i4 | UBP21 | 0.07 | 1.75 | -3.98 | 0.00 |
| TRINITY_DN103236_c0_g1_i3 | CD6 | 0.00 | 0.63 | -5.17 | 0.00 |
| TRINITY_DN103236_c0_g1_i4 | CD6 | 0.02 | 0.70 | -4.96 | 0.00 |
| TRINITY_DN103236_c0_g1_i7 | CD6 | 0.02 | 1.30 | -5.58 | 0.00 |
| TRINITY_DN103236_c0_g1_i9 | CD6 | 0.42 | 1.79 | -2.10 | 0.00 |
| TRINITY_DN103236_c0_g3_i1 | C163A | 0.96 | 8.10 | -2.90 | 0.00 |
| TRINITY_DN103239_c2_g1_i15 | ENDD1 | 0.06 | 2.24 | -3.64 | 0.00 |
| TRINITY_DN103239_c2_g1_i17 | ENDD1 | 0.65 | 25.88 | -5.44 | 0.00 |
| TRINITY_DN103240_c0_g1_i11 | MUC2L | 0.05 | 5.49 | -6.55 | 0.00 |
| TRINITY_DN103240_c0_g1_i13 | MUC2L | 13.94 | 0.20 | 6.12 | 0.00 |
| TRINITY_DN103240_c0_g1_i16 | MUC2L | 12.45 | 0.52 | 4.58 | 0.00 |
| TRINITY_DN103240_c0_g1_i18 | MUC2 | 2.98 | 123.37 | -5.38 | 0.00 |
| TRINITY_DN103240_c0_g1_i7 | MUC5A | 0.00 | 2.37 | -7.13 | 0.00 |
| TRINITY_DN103250_c1_g1_i22 | CNOT2 | 0.35 | 0.00 | 4.99 | 0.00 |
| TRINITY_DN103252_c13_g1_i1 | C3AR | 0.19 | 1.78 | -3.30 | 0.00 |
| TRINITY_DN103256_c3_g1_i2 | SE1BA | 0.00 | 1.56 | -6.36 | 0.00 |
| TRINITY_DN103280_c1_g2_i3 | CYAA | 1.32 | 6.17 | -2.24 | 0.00 |
| TRINITY_DN103285_c0_g1_i6 | BORG5 | 1.21 | 0.40 | 1.55 | 0.00 |
| TRINITY_DN103290_c2_g1_i1 | RFXAP | 0.00 | 1.49 | -5.13 | 0.00 |
| TRINITY_DN103291_c11_g2_i1 | | 1.02 | 4.32 | -2.10 | 0.00 |
| TRINITY_DN103302_c2_g2_i2 | NCF1 | 2.74 | 10.14 | -1.95 | 0.00 |
| TRINITY_DN103302_c2_g5_i2 | NCF1 | 0.47 | 1.64 | -1.95 | 0.00 |
| TRINITY_DN103304_c8_g1_i1 | TC1A | 0.38 | 3.60 | -3.04 | 0.00 |
| TRINITY_DN103325_c0_g1_i1 | | 3.93 | 2.38 | 0.72 | 0.00 |
| TRINITY_DN103335_c1_g1_i16 | | 0.23 | 1.94 | -3.22 | 0.00 |
| TRINITY_DN103343_c1_g2_i5 | WLS | 13.55 | 8.08 | 0.73 | 0.00 |

| | | | | | |
|---|---|---|---|---|---|
| TRINITY_DN103349_c0_g1_i2 | CRLD2 | 3.06 | 1.37 | 1.13 | 0.00 |
| TRINITY_DN103349_c0_g1_i4 | CRLD2 | 2.68 | 1.48 | 0.86 | 0.00 |
| TRINITY_DN103351_c7_g1_i2 | GIMA4 | 0.12 | 5.18 | -5.65 | 0.00 |
| TRINITY_DN103353_c10_g1_i1 | PTN18 | 3.08 | 7.37 | -1.30 | 0.00 |
| TRINITY_DN103359_c0_g1_i2 | IRF8 | 2.57 | 5.59 | -1.15 | 0.00 |
| TRINITY_DN103378_c2_g3_i3 | | 0.19 | 1.63 | -3.18 | 0.00 |
| TRINITY_DN103378_c2_g4_i5 | NF7O | 0.06 | 4.49 | -4.32 | 0.00 |
| TRINITY_DN103381_c0_g3_i1 | | 1.34 | 4.19 | -1.63 | 0.00 |
| TRINITY_DN103383_c4_g1_i2 | TAGL | 146.03 | 69.31 | 1.08 | 0.00 |
| TRINITY_DN103408_c2_g2_i1 | GVIN1 | 0.01 | 1.62 | -6.42 | 0.00 |
| TRINITY_DN103408_c2_g2_i6 | GVIN1 | 0.00 | 9.73 | -9.31 | 0.00 |
| TRINITY_DN103416_c0_g1_i3 | MSMB | 7.84 | 12.98 | -0.76 | 0.00 |
| TRINITY_DN103425_c4_g1_i1 | IL7RA | 0.22 | 4.25 | -4.26 | 0.00 |
| TRINITY_DN103425_c4_g1_i5 | IL7RA | 0.00 | 0.57 | -5.04 | 0.00 |
| TRINITY_DN103425_c4_g1_i7 | IL7RA | 2.37 | 6.15 | -1.42 | 0.00 |
| TRINITY_DN103435_c1_g1_i1 | | 203.74 | 782.13 | -1.97 | 0.00 |
| TRINITY_DN103435_c1_g2_i4 | HG2A | 0.09 | 46.16 | -9.09 | 0.00 |
| TRINITY_DN103435_c1_g2_i5 | HG2A | 336.34 | 885.00 | -1.36 | 0.00 |
| TRINITY_DN103435_c1_g2_i7 | HG2A | 0.00 | 1.40 | -6.07 | 0.00 |
| TRINITY_DN103435_c1_g3_i1 | | 0.00 | 13.29 | -4.80 | 0.00 |
| TRINITY_DN103435_c1_g6_i1 | | 0.51 | 7.08 | -3.89 | 0.00 |
| TRINITY_DN103435_c1_g8_i2 | HG2A | 15.71 | 57.46 | -1.88 | 0.00 |
| TRINITY_DN103439_c5_g1_i1 | | 3.83 | 32.03 | -3.13 | 0.00 |
| TRINITY_DN103442_c0_g1_i1 | BICL1 | 0.86 | 2.00 | -1.21 | 0.00 |
| TRINITY_DN103446_c0_g1_i8 | RAB5A | 0.00 | 0.80 | -4.33 | 0.00 |
| TRINITY_DN103447_c1_g2_i4 | | 0.11 | 2.17 | -4.00 | 0.00 |
| TRINITY_DN103448_c5_g1_i2 | | 1.05 | 0.33 | 1.68 | 0.00 |
| TRINITY_DN103448_c6_g2_i3 | ARH40 | 0.42 | 0.05 | 2.83 | 0.00 |
| TRINITY_DN103452_c3_g7_i1 | | 0.12 | 0.99 | -3.32 | 0.00 |
| TRINITY_DN103453_c6_g3_i1 | | 0.18 | 2.95 | -3.46 | 0.00 |
| TRINITY_DN103461_c8_g2_i2 | STAG2 | 1.83 | 0.02 | 6.65 | 0.00 |
| TRINITY_DN103471_c10_g2_i1 | | 2.79 | 7.99 | -1.48 | 0.00 |
| TRINITY_DN103478_c6_g1_i1 | | 5.47 | 13.90 | -1.35 | 0.00 |
| TRINITY_DN103478_c6_g2_i1 | | 4.32 | 8.77 | -1.02 | 0.00 |
| TRINITY_DN103488_c3_g1_i2 | | 0.48 | 1.19 | -1.30 | 0.00 |
| TRINITY_DN103491_c4_g1_i1 | AGRG4 | 2.22 | 17.51 | -2.97 | 0.00 |
| TRINITY_DN103491_c4_g1_i2 | AGRG2 | 0.00 | 1.23 | -4.91 | 0.00 |
| TRINITY_DN103491_c7_g1_i1 | AGRG6 | 1.26 | 15.17 | -3.35 | 0.00 |
| TRINITY_DN103503_c1_g1_i1 | I17RE | 0.41 | 2.14 | -2.43 | 0.00 |
| TRINITY_DN103503_c1_g2_i1 | | 0.84 | 6.57 | -2.91 | 0.00 |
| TRINITY_DN103503_c1_g3_i1 | | 0.54 | 9.35 | -3.67 | 0.00 |
| TRINITY_DN103513_c0_g1_i5 | LOX5 | 1.65 | 7.02 | -2.07 | 0.00 |

| | | | | | |
|---|---|---|---|---|---|
| TRINITY_DN103513_c0_g1_i7 | LOX5 | 0.17 | 2.72 | -3.91 | 0.00 |
| TRINITY_DN103513_c0_g4_i1 | LOX5 | 3.48 | 28.36 | -2.84 | 0.00 |
| TRINITY_DN103517_c2_g1_i1 | HBB1 | 146.47 | 26.67 | 2.74 | 0.00 |
| TRINITY_DN103517_c2_g1_i2 | HBB1 | 62.94 | 6.05 | 3.66 | 0.00 |
| TRINITY_DN103526_c3_g1_i1 | | 7.86 | 15.72 | -1.01 | 0.00 |
| TRINITY_DN103533_c7_g1_i1 | | 16.31 | 101.94 | -2.70 | 0.00 |
| TRINITY_DN103534_c4_g1_i8 | ACAP2 | 0.85 | 2.88 | -1.85 | 0.00 |
| TRINITY_DN103540_c10_g1_i3 | | 3.59 | 27.25 | -3.10 | 0.00 |
| TRINITY_DN103541_c1_g1_i1 | | 0.41 | 1.76 | -2.11 | 0.00 |
| TRINITY_DN103541_c4_g4_i1 | | 21.24 | 48.94 | -1.21 | 0.00 |
| TRINITY_DN103543_c0_g1_i2 | LRMP | 1.33 | 3.12 | -1.23 | 0.00 |
| TRINITY_DN103547_c2_g1_i17 | | 6.54 | 0.00 | 5.40 | 0.00 |
| TRINITY_DN103547_c2_g1_i20 | | 4.55 | 19.05 | -2.22 | 0.00 |
| TRINITY_DN103553_c0_g1_i2 | CCR9 | 1.57 | 3.90 | -1.33 | 0.00 |
| TRINITY_DN103555_c0_g2_i7 | NSD2 | 0.00 | 0.39 | -4.62 | 0.00 |
| TRINITY_DN103561_c10_g1_i6 | TNR5 | 0.00 | 2.88 | -6.40 | 0.00 |
| TRINITY_DN103569_c0_g2_i1 | GPR25 | 0.42 | 2.93 | -2.83 | 0.00 |
| TRINITY_DN103586_c0_g1_i7 | R213A | 2.65 | 6.44 | -1.29 | 0.00 |
| TRINITY_DN103598_c5_g2_i1 | | 7.06 | 1.64 | 2.13 | 0.00 |
| TRINITY_DN103608_c6_g1_i1 | LORF2 | 0.00 | 2.79 | -23.16 | 0.00 |
| TRINITY_DN103608_c6_g1_i5 | LORF2 | 0.00 | 1.42 | -22.20 | 0.00 |
| TRINITY_DN103608_c6_g1_i6 | LORF2 | 0.00 | 1.41 | -22.20 | 0.00 |
| TRINITY_DN103616_c0_g3_i5 | CCL19 | 1.00 | 15.03 | -3.91 | 0.00 |
| TRINITY_DN103620_c0_g1_i3 | SAMD9 | 0.01 | 0.60 | -5.88 | 0.00 |
| TRINITY_DN103647_c0_g2_i1 | | 5.76 | 0.00 | 4.72 | 0.00 |
| TRINITY_DN103654_c0_g1_i1 | CCD80 | 3.37 | 1.31 | 1.42 | 0.00 |
| TRINITY_DN103656_c2_g1_i4 | RAB6A | 1.49 | 0.15 | 3.24 | 0.00 |
| TRINITY_DN103666_c3_g1_i2 | HCK | 0.67 | 2.71 | -1.99 | 0.00 |
| TRINITY_DN103666_c3_g1_i3 | HCK | 7.59 | 30.25 | -2.05 | 0.00 |
| TRINITY_DN103666_c3_g2_i3 | HCK | 2.32 | 19.11 | -3.17 | 0.00 |
| TRINITY_DN103672_c2_g1_i10 | PAXI | 1.85 | 0.15 | 3.73 | 0.00 |
| TRINITY_DN103673_c5_g1_i1 | | 0.92 | 5.43 | -2.64 | 0.00 |
| TRINITY_DN103682_c1_g1_i6 | PDPR | 0.88 | 0.01 | 5.79 | 0.00 |
| TRINITY_DN103684_c1_g1_i1 | SEPT6 | 1.45 | 5.69 | -1.91 | 0.00 |
| TRINITY_DN103688_c3_g1_i1 | AKA12 | 0.00 | 1.24 | -7.06 | 0.00 |
| TRINITY_DN103717_c0_g1_i1 | CAD11 | 7.03 | 1.88 | 1.91 | 0.00 |
| TRINITY_DN103717_c2_g2_i4 | TOX2 | 0.04 | 1.29 | -4.60 | 0.00 |
| TRINITY_DN103727_c3_g1_i2 | | 13.51 | 0.48 | 4.96 | 0.00 |
| TRINITY_DN103729_c0_g1_i1 | | 1.49 | 17.66 | -3.56 | 0.00 |
| TRINITY_DN103729_c0_g1_i13 | | 0.08 | 4.15 | -4.16 | 0.00 |
| TRINITY_DN103729_c0_g1_i18 | | 0.09 | 1.35 | -3.84 | 0.00 |
| TRINITY_DN103729_c0_g1_i3 | | 0.00 | 2.88 | -4.27 | 0.00 |

| | | | | | |
|---|---|---|---|---|---|
| TRINITY_DN103729_c0_g1_i7 | | 0.00 | 4.23 | -5.49 | 0.00 |
| TRINITY_DN103729_c0_g1_i8 | | 0.15 | 3.13 | -4.64 | 0.00 |
| TRINITY_DN103729_c0_g1_i9 | | 0.23 | 3.19 | -3.71 | 0.00 |
| TRINITY_DN103740_c6_g1_i1 | | 6.09 | 19.96 | -1.72 | 0.00 |
| TRINITY_DN103758_c0_g1_i3 | SAP | 13.85 | 46.50 | -1.76 | 0.00 |
| TRINITY_DN103763_c1_g1_i6 | GHR | 8.03 | 4.94 | 0.67 | 0.00 |
| TRINITY_DN103763_c1_g1_i9 | GHR | 5.67 | 1.87 | 1.61 | 0.00 |
| TRINITY_DN103785_c6_g1_i3 | | 0.79 | 3.34 | -2.09 | 0.00 |
| TRINITY_DN103792_c1_g1_i18 | AGRG5 | 2.79 | 9.00 | -1.67 | 0.00 |
| TRINITY_DN103818_c3_g1_i1 | | 5.39 | 2.46 | 1.15 | 0.00 |
| TRINITY_DN103818_c4_g1_i2 | XCR1 | 3.19 | 9.15 | -1.53 | 0.00 |
| TRINITY_DN103819_c0_g2_i2 | GDIR2 | 11.78 | 31.50 | -1.42 | 0.00 |
| TRINITY_DN103822_c0_g1_i7 | SC24A | 0.05 | 0.90 | -4.33 | 0.00 |
| TRINITY_DN103823_c1_g2_i5 | PKHG7 | 0.54 | 2.33 | -2.12 | 0.00 |
| TRINITY_DN103843_c1_g2_i2 | | 8.74 | 1.94 | 2.21 | 0.00 |
| TRINITY_DN103849_c1_g2_i1 | GNAQ | 0.03 | 1.04 | -4.45 | 0.00 |
| TRINITY_DN103858_c2_g1_i5 | G6PD | 3.58 | 10.05 | -1.53 | 0.00 |
| TRINITY_DN103866_c3_g2_i2 | YTX2 | 0.39 | 2.12 | -2.47 | 0.00 |
| TRINITY_DN103885_c0_g2_i9 | | 1.90 | 0.21 | 3.17 | 0.00 |
| TRINITY_DN103893_c0_g1_i6 | | 0.14 | 6.79 | -5.30 | 0.00 |
| TRINITY_DN103894_c0_g1_i2 | AT2A3 | 7.46 | 17.17 | -1.24 | 0.00 |
| TRINITY_DN103898_c2_g1_i2 | FA49A | 7.23 | 15.31 | -1.12 | 0.00 |
| TRINITY_DN103899_c0_g3_i3 | SYNE1 | 0.09 | 1.06 | -3.53 | 0.00 |
| TRINITY_DN103904_c4_g1_i1 | DNJA2 | 2.53 | 0.00 | 7.32 | 0.00 |
| TRINITY_DN103904_c5_g1_i10 | | 1.49 | 18.69 | -3.65 | 0.00 |
| TRINITY_DN103905_c1_g4_i2 | ZC12B | 0.07 | 1.04 | -3.28 | 0.00 |
| TRINITY_DN103926_c2_g1_i1 | | 6.30 | 2.88 | 1.14 | 0.00 |
| TRINITY_DN103948_c3_g1_i3 | VWA7 | 0.45 | 10.55 | -4.48 | 0.00 |
| TRINITY_DN103948_c3_g1_i7 | VWA7 | 0.02 | 0.92 | -5.11 | 0.00 |
| TRINITY_DN103948_c3_g3_i1 | | 16.71 | 6.02 | 1.47 | 0.00 |
| TRINITY_DN103949_c4_g3_i1 | DCR1A | 1.24 | 3.63 | -1.54 | 0.00 |
| TRINITY_DN103959_c5_g2_i1 | BTLA | 0.02 | 0.64 | -3.76 | 0.00 |
| TRINITY_DN103990_c2_g1_i4 | GRM1C | 1.50 | 4.58 | -1.58 | 0.00 |
| TRINITY_DN103990_c2_g2_i7 | GRM1C | 0.19 | 1.48 | -3.03 | 0.00 |
| TRINITY_DN104006_c3_g1_i1 | | 5.52 | 0.32 | 3.79 | 0.00 |
| TRINITY_DN104006_c6_g1_i1 | | 0.42 | 4.55 | -3.46 | 0.00 |
| TRINITY_DN104009_c1_g2_i2 | TRI66 | 0.43 | 2.43 | -2.48 | 0.00 |
| TRINITY_DN104009_c1_g2_i3 | TRI66 | 0.51 | 1.92 | -1.90 | 0.00 |
| TRINITY_DN104015_c1_g1_i1 | | 31.09 | 13.33 | 1.20 | 0.00 |
| TRINITY_DN104043_c14_g1_i1 | | 3.00 | 0.28 | 3.03 | 0.00 |
| TRINITY_DN104062_c0_g1_i1 | | 0.79 | 10.72 | -3.80 | 0.00 |
| TRINITY_DN104062_c1_g1_i12 | | 0.04 | 1.07 | -3.70 | 0.00 |

| | | | | | |
|---|---|---|---|---|---|
| TRINITY_DN104062_c1_g1_i6 | | 1.61 | 5.25 | -1.73 | 0.00 |
| TRINITY_DN104062_c2_g1_i3 | | 6.81 | 2.73 | 1.37 | 0.00 |
| TRINITY_DN104064_c0_g1_i11 | ITB2 | 0.40 | 7.43 | -4.23 | 0.00 |
| TRINITY_DN104064_c0_g1_i7 | ITB2 | 3.59 | 8.28 | -1.21 | 0.00 |
| TRINITY_DN104072_c0_g2_i6 | CASPC | 0.00 | 1.02 | -4.93 | 0.00 |
| TRINITY_DN104079_c7_g2_i1 | | 5.47 | 14.15 | -1.39 | 0.00 |
| TRINITY_DN104079_c8_g3_i1 | | 55.45 | 25.90 | 1.14 | 0.00 |
| TRINITY_DN104086_c1_g6_i1 | | 1.39 | 31.98 | -4.38 | 0.00 |
| TRINITY_DN104092_c1_g1_i2 | SRRM1 | 0.78 | 0.14 | 2.67 | 0.00 |
| TRINITY_DN104095_c7_g2_i2 | NCF4 | 1.91 | 4.98 | -1.38 | 0.00 |
| TRINITY_DN104111_c0_g1_i1 | PLK3 | 0.57 | 1.83 | -1.69 | 0.00 |
| TRINITY_DN104111_c0_g1_i3 | PLK3 | 1.79 | 4.26 | -1.28 | 0.00 |
| TRINITY_DN104121_c0_g1_i9 | | 0.79 | 0.01 | 5.80 | 0.00 |
| TRINITY_DN104123_c2_g3_i3 | TBA3 | 1.22 | 27.04 | -4.47 | 0.00 |
| TRINITY_DN104131_c1_g3_i1 | | 1.59 | 4.50 | -1.52 | 0.00 |
| TRINITY_DN104136_c1_g1_i8 | DHX58 | 0.03 | 0.58 | -4.32 | 0.00 |
| TRINITY_DN104152_c0_g1_i6 | PPGB | 9.04 | 16.81 | -0.91 | 0.00 |
| TRINITY_DN104156_c6_g2_i2 | | 2.90 | 12.15 | -2.13 | 0.00 |
| TRINITY_DN104156_c6_g2_i3 | | 0.04 | 2.32 | -5.33 | 0.00 |
| TRINITY_DN104156_c6_g6_i1 | | 19.03 | 52.73 | -1.46 | 0.00 |
| TRINITY_DN104169_c10_g1_i1 | | 0.12 | 1.94 | -4.07 | 0.00 |
| TRINITY_DN104177_c2_g4_i1 | FGFP1 | 5.83 | 2.35 | 1.36 | 0.00 |
| TRINITY_DN104191_c7_g1_i2 | PARD3 | 0.90 | 0.07 | 3.52 | 0.00 |
| TRINITY_DN104195_c1_g8_i1 | UBP33 | 1.85 | 9.37 | -2.26 | 0.00 |
| TRINITY_DN104210_c0_g2_i1 | CSCL1 | 0.00 | 1.71 | -7.34 | 0.00 |
| TRINITY_DN104231_c8_g3_i1 | | 2.44 | 19.33 | -2.98 | 0.00 |
| TRINITY_DN104234_c3_g1_i2 | CHD2 | 6.01 | 0.00 | 5.18 | 0.00 |
| TRINITY_DN104236_c2_g1_i10 | MALD2 | 0.97 | 0.00 | 6.46 | 0.00 |
| TRINITY_DN104240_c1_g1_i10 | CCNG1 | 0.00 | 1.01 | -5.16 | 0.00 |
| TRINITY_DN104269_c4_g1_i2 | I10R1 | 0.20 | 1.50 | -2.92 | 0.00 |
| TRINITY_DN104269_c4_g1_i4 | I10R1 | 0.53 | 2.79 | -2.40 | 0.00 |
| TRINITY_DN104278_c2_g1_i1 | BGH3 | 8.90 | 4.58 | 0.94 | 0.00 |
| TRINITY_DN104300_c8_g3_i1 | | 1.82 | 4.08 | -1.18 | 0.00 |
| TRINITY_DN104320_c7_g3_i2 | | 1.59 | 4.63 | -1.57 | 0.00 |
| TRINITY_DN104327_c0_g1_i16 | HIPK3 | 0.03 | 0.78 | -5.34 | 0.00 |
| TRINITY_DN104341_c3_g1_i4 | LKHA4 | 1.52 | 3.83 | -1.35 | 0.00 |
| TRINITY_DN104342_c0_g1_i11 | SETVS | 17.62 | 46.52 | -1.37 | 0.00 |
| TRINITY_DN104342_c0_g1_i31 | CY24B | 0.03 | 0.71 | -3.86 | 0.00 |
| TRINITY_DN104351_c1_g2_i7 | K1C13 | 104.35 | 18.09 | 2.56 | 0.00 |
| TRINITY_DN104413_c3_g1_i1 | RN138 | 0.44 | 3.72 | -3.12 | 0.00 |
| TRINITY_DN104413_c3_g1_i2 | RN138 | 0.27 | 3.69 | -3.73 | 0.00 |
| TRINITY_DN104419_c1_g1_i9 | CO9A3 | 2.49 | 0.46 | 2.36 | 0.00 |

| | | | | | |
|---|---|---|---|---|---|
| TRINITY_DN104439_c4_g1_i1 | | 0.21 | 1.04 | -2.32 | 0.00 |
| TRINITY_DN104443_c1_g1_i3 | LY75 | 0.12 | 1.68 | -3.63 | 0.00 |
| TRINITY_DN104451_c1_g1_i9 | DYN2 | 0.00 | 0.60 | -4.79 | 0.00 |
| TRINITY_DN104460_c1_g3_i1 | K1C13 | 176.33 | 66.11 | 1.42 | 0.00 |
| TRINITY_DN104460_c1_g3_i2 | K1C13 | 63.33 | 12.83 | 2.36 | 0.00 |
| TRINITY_DN104460_c1_g3_i7 | K1C13 | 111.12 | 44.97 | 1.41 | 0.00 |
| TRINITY_DN104463_c0_g3_i1 | | 1.75 | 6.79 | -1.88 | 0.00 |
| TRINITY_DN104468_c12_g4_i1 | | 0.97 | 7.69 | -3.02 | 0.00 |
| TRINITY_DN104468_c13_g2_i1 | | 10.08 | 5.59 | 0.86 | 0.00 |
| TRINITY_DN104474_c2_g1_i5 | GBP1 | 4.58 | 17.39 | -1.95 | 0.00 |
| TRINITY_DN104474_c2_g2_i4 | GBP1 | 5.60 | 19.59 | -1.79 | 0.00 |
| TRINITY_DN104474_c2_g2_i7 | GBP1 | 0.56 | 8.72 | -3.90 | 0.00 |
| TRINITY_DN104474_c2_g2_i9 | GBP1 | 2.46 | 14.87 | -2.62 | 0.00 |
| TRINITY_DN104474_c2_g3_i12 | GBP1 | 1.36 | 9.95 | -2.86 | 0.00 |
| TRINITY_DN104474_c2_g3_i3 | GBP1 | 4.05 | 15.92 | -1.95 | 0.00 |
| TRINITY_DN104474_c2_g3_i9 | GBP1 | 2.04 | 5.80 | -1.51 | 0.00 |
| TRINITY_DN104475_c2_g1_i11 | HECA2 | 2.22 | 7.96 | -1.83 | 0.00 |
| TRINITY_DN104481_c6_g1_i5 | ZN366 | 0.91 | 1.93 | -1.11 | 0.00 |
| TRINITY_DN104487_c4_g1_i8 | B3AT | 0.16 | 4.54 | -4.78 | 0.00 |
| TRINITY_DN104517_c0_g1_i4 | CO3 | 1.88 | 0.04 | 5.81 | 0.00 |
| TRINITY_DN104519_c1_g8_i1 | | 0.29 | 3.66 | -3.79 | 0.00 |
| TRINITY_DN104546_c4_g1_i9 | ARF4 | 7.09 | 0.00 | 7.21 | 0.00 |
| TRINITY_DN104553_c7_g2_i3 | CATH | 52.84 | 124.80 | -1.23 | 0.00 |
| TRINITY_DN104557_c2_g1_i1 | FMNL1 | 1.67 | 4.57 | -1.47 | 0.00 |
| TRINITY_DN104562_c1_g3_i1 | SRC8 | 0.16 | 12.11 | -5.90 | 0.00 |
| TRINITY_DN104562_c1_g3_i5 | SRC8 | 1.83 | 0.25 | 2.82 | 0.00 |
| TRINITY_DN104562_c1_g3_i7 | SRC8 | 0.00 | 4.19 | -7.72 | 0.00 |
| TRINITY_DN104569_c3_g1_i2 | LAMB4 | 5.92 | 2.40 | 1.33 | 0.00 |
| TRINITY_DN104574_c3_g3_i1 | IL21R | 0.00 | 0.66 | -5.12 | 0.00 |
| TRINITY_DN104574_c3_g3_i2 | IL21R | 0.12 | 0.88 | -2.91 | 0.00 |
| TRINITY_DN104574_c3_g3_i3 | IL21R | 0.14 | 1.63 | -3.49 | 0.00 |
| TRINITY_DN104574_c3_g3_i4 | IL21R | 0.00 | 1.25 | -6.34 | 0.00 |
| TRINITY_DN104578_c1_g2_i2 | HMCN2 | 1.23 | 3.93 | -1.76 | 0.00 |
| TRINITY_DN104581_c5_g1_i10 | CD276 | 0.16 | 1.14 | -2.93 | 0.00 |
| TRINITY_DN104585_c1_g1_i13 | MBP | 6.13 | 2.80 | 1.11 | 0.00 |
| TRINITY_DN104587_c8_g1_i2 | CCR5 | 0.71 | 2.17 | -1.68 | 0.00 |
| TRINITY_DN104587_c8_g1_i4 | CCR5 | 0.17 | 3.87 | -4.52 | 0.00 |
| TRINITY_DN104588_c4_g1_i10 | | 0.11 | 5.57 | -5.07 | 0.00 |
| TRINITY_DN104588_c4_g1_i4 | AGRF3 | 0.33 | 3.05 | -3.15 | 0.00 |
| TRINITY_DN104588_c4_g2_i1 | | 0.12 | 1.45 | -3.03 | 0.00 |
| TRINITY_DN104601_c3_g1_i10 | | 14.90 | 34.44 | -1.23 | 0.00 |
| TRINITY_DN104604_c4_g1_i8 | | 0.00 | 0.96 | -4.83 | 0.00 |

| | | | | | |
|---|---|---|---|---|---|
| TRINITY_DN104604_c5_g1_i4 | | 1.29 | 8.23 | -2.63 | 0.00 |
| TRINITY_DN104633_c1_g1_i4 | | 0.23 | 15.71 | -5.41 | 0.00 |
| TRINITY_DN104633_c1_g9_i1 | | 0.00 | 12.19 | -4.56 | 0.00 |
| TRINITY_DN104634_c2_g2_i3 | | 16.37 | 4.09 | 2.05 | 0.00 |
| TRINITY_DN104641_c3_g5_i1 | | 3.04 | 10.99 | -1.90 | 0.00 |
| TRINITY_DN104644_c2_g1_i4 | ANXA4 | 16.27 | 8.02 | 0.99 | 0.00 |
| TRINITY_DN104660_c0_g2_i1 | HS90B | 368.01 | 644.00 | -0.81 | 0.00 |
| TRINITY_DN104678_c5_g6_i1 | | 0.27 | 11.46 | -4.65 | 0.00 |
| TRINITY_DN104685_c1_g1_i10 | K1468 | 0.19 | 1.23 | -2.68 | 0.00 |
| TRINITY_DN104690_c5_g2_i1 | | 2.36 | 7.59 | -1.67 | 0.00 |
| TRINITY_DN104691_c2_g2_i2 | PLCL2 | 0.59 | 2.42 | -2.00 | 0.00 |
| TRINITY_DN104691_c3_g4_i1 | PLCL2 | 0.57 | 1.63 | -1.57 | 0.00 |
| TRINITY_DN104698_c3_g2_i3 | GPC1 | 4.13 | 2.20 | 0.91 | 0.00 |
| TRINITY_DN104715_c6_g2_i1 | | 2.04 | 0.23 | 3.32 | 0.00 |
| TRINITY_DN104722_c13_g1_i2 | | 0.17 | 2.52 | -3.52 | 0.00 |
| TRINITY_DN104733_c2_g1_i1 | AMFR | 1.21 | 0.00 | 6.88 | 0.00 |
| TRINITY_DN104735_c0_g2_i1 | PTPRZ | 1.42 | 0.50 | 1.52 | 0.00 |
| TRINITY_DN104745_c3_g1_i1 | | 0.56 | 2.09 | -1.94 | 0.00 |
| TRINITY_DN104753_c6_g1_i1 | | 3.18 | 19.35 | -2.68 | 0.00 |
| TRINITY_DN104761_c1_g1_i3 | MUCM | 1.52 | 10.82 | -2.96 | 0.00 |
| TRINITY_DN104761_c1_g1_i5 | IGHM | 1.98 | 19.18 | -3.30 | 0.00 |
| TRINITY_DN104761_c1_g5_i2 | HV103 | 0.00 | 3.63 | -4.13 | 0.00 |
| TRINITY_DN104766_c7_g1_i1 | | 22.69 | 12.58 | 0.82 | 0.00 |
| TRINITY_DN104769_c6_g2_i1 | IFIT5 | 0.16 | 1.96 | -3.33 | 0.00 |
| TRINITY_DN104776_c1_g2_i1 | GBP1 | 3.48 | 14.96 | -2.12 | 0.00 |
| TRINITY_DN104776_c1_g3_i1 | GBP1 | 0.29 | 3.25 | -3.53 | 0.00 |
| TRINITY_DN104789_c1_g1_i21 | FGD4 | 0.89 | 0.00 | 6.81 | 0.00 |
| TRINITY_DN104791_c3_g1_i1 | USPL1 | 1.06 | 0.00 | 4.84 | 0.00 |
| TRINITY_DN104805_c0_g1_i5 | MCF2L | 0.13 | 1.04 | -2.95 | 0.00 |
| TRINITY_DN104805_c0_g1_i7 | MCF2L | 0.14 | 1.43 | -3.42 | 0.00 |
| TRINITY_DN104833_c1_g1_i10 | PRP39 | 0.00 | 2.71 | -6.48 | 0.00 |
| TRINITY_DN104833_c1_g1_i2 | PRP39 | 0.00 | 3.47 | -8.23 | 0.00 |
| TRINITY_DN104842_c5_g1_i4 | 3BP1 | 1.37 | 3.68 | -1.43 | 0.00 |
| TRINITY_DN104842_c5_g1_i9 | 3BP1 | 0.53 | 1.60 | -1.64 | 0.00 |
| TRINITY_DN104844_c3_g6_i2 | | 0.00 | 7.59 | -6.36 | 0.00 |
| TRINITY_DN104857_c0_g1_i11 | NFKB1 | 0.03 | 2.35 | -6.39 | 0.00 |
| TRINITY_DN104861_c1_g1_i2 | MYH9 | 0.32 | 4.29 | -3.62 | 0.00 |
| TRINITY_DN104861_c1_g1_i3 | MYH9 | 0.00 | 2.62 | -6.81 | 0.00 |
| TRINITY_DN104880_c5_g1_i13 | IP3KB | 0.19 | 1.36 | -2.90 | 0.00 |
| TRINITY_DN104880_c5_g3_i1 | | 0.26 | 9.14 | -4.67 | 0.00 |
| TRINITY_DN104886_c11_g2_i1 | | 0.63 | 1.75 | -1.49 | 0.00 |
| TRINITY_DN104915_c3_g1_i2 | SPY2 | 1.17 | 3.34 | -1.51 | 0.00 |

| | | | | | |
|---|---|---|---|---|---|
| TRINITY_DN104931_c2_g2_i2 | | 0.78 | 12.18 | -3.83 | 0.00 |
| TRINITY_DN104934_c1_g1_i5 | RUNX3 | 3.37 | 7.63 | -1.19 | 0.00 |
| TRINITY_DN104936_c2_g1_i7 | GRDN | 0.70 | 1.94 | -1.51 | 0.00 |
| TRINITY_DN104952_c3_g1_i6 | ELMO1 | 0.03 | 0.71 | -4.34 | 0.00 |
| TRINITY_DN104960_c2_g1_i1 | EM55 | 2.40 | 4.99 | -1.08 | 0.00 |
| TRINITY_DN104971_c3_g1_i1 | COL12 | 8.67 | 4.17 | 1.07 | 0.00 |
| TRINITY_DN104971_c3_g1_i4 | COL12 | 2.51 | 0.86 | 1.53 | 0.00 |
| TRINITY_DN104977_c6_g1_i2 | | 0.32 | 16.53 | -5.32 | 0.00 |
| TRINITY_DN104982_c2_g1_i3 | | 30.89 | 13.17 | 1.27 | 0.00 |
| TRINITY_DN104982_c2_g1_i4 | | 27.90 | 12.92 | 1.15 | 0.00 |
| TRINITY_DN104989_c1_g1_i12 | PI3R6 | 0.64 | 2.72 | -2.14 | 0.00 |
| TRINITY_DN104998_c0_g1_i21 | | 3.42 | 0.09 | 5.06 | 0.00 |
| TRINITY_DN105001_c8_g1_i2 | MIME | 13.34 | 5.60 | 1.25 | 0.00 |
| TRINITY_DN105042_c1_g3_i1 | | 12.65 | 7.23 | 0.80 | 0.00 |
| TRINITY_DN105051_c1_g1_i1 | IL6RA | 2.01 | 6.42 | -1.69 | 0.00 |
| TRINITY_DN105051_c1_g1_i9 | IL6RA | 2.43 | 5.17 | -1.10 | 0.00 |
| TRINITY_DN105062_c0_g3_i1 | | 9.54 | 32.79 | -1.74 | 0.00 |
| TRINITY_DN105063_c6_g1_i2 | | 0.76 | 16.20 | -4.42 | 0.00 |
| TRINITY_DN105070_c3_g1_i2 | | 0.61 | 11.78 | -4.09 | 0.00 |
| TRINITY_DN105070_c3_g1_i3 | | 0.20 | 13.80 | -5.40 | 0.00 |
| TRINITY_DN105073_c2_g1_i1 | LOX12 | 0.00 | 5.14 | -4.62 | 0.00 |
| TRINITY_DN105073_c4_g1_i3 | | 9.69 | 17.04 | -0.85 | 0.00 |
| TRINITY_DN105075_c3_g1_i2 | | 3.82 | 30.70 | -3.01 | 0.00 |
| TRINITY_DN105091_c2_g1_i1 | STAT4 | 2.66 | 9.40 | -1.82 | 0.00 |
| TRINITY_DN105107_c10_g1_i1 | | 2.37 | 6.99 | -1.58 | 0.00 |
| TRINITY_DN105107_c11_g1_i1 | BMP4 | 4.99 | 2.54 | 0.99 | 0.00 |
| TRINITY_DN105119_c0_g1_i10 | CEP95 | 0.00 | 0.65 | -5.76 | 0.00 |
| TRINITY_DN105123_c3_g1_i1 | CLC4M | 0.40 | 20.39 | -5.32 | 0.00 |
| TRINITY_DN105133_c1_g1_i9 | BIR | 2.01 | 6.99 | -1.78 | 0.00 |
| TRINITY_DN105133_c1_g3_i1 | | 6.32 | 12.68 | -0.98 | 0.00 |
| TRINITY_DN105136_c0_g1_i17 | VM2US | 0.00 | 1.43 | -5.21 | 0.00 |
| TRINITY_DN105138_c5_g1_i10 | | 1.39 | 4.67 | -1.76 | 0.00 |
| TRINITY_DN105138_c5_g1_i13 | LY9 | 0.06 | 1.64 | -4.47 | 0.00 |
| TRINITY_DN105138_c5_g1_i5 | CD2 | 4.06 | 14.51 | -1.91 | 0.00 |
| TRINITY_DN105151_c4_g7_i1 | | 1.52 | 3.68 | -1.31 | 0.00 |
| TRINITY_DN105163_c2_g2_i5 | | 5.08 | 35.64 | -2.78 | 0.00 |
| TRINITY_DN105166_c3_g2_i5 | STRP1 | 0.50 | 1.90 | -1.97 | 0.00 |
| TRINITY_DN105175_c2_g1_i2 | CATH | 2.44 | 0.41 | 2.74 | 0.00 |
| TRINITY_DN105177_c1_g1_i16 | ARC1B | 15.31 | 58.12 | -1.95 | 0.00 |
| TRINITY_DN105177_c1_g1_i7 | | 1.50 | 42.26 | -4.80 | 0.00 |
| TRINITY_DN105181_c5_g2_i1 | | 16.42 | 5.72 | 1.53 | 0.00 |
| TRINITY_DN105187_c0_g1_i4 | MPRGA | 0.13 | 1.11 | -3.03 | 0.00 |

| | | | | | |
|---|---|---|---|---|---|
| TRINITY_DN105189_c2_g1_i2 | | 1.11 | 0.00 | 5.16 | 0.00 |
| TRINITY_DN105189_c3_g5_i1 | | 0.54 | 1.98 | -1.88 | 0.00 |
| TRINITY_DN105190_c1_g3_i1 | CD2 | 0.90 | 7.60 | -3.08 | 0.00 |
| TRINITY_DN105191_c6_g1_i2 | | 2.62 | 1.17 | 1.14 | 0.00 |
| TRINITY_DN105197_c0_g1_i12 | SPD2A | 0.00 | 0.31 | -5.19 | 0.00 |
| TRINITY_DN105207_c0_g1_i5 | TAP2 | 0.80 | 4.90 | -2.67 | 0.00 |
| TRINITY_DN105207_c0_g1_i6 | TAP2 | 0.69 | 2.92 | -2.09 | 0.00 |
| TRINITY_DN105226_c6_g1_i7 | | 2.23 | 0.22 | 2.94 | 0.00 |
| TRINITY_DN105249_c3_g1_i6 | VAV | 2.87 | 8.40 | -1.61 | 0.00 |
| TRINITY_DN105259_c1_g2_i7 | SORT1 | 2.98 | 11.16 | -1.95 | 0.00 |
| TRINITY_DN105261_c0_g1_i1 | HELZ2 | 0.46 | 2.64 | -2.55 | 0.00 |
| TRINITY_DN105268_c4_g1_i8 | | 8.42 | 63.65 | -2.85 | 0.00 |
| TRINITY_DN105284_c3_g2_i7 | | 0.00 | 4.21 | -4.91 | 0.00 |
| TRINITY_DN105284_c3_g2_i9 | | 0.14 | 1.13 | -3.05 | 0.00 |
| TRINITY_DN105286_c0_g1_i3 | SYNE1 | 1.02 | 2.66 | -1.37 | 0.00 |
| TRINITY_DN105297_c13_g2_i2 | EMAL3 | 0.00 | 2.50 | -6.41 | 0.00 |
| TRINITY_DN105307_c3_g2_i4 | DHR11 | 6.72 | 3.30 | 0.98 | 0.00 |
| TRINITY_DN105326_c0_g1_i8 | PERE | 0.36 | 5.46 | -3.87 | 0.00 |
| TRINITY_DN105340_c6_g8_i1 | | 3.51 | 1.03 | 1.76 | 0.00 |
| TRINITY_DN105355_c0_g2_i11 | TCB | 5.03 | 16.57 | -1.76 | 0.00 |
| TRINITY_DN105355_c0_g2_i6 | TCB | 3.18 | 19.81 | -2.59 | 0.00 |
| TRINITY_DN105359_c0_g1_i1 | | 0.37 | 6.67 | -3.24 | 0.00 |
| TRINITY_DN105369_c1_g1_i1 | | 27.60 | 11.93 | 1.18 | 0.00 |
| TRINITY_DN105370_c2_g3_i1 | | 0.34 | 3.29 | -2.95 | 0.00 |
| TRINITY_DN105376_c0_g1_i3 | UN13B | 0.28 | 1.07 | -1.90 | 0.00 |
| TRINITY_DN105376_c0_g1_i6 | UN13B | 0.05 | 0.34 | -2.76 | 0.00 |
| TRINITY_DN105378_c5_g1_i2 | | 0.62 | 4.09 | -2.73 | 0.00 |
| TRINITY_DN105378_c5_g2_i11 | AFF3 | 0.08 | 0.64 | -2.91 | 0.00 |
| TRINITY_DN105383_c3_g1_i6 | | 6.72 | 3.74 | 0.83 | 0.00 |
| TRINITY_DN105399_c10_g2_i9 | | 0.00 | 2.25 | -5.70 | 0.00 |
| TRINITY_DN105406_c2_g5_i3 | MDC1 | 0.87 | 0.00 | 5.95 | 0.00 |
| TRINITY_DN105406_c2_g5_i6 | MDC1 | 0.00 | 0.90 | -5.70 | 0.00 |
| TRINITY_DN105411_c2_g1_i4 | PDPK1 | 0.58 | 1.60 | -1.48 | 0.00 |
| TRINITY_DN105412_c5_g1_i11 | KV315 | 1.18 | 10.62 | -3.21 | 0.00 |
| TRINITY_DN105414_c2_g1_i3 | | 1.63 | 23.35 | -3.81 | 0.00 |
| TRINITY_DN105430_c2_g1_i12 | PA24F | 1.43 | 0.10 | 3.90 | 0.00 |
| TRINITY_DN105440_c2_g1_i15 | RC3H2 | 0.17 | 1.25 | -2.95 | 0.00 |
| TRINITY_DN105451_c6_g2_i3 | | 0.38 | 2.20 | -2.48 | 0.00 |
| TRINITY_DN105451_c6_g5_i1 | MFS6L | 0.28 | 0.92 | -1.62 | 0.00 |
| TRINITY_DN105467_c0_g5_i2 | | 0.00 | 5.64 | -7.57 | 0.00 |
| TRINITY_DN105468_c1_g2_i7 | CNNM3 | 0.59 | 0.00 | 5.78 | 0.00 |
| TRINITY_DN105474_c1_g3_i2 | SAM9L | 0.25 | 4.29 | -4.04 | 0.00 |

| | | | | | |
|---|---|---|---|---|---|
| TRINITY_DN105474_c1_g3_i3 | SAM9L | 0.03 | 0.86 | -4.90 | 0.00 |
| TRINITY_DN105474_c1_g3_i5 | SAM9L | 0.05 | 1.53 | -4.66 | 0.00 |
| TRINITY_DN105474_c1_g3_i9 | SAM9L | 0.00 | 0.50 | -4.17 | 0.00 |
| TRINITY_DN105474_c1_g5_i1 | SAM9L | 0.73 | 9.77 | -3.65 | 0.00 |
| TRINITY_DN105482_c11_g3_i1 | | 3.42 | 8.36 | -1.31 | 0.00 |
| TRINITY_DN105483_c0_g1_i8 | SYNE3 | 0.10 | 1.30 | -3.83 | 0.00 |
| TRINITY_DN105495_c5_g2_i1 | | 3.14 | 35.69 | -3.38 | 0.00 |
| TRINITY_DN105503_c2_g1_i8 | | 1.47 | 7.23 | -2.22 | 0.00 |
| TRINITY_DN105510_c3_g1_i1 | | 7.01 | 4.08 | 0.77 | 0.00 |
| TRINITY_DN105520_c0_g1_i2 | | 14.58 | 2.36 | 2.67 | 0.00 |
| TRINITY_DN105520_c1_g1_i5 | NECT3 | 1.02 | 4.07 | -2.04 | 0.00 |
| TRINITY_DN105522_c4_g1_i1 | CD4 | 1.31 | 6.29 | -2.28 | 0.00 |
| TRINITY_DN105522_c4_g1_i11 | CD4 | 1.37 | 6.31 | -2.17 | 0.00 |
| TRINITY_DN105533_c6_g2_i1 | FYNB | 3.68 | 10.62 | -1.55 | 0.00 |
| TRINITY_DN105540_c2_g1_i5 | AEBP1 | 12.85 | 6.11 | 1.12 | 0.00 |
| TRINITY_DN105552_c8_g1_i1 | | 0.42 | 12.68 | -4.79 | 0.00 |
| TRINITY_DN105565_c1_g2_i1 | | 10.46 | 17.78 | -0.76 | 0.00 |
| TRINITY_DN105568_c12_g2_i1 | | 1.61 | 3.46 | -1.09 | 0.00 |
| TRINITY_DN105575_c2_g1_i4 | CGNL1 | 0.00 | 1.25 | -6.18 | 0.00 |
| TRINITY_DN105577_c2_g1_i7 | DRAM1 | 0.50 | 4.10 | -3.02 | 0.00 |
| TRINITY_DN105580_c0_g1_i7 | SHIP1 | 0.08 | 4.04 | -5.66 | 0.00 |
| TRINITY_DN105585_c0_g2_i6 | CBL | 0.11 | 1.65 | -3.32 | 0.00 |
| TRINITY_DN105594_c4_g1_i2 | MMP2 | 135.10 | 72.28 | 0.91 | 0.00 |
| TRINITY_DN105599_c2_g1_i1 | PLSL | 10.01 | 22.86 | -1.24 | 0.00 |
| TRINITY_DN105599_c2_g1_i7 | PLSL | 11.01 | 24.88 | -1.22 | 0.00 |
| TRINITY_DN105599_c2_g1_i8 | PLSL | 18.37 | 41.74 | -1.20 | 0.00 |
| TRINITY_DN105607_c5_g3_i1 | | 0.81 | 3.52 | -2.12 | 0.00 |
| TRINITY_DN105637_c5_g2_i5 | | 3.40 | 0.24 | 2.97 | 0.00 |
| TRINITY_DN105655_c3_g4_i3 | | 0.00 | 26.11 | -6.58 | 0.00 |
| TRINITY_DN105655_c3_g4_i4 | | 0.00 | 12.33 | -7.86 | 0.00 |
| TRINITY_DN105655_c3_g4_i5 | | 0.00 | 30.48 | -5.41 | 0.00 |
| TRINITY_DN105659_c2_g2_i1 | | 0.00 | 1.57 | -5.26 | 0.00 |
| TRINITY_DN105668_c0_g1_i5 | MSLNL | 4.88 | 0.53 | 3.12 | 0.00 |
| TRINITY_DN105668_c0_g2_i2 | | 8.95 | 0.51 | 4.06 | 0.00 |
| TRINITY_DN105690_c6_g1_i11 | RHG15 | 0.03 | 1.80 | -5.48 | 0.00 |
| TRINITY_DN105690_c6_g1_i12 | RHG15 | 1.87 | 4.83 | -1.40 | 0.00 |
| TRINITY_DN105690_c6_g1_i2 | RHG15 | 1.18 | 3.14 | -1.42 | 0.00 |
| TRINITY_DN105695_c2_g3_i1 | HDAC2 | 0.55 | 5.85 | -3.42 | 0.00 |
| TRINITY_DN105701_c4_g1_i1 | ZC3H1 | 0.27 | 1.85 | -2.88 | 0.00 |
| TRINITY_DN105701_c4_g1_i16 | ZC3H1 | 0.04 | 0.37 | -3.15 | 0.00 |
| TRINITY_DN105705_c0_g3_i1 | LS14A | 4.37 | 2.87 | 0.61 | 0.00 |
| TRINITY_DN105705_c0_g3_i4 | LS14A | 0.00 | 0.64 | -5.45 | 0.00 |

| | | | | | |
|---|---|---|---|---|---|
| TRINITY_DN105741_c1_g1_i6 | CALD1 | 30.37 | 19.24 | 0.62 | 0.00 |
| TRINITY_DN105741_c1_g2_i1 | TNNT2 | 15.01 | 4.45 | 1.83 | 0.00 |
| TRINITY_DN105747_c1_g1_i1 | ITAX | 1.89 | 5.87 | -1.65 | 0.00 |
| TRINITY_DN105779_c0_g1_i2 | GVIN1 | 0.55 | 0.39 | -3.42 | 0.00 |
| TRINITY_DN105779_c0_g1_i4 | GVIN1 | 2.17 | 0.00 | 7.26 | 0.00 |
| TRINITY_DN105779_c0_g1_i7 | GVIN1 | 0.22 | 6.21 | -4.99 | 0.00 |
| TRINITY_DN105779_c1_g1_i11 | HPS3 | 0.42 | 1.32 | -1.64 | 0.00 |
| TRINITY_DN105790_c0_g1_i1 | A4 | 8.42 | 4.09 | 1.11 | 0.00 |
| TRINITY_DN105791_c2_g1_i2 | CD248 | 4.66 | 1.23 | 1.97 | 0.00 |
| TRINITY_DN105798_c2_g1_i4 | VP9D1 | 0.12 | 2.71 | -3.98 | 0.00 |
| TRINITY_DN105812_c0_g1_i7 | FADS2 | 0.77 | 2.16 | -1.50 | 0.00 |
| TRINITY_DN105815_c0_g3_i1 | HOME2 | 11.77 | 18.93 | -0.70 | 0.00 |
| TRINITY_DN105836_c7_g2_i2 | GBP1 | 8.48 | 37.31 | -2.10 | 0.00 |
| TRINITY_DN105837_c6_g1_i3 | IGK | 0.00 | 0.73 | -3.70 | 0.00 |
| TRINITY_DN105841_c2_g1_i5 | FGR1A | 0.01 | 1.08 | -4.42 | 0.00 |
| TRINITY_DN105843_c2_g3_i1 | | 2.49 | 0.16 | 3.48 | 0.00 |
| TRINITY_DN105846_c2_g1_i4 | CALD1 | 1.30 | 7.98 | -2.71 | 0.00 |
| TRINITY_DN105846_c2_g2_i1 | | 10.30 | 31.10 | -1.59 | 0.00 |
| TRINITY_DN105851_c3_g1_i7 | AT11B | 0.00 | 0.61 | -5.91 | 0.00 |
| TRINITY_DN105853_c1_g2_i1 | | 8.67 | 2.11 | 2.16 | 0.00 |
| TRINITY_DN105858_c1_g1_i1 | | 0.17 | 10.20 | -4.16 | 0.00 |
| TRINITY_DN105858_c4_g1_i1 | | 1.80 | 19.19 | -3.40 | 0.00 |
| TRINITY_DN105859_c2_g1_i3 | TSP4B | 14.56 | 7.78 | 0.89 | 0.00 |
| TRINITY_DN105874_c0_g1_i2 | RIMB2 | 0.00 | 0.74 | -4.19 | 0.00 |
| TRINITY_DN105893_c0_g1_i1 | PK3CG | 2.02 | 7.67 | -1.94 | 0.00 |
| TRINITY_DN105893_c0_g1_i5 | PK3CG | 0.40 | 1.60 | -2.02 | 0.00 |
| TRINITY_DN105896_c0_g1_i6 | ARIP4 | 0.00 | 0.56 | -3.92 | 0.00 |
| TRINITY_DN105900_c7_g1_i11 | GT2D2 | 1.34 | 0.01 | 6.30 | 0.00 |
| TRINITY_DN105906_c2_g1_i1 | TNR9 | 0.43 | 1.57 | -1.86 | 0.00 |
| TRINITY_DN105907_c5_g2_i2 | TAOK3 | 1.84 | 4.09 | -1.14 | 0.00 |
| TRINITY_DN105910_c0_g2_i8 | LTBP3 | 1.53 | 0.44 | 1.86 | 0.00 |
| TRINITY_DN105918_c2_g1_i1 | UN13D | 0.33 | 2.05 | -2.60 | 0.00 |
| TRINITY_DN105918_c2_g1_i12 | UN13D | 0.39 | 2.26 | -2.46 | 0.00 |
| TRINITY_DN105922_c1_g2_i2 | | 13.36 | 1.01 | 3.86 | 0.00 |
| TRINITY_DN105941_c1_g4_i1 | | 9.07 | 2.58 | 1.84 | 0.00 |
| TRINITY_DN105945_c0_g1_i3 | LOXL1 | 4.90 | 1.99 | 1.27 | 0.00 |
| TRINITY_DN105952_c1_g1_i12 | STRN | 0.64 | 0.00 | 5.08 | 0.00 |
| TRINITY_DN105977_c6_g1_i2 | PTN6 | 2.04 | 8.24 | -2.01 | 0.00 |
| TRINITY_DN105977_c6_g1_i6 | PTN6 | 7.29 | 13.01 | -0.85 | 0.00 |
| TRINITY_DN105990_c0_g1_i9 | CEAM1 | 0.00 | 0.43 | -4.99 | 0.00 |
| TRINITY_DN105997_c1_g2_i2 | KCNH2 | 0.00 | 0.60 | -6.21 | 0.00 |
| TRINITY_DN106002_c6_g2_i1 | FLNA | 4.72 | 0.33 | 3.54 | 0.00 |

| | | | | | |
|---|---|---|---|---|---|
| TRINITY_DN106007_c1_g1_i1 | PREX1 | 0.15 | 1.78 | -3.19 | 0.00 |
| TRINITY_DN106007_c1_g1_i4 | PREX1 | 0.90 | 2.02 | -1.18 | 0.00 |
| TRINITY_DN106008_c3_g1_i1 | GIMA4 | 3.56 | 12.90 | -1.90 | 0.00 |
| TRINITY_DN106008_c3_g1_i4 | GIMA7 | 10.24 | 75.06 | -2.86 | 0.00 |
| TRINITY_DN106014_c3_g2_i10 | PKHA6 | 0.07 | 1.47 | -4.36 | 0.00 |
| TRINITY_DN106017_c2_g1_i3 | FAK2 | 1.78 | 7.77 | -2.15 | 0.00 |
| TRINITY_DN106020_c0_g1_i10 | IDE | 3.23 | 2.18 | 0.55 | 0.00 |
| TRINITY_DN106020_c0_g1_i6 | IDE | 0.00 | 0.66 | -5.42 | 0.00 |
| TRINITY_DN106034_c2_g2_i1 | | 7.72 | 19.52 | -1.36 | 0.00 |
| TRINITY_DN106034_c3_g1_i17 | FCL | 8.58 | 19.01 | -1.18 | 0.00 |
| TRINITY_DN106036_c11_g1_i1 | | 0.32 | 9.12 | -3.98 | 0.00 |
| TRINITY_DN106037_c0_g1_i12 | DZIP1 | 0.32 | 0.01 | 4.93 | 0.00 |
| TRINITY_DN106037_c0_g1_i9 | DZIP1 | 0.58 | 0.03 | 4.59 | 0.00 |
| TRINITY_DN106038_c7_g2_i1 | | 1.93 | 4.93 | -1.36 | 0.00 |
| TRINITY_DN106043_c3_g1_i1 | BCAP | 0.13 | 1.15 | -3.17 | 0.00 |
| TRINITY_DN106043_c3_g1_i7 | BCAP | 0.39 | 1.52 | -1.93 | 0.00 |
| TRINITY_DN106067_c0_g1_i1 | GALT8 | 0.10 | 9.11 | -5.96 | 0.00 |
| TRINITY_DN106067_c0_g1_i10 | GLT18 | 3.12 | 6.33 | -1.05 | 0.00 |
| TRINITY_DN106067_c0_g1_i2 | GLT18 | 0.03 | 4.51 | -5.90 | 0.00 |
| TRINITY_DN106067_c0_g1_i8 | GALT8 | 0.25 | 9.89 | -5.20 | 0.00 |
| TRINITY_DN106067_c0_g2_i3 | GALT8 | 0.16 | 8.81 | -5.65 | 0.00 |
| TRINITY_DN106067_c0_g2_i6 | GALT8 | 13.65 | 32.02 | -1.26 | 0.00 |
| TRINITY_DN106067_c0_g4_i1 | | 0.88 | 15.56 | -4.09 | 0.00 |
| TRINITY_DN106081_c1_g1_i1 | NCS1 | 1.12 | 0.46 | 1.24 | 0.00 |
| TRINITY_DN106087_c4_g1_i3 | SDHL | 11.27 | 39.67 | -1.86 | 0.00 |
| TRINITY_DN106109_c2_g1_i11 | | 1.08 | 9.82 | -3.15 | 0.00 |
| TRINITY_DN106109_c2_g1_i14 | | 2.82 | 15.32 | -2.45 | 0.00 |
| TRINITY_DN106109_c2_g1_i16 | | 1.63 | 16.74 | -3.52 | 0.00 |
| TRINITY_DN106137_c11_g3_i1 | | 0.68 | 2.09 | -1.65 | 0.00 |
| TRINITY_DN106139_c4_g1_i3 | | 9.13 | 5.25 | 0.78 | 0.00 |
| TRINITY_DN106170_c3_g2_i3 | | 2.12 | 6.87 | -1.73 | 0.00 |
| TRINITY_DN106170_c5_g1_i13 | SIA8F | 0.04 | 0.74 | -4.47 | 0.00 |
| TRINITY_DN106172_c10_g1_i3 | | 0.03 | 1.19 | -5.18 | 0.00 |
| TRINITY_DN106173_c1_g2_i8 | | 18.05 | 33.38 | -0.88 | 0.00 |
| TRINITY_DN106174_c0_g3_i1 | SMBT2 | 9.96 | 42.63 | -2.07 | 0.00 |
| TRINITY_DN106182_c1_g2_i1 | MEFV | 1.04 | 12.24 | -3.36 | 0.00 |
| TRINITY_DN106190_c2_g1_i1 | | 96.66 | 25.95 | 2.00 | 0.00 |
| TRINITY_DN106205_c9_g3_i1 | CSK | 2.47 | 5.57 | -1.16 | 0.00 |
| TRINITY_DN106209_c2_g1_i3 | KACB | 0.89 | 3.50 | -1.87 | 0.00 |
| TRINITY_DN106209_c3_g2_i3 | | 0.00 | 5.38 | -3.92 | 0.00 |
| TRINITY_DN106222_c4_g4_i1 | CNPY3 | 4.63 | 11.30 | -1.28 | 0.00 |
| TRINITY_DN106230_c1_g1_i15 | GCC2 | 0.35 | 7.00 | -4.06 | 0.00 |

| | | | | | |
|---|---|---|---|---|---|
| TRINITY_DN106239_c1_g1_i8 | HVM15 | 0.00 | 2.66 | -4.17 | 0.00 |
| TRINITY_DN106249_c5_g1_i1 | | 0.63 | 30.52 | -5.43 | 0.00 |
| TRINITY_DN106250_c0_g1_i2 | B915 | 3.16 | 7.39 | -1.23 | 0.00 |
| TRINITY_DN106250_c0_g8_i1 | | 3.44 | 7.45 | -1.11 | 0.00 |
| TRINITY_DN106257_c0_g3_i1 | SMC1A | 1.73 | 12.66 | -2.81 | 0.00 |
| TRINITY_DN106258_c0_g1_i1 | | 0.22 | 2.99 | -3.97 | 0.00 |
| TRINITY_DN106273_c0_g1_i12 | MPSF | 2.14 | 0.02 | 6.61 | 0.00 |
| TRINITY_DN106273_c0_g1_i6 | MPSF | 0.00 | 4.37 | -24.06 | 0.00 |
| TRINITY_DN106277_c11_g2_i1 | | 0.14 | 1.18 | -3.11 | 0.00 |
| TRINITY_DN106315_c4_g2_i5 | | 1.58 | 13.82 | -3.03 | 0.00 |
| TRINITY_DN106315_c4_g4_i2 | MIA | 8.10 | 2.86 | 1.71 | 0.00 |
| TRINITY_DN106317_c8_g4_i4 | | 0.00 | 2.17 | -4.34 | 0.00 |
| TRINITY_DN106318_c1_g2_i1 | ICAM1 | 1.37 | 7.71 | -2.58 | 0.00 |
| TRINITY_DN106318_c1_g2_i2 | ICAM1 | 3.14 | 8.43 | -1.46 | 0.00 |
| TRINITY_DN106329_c1_g1_i7 | ETS1A | 3.84 | 7.66 | -1.05 | 0.00 |
| TRINITY_DN106331_c8_g1_i1 | | 0.33 | 3.02 | -3.53 | 0.00 |
| TRINITY_DN106334_c1_g1_i7 | URGCP | 0.06 | 7.29 | -6.52 | 0.00 |
| TRINITY_DN106334_c1_g2_i1 | GVIN1 | 0.19 | 9.15 | -5.24 | 0.00 |
| TRINITY_DN106353_c3_g1_i5 | | 4.34 | 24.01 | -2.51 | 0.00 |
| TRINITY_DN106367_c2_g1_i2 | ARF2 | 0.00 | 4.51 | -7.48 | 0.00 |
| TRINITY_DN106391_c3_g1_i1 | INSI1 | 3.42 | 6.45 | -0.94 | 0.00 |
| TRINITY_DN106392_c0_g1_i5 | SHOT1 | 1.86 | 4.79 | -1.38 | 0.00 |
| TRINITY_DN106398_c6_g1_i4 | | 12.95 | 60.51 | -2.28 | 0.00 |
| TRINITY_DN106409_c4_g2_i2 | EMSA1 | 1.15 | 0.03 | 4.89 | 0.00 |
| TRINITY_DN106410_c1_g2_i12 | M4K1 | 0.02 | 1.33 | -5.43 | 0.00 |
| TRINITY_DN106410_c1_g2_i15 | M4K1 | 0.03 | 1.38 | -4.56 | 0.00 |
| TRINITY_DN106415_c0_g2_i1 | IF2B3 | 1.25 | 0.01 | 6.91 | 0.00 |
| TRINITY_DN106424_c6_g9_i1 | | 0.95 | 10.11 | -3.36 | 0.00 |
| TRINITY_DN106428_c15_g1_i1 | | 8.37 | 19.32 | -1.20 | 0.00 |
| TRINITY_DN106438_c10_g2_i2 | | 3.03 | 0.56 | 2.56 | 0.00 |
| TRINITY_DN106439_c0_g1_i1 | | 5.36 | 18.06 | -1.77 | 0.00 |
| TRINITY_DN106439_c0_g3_i1 | | 0.95 | 3.14 | -1.67 | 0.00 |
| TRINITY_DN106439_c1_g1_i3 | RAB44 | 1.03 | 3.88 | -1.93 | 0.00 |
| TRINITY_DN106439_c1_g3_i1 | | 1.40 | 6.09 | -2.12 | 0.00 |
| TRINITY_DN106439_c1_g4_i1 | | 0.90 | 17.45 | -4.06 | 0.00 |
| TRINITY_DN106444_c1_g1_i6 | ARAP1 | 1.05 | 3.06 | -1.56 | 0.00 |
| TRINITY_DN106445_c3_g1_i1 | PAR14 | 0.01 | 0.57 | -5.02 | 0.00 |
| TRINITY_DN106447_c3_g2_i2 | FMOD | 2.78 | 0.06 | 5.21 | 0.00 |
| TRINITY_DN106450_c2_g1_i3 | DOCK2 | 0.01 | 0.49 | -5.05 | 0.00 |
| TRINITY_DN106450_c2_g1_i8 | DOCK2 | 0.04 | 1.38 | -5.92 | 0.00 |
| TRINITY_DN106461_c1_g1_i6 | KC1DB | 0.05 | 2.10 | -5.35 | 0.00 |
| TRINITY_DN106469_c0_g1_i1 | | 0.24 | 3.52 | -3.54 | 0.00 |

| | | | | | |
|---|---|---|---|---|---|
| TRINITY_DN106484_c1_g1_i17 | ATG9A | 0.01 | 0.49 | -5.17 | 0.00 |
| TRINITY_DN106500_c2_g1_i1 | HCLS1 | 6.75 | 22.33 | -1.81 | 0.00 |
| TRINITY_DN106503_c0_g1_i6 | NCOA3 | 1.59 | 7.33 | -2.24 | 0.00 |
| TRINITY_DN106533_c3_g1_i1 | | 0.44 | 3.47 | -3.04 | 0.00 |
| TRINITY_DN106536_c1_g2_i5 | TMC7 | 0.00 | 0.87 | -6.10 | 0.00 |
| TRINITY_DN106557_c7_g2_i2 | | 3.89 | 14.98 | -1.84 | 0.00 |
| TRINITY_DN106560_c2_g1_i1 | | 0.94 | 5.53 | -2.55 | 0.00 |
| TRINITY_DN106575_c3_g3_i3 | ICOSL | 0.61 | 2.38 | -1.92 | 0.00 |
| TRINITY_DN106580_c2_g1_i16 | CARL1 | 1.62 | 0.05 | 4.88 | 0.00 |
| TRINITY_DN106601_c3_g1_i7 | GOGA4 | 0.02 | 0.67 | -4.85 | 0.00 |
| TRINITY_DN106603_c1_g3_i8 | NECT3 | 0.70 | 4.21 | -2.74 | 0.00 |
| TRINITY_DN106612_c2_g1_i10 | SI1L2 | 0.34 | 1.16 | -1.78 | 0.00 |
| TRINITY_DN106612_c2_g1_i8 | SI1L1 | 0.44 | 1.64 | -1.89 | 0.00 |
| TRINITY_DN106614_c0_g1_i1 | EFHD2 | 27.76 | 40.61 | -0.58 | 0.00 |
| TRINITY_DN106617_c1_g2_i8 | CAM1B | 0.02 | 0.41 | -3.84 | 0.00 |
| TRINITY_DN106626_c6_g1_i17 | GPC6A | 0.21 | 0.86 | -2.16 | 0.00 |
| TRINITY_DN106627_c2_g2_i5 | | 5.66 | 0.28 | 4.29 | 0.00 |
| TRINITY_DN106632_c3_g1_i13 | WNK1 | 0.00 | 0.85 | -5.93 | 0.00 |
| TRINITY_DN106635_c17_g2_i3 | TCB1 | 11.14 | 0.86 | 3.83 | 0.00 |
| TRINITY_DN106647_c3_g1_i2 | IFI44 | 0.76 | 5.79 | -2.99 | 0.00 |
| TRINITY_DN106647_c3_g1_i4 | IFI44 | 0.28 | 5.45 | -4.33 | 0.00 |
| TRINITY_DN106647_c3_g1_i5 | IFI44 | 6.03 | 67.09 | -3.41 | 0.00 |
| TRINITY_DN106651_c2_g2_i4 | COHA1 | 11.40 | 2.82 | 1.07 | 0.00 |
| TRINITY_DN106653_c1_g1_i9 | HARB1 | 0.05 | 1.40 | -5.40 | 0.00 |
| TRINITY_DN106665_c2_g1_i4 | TNIP1 | 9.03 | 19.15 | -1.10 | 0.00 |
| TRINITY_DN106665_c2_g6_i1 | | 21.64 | 31.95 | -0.59 | 0.00 |
| TRINITY_DN106672_c2_g1_i1 | E41L2 | 4.16 | 9.77 | -1.19 | 0.00 |
| TRINITY_DN106689_c2_g1_i1 | | 13.84 | 23.98 | -0.79 | 0.00 |
| TRINITY_DN106701_c4_g1_i3 | K1C13 | 100.67 | 33.39 | 1.72 | 0.00 |
| TRINITY_DN106701_c4_g1_i4 | K1C13 | 123.06 | 49.22 | 1.47 | 0.00 |
| TRINITY_DN106701_c4_g2_i1 | K1C13 | 136.32 | 50.83 | 1.53 | 0.00 |
| TRINITY_DN106701_c4_g4_i4 | K1C13 | 102.18 | 34.10 | 1.64 | 0.00 |
| TRINITY_DN106701_c4_g4_i6 | K1C13 | 488.52 | 148.04 | 1.92 | 0.00 |
| TRINITY_DN106709_c1_g3_i1 | | 8.11 | 22.56 | -1.46 | 0.00 |
| TRINITY_DN106722_c1_g1_i7 | GLCNE | 0.00 | 1.35 | -5.64 | 0.00 |
| TRINITY_DN106740_c2_g7_i1 | | 30.31 | 12.34 | 1.34 | 0.00 |
| TRINITY_DN106759_c4_g1_i4 | MRP1 | 0.67 | 4.98 | -2.78 | 0.00 |
| TRINITY_DN106759_c4_g3_i1 | MRP3 | 4.72 | 14.83 | -1.65 | 0.00 |
| TRINITY_DN106759_c4_g5_i1 | MRP2 | 2.77 | 22.20 | -2.85 | 0.00 |
| TRINITY_DN106766_c4_g4_i1 | | 3.44 | 1.23 | 1.55 | 0.00 |
| TRINITY_DN106774_c2_g2_i3 | LAMB1 | 6.17 | 3.13 | 1.02 | 0.00 |
| TRINITY_DN106783_c3_g1_i4 | HOOK2 | 0.00 | 1.29 | -6.45 | 0.00 |

| | | | | | |
|---|---|---|---|---|---|
| TRINITY_DN106791_c5_g1_i7 | SP14L | 0.80 | 4.21 | -2.37 | 0.00 |
| TRINITY_DN106797_c14_g1_i1 | | 0.00 | 11.07 | -5.05 | 0.00 |
| TRINITY_DN106801_c1_g1_i2 | TF29 | 0.00 | 0.79 | -6.33 | 0.00 |
| TRINITY_DN106818_c9_g1_i1 | | 75.38 | 127.53 | -0.73 | 0.00 |
| TRINITY_DN106841_c5_g1_i6 | | 239.35 | 120.27 | 1.05 | 0.00 |
| TRINITY_DN106841_c5_g1_i8 | CO1A1 | 202.30 | 98.88 | 1.06 | 0.00 |
| TRINITY_DN106855_c2_g2_i1 | DGKZ | 0.19 | 1.13 | -2.72 | 0.00 |
| TRINITY_DN106890_c4_g1_i6 | MICA2 | 0.00 | 0.72 | -6.09 | 0.00 |
| TRINITY_DN106899_c1_g1_i1 | CO1A1 | 268.57 | 133.93 | 1.05 | 0.00 |
| TRINITY_DN106904_c1_g1_i4 | | 10.58 | 0.79 | 3.78 | 0.00 |
| TRINITY_DN106915_c5_g2_i5 | EFC1 | 15.94 | 1.41 | 3.54 | 0.00 |
| TRINITY_DN106926_c2_g2_i6 | TRPM4 | 0.10 | 2.12 | -4.26 | 0.00 |
| TRINITY_DN106936_c9_g3_i1 | | 0.60 | 8.87 | -3.75 | 0.00 |
| TRINITY_DN106951_c1_g2_i2 | BLNK | 0.14 | 0.76 | -2.42 | 0.00 |
| TRINITY_DN106954_c3_g1_i1 | MPRI | 0.01 | 0.30 | -3.85 | 0.00 |
| TRINITY_DN106971_c7_g1_i1 | | 1.82 | 51.97 | -4.86 | 0.00 |
| TRINITY_DN106971_c7_g1_i2 | | 25.11 | 232.97 | -3.26 | 0.00 |
| TRINITY_DN106971_c7_g1_i3 | | 3.42 | 63.33 | -4.25 | 0.00 |
| TRINITY_DN106984_c6_g1_i6 | FCGBP | 4.56 | 1.62 | 1.49 | 0.00 |
| TRINITY_DN106984_c6_g1_i8 | FCGBP | 27.26 | 6.00 | 2.13 | 0.00 |
| TRINITY_DN106992_c3_g1_i2 | SORL | 0.00 | 0.79 | -6.82 | 0.00 |
| TRINITY_DN106993_c0_g1_i6 | LIPB2 | 0.02 | 0.48 | -4.80 | 0.00 |
| TRINITY_DN106994_c1_g2_i1 | | 34.63 | 15.67 | 1.16 | 0.00 |
| TRINITY_DN106994_c2_g2_i6 | CO5A1 | 13.92 | 3.81 | 1.92 | 0.00 |
| TRINITY_DN106997_c1_g2_i1 | THB | 0.00 | 2.12 | -5.29 | 0.00 |
| TRINITY_DN107000_c2_g3_i1 | | 17.17 | 2.79 | 2.71 | 0.00 |
| TRINITY_DN107048_c7_g3_i1 | | 0.00 | 8.15 | -6.38 | 0.00 |
| TRINITY_DN107054_c12_g1_i4 | | 38.83 | 1.63 | 4.69 | 0.00 |
| TRINITY_DN107054_c12_g3_i2 | | 5.11 | 13.83 | -1.45 | 0.00 |
| TRINITY_DN107079_c5_g2_i1 | | 30.02 | 63.62 | -1.10 | 0.00 |
| TRINITY_DN107079_c5_g6_i1 | | 29.62 | 76.17 | -1.36 | 0.00 |
| TRINITY_DN107094_c6_g1_i2 | | 1.90 | 5.85 | -1.65 | 0.00 |
| TRINITY_DN107096_c2_g1_i4 | KLF11 | 8.85 | 2.94 | 1.55 | 0.00 |
| TRINITY_DN107112_c2_g1_i1 | MYSS | 10.69 | 1.56 | 2.72 | 0.00 |
| TRINITY_DN107130_c6_g1_i1 | | 1.47 | 0.12 | 3.79 | 0.00 |
| TRINITY_DN107140_c42_g2_i8 | AATM | 0.13 | 4.44 | -3.99 | 0.00 |
| TRINITY_DN107145_c28_g2_i1 | IRF4 | 0.57 | 3.62 | -2.47 | 0.00 |
| TRINITY_DN107146_c73_g1_i1 | LMO7 | 11.82 | 4.91 | 1.29 | 0.00 |
| TRINITY_DN107149_c53_g1_i1 | | 0.81 | 3.66 | -2.11 | 0.00 |
| TRINITY_DN107152_c11_g1_i1 | YTX2 | 1.42 | 3.03 | -1.13 | 0.00 |
| TRINITY_DN107157_c9_g2_i1 | | 30.45 | 113.16 | -1.93 | 0.00 |
| TRINITY_DN108183_c0_g1_i1 | | 1.83 | 16.34 | -3.04 | 0.00 |

| | | | | | |
|---|---|---|---|---|---|
| TRINITY_DN111156_c0_g1_i1 | | 0.09 | 7.11 | -3.81 | 0.00 |
| TRINITY_DN115242_c0_g1_i1 | | 15.28 | 47.70 | -1.58 | 0.00 |
| TRINITY_DN121623_c0_g1_i1 | | 13.72 | 47.92 | -1.83 | 0.00 |
| TRINITY_DN125187_c0_g1_i1 | | 23.63 | 8.49 | 1.45 | 0.00 |
| TRINITY_DN133738_c0_g1_i1 | HOME2 | 10.31 | 37.09 | -1.83 | 0.00 |
| TRINITY_DN1361_c0_g1_i1 | | 0.00 | 6.81 | -3.83 | 0.00 |
| TRINITY_DN23370_c0_g1_i1 | | 3.92 | 24.60 | -2.69 | 0.00 |
| TRINITY_DN23667_c0_g1_i1 | | 0.00 | 1.20 | -3.76 | 0.00 |
| TRINITY_DN3196_c0_g1_i1 | | 1.17 | 8.26 | -2.92 | 0.00 |
| TRINITY_DN36087_c0_g1_i1 | | 0.15 | 1.57 | -3.10 | 0.00 |
| TRINITY_DN38417_c0_g1_i1 | | 0.00 | 3.47 | -3.85 | 0.00 |
| TRINITY_DN43023_c0_g1_i1 | N42L1 | 0.82 | 4.25 | -2.48 | 0.00 |
| TRINITY_DN44472_c0_g1_i1 | | 2.35 | 9.38 | -1.97 | 0.00 |
| TRINITY_DN44584_c0_g1_i1 | | 11.54 | 42.83 | -1.92 | 0.00 |
| TRINITY_DN5681_c0_g1_i2 | ARL6 | 0.04 | 1.30 | -3.55 | 0.00 |
| TRINITY_DN569_c0_g2_i1 | GP119 | 2.91 | 0.29 | 3.17 | 0.00 |
| TRINITY_DN59000_c0_g1_i1 | NOD1 | 0.43 | 1.66 | -2.02 | 0.00 |
| TRINITY_DN60275_c0_g1_i4 | | 0.09 | 2.43 | -4.37 | 0.00 |
| TRINITY_DN60666_c0_g1_i1 | CLD3 | 11.15 | 77.45 | -2.82 | 0.00 |
| TRINITY_DN60727_c0_g2_i1 | | 0.00 | 0.74 | -3.76 | 0.00 |
| TRINITY_DN60944_c0_g1_i1 | | 2.10 | 6.48 | -1.69 | 0.00 |
| TRINITY_DN61172_c0_g1_i1 | | 0.81 | 3.64 | -2.37 | 0.00 |
| TRINITY_DN61948_c0_g1_i1 | | 1.33 | 12.97 | -3.18 | 0.00 |
| TRINITY_DN6588_c0_g1_i1 | | 1.64 | 7.37 | -2.22 | 0.00 |
| TRINITY_DN66397_c0_g1_i2 | | 0.00 | 16.82 | -4.43 | 0.00 |
| TRINITY_DN68307_c0_g1_i1 | CCL4 | 6.41 | 59.48 | -3.19 | 0.00 |
| TRINITY_DN71151_c0_g1_i1 | | 0.16 | 0.82 | -2.30 | 0.00 |
| TRINITY_DN71262_c1_g1_i1 | | 0.00 | 6.46 | -3.91 | 0.00 |
| TRINITY_DN71475_c0_g1_i1 | | 0.06 | 0.83 | -3.38 | 0.00 |
| TRINITY_DN71799_c0_g1_i2 | PCKGC | 1.17 | 0.25 | 2.16 | 0.00 |
| TRINITY_DN71842_c0_g1_i1 | ENDD1 | 0.00 | 2.03 | -5.76 | 0.00 |
| TRINITY_DN72285_c0_g1_i1 | P2Y11 | 0.15 | 0.77 | -2.42 | 0.00 |
| TRINITY_DN72304_c0_g1_i1 | CLTR2 | 0.72 | 2.68 | -1.93 | 0.00 |
| TRINITY_DN72734_c0_g1_i1 | | 0.10 | 3.79 | -4.42 | 0.00 |
| TRINITY_DN72749_c0_g2_i1 | S10A1 | 6.84 | 27.38 | -2.05 | 0.00 |
| TRINITY_DN73207_c0_g1_i1 | | 0.38 | 2.47 | -2.67 | 0.00 |
| TRINITY_DN73373_c0_g1_i1 | ENDD1 | 0.29 | 4.48 | -3.98 | 0.00 |
| TRINITY_DN74958_c0_g1_i1 | | 0.53 | 10.73 | -3.43 | 0.00 |
| TRINITY_DN76112_c0_g1_i1 | | 0.16 | 4.60 | -3.87 | 0.00 |
| TRINITY_DN76313_c0_g1_i1 | GPAT4 | 0.23 | 2.31 | -3.35 | 0.00 |
| TRINITY_DN76612_c0_g1_i1 | FCERG | 8.62 | 27.47 | -1.73 | 0.00 |
| TRINITY_DN76612_c0_g1_i2 | FCERG | 3.27 | 12.10 | -1.91 | 0.00 |

| | | | | | |
|---|---|---|---|---|---|
| TRINITY_DN77803_c0_g2_i1 | ALN | 1.26 | 0.05 | 4.05 | 0.00 |
| TRINITY_DN78257_c0_g1_i1 | DYR | 7.91 | 4.14 | 0.96 | 0.00 |
| TRINITY_DN78417_c0_g1_i1 | | 0.05 | 1.43 | -3.94 | 0.00 |
| TRINITY_DN78624_c0_g1_i1 | IRG1 | 0.16 | 1.37 | -3.17 | 0.00 |
| TRINITY_DN78990_c0_g1_i1 | | 0.16 | 1.63 | -3.14 | 0.00 |
| TRINITY_DN79211_c0_g1_i1 | FA5 | 0.30 | 9.20 | -3.47 | 0.00 |
| TRINITY_DN79289_c0_g1_i2 | | 1.20 | 22.07 | -3.73 | 0.00 |
| TRINITY_DN79418_c0_g1_i1 | | 26.79 | 75.02 | -1.47 | 0.00 |
| TRINITY_DN79651_c0_g1_i1 | ESPN | 0.27 | 3.39 | -3.32 | 0.00 |
| TRINITY_DN79854_c0_g1_i1 | | 2.27 | 0.20 | 3.71 | 0.00 |
| TRINITY_DN80026_c0_g1_i1 | | 0.15 | 2.98 | -3.39 | 0.00 |
| TRINITY_DN80180_c0_g1_i1 | | 0.25 | 2.82 | -3.36 | 0.00 |
| TRINITY_DN80741_c0_g1_i1 | | 106.82 | 19.21 | 2.60 | 0.00 |
| TRINITY_DN80773_c0_g2_i1 | | 0.85 | 6.42 | -2.99 | 0.00 |
| TRINITY_DN80798_c0_g1_i1 | IFM5 | 8.94 | 3.15 | 1.62 | 0.00 |
| TRINITY_DN80867_c0_g1_i1 | | 1.50 | 10.93 | -2.80 | 0.00 |
| TRINITY_DN80947_c0_g1_i1 | | 0.00 | 1.33 | -3.83 | 0.00 |
| TRINITY_DN81260_c0_g1_i1 | | 0.07 | 1.01 | -3.38 | 0.00 |
| TRINITY_DN81341_c0_g1_i4 | ST17A | 0.02 | 0.99 | -4.71 | 0.00 |
| TRINITY_DN81624_c0_g1_i2 | | 8.40 | 0.67 | 3.57 | 0.00 |
| TRINITY_DN81690_c0_g1_i1 | ADK | 3.75 | 1.35 | 1.49 | 0.00 |
| TRINITY_DN81795_c1_g1_i1 | | 0.32 | 5.59 | -3.76 | 0.00 |
| TRINITY_DN81953_c0_g1_i1 | FCGBP | 11.53 | 2.53 | 2.14 | 0.00 |
| TRINITY_DN81969_c0_g1_i1 | CLC4M | 3.77 | 0.65 | 2.45 | 0.00 |
| TRINITY_DN82017_c0_g1_i2 | NDUA4 | 13.76 | 5.75 | 1.23 | 0.00 |
| TRINITY_DN82018_c0_g1_i1 | | 27.97 | 16.07 | 0.81 | 0.00 |
| TRINITY_DN82134_c0_g1_i1 | | 0.48 | 9.43 | -3.59 | 0.00 |
| TRINITY_DN82337_c0_g1_i1 | | 0.35 | 1.49 | -2.18 | 0.00 |
| TRINITY_DN82690_c0_g1_i1 | | 0.09 | 75.89 | -9.90 | 0.00 |
| TRINITY_DN82690_c0_g1_i2 | | 19.48 | 758.38 | -5.22 | 0.00 |
| TRINITY_DN82746_c0_g1_i1 | | 2.88 | 8.73 | -1.54 | 0.00 |
| TRINITY_DN82772_c0_g1_i1 | | 6.15 | 24.64 | -1.94 | 0.00 |
| TRINITY_DN82845_c0_g1_i1 | HPRT | 0.06 | 1.83 | -3.60 | 0.00 |
| TRINITY_DN82919_c0_g1_i1 | PSB7 | 37.43 | 19.04 | 1.04 | 0.00 |
| TRINITY_DN83100_c0_g1_i1 | | 1.43 | 13.01 | -2.98 | 0.00 |
| TRINITY_DN83234_c0_g1_i2 | ZN831 | 0.10 | 1.02 | -2.96 | 0.00 |
| TRINITY_DN83451_c0_g1_i1 | | 8.67 | 32.45 | -1.97 | 0.00 |
| TRINITY_DN83639_c0_g1_i1 | | 10.71 | 65.68 | -2.73 | 0.00 |
| TRINITY_DN83856_c0_g1_i1 | | 0.00 | 6.11 | -3.96 | 0.00 |
| TRINITY_DN83889_c1_g1_i1 | TNR9 | 0.03 | 1.19 | -4.08 | 0.00 |
| TRINITY_DN83901_c0_g2_i3 | | 0.04 | 1.67 | -4.84 | 0.00 |
| TRINITY_DN83923_c0_g1_i3 | | 0.54 | 5.69 | -3.10 | 0.00 |

| | | | | | |
|---|---|---|---|---|---|
| TRINITY_DN84204_c0_g1_i1 | FGL2 | 1.48 | 0.14 | 3.62 | 0.00 |
| TRINITY_DN84224_c0_g1_i1 | | 0.00 | 2.44 | -3.84 | 0.00 |
| TRINITY_DN84359_c0_g1_i1 | | 0.30 | 11.14 | -3.91 | 0.00 |
| TRINITY_DN84385_c0_g1_i1 | AGRA2 | 1.88 | 0.18 | 3.15 | 0.00 |
| TRINITY_DN84508_c0_g1_i1 | R213A | 0.23 | 2.04 | -3.01 | 0.00 |
| TRINITY_DN84605_c0_g1_i1 | | 11.77 | 39.05 | -1.73 | 0.00 |
| TRINITY_DN84672_c0_g1_i1 | ENDD1 | 0.02 | 1.52 | -4.98 | 0.00 |
| TRINITY_DN84740_c0_g1_i1 | MRP1 | 0.57 | 4.51 | -2.76 | 0.00 |
| TRINITY_DN84762_c0_g1_i1 | SLAP2 | 0.08 | 1.19 | -3.84 | 0.00 |
| TRINITY_DN84762_c0_g2_i1 | SLAP2 | 0.35 | 2.69 | -2.96 | 0.00 |
| TRINITY_DN84928_c0_g1_i3 | | 107.24 | 384.61 | -1.78 | 0.00 |
| TRINITY_DN84955_c1_g1_i1 | PGFRL | 33.84 | 16.41 | 1.07 | 0.00 |
| TRINITY_DN84985_c0_g1_i1 | | 25.83 | 92.38 | -1.86 | 0.00 |
| TRINITY_DN85079_c0_g1_i1 | RABP1 | 3.38 | 0.79 | 2.13 | 0.00 |
| TRINITY_DN85289_c0_g1_i1 | CCL3 | 0.26 | 5.95 | -4.48 | 0.00 |
| TRINITY_DN85306_c0_g1_i1 | MRP3 | 3.89 | 17.20 | -2.14 | 0.00 |
| TRINITY_DN85316_c0_g1_i1 | | 0.27 | 9.76 | -3.89 | 0.00 |
| TRINITY_DN85388_c0_g1_i1 | | 0.12 | 0.99 | -2.73 | 0.00 |
| TRINITY_DN85452_c0_g1_i1 | CXG1 | 1.79 | 0.41 | 2.14 | 0.00 |
| TRINITY_DN85491_c0_g1_i1 | | 2.93 | 1.01 | 1.55 | 0.00 |
| TRINITY_DN85560_c0_g1_i1 | | 1.58 | 0.45 | 1.75 | 0.00 |
| TRINITY_DN85611_c0_g1_i1 | EVI2A | 4.11 | 11.26 | -1.51 | 0.00 |
| TRINITY_DN85717_c0_g1_i1 | | 10.69 | 59.42 | -2.47 | 0.00 |
| TRINITY_DN85732_c0_g2_i1 | | 0.98 | 15.14 | -3.92 | 0.00 |
| TRINITY_DN85766_c0_g1_i1 | | 1.04 | 5.21 | -2.39 | 0.00 |
| TRINITY_DN85777_c0_g1_i2 | TM35A | 1.35 | 3.11 | -1.23 | 0.00 |
| TRINITY_DN85808_c0_g1_i2 | CA123 | 0.36 | 2.56 | -3.03 | 0.00 |
| TRINITY_DN85928_c0_g1_i1 | | 5.58 | 29.07 | -2.42 | 0.00 |
| TRINITY_DN85937_c1_g1_i1 | FARX | 0.57 | 9.17 | -3.05 | 0.00 |
| TRINITY_DN86001_c0_g1_i2 | SLAP1 | 12.83 | 39.99 | -1.65 | 0.00 |
| TRINITY_DN86039_c0_g1_i2 | | 0.00 | 20.14 | -6.36 | 0.00 |
| TRINITY_DN86106_c1_g1_i1 | | 0.94 | 7.64 | -3.16 | 0.00 |
| TRINITY_DN86106_c1_g2_i4 | | 0.77 | 9.68 | -3.66 | 0.00 |
| TRINITY_DN86195_c0_g1_i1 | | 40.64 | 84.22 | -1.07 | 0.00 |
| TRINITY_DN86197_c0_g1_i1 | | 0.05 | 3.16 | -4.49 | 0.00 |
| TRINITY_DN86214_c0_g1_i1 | GPR35 | 0.27 | 1.42 | -2.38 | 0.00 |
| TRINITY_DN86254_c0_g1_i1 | | 111.75 | 631.83 | -2.64 | 0.00 |
| TRINITY_DN86290_c0_g1_i1 | USMG5 | 178.91 | 98.78 | 0.82 | 0.00 |
| TRINITY_DN86328_c0_g1_i1 | | 0.12 | 2.11 | -3.73 | 0.00 |
| TRINITY_DN86407_c0_g1_i4 | | 0.00 | 1.82 | -4.00 | 0.00 |
| TRINITY_DN86415_c0_g1_i3 | KLK8 | 0.00 | 8.56 | -7.50 | 0.00 |
| TRINITY_DN86467_c0_g1_i1 | | 0.36 | 4.33 | -3.46 | 0.00 |

| | | | | | |
|---|---|---|---|---|---|
| TRINITY_DN86469_c0_g2_i3 | FAM3C | 3.98 | 13.84 | -1.79 | 0.00 |
| TRINITY_DN86495_c0_g1_i1 | RABP2 | 9.40 | 3.87 | 1.30 | 0.00 |
| TRINITY_DN86512_c0_g1_i4 | SMD3 | 12.60 | 18.66 | -0.59 | 0.00 |
| TRINITY_DN86527_c0_g1_i1 | | 5.95 | 16.53 | -1.50 | 0.00 |
| TRINITY_DN86611_c0_g1_i1 | | 5.82 | 39.97 | -2.78 | 0.00 |
| TRINITY_DN86628_c0_g2_i1 | PERE | 0.19 | 3.85 | -4.27 | 0.00 |
| TRINITY_DN86633_c0_g1_i1 | NF7O | 1.26 | 2.68 | -1.14 | 0.00 |
| TRINITY_DN86679_c0_g1_i1 | | 1.32 | 0.43 | 1.59 | 0.00 |
| TRINITY_DN86706_c0_g2_i1 | | 0.26 | 2.48 | -3.09 | 0.00 |
| TRINITY_DN86759_c0_g1_i1 | | 3.02 | 0.12 | 4.36 | 0.00 |
| TRINITY_DN86773_c0_g2_i1 | C1QL4 | 27.83 | 11.92 | 1.22 | 0.00 |
| TRINITY_DN86777_c0_g1_i1 | | 5.07 | 1.24 | 2.09 | 0.00 |
| TRINITY_DN86849_c0_g1_i5 | | 1.25 | 7.39 | -2.37 | 0.00 |
| TRINITY_DN86887_c0_g1_i3 | CCL20 | 0.53 | 9.58 | -4.30 | 0.00 |
| TRINITY_DN86901_c0_g1_i2 | | 0.94 | 0.06 | 3.47 | 0.00 |
| TRINITY_DN86927_c0_g1_i1 | PANX3 | 1.01 | 0.14 | 2.77 | 0.00 |
| TRINITY_DN86965_c0_g1_i2 | | 0.84 | 7.60 | -3.14 | 0.00 |
| TRINITY_DN86965_c0_g2_i1 | UBIL | 5.46 | 47.39 | -3.11 | 0.00 |
| TRINITY_DN87020_c0_g2_i1 | | 4.35 | 8.79 | -1.01 | 0.00 |
| TRINITY_DN87120_c0_g2_i1 | IRG1 | 0.04 | 1.37 | -3.55 | 0.00 |
| TRINITY_DN87120_c0_g3_i2 | | 1.40 | 16.65 | -3.28 | 0.00 |
| TRINITY_DN87169_c0_g1_i3 | DTX3L | 54.09 | 138.42 | -1.32 | 0.00 |
| TRINITY_DN87180_c0_g1_i3 | SLK | 0.41 | 4.57 | -3.44 | 0.00 |
| TRINITY_DN87219_c0_g1_i3 | | 7.61 | 0.44 | 3.71 | 0.00 |
| TRINITY_DN87241_c0_g1_i2 | | 0.78 | 8.89 | -3.36 | 0.00 |
| TRINITY_DN87242_c2_g1_i1 | | 2.69 | 15.28 | -2.51 | 0.00 |
| TRINITY_DN87242_c2_g2_i1 | | 0.17 | 3.64 | -4.19 | 0.00 |
| TRINITY_DN87323_c0_g1_i1 | GPR4 | 0.46 | 1.54 | -1.67 | 0.00 |
| TRINITY_DN87416_c0_g4_i2 | | 0.00 | 5.13 | -6.69 | 0.00 |
| TRINITY_DN87431_c0_g1_i1 | | 0.43 | 2.67 | -2.60 | 0.00 |
| TRINITY_DN87431_c0_g1_i3 | | 0.25 | 1.63 | -2.79 | 0.00 |
| TRINITY_DN87479_c0_g2_i1 | | 2.60 | 1.21 | 1.15 | 0.00 |
| TRINITY_DN87510_c0_g1_i3 | | 25.39 | 11.58 | 1.12 | 0.00 |
| TRINITY_DN87516_c0_g1_i1 | SAPC1 | 3.70 | 1.05 | 1.86 | 0.00 |
| TRINITY_DN87535_c0_g2_i2 | | 0.59 | 2.51 | -2.16 | 0.00 |
| TRINITY_DN87548_c0_g1_i9 | | 52.18 | 163.67 | -1.65 | 0.00 |
| TRINITY_DN87553_c0_g1_i3 | | 0.41 | 3.53 | -3.03 | 0.00 |
| TRINITY_DN87560_c0_g1_i2 | IGSF6 | 3.96 | 9.19 | -1.28 | 0.00 |
| TRINITY_DN87577_c0_g1_i1 | | 5.72 | 42.50 | -2.85 | 0.00 |
| TRINITY_DN87635_c0_g2_i2 | IL17F | 0.09 | 2.73 | -4.53 | 0.00 |
| TRINITY_DN87702_c0_g1_i1 | | 38.69 | 161.81 | -2.06 | 0.00 |
| TRINITY_DN87721_c0_g1_i1 | | 244.71 | 128.60 | 1.02 | 0.00 |

| | | | | | |
|---|---|---|---|---|---|
| TRINITY_DN87790_c0_g1_i3 | | 0.53 | 3.36 | -2.72 | 0.00 |
| TRINITY_DN87876_c0_g1_i1 | IEX1 | 9.66 | 23.38 | -1.30 | 0.00 |
| TRINITY_DN87878_c0_g1_i2 | IMP1L | 14.35 | 6.15 | 1.21 | 0.00 |
| TRINITY_DN87879_c0_g1_i1 | | 10.14 | 21.80 | -1.14 | 0.00 |
| TRINITY_DN87879_c0_g2_i1 | | 5.30 | 12.23 | -1.22 | 0.00 |
| TRINITY_DN87884_c0_g1_i1 | PLC | 0.18 | 1.56 | -3.11 | 0.00 |
| TRINITY_DN87922_c0_g1_i1 | LYG | 159.92 | 298.46 | -0.88 | 0.00 |
| TRINITY_DN87922_c0_g1_i4 | LYG | 38.54 | 63.81 | -0.75 | 0.00 |
| TRINITY_DN87925_c0_g1_i3 | SAM12 | 10.87 | 7.04 | 0.62 | 0.00 |
| TRINITY_DN87938_c0_g1_i4 | | 0.84 | 3.21 | -1.95 | 0.00 |
| TRINITY_DN87974_c0_g1_i1 | | 8.75 | 30.82 | -1.77 | 0.00 |
| TRINITY_DN87995_c0_g1_i1 | PPT2A | 2.28 | 6.12 | -1.40 | 0.00 |
| TRINITY_DN87999_c0_g3_i1 | | 0.43 | 2.28 | -2.41 | 0.00 |
| TRINITY_DN88002_c0_g1_i1 | LONF3 | 1.19 | 0.54 | 1.14 | 0.00 |
| TRINITY_DN88023_c0_g1_i1 | | 0.80 | 9.69 | -3.70 | 0.00 |
| TRINITY_DN88023_c0_g2_i1 | | 26.46 | 175.19 | -2.67 | 0.00 |
| TRINITY_DN88042_c0_g1_i1 | | 1.15 | 10.12 | -3.14 | 0.00 |
| TRINITY_DN88078_c0_g2_i1 | ZN366 | 2.04 | 4.97 | -1.30 | 0.00 |
| TRINITY_DN88081_c0_g1_i1 | | 7.59 | 3.92 | 0.95 | 0.00 |
| TRINITY_DN88082_c0_g1_i1 | | 18.57 | 55.02 | -1.62 | 0.00 |
| TRINITY_DN88099_c0_g1_i1 | | 0.46 | 7.90 | -3.41 | 0.00 |
| TRINITY_DN88099_c0_g1_i10 | | 2.23 | 41.29 | -4.47 | 0.00 |
| TRINITY_DN88099_c0_g1_i3 | | 0.07 | 1.41 | -3.69 | 0.00 |
| TRINITY_DN88100_c0_g1_i1 | | 6.55 | 73.77 | -3.50 | 0.00 |
| TRINITY_DN88121_c0_g1_i1 | | 4.09 | 21.12 | -2.40 | 0.00 |
| TRINITY_DN88155_c0_g1_i2 | | 14.12 | 33.02 | -1.24 | 0.00 |
| TRINITY_DN88178_c0_g1_i1 | | 15.26 | 47.05 | -1.61 | 0.00 |
| TRINITY_DN88189_c0_g2_i1 | RIC3 | 1.54 | 5.29 | -1.76 | 0.00 |
| TRINITY_DN88215_c1_g3_i2 | COLL2 | 0.66 | 18.67 | -4.02 | 0.00 |
| TRINITY_DN88251_c4_g1_i2 | TRI63 | 2.38 | 0.61 | 1.90 | 0.00 |
| TRINITY_DN88280_c0_g2_i3 | | 2.60 | 12.21 | -2.32 | 0.00 |
| TRINITY_DN88290_c1_g1_i5 | | 1.84 | 6.97 | -1.92 | 0.00 |
| TRINITY_DN88299_c0_g1_i2 | T3JAM | 0.23 | 2.94 | -3.76 | 0.00 |
| TRINITY_DN88300_c2_g1_i1 | CCL2 | 6.34 | 14.08 | -1.19 | 0.00 |
| TRINITY_DN88326_c0_g2_i1 | APOC1 | 207.41 | 70.29 | 1.73 | 0.00 |
| TRINITY_DN88368_c0_g1_i2 | CCL4 | 0.08 | 3.42 | -4.97 | 0.00 |
| TRINITY_DN88392_c0_g1_i1 | | 71.57 | 175.83 | -1.32 | 0.00 |
| TRINITY_DN88394_c0_g1_i2 | FADD | 8.13 | 13.24 | -0.71 | 0.00 |
| TRINITY_DN88496_c0_g1_i1 | | 28.61 | 10.05 | 0.79 | 0.00 |
| TRINITY_DN88515_c0_g1_i3 | | 0.24 | 6.34 | -4.09 | 0.00 |
| TRINITY_DN88548_c0_g1_i3 | CCL19 | 10.94 | 42.05 | -1.81 | 0.00 |
| TRINITY_DN88556_c0_g1_i1 | IF44L | 0.01 | 1.63 | -5.63 | 0.00 |

| | | | | | |
|---|---|---|---|---|---|
| TRINITY_DN88556_c0_g1_i2 | IF44L | 0.11 | 5.79 | -5.79 | 0.00 |
| TRINITY_DN88556_c0_g1_i3 | IF44L | 0.03 | 2.50 | -5.74 | 0.00 |
| TRINITY_DN88572_c1_g1_i1 | M1I1B | 34.61 | 57.95 | -0.73 | 0.00 |
| TRINITY_DN88592_c3_g3_i1 | | 0.08 | 1.29 | -3.26 | 0.00 |
| TRINITY_DN88616_c1_g1_i4 | | 2.09 | 11.67 | -2.45 | 0.00 |
| TRINITY_DN88616_c1_g1_i9 | | 3.04 | 23.21 | -3.03 | 0.00 |
| TRINITY_DN88616_c1_g2_i1 | | 2.23 | 10.86 | -2.41 | 0.00 |
| TRINITY_DN88616_c1_g2_i4 | CD3D | 2.67 | 13.84 | -2.35 | 0.00 |
| TRINITY_DN88616_c1_g3_i1 | | 3.68 | 18.07 | -2.42 | 0.00 |
| TRINITY_DN88616_c1_g3_i2 | | 39.97 | 233.64 | -2.59 | 0.00 |
| TRINITY_DN88634_c0_g1_i5 | | 12.13 | 31.07 | -1.32 | 0.00 |
| TRINITY_DN88640_c13_g1_i3 | | 0.45 | 2.51 | -2.45 | 0.00 |
| TRINITY_DN88650_c6_g1_i1 | | 0.14 | 1.89 | -3.40 | 0.00 |
| TRINITY_DN88668_c0_g1_i2 | T255B | 2.27 | 0.77 | 1.58 | 0.00 |
| TRINITY_DN88675_c0_g1_i2 | GBG13 | 2.67 | 22.45 | -3.07 | 0.00 |
| TRINITY_DN88687_c3_g1_i2 | | 0.75 | 6.69 | -3.22 | 0.00 |
| TRINITY_DN88715_c0_g1_i1 | | 2.95 | 111.92 | -5.22 | 0.00 |
| TRINITY_DN88740_c0_g1_i1 | | 4.61 | 12.79 | -1.46 | 0.00 |
| TRINITY_DN88740_c0_g3_i1 | SELPL | 4.03 | 12.05 | -1.60 | 0.00 |
| TRINITY_DN88741_c3_g1_i1 | | 2.03 | 6.74 | -1.62 | 0.00 |
| TRINITY_DN88742_c0_g1_i1 | | 31.57 | 113.66 | -1.88 | 0.00 |
| TRINITY_DN88750_c0_g4_i2 | | 0.16 | 1.21 | -3.22 | 0.00 |
| TRINITY_DN88758_c0_g1_i2 | | 3.05 | 0.30 | 3.50 | 0.00 |
| TRINITY_DN88765_c0_g1_i1 | | 1.37 | 26.02 | -4.27 | 0.00 |
| TRINITY_DN88765_c0_g1_i2 | | 0.13 | 4.72 | -5.17 | 0.00 |
| TRINITY_DN88765_c0_g1_i3 | | 0.19 | 5.80 | -5.11 | 0.00 |
| TRINITY_DN88765_c0_g1_i5 | | 0.94 | 11.01 | -3.65 | 0.00 |
| TRINITY_DN88794_c8_g1_i1 | | 1.33 | 3.18 | -1.27 | 0.00 |
| TRINITY_DN88823_c0_g1_i1 | | 0.21 | 2.28 | -3.13 | 0.00 |
| TRINITY_DN88855_c0_g3_i1 | NADL2 | 2.33 | 3.77 | -0.72 | 0.00 |
| TRINITY_DN88867_c0_g3_i2 | F111A | 0.00 | 2.30 | -5.17 | 0.00 |
| TRINITY_DN88881_c2_g1_i1 | | 2.52 | 14.97 | -2.44 | 0.00 |
| TRINITY_DN88892_c0_g1_i1 | OLF3A | 23.81 | 12.66 | 0.90 | 0.00 |
| TRINITY_DN88911_c0_g1_i10 | | 1.91 | 0.45 | 2.08 | 0.00 |
| TRINITY_DN88930_c2_g1_i2 | | 1.15 | 11.37 | -3.54 | 0.00 |
| TRINITY_DN88970_c12_g1_i1 | | 0.28 | 2.07 | -2.69 | 0.00 |
| TRINITY_DN88987_c3_g1_i10 | CCL4 | 0.08 | 29.67 | -7.40 | 0.00 |
| TRINITY_DN88987_c3_g1_i12 | CCL4 | 0.27 | 16.70 | -5.83 | 0.00 |
| TRINITY_DN88987_c3_g1_i4 | CCL4 | 0.00 | 23.06 | -22.36 | 0.00 |
| TRINITY_DN88987_c3_g3_i1 | | 1.51 | 41.83 | -4.82 | 0.00 |
| TRINITY_DN88987_c3_g7_i1 | | 8.69 | 166.77 | -4.28 | 0.00 |
| TRINITY_DN88997_c0_g2_i6 | CX6C1 | 0.00 | 32.58 | -8.26 | 0.00 |

| | | | | | |
|---|---|---|---|---|---|
| TRINITY_DN88999_c3_g1_i1 | HSP70 | 28.77 | 11.64 | 1.31 | 0.00 |
| TRINITY_DN89008_c0_g5_i3 | GRP3 | 0.09 | 0.81 | -3.20 | 0.00 |
| TRINITY_DN89012_c0_g1_i2 | DAPP1 | 1.72 | 7.39 | -2.06 | 0.00 |
| TRINITY_DN89020_c0_g1_i5 | LEG3 | 0.79 | 0.03 | 4.31 | 0.00 |
| TRINITY_DN89029_c3_g1_i3 | | 0.00 | 2.94 | -5.25 | 0.00 |
| TRINITY_DN89040_c3_g1_i2 | | 21.85 | 2.51 | 3.21 | 0.00 |
| TRINITY_DN89042_c1_g1_i4 | | 186.53 | 83.28 | 1.14 | 0.00 |
| TRINITY_DN89060_c6_g5_i1 | | 25.22 | 88.37 | -1.84 | 0.00 |
| TRINITY_DN89065_c5_g1_i1 | | 3.44 | 17.77 | -2.45 | 0.00 |
| TRINITY_DN89087_c3_g1_i8 | CD6 | 0.26 | 4.26 | -4.09 | 0.00 |
| TRINITY_DN89099_c0_g1_i2 | | 10.98 | 66.77 | -2.59 | 0.00 |
| TRINITY_DN89099_c0_g1_i8 | | 8.55 | 28.45 | -1.76 | 0.00 |
| TRINITY_DN89113_c0_g1_i1 | PLBL1 | 7.12 | 14.08 | -0.97 | 0.00 |
| TRINITY_DN89132_c0_g1_i2 | HPSE | 3.55 | 9.72 | -1.46 | 0.00 |
| TRINITY_DN89133_c0_g1_i6 | STAT3 | 2.28 | 0.10 | 4.61 | 0.00 |
| TRINITY_DN89163_c0_g1_i1 | | 0.00 | 0.96 | -3.81 | 0.00 |
| TRINITY_DN89172_c2_g1_i1 | | 20.40 | 3.69 | 2.46 | 0.00 |
| TRINITY_DN89278_c0_g1_i1 | | 0.00 | 14.45 | -8.47 | 0.00 |
| TRINITY_DN89278_c0_g1_i3 | | 38.00 | 6.68 | 2.55 | 0.00 |
| TRINITY_DN89285_c0_g1_i11 | C1QA | 0.00 | 9.47 | -20.66 | 0.00 |
| TRINITY_DN89292_c0_g1_i4 | MYPR | 1.13 | 0.00 | 5.73 | 0.00 |
| TRINITY_DN89303_c11_g1_i1 | | 5.94 | 2.04 | 1.55 | 0.00 |
| TRINITY_DN89331_c0_g1_i1 | | 0.75 | 13.37 | -4.12 | 0.00 |
| TRINITY_DN89337_c2_g1_i1 | BIN3 | 13.37 | 5.45 | 1.29 | 0.00 |
| TRINITY_DN89341_c2_g7_i1 | | 4.15 | 12.71 | -1.53 | 0.00 |
| TRINITY_DN89390_c2_g1_i1 | | 3.22 | 9.92 | -1.62 | 0.00 |
| TRINITY_DN89398_c6_g1_i10 | RS12 | 463.26 | 157.44 | 1.70 | 0.00 |
| TRINITY_DN89404_c0_g2_i2 | CCR6 | 0.37 | 4.99 | -3.75 | 0.00 |
| TRINITY_DN89409_c0_g2_i4 | CID2A | 2.28 | 6.02 | -1.42 | 0.00 |
| TRINITY_DN89413_c0_g1_i2 | | 3.28 | 10.96 | -1.73 | 0.00 |
| TRINITY_DN89414_c2_g1_i3 | MAG | 0.38 | 2.25 | -2.58 | 0.00 |
| TRINITY_DN89415_c6_g2_i8 | CBL | 0.14 | 1.86 | -3.88 | 0.00 |
| TRINITY_DN89419_c3_g1_i3 | CRIP2 | 6.72 | 26.45 | -2.02 | 0.00 |
| TRINITY_DN89428_c0_g1_i14 | | 1.40 | 0.03 | 5.51 | 0.00 |
| TRINITY_DN89447_c6_g1_i3 | | 0.22 | 3.35 | -3.69 | 0.00 |
| TRINITY_DN89448_c4_g1_i2 | | 0.00 | 12.52 | -4.00 | 0.00 |
| TRINITY_DN89472_c9_g1_i1 | | 0.41 | 2.70 | -2.72 | 0.00 |
| TRINITY_DN89500_c3_g1_i2 | NACA | 0.06 | 0.94 | -3.59 | 0.00 |
| TRINITY_DN89501_c4_g1_i1 | | 0.66 | 40.50 | -5.88 | 0.00 |
| TRINITY_DN89501_c4_g1_i10 | | 0.02 | 4.54 | -6.96 | 0.00 |
| TRINITY_DN89501_c4_g1_i2 | | 0.23 | 12.32 | -5.74 | 0.00 |
| TRINITY_DN89501_c4_g1_i3 | | 0.14 | 6.47 | -5.42 | 0.00 |

| | | | | | |
|---|---|---|---|---|---|
| TRINITY_DN89501_c4_g1_i4 | | 0.04 | 7.44 | -7.15 | 0.00 |
| TRINITY_DN89501_c4_g1_i5 | | 0.04 | 7.48 | -7.05 | 0.00 |
| TRINITY_DN89501_c4_g1_i7 | | 0.13 | 5.69 | -5.34 | 0.00 |
| TRINITY_DN89501_c4_g1_i8 | | 0.08 | 10.28 | -7.13 | 0.00 |
| TRINITY_DN89506_c8_g1_i2 | | 1.67 | 9.22 | -2.45 | 0.00 |
| TRINITY_DN89517_c0_g2_i1 | | 12.58 | 82.14 | -2.73 | 0.00 |
| TRINITY_DN89543_c3_g1_i1 | | 0.72 | 4.10 | -2.52 | 0.00 |
| TRINITY_DN89567_c1_g2_i4 | | 0.05 | 2.39 | -4.03 | 0.00 |
| TRINITY_DN89567_c1_g2_i6 | | 1.40 | 6.43 | -2.09 | 0.00 |
| TRINITY_DN89567_c1_g3_i2 | | 0.17 | 7.22 | -5.27 | 0.00 |
| TRINITY_DN89569_c3_g1_i1 | | 12.62 | 0.12 | 4.66 | 0.00 |
| TRINITY_DN89572_c4_g8_i1 | IF1AX | 11.04 | 1.14 | 3.19 | 0.00 |
| TRINITY_DN89600_c0_g5_i1 | | 2.64 | 0.78 | 1.76 | 0.00 |
| TRINITY_DN89604_c2_g1_i15 | VIME | 43.61 | 28.12 | 0.62 | 0.00 |
| TRINITY_DN89604_c2_g1_i9 | VIME | 6.87 | 0.91 | 3.17 | 0.00 |
| TRINITY_DN89604_c2_g2_i2 | VIME | 2.22 | 0.47 | 2.39 | 0.00 |
| TRINITY_DN89605_c5_g1_i1 | NMES1 | 36.97 | 70.38 | -0.96 | 0.00 |
| TRINITY_DN89624_c0_g1_i1 | CO2A1 | 6.95 | 1.39 | 2.35 | 0.00 |
| TRINITY_DN89660_c0_g2_i1 | | 0.05 | 0.70 | -3.25 | 0.00 |
| TRINITY_DN89660_c1_g1_i14 | | 0.68 | 5.19 | -2.79 | 0.00 |
| TRINITY_DN89660_c1_g1_i15 | | 0.30 | 8.50 | -3.97 | 0.00 |
| TRINITY_DN89677_c0_g1_i5 | | 0.32 | 0.02 | 4.03 | 0.00 |
| TRINITY_DN89682_c2_g1_i7 | RBM28 | 0.07 | 1.29 | -4.03 | 0.00 |
| TRINITY_DN89690_c0_g3_i1 | ADT2 | 62.59 | 39.40 | 0.68 | 0.00 |
| TRINITY_DN89693_c5_g1_i1 | | 0.07 | 0.92 | -3.41 | 0.00 |
| TRINITY_DN89706_c6_g3_i1 | PD1L1 | 1.15 | 2.50 | -1.12 | 0.00 |
| TRINITY_DN89709_c0_g1_i1 | AATF | 0.00 | 1.57 | -4.10 | 0.00 |
| TRINITY_DN89730_c7_g2_i6 | PTGIS | 0.19 | 0.71 | -1.98 | 0.00 |
| TRINITY_DN89737_c0_g1_i4 | RM46 | 7.77 | 1.02 | 2.85 | 0.00 |
| TRINITY_DN89737_c0_g1_i5 | RM46 | 0.17 | 2.35 | -3.84 | 0.00 |
| TRINITY_DN89745_c2_g3_i1 | | 5.41 | 0.24 | 4.76 | 0.00 |
| TRINITY_DN89746_c0_g1_i3 | RET4B | 430.62 | 194.42 | 1.18 | 0.00 |
| TRINITY_DN89746_c0_g1_i8 | RET4A | 70.61 | 34.31 | 1.09 | 0.00 |
| TRINITY_DN89774_c3_g1_i11 | IVD | 0.00 | 1.54 | -5.67 | 0.00 |
| TRINITY_DN89782_c4_g1_i1 | | 0.00 | 10.87 | -4.26 | 0.00 |
| TRINITY_DN89789_c0_g1_i2 | | 0.00 | 8.33 | -7.17 | 0.00 |
| TRINITY_DN89798_c4_g1_i3 | | 37.16 | 18.85 | 1.06 | 0.00 |
| TRINITY_DN89802_c0_g1_i2 | | 3.58 | 37.63 | -3.27 | 0.00 |
| TRINITY_DN89802_c0_g1_i3 | | 9.18 | 40.61 | -2.19 | 0.00 |
| TRINITY_DN89804_c0_g1_i2 | | 0.89 | 2.13 | -1.23 | 0.00 |
| TRINITY_DN89816_c2_g2_i1 | TPSN | 14.31 | 43.75 | -1.63 | 0.00 |
| TRINITY_DN89820_c0_g1_i3 | TNFA | 0.16 | 1.11 | -2.96 | 0.00 |

| | | | | | |
|---|---|---|---|---|---|
| TRINITY_DN89822_c9_g2_i2 | | 0.81 | 0.00 | 5.38 | 0.00 |
| TRINITY_DN89826_c4_g3_i2 | | 5.60 | 2.66 | 1.03 | 0.00 |
| TRINITY_DN89834_c2_g1_i3 | TNF14 | 0.00 | 0.90 | -4.93 | 0.00 |
| TRINITY_DN89845_c3_g1_i1 | | 0.70 | 8.94 | -3.54 | 0.00 |
| TRINITY_DN89847_c0_g2_i7 | B4GT1 | 1.12 | 5.41 | -2.25 | 0.00 |
| TRINITY_DN89880_c0_g1_i1 | TRI36 | 0.24 | 0.91 | -1.92 | 0.00 |
| TRINITY_DN89885_c0_g1_i1 | | 3.13 | 9.74 | -1.65 | 0.00 |
| TRINITY_DN89896_c9_g1_i2 | | 0.41 | 2.62 | -2.69 | 0.00 |
| TRINITY_DN89896_c9_g1_i3 | | 23.91 | 157.91 | -2.79 | 0.00 |
| TRINITY_DN89915_c3_g2_i1 | | 2.66 | 22.40 | -2.90 | 0.00 |
| TRINITY_DN89940_c3_g1_i1 | APOH | 6.73 | 1.19 | 2.60 | 0.00 |
| TRINITY_DN89952_c0_g1_i2 | NUBP2 | 0.00 | 3.23 | -6.59 | 0.00 |
| TRINITY_DN89955_c2_g1_i2 | CIKS | 0.32 | 1.36 | -2.11 | 0.00 |
| TRINITY_DN89957_c0_g2_i1 | | 0.23 | 2.83 | -3.38 | 0.00 |
| TRINITY_DN89957_c0_g6_i1 | | 0.40 | 5.86 | -3.24 | 0.00 |
| TRINITY_DN89958_c2_g1_i1 | | 0.43 | 4.08 | -2.98 | 0.00 |
| TRINITY_DN89958_c2_g2_i2 | CLC4F | 0.00 | 1.35 | -4.95 | 0.00 |
| TRINITY_DN89965_c10_g2_i1 | | 23.68 | 16.69 | 0.51 | 0.00 |
| TRINITY_DN89967_c8_g1_i1 | COTL1 | 91.33 | 172.18 | -0.94 | 0.00 |
| TRINITY_DN89967_c8_g1_i2 | COTL1 | 92.47 | 149.25 | -0.73 | 0.00 |
| TRINITY_DN89968_c4_g1_i2 | ARHG3 | 7.54 | 23.17 | -1.66 | 0.00 |
| TRINITY_DN89968_c5_g2_i3 | | 1.54 | 5.15 | -1.73 | 0.00 |
| TRINITY_DN89968_c5_g3_i1 | | 0.44 | 6.10 | -3.23 | 0.00 |
| TRINITY_DN89977_c9_g1_i2 | | 1.28 | 12.33 | -3.22 | 0.00 |
| TRINITY_DN90017_c0_g1_i1 | CTHR1 | 5.22 | 2.22 | 1.26 | 0.00 |
| TRINITY_DN90039_c5_g1_i2 | | 64.67 | 20.73 | 1.70 | 0.00 |
| TRINITY_DN90056_c8_g1_i1 | | 1.13 | 13.41 | -3.40 | 0.00 |
| TRINITY_DN90057_c4_g1_i2 | SH21A | 2.75 | 11.37 | -2.09 | 0.00 |
| TRINITY_DN90068_c1_g5_i1 | | 6.34 | 0.22 | 4.69 | 0.00 |
| TRINITY_DN90069_c0_g4_i1 | | 0.81 | 3.80 | -2.20 | 0.00 |
| TRINITY_DN90076_c0_g1_i1 | | 2.38 | 9.62 | -2.01 | 0.00 |
| TRINITY_DN90085_c7_g2_i2 | | 2.19 | 0.73 | 1.63 | 0.00 |
| TRINITY_DN90087_c0_g1_i4 | | 0.02 | 1.09 | -4.88 | 0.00 |
| TRINITY_DN90104_c0_g1_i2 | GCNT3 | 0.11 | 1.00 | -3.15 | 0.00 |
| TRINITY_DN90140_c6_g3_i1 | | 2.69 | 15.39 | -2.56 | 0.00 |
| TRINITY_DN90144_c2_g4_i2 | | 36.86 | 106.19 | -1.45 | 0.00 |
| TRINITY_DN90150_c3_g3_i7 | | 8.86 | 28.96 | -1.70 | 0.00 |
| TRINITY_DN90157_c5_g2_i1 | | 0.07 | 2.77 | -3.80 | 0.00 |
| TRINITY_DN90157_c5_g9_i1 | | 1.52 | 0.11 | 3.61 | 0.00 |
| TRINITY_DN90165_c5_g2_i1 | | 20.85 | 82.25 | -1.96 | 0.00 |
| TRINITY_DN90169_c8_g1_i1 | | 0.11 | 2.94 | -4.04 | 0.00 |
| TRINITY_DN90176_c0_g1_i3 | M1I1B | 1.13 | 2.80 | -1.34 | 0.00 |

| | | | | | |
|---|---|---|---|---|---|
| TRINITY_DN90186_c5_g4_i1 | | 5.66 | 35.63 | -2.52 | 0.00 |
| TRINITY_DN90203_c10_g1_i2 | RNLS | 0.04 | 1.24 | -3.63 | 0.00 |
| TRINITY_DN90208_c0_g1_i1 | HMX2 | 0.05 | 1.17 | -4.24 | 0.00 |
| TRINITY_DN90208_c0_g1_i3 | HMX2 | 0.68 | 1.96 | -1.50 | 0.00 |
| TRINITY_DN90211_c10_g1_i3 | PSB7 | 27.85 | 50.04 | -0.88 | 0.00 |
| TRINITY_DN90217_c3_g1_i3 | SAMN1 | 2.27 | 5.64 | -1.30 | 0.00 |
| TRINITY_DN90233_c1_g2_i1 | GBP4 | 0.26 | 4.31 | -3.97 | 0.00 |
| TRINITY_DN90259_c6_g1_i2 | | 1.86 | 15.00 | -2.97 | 0.00 |
| TRINITY_DN90274_c8_g3_i1 | | 0.00 | 3.18 | -6.38 | 0.00 |
| TRINITY_DN90275_c7_g1_i1 | | 0.83 | 4.01 | -2.33 | 0.00 |
| TRINITY_DN90293_c1_g1_i1 | PPIP1 | 0.55 | 2.04 | -1.84 | 0.00 |
| TRINITY_DN90314_c5_g6_i1 | | 0.26 | 1.37 | -2.27 | 0.00 |
| TRINITY_DN90352_c0_g3_i4 | TWST2 | 1.98 | 0.64 | 1.69 | 0.00 |
| TRINITY_DN90357_c4_g2_i8 | | 16.70 | 90.75 | -2.58 | 0.00 |
| TRINITY_DN90368_c10_g1_i1 | CLD4 | 1.27 | 4.91 | -1.95 | 0.00 |
| TRINITY_DN90378_c0_g1_i5 | MFAP2 | 36.66 | 19.30 | 0.96 | 0.00 |
| TRINITY_DN90382_c3_g4_i1 | | 0.43 | 1.97 | -2.11 | 0.00 |
| TRINITY_DN90413_c0_g1_i1 | CLC4F | 0.92 | 4.70 | -2.36 | 0.00 |
| TRINITY_DN90424_c0_g1_i2 | JUNB | 6.25 | 3.00 | 1.04 | 0.00 |
| TRINITY_DN90431_c1_g1_i2 | SAMN1 | 1.37 | 5.90 | -2.17 | 0.00 |
| TRINITY_DN90431_c1_g1_i3 | SAMN1 | 0.98 | 2.35 | -1.28 | 0.00 |
| TRINITY_DN90450_c6_g2_i3 | F213A | 1.90 | 5.33 | -1.57 | 0.00 |
| TRINITY_DN90455_c5_g2_i1 | | 14.52 | 3.73 | 2.00 | 0.00 |
| TRINITY_DN90482_c3_g1_i1 | | 3.14 | 6.46 | -1.05 | 0.00 |
| TRINITY_DN90482_c5_g1_i13 | PLA2R | 0.34 | 11.09 | -4.95 | 0.00 |
| TRINITY_DN90484_c5_g2_i1 | | 0.20 | 1.36 | -2.82 | 0.00 |
| TRINITY_DN90506_c7_g1_i1 | | 0.98 | 7.48 | -2.85 | 0.00 |
| TRINITY_DN90507_c6_g2_i1 | GIMA7 | 3.44 | 8.51 | -1.30 | 0.00 |
| TRINITY_DN90507_c6_g3_i1 | | 4.42 | 19.04 | -2.07 | 0.00 |
| TRINITY_DN90527_c0_g3_i1 | GIMA4 | 4.07 | 11.71 | -1.56 | 0.00 |
| TRINITY_DN90527_c0_g4_i1 | GIMA5 | 4.09 | 19.41 | -2.29 | 0.00 |
| TRINITY_DN90539_c4_g1_i3 | | 11.60 | 30.48 | -1.39 | 0.00 |
| TRINITY_DN90544_c0_g1_i3 | | 0.76 | 0.05 | 4.08 | 0.00 |
| TRINITY_DN90546_c1_g1_i4 | | 39.97 | 111.29 | -1.51 | 0.00 |
| TRINITY_DN90569_c7_g1_i1 | | 3.13 | 5.73 | -0.90 | 0.00 |
| TRINITY_DN90576_c2_g2_i2 | | 0.60 | 10.75 | -4.15 | 0.00 |
| TRINITY_DN90579_c0_g1_i2 | LEG | 79.63 | 26.87 | 1.63 | 0.00 |
| TRINITY_DN90586_c0_g1_i1 | MYO1B | 3.83 | 1.84 | 1.05 | 0.00 |
| TRINITY_DN90597_c9_g2_i4 | | 3.93 | 34.03 | -3.28 | 0.00 |
| TRINITY_DN90610_c0_g2_i4 | PL8L1 | 3.03 | 9.57 | -1.68 | 0.00 |
| TRINITY_DN90621_c11_g2_i1 | | 0.57 | 4.56 | -2.95 | 0.00 |
| TRINITY_DN90624_c0_g1_i1 | PPDPF | 24.90 | 3.50 | 2.86 | 0.00 |

| | | | | | |
|---|---|---|---|---|---|
| TRINITY_DN90641_c1_g2_i3 | L1CAM | 0.25 | 1.14 | -2.20 | 0.00 |
| TRINITY_DN90654_c0_g2_i3 | MYL6 | 54.42 | 28.29 | 0.96 | 0.00 |
| TRINITY_DN90658_c1_g1_i3 | | 0.85 | 8.52 | -3.24 | 0.00 |
| TRINITY_DN90665_c6_g1_i2 | | 1.42 | 0.13 | 3.48 | 0.00 |
| TRINITY_DN90669_c4_g1_i4 | | 2.16 | 32.87 | -3.91 | 0.00 |
| TRINITY_DN90677_c3_g3_i1 | | 0.00 | 1.56 | -4.15 | 0.00 |
| TRINITY_DN90686_c1_g1_i5 | LYG | 7.09 | 21.05 | -1.62 | 0.00 |
| TRINITY_DN90696_c0_g4_i2 | | 0.33 | 2.16 | -2.71 | 0.00 |
| TRINITY_DN90697_c3_g1_i3 | | 0.13 | 1.60 | -3.36 | 0.00 |
| TRINITY_DN90700_c0_g5_i1 | | 46.76 | 84.28 | -0.88 | 0.00 |
| TRINITY_DN90707_c2_g1_i1 | HINT1 | 0.40 | 3.54 | -3.24 | 0.00 |
| TRINITY_DN90716_c1_g1_i13 | | 1.03 | 11.35 | -3.07 | 0.00 |
| TRINITY_DN90731_c8_g1_i1 | | 0.59 | 1.86 | -1.70 | 0.00 |
| TRINITY_DN90742_c1_g1_i6 | | 6.17 | 0.22 | 4.84 | 0.00 |
| TRINITY_DN90747_c11_g1_i2 | | 9.64 | 34.12 | -1.76 | 0.00 |
| TRINITY_DN90747_c12_g6_i1 | | 0.36 | 2.48 | -3.03 | 0.00 |
| TRINITY_DN90748_c4_g1_i1 | | 0.18 | 2.30 | -3.33 | 0.00 |
| TRINITY_DN90751_c10_g1_i1 | TVB2 | 0.26 | 2.52 | -3.47 | 0.00 |
| TRINITY_DN90751_c9_g1_i1 | | 4.51 | 20.81 | -2.23 | 0.00 |
| TRINITY_DN90755_c0_g3_i2 | | 0.08 | 0.99 | -3.67 | 0.00 |
| TRINITY_DN90755_c0_g3_i3 | | 2.68 | 0.72 | 1.90 | 0.00 |
| TRINITY_DN90755_c0_g7_i1 | | 0.35 | 3.83 | -3.49 | 0.00 |
| TRINITY_DN90759_c0_g2_i4 | DAAM1 | 0.00 | 1.46 | -6.86 | 0.00 |
| TRINITY_DN90771_c7_g1_i8 | | 0.67 | 0.00 | 4.41 | 0.00 |
| TRINITY_DN90772_c3_g1_i1 | | 3.95 | 346.90 | -6.33 | 0.00 |
| TRINITY_DN90772_c3_g1_i10 | | 1.70 | 239.55 | -6.87 | 0.00 |
| TRINITY_DN90772_c3_g1_i15 | | 7.55 | 443.35 | -5.80 | 0.00 |
| TRINITY_DN90772_c3_g1_i17 | | 0.22 | 12.52 | -5.44 | 0.00 |
| TRINITY_DN90772_c3_g1_i8 | | 0.67 | 56.53 | -5.82 | 0.00 |
| TRINITY_DN90773_c1_g1_i6 | GP171 | 0.93 | 5.73 | -2.66 | 0.00 |
| TRINITY_DN90776_c0_g2_i10 | NDUS2 | 4.35 | 12.74 | -1.58 | 0.00 |
| TRINITY_DN90787_c0_g6_i1 | | 11.66 | 4.55 | 1.34 | 0.00 |
| TRINITY_DN90795_c4_g1_i1 | | 0.30 | 9.46 | -3.68 | 0.00 |
| TRINITY_DN90806_c0_g1_i2 | LAP4A | 13.59 | 8.21 | 0.78 | 0.00 |
| TRINITY_DN90823_c9_g1_i1 | RTBS | 4.17 | 0.20 | 3.82 | 0.00 |
| TRINITY_DN90825_c1_g1_i4 | ACTN1 | 5.61 | 3.20 | 0.81 | 0.00 |
| TRINITY_DN90833_c0_g2_i5 | | 9.15 | 1.03 | 3.13 | 0.00 |
| TRINITY_DN90838_c2_g3_i1 | | 0.58 | 254.18 | -8.24 | 0.00 |
| TRINITY_DN90846_c3_g1_i6 | | 1.87 | 17.90 | -3.23 | 0.00 |
| TRINITY_DN90846_c3_g2_i1 | | 143.56 | 425.37 | -1.63 | 0.00 |
| TRINITY_DN90859_c11_g1_i4 | | 0.00 | 4.86 | -3.98 | 0.00 |
| TRINITY_DN90884_c9_g1_i1 | | 9.01 | 32.72 | -1.85 | 0.00 |

| | | | | | |
|---|---|---|---|---|---|
| TRINITY_DN90892_c2_g1_i3 | | 0.39 | 3.62 | -3.30 | 0.00 |
| TRINITY_DN90901_c6_g2_i5 | | 0.84 | 6.97 | -2.98 | 0.00 |
| TRINITY_DN90905_c6_g1_i1 | | 0.00 | 2.24 | -5.23 | 0.00 |
| TRINITY_DN90912_c1_g2_i9 | OTOAN | 0.14 | 3.37 | -4.62 | 0.00 |
| TRINITY_DN90922_c10_g1_i1 | GMFG | 5.52 | 13.24 | -1.23 | 0.00 |
| TRINITY_DN90922_c10_g1_i4 | GMFG | 16.94 | 37.66 | -1.13 | 0.00 |
| TRINITY_DN90940_c4_g2_i1 | | 18.47 | 6.17 | 1.53 | 0.00 |
| TRINITY_DN90944_c2_g1_i1 | HG2A | 0.73 | 3.36 | -2.22 | 0.00 |
| TRINITY_DN90944_c2_g1_i4 | HG2A | 163.45 | 431.25 | -1.36 | 0.00 |
| TRINITY_DN90976_c9_g1_i3 | | 0.05 | 0.92 | -4.17 | 0.00 |
| TRINITY_DN91001_c2_g2_i1 | R213A | 1.30 | 3.72 | -1.54 | 0.00 |
| TRINITY_DN91007_c0_g1_i1 | | 0.10 | 2.67 | -4.06 | 0.00 |
| TRINITY_DN91032_c1_g1_i7 | CATB | 83.01 | 135.57 | -0.75 | 0.00 |
| TRINITY_DN91057_c0_g2_i2 | | 3.33 | 0.93 | 1.86 | 0.00 |
| TRINITY_DN91078_c2_g2_i1 | | 17.87 | 1.83 | 3.21 | 0.00 |
| TRINITY_DN91099_c4_g2_i4 | KAP1 | 0.95 | 2.60 | -1.48 | 0.00 |
| TRINITY_DN91101_c10_g1_i5 | RIPK4 | 4.23 | 8.09 | -0.93 | 0.00 |
| TRINITY_DN91129_c0_g1_i3 | | 2.62 | 0.08 | 4.56 | 0.00 |
| TRINITY_DN91142_c0_g1_i2 | HVM16 | 0.31 | 14.91 | -5.46 | 0.00 |
| TRINITY_DN91142_c0_g1_i7 | HV02 | 0.00 | 5.16 | -5.11 | 0.00 |
| TRINITY_DN91142_c0_g8_i1 | HVM43 | 0.13 | 9.58 | -5.26 | 0.00 |
| TRINITY_DN91143_c2_g1_i1 | CCL4 | 4.16 | 9.95 | -1.33 | 0.00 |
| TRINITY_DN91144_c5_g4_i2 | | 0.47 | 3.34 | -2.82 | 0.00 |
| TRINITY_DN91148_c0_g1_i2 | OCSTP | 0.04 | 3.45 | -5.81 | 0.00 |
| TRINITY_DN91154_c4_g1_i1 | OSTF1 | 19.93 | 28.69 | -0.56 | 0.00 |
| TRINITY_DN91156_c1_g1_i4 | | 1.38 | 14.17 | -3.84 | 0.00 |
| TRINITY_DN91157_c0_g1_i2 | CAN2 | 5.98 | 14.06 | -1.26 | 0.00 |
| TRINITY_DN91158_c10_g1_i1 | | 0.87 | 4.45 | -2.32 | 0.00 |
| TRINITY_DN91164_c6_g1_i1 | | 3.58 | 9.73 | -1.46 | 0.00 |
| TRINITY_DN91168_c0_g1_i7 | | 0.53 | 6.05 | -3.58 | 0.00 |
| TRINITY_DN91168_c0_g2_i3 | | 23.56 | 69.02 | -1.59 | 0.00 |
| TRINITY_DN91183_c3_g1_i2 | | 2.59 | 0.00 | 5.34 | 0.00 |
| TRINITY_DN91190_c5_g1_i3 | | 1.31 | 4.96 | -2.01 | 0.00 |
| TRINITY_DN91190_c6_g1_i2 | SOX4 | 0.53 | 1.50 | -1.51 | 0.00 |
| TRINITY_DN91193_c4_g2_i2 | | 0.08 | 2.94 | -4.89 | 0.00 |
| TRINITY_DN91245_c0_g1_i1 | | 7.24 | 2.00 | 1.88 | 0.00 |
| TRINITY_DN91257_c1_g1_i1 | | 3.29 | 0.96 | 1.72 | 0.00 |
| TRINITY_DN91261_c3_g1_i1 | | 0.70 | 9.90 | -3.70 | 0.00 |
| TRINITY_DN91261_c3_g2_i1 | | 0.09 | 2.87 | -3.99 | 0.00 |
| TRINITY_DN91261_c3_g2_i2 | | 10.23 | 254.48 | -4.54 | 0.00 |
| TRINITY_DN91263_c8_g1_i1 | | 3.26 | 12.94 | -1.97 | 0.00 |
| TRINITY_DN91269_c0_g1_i4 | | 1.63 | 0.16 | 3.56 | 0.00 |

| | | | | | |
|---|---|---|---|---|---|
| TRINITY_DN91286_c0_g1_i14 | NED4L | 0.60 | 0.00 | 6.03 | 0.00 |
| TRINITY_DN91287_c10_g1_i1 | | 1632.39 | 540.81 | 1.61 | 0.00 |
| TRINITY_DN91302_c5_g2_i5 | FYB | 2.19 | 8.49 | -1.98 | 0.00 |
| TRINITY_DN91304_c0_g2_i2 | ARHGC | 3.42 | 20.52 | -2.62 | 0.00 |
| TRINITY_DN91308_c1_g5_i1 | | 19.28 | 4.65 | 2.06 | 0.00 |
| TRINITY_DN91316_c0_g1_i1 | CXCR4 | 7.64 | 18.42 | -1.29 | 0.00 |
| TRINITY_DN91333_c7_g1_i1 | | 0.26 | 2.58 | -3.67 | 0.00 |
| TRINITY_DN91338_c1_g1_i14 | ION3 | 443.48 | 146.10 | 1.65 | 0.00 |
| TRINITY_DN91339_c5_g2_i1 | CLDY | 42.87 | 89.86 | -1.10 | 0.00 |
| TRINITY_DN91339_c6_g2_i1 | CLD4 | 1.04 | 26.01 | -4.48 | 0.00 |
| TRINITY_DN91339_c6_g3_i1 | CLDY | 66.04 | 126.62 | -1.00 | 0.00 |
| TRINITY_DN91340_c3_g3_i1 | | 1.45 | 12.51 | -3.38 | 0.00 |
| TRINITY_DN91341_c0_g1_i3 | ADCY4 | 0.92 | 4.70 | -2.41 | 0.00 |
| TRINITY_DN91341_c1_g1_i2 | | 4.82 | 12.67 | -1.38 | 0.00 |
| TRINITY_DN91342_c0_g8_i3 | TPM4 | 12.27 | 6.18 | 0.96 | 0.00 |
| TRINITY_DN91347_c1_g1_i2 | GRAK | 2.67 | 9.56 | -1.88 | 0.00 |
| TRINITY_DN91347_c2_g1_i3 | | 2.27 | 9.33 | -2.06 | 0.00 |
| TRINITY_DN91347_c3_g3_i2 | | 73.80 | 28.89 | 1.35 | 0.00 |
| TRINITY_DN91356_c2_g7_i1 | | 5.19 | 2.62 | 0.98 | 0.00 |
| TRINITY_DN91364_c13_g1_i1 | | 9.39 | 24.63 | -1.44 | 0.00 |
| TRINITY_DN91364_c13_g2_i4 | MPEG1 | 0.00 | 28.35 | -25.52 | 0.00 |
| TRINITY_DN91370_c2_g2_i2 | IL17F | 0.17 | 5.22 | -4.98 | 0.00 |
| TRINITY_DN91380_c4_g1_i1 | | 0.79 | 6.57 | -3.11 | 0.00 |
| TRINITY_DN91388_c0_g1_i1 | | 4.30 | 0.25 | 4.06 | 0.00 |
| TRINITY_DN91388_c0_g1_i3 | | 0.00 | 3.20 | -6.51 | 0.00 |
| TRINITY_DN91401_c0_g3_i1 | FIBIN | 8.01 | 4.20 | 0.93 | 0.00 |
| TRINITY_DN91412_c5_g1_i1 | | 0.20 | 5.95 | -5.02 | 0.00 |
| TRINITY_DN91423_c3_g2_i2 | FLOT2 | 0.00 | 1.90 | -5.19 | 0.00 |
| TRINITY_DN91436_c0_g1_i3 | | 4.13 | 22.88 | -2.43 | 0.00 |
| TRINITY_DN91441_c6_g1_i1 | | 0.13 | 1.59 | -3.62 | 0.00 |
| TRINITY_DN91455_c3_g1_i5 | DESP | 8.91 | 3.28 | 1.41 | 0.00 |
| TRINITY_DN91460_c0_g1_i2 | S38A3 | 0.16 | 1.41 | -3.08 | 0.00 |
| TRINITY_DN91478_c6_g3_i1 | | 0.08 | 3.75 | -4.19 | 0.00 |
| TRINITY_DN91481_c12_g3_i1 | | 10.05 | 44.34 | -2.08 | 0.00 |
| TRINITY_DN91501_c4_g6_i2 | CAZA2 | 3.23 | 0.15 | 4.92 | 0.00 |
| TRINITY_DN91516_c3_g1_i3 | AP1S3 | 2.85 | 8.94 | -1.69 | 0.00 |
| TRINITY_DN91517_c1_g1_i1 | GILT | 0.65 | 5.77 | -4.15 | 0.00 |
| TRINITY_DN91517_c1_g1_i2 | GILT | 44.75 | 183.88 | -2.02 | 0.00 |
| TRINITY_DN91517_c1_g2_i2 | GILT | 17.46 | 39.47 | -1.14 | 0.00 |
| TRINITY_DN91526_c3_g2_i11 | DOCK2 | 4.17 | 9.82 | -1.23 | 0.00 |
| TRINITY_DN91526_c3_g2_i15 | DOCK2 | 1.01 | 4.46 | -2.15 | 0.00 |
| TRINITY_DN91526_c3_g2_i4 | DOCK2 | 0.23 | 0.96 | -2.11 | 0.00 |

| | | | | | |
|---|---|---|---|---|---|
| TRINITY_DN91528_c3_g1_i1 | TMLH | 0.00 | 1.11 | -5.82 | 0.00 |
| TRINITY_DN91549_c4_g1_i1 | | 2.39 | 0.00 | 4.99 | 0.00 |
| TRINITY_DN91549_c5_g1_i3 | | 2.81 | 0.30 | 2.93 | 0.00 |
| TRINITY_DN91561_c4_g1_i1 | CCR6 | 0.33 | 3.33 | -3.50 | 0.00 |
| TRINITY_DN91561_c4_g1_i2 | CCR6 | 0.01 | 1.77 | -5.29 | 0.00 |
| TRINITY_DN91561_c4_g1_i4 | CCR6 | 0.57 | 7.00 | -3.55 | 0.00 |
| TRINITY_DN91578_c6_g1_i2 | | 0.13 | 1.10 | -3.07 | 0.00 |
| TRINITY_DN91582_c0_g1_i1 | | 1.80 | 35.76 | -4.44 | 0.00 |
| TRINITY_DN91582_c8_g2_i1 | | 1.57 | 50.20 | -4.98 | 0.00 |
| TRINITY_DN91582_c8_g2_i2 | | 1.98 | 27.39 | -3.93 | 0.00 |
| TRINITY_DN91582_c8_g6_i1 | | 2.43 | 45.94 | -4.24 | 0.00 |
| TRINITY_DN91585_c3_g3_i7 | XBP1 | 23.98 | 13.23 | 0.91 | 0.00 |
| TRINITY_DN91593_c0_g3_i3 | K1C13 | 8.42 | 1.56 | 2.48 | 0.00 |
| TRINITY_DN91595_c1_g3_i1 | CD248 | 2.06 | 0.95 | 1.08 | 0.00 |
| TRINITY_DN91601_c8_g5_i1 | | 1.14 | 7.09 | -2.63 | 0.00 |
| TRINITY_DN91616_c2_g1_i4 | | 0.35 | 1.96 | -2.58 | 0.00 |
| TRINITY_DN91618_c1_g3_i1 | | 1.11 | 2.79 | -1.32 | 0.00 |
| TRINITY_DN91626_c0_g1_i3 | PMEL | 1.36 | 0.10 | 4.03 | 0.00 |
| TRINITY_DN91636_c1_g3_i1 | | 10.27 | 0.52 | 3.53 | 0.00 |
| TRINITY_DN91637_c1_g1_i6 | SBP2L | 0.00 | 1.09 | -6.44 | 0.00 |
| TRINITY_DN91637_c1_g4_i1 | | 4.11 | 22.51 | -2.46 | 0.00 |
| TRINITY_DN91639_c1_g1_i1 | DGKD | 0.21 | 3.97 | -4.24 | 0.00 |
| TRINITY_DN91652_c2_g2_i1 | | 50.10 | 22.30 | 1.27 | 0.00 |
| TRINITY_DN91694_c3_g1_i3 | F13A | 11.46 | 3.05 | 1.90 | 0.00 |
| TRINITY_DN91703_c13_g2_i2 | ENTP2 | 2.31 | 4.90 | -1.12 | 0.00 |
| TRINITY_DN91705_c1_g1_i9 | | 153.98 | 251.19 | -0.72 | 0.00 |
| TRINITY_DN91712_c13_g1_i1 | | 6.87 | 21.98 | -1.67 | 0.00 |
| TRINITY_DN91712_c5_g1_i1 | | 94.27 | 39.48 | 1.24 | 0.00 |
| TRINITY_DN91718_c0_g4_i2 | | 2.25 | 0.55 | 2.03 | 0.00 |
| TRINITY_DN91743_c3_g2_i1 | CML1 | 0.20 | 0.84 | -2.13 | 0.00 |
| TRINITY_DN91743_c3_g3_i1 | | 0.04 | 1.02 | -3.61 | 0.00 |
| TRINITY_DN91760_c3_g2_i3 | | 160.74 | 84.55 | 0.93 | 0.00 |
| TRINITY_DN91773_c0_g1_i3 | | 0.00 | 4.59 | -6.89 | 0.00 |
| TRINITY_DN91788_c3_g4_i1 | IL2RG | 9.28 | 29.57 | -1.70 | 0.00 |
| TRINITY_DN91791_c13_g1_i1 | HINT3 | 0.33 | 2.13 | -2.52 | 0.00 |
| TRINITY_DN91793_c8_g1_i1 | ENDUC | 9.57 | 20.81 | -1.16 | 0.00 |
| TRINITY_DN91794_c5_g1_i2 | VG87 | 41.57 | 64.55 | -0.67 | 0.00 |
| TRINITY_DN91795_c0_g1_i2 | M3K8 | 0.69 | 3.41 | -2.36 | 0.00 |
| TRINITY_DN91796_c2_g1_i6 | TGFI1 | 5.03 | 2.45 | 1.01 | 0.00 |
| TRINITY_DN91806_c6_g3_i2 | GRT1A | 0.72 | 2.88 | -1.98 | 0.00 |
| TRINITY_DN91811_c1_g2_i8 | TRI25 | 0.00 | 2.38 | -6.44 | 0.00 |
| TRINITY_DN91812_c7_g1_i1 | | 0.57 | 4.33 | -3.03 | 0.00 |

| | | | | | |
|---|---|---|---|---|---|
| TRINITY_DN91815_c1_g3_i3 | | 1.53 | 3.23 | -1.04 | 0.00 |
| TRINITY_DN91824_c3_g2_i2 | GMPPB | 7.27 | 0.49 | 3.76 | 0.00 |
| TRINITY_DN91824_c5_g3_i1 | | 54.76 | 29.17 | 0.90 | 0.00 |
| TRINITY_DN91824_c5_g4_i1 | | 6.70 | 22.13 | -1.74 | 0.00 |
| TRINITY_DN91829_c7_g2_i1 | | 3.76 | 8.56 | -1.17 | 0.00 |
| TRINITY_DN91845_c0_g1_i4 | CACO2 | 0.00 | 1.19 | -5.40 | 0.00 |
| TRINITY_DN91845_c0_g4_i1 | | 0.60 | 5.18 | -3.19 | 0.00 |
| TRINITY_DN91851_c9_g1_i2 | | 0.68 | 3.42 | -2.38 | 0.00 |
| TRINITY_DN91853_c0_g1_i3 | IRF4 | 0.11 | 0.92 | -3.24 | 0.00 |
| TRINITY_DN91853_c0_g1_i6 | IRF4 | 0.00 | 0.38 | -4.19 | 0.00 |
| TRINITY_DN91874_c0_g1_i2 | SPAT2 | 2.14 | 4.85 | -1.16 | 0.00 |
| TRINITY_DN91878_c1_g1_i3 | TNNI1 | 2.01 | 8.92 | -2.19 | 0.00 |
| TRINITY_DN91887_c6_g1_i6 | | 0.08 | 2.70 | -4.58 | 0.00 |
| TRINITY_DN91893_c3_g1_i10 | RGS1 | 0.00 | 1.19 | -5.64 | 0.00 |
| TRINITY_DN91893_c3_g1_i11 | RGS1 | 1.90 | 12.89 | -2.77 | 0.00 |
| TRINITY_DN91893_c3_g1_i13 | RGS21 | 1.57 | 12.08 | -3.01 | 0.00 |
| TRINITY_DN91893_c3_g1_i14 | RGS5 | 0.47 | 3.53 | -2.93 | 0.00 |
| TRINITY_DN91893_c3_g1_i2 | RGS21 | 0.00 | 1.05 | -5.37 | 0.00 |
| TRINITY_DN91893_c3_g1_i3 | RGS1 | 0.00 | 1.55 | -5.69 | 0.00 |
| TRINITY_DN91893_c3_g1_i4 | RGS2 | 4.79 | 24.25 | -2.29 | 0.00 |
| TRINITY_DN91893_c3_g1_i6 | RGS2 | 16.17 | 88.93 | -2.50 | 0.00 |
| TRINITY_DN91897_c2_g1_i1 | | 8.11 | 21.21 | -1.35 | 0.00 |
| TRINITY_DN91922_c0_g4_i1 | | 0.23 | 3.59 | -3.93 | 0.00 |
| TRINITY_DN91922_c0_g4_i4 | AGRG1 | 0.96 | 8.93 | -3.29 | 0.00 |
| TRINITY_DN91922_c0_g4_i5 | | 0.06 | 2.76 | -5.17 | 0.00 |
| TRINITY_DN91932_c4_g1_i7 | DLRB2 | 0.37 | 0.00 | 4.48 | 0.00 |
| TRINITY_DN91940_c4_g2_i7 | ISK1 | 3.77 | 14.51 | -1.96 | 0.00 |
| TRINITY_DN91960_c5_g1_i3 | | 2.70 | 9.50 | -1.83 | 0.00 |
| TRINITY_DN91964_c0_g1_i9 | CCR7 | 2.96 | 8.72 | -1.64 | 0.00 |
| TRINITY_DN91991_c4_g1_i2 | | 19.66 | 6.52 | 1.82 | 0.00 |
| TRINITY_DN91992_c10_g1_i1 | | 1.37 | 7.40 | -2.39 | 0.00 |
| TRINITY_DN92008_c1_g1_i3 | NRK2 | 47.29 | 22.48 | 1.08 | 0.00 |
| TRINITY_DN92008_c1_g1_i6 | NRK2 | 222.83 | 154.26 | 0.57 | 0.00 |
| TRINITY_DN92014_c4_g1_i1 | | 0.35 | 2.84 | -2.96 | 0.00 |
| TRINITY_DN92018_c2_g2_i5 | TMM54 | 0.00 | 9.31 | -23.24 | 0.00 |
| TRINITY_DN92024_c1_g1_i9 | MCF2L | 0.19 | 1.78 | -3.23 | 0.00 |
| TRINITY_DN92026_c2_g2_i9 | PARP9 | 3.69 | 7.82 | -1.09 | 0.00 |
| TRINITY_DN92031_c1_g1_i1 | | 0.16 | 1.63 | -3.40 | 0.00 |
| TRINITY_DN92034_c10_g1_i5 | | 1.80 | 6.83 | -1.94 | 0.00 |
| TRINITY_DN92045_c2_g1_i1 | TT21B | 3.20 | 0.63 | 2.45 | 0.00 |
| TRINITY_DN92046_c2_g1_i6 | MRC2 | 0.26 | 9.11 | -5.17 | 0.00 |
| TRINITY_DN92046_c2_g1_i8 | LYAM1 | 0.00 | 1.22 | -4.81 | 0.00 |

| | | | | | |
|---|---|---|---|---|---|
| TRINITY_DN92065_c7_g1_i3 | CNFNB | 0.00 | 3.33 | -6.39 | 0.00 |
| TRINITY_DN92086_c4_g1_i2 | | 0.04 | 4.80 | -5.98 | 0.00 |
| TRINITY_DN92087_c1_g1_i15 | RHOF | 1.44 | 5.27 | -1.92 | 0.00 |
| TRINITY_DN92089_c9_g1_i1 | ROA2 | 0.70 | 18.02 | -4.57 | 0.00 |
| TRINITY_DN92120_c7_g1_i1 | ABCF2 | 0.49 | 1.53 | -1.67 | 0.00 |
| TRINITY_DN92124_c2_g1_i4 | | 0.34 | 8.67 | -4.78 | 0.00 |
| TRINITY_DN92134_c7_g1_i1 | | 0.59 | 2.42 | -2.07 | 0.00 |
| TRINITY_DN92138_c0_g2_i1 | | 30.98 | 1.50 | 4.16 | 0.00 |
| TRINITY_DN92140_c0_g1_i3 | IF44L | 9.59 | 17.47 | -0.87 | 0.00 |
| TRINITY_DN92170_c6_g1_i5 | DI2BA | 0.00 | 1.25 | -4.44 | 0.00 |
| TRINITY_DN92193_c10_g2_i1 | | 17.74 | 79.49 | -2.23 | 0.00 |
| TRINITY_DN92193_c10_g2_i2 | | 6.07 | 22.99 | -2.00 | 0.00 |
| TRINITY_DN92199_c2_g1_i1 | | 3.36 | 20.93 | -2.81 | 0.00 |
| TRINITY_DN92215_c2_g1_i1 | PAR14 | 9.38 | 23.30 | -1.34 | 0.00 |
| TRINITY_DN92220_c4_g1_i1 | | 1.66 | 5.49 | -1.80 | 0.00 |
| TRINITY_DN92227_c2_g1_i1 | | 1.72 | 4.91 | -1.53 | 0.00 |
| TRINITY_DN92227_c3_g3_i2 | TR11B | 0.17 | 4.29 | -4.64 | 0.00 |
| TRINITY_DN92241_c11_g1_i9 | | 0.13 | 2.20 | -4.10 | 0.00 |
| TRINITY_DN92254_c0_g1_i8 | IKBA | 39.31 | 79.10 | -0.99 | 0.00 |
| TRINITY_DN92258_c0_g1_i3 | HMR1 | 0.02 | 1.38 | -4.79 | 0.00 |
| TRINITY_DN92279_c8_g1_i1 | | 8.33 | 31.11 | -1.99 | 0.00 |
| TRINITY_DN92280_c0_g1_i2 | BRYP | 273.71 | 166.32 | 0.74 | 0.00 |
| TRINITY_DN92280_c0_g3_i1 | | 16.17 | 0.00 | 5.39 | 0.00 |
| TRINITY_DN92290_c3_g3_i7 | SCAM1 | 1.24 | 4.75 | -1.92 | 0.00 |
| TRINITY_DN92291_c4_g1_i1 | CNMD | 6.34 | 1.83 | 1.79 | 0.00 |
| TRINITY_DN92292_c5_g1_i1 | A33 | 1.51 | 4.56 | -1.57 | 0.00 |
| TRINITY_DN92299_c4_g1_i2 | | 2.74 | 12.33 | -2.17 | 0.00 |
| TRINITY_DN92317_c1_g2_i1 | | 27.81 | 111.99 | -2.01 | 0.00 |
| TRINITY_DN92321_c0_g1_i5 | IKBE | 0.32 | 2.10 | -2.72 | 0.00 |
| TRINITY_DN92321_c0_g1_i7 | IKBE | 1.83 | 7.74 | -2.09 | 0.00 |
| TRINITY_DN92328_c5_g2_i4 | SAP | 34.11 | 57.20 | -0.77 | 0.00 |
| TRINITY_DN92334_c2_g1_i4 | EFC4B | 0.84 | 2.53 | -1.57 | 0.00 |
| TRINITY_DN92338_c0_g1_i3 | TBCC1 | 0.78 | 0.05 | 3.98 | 0.00 |
| TRINITY_DN92367_c0_g1_i7 | PLOD1 | 1.03 | 0.17 | 2.56 | 0.00 |
| TRINITY_DN92379_c0_g1_i5 | JAK1 | 0.02 | 1.36 | -5.50 | 0.00 |
| TRINITY_DN92381_c0_g1_i12 | | 0.42 | 8.31 | -3.58 | 0.00 |
| TRINITY_DN92381_c0_g1_i14 | CCL13 | 1.86 | 12.19 | -2.72 | 0.00 |
| TRINITY_DN92381_c0_g1_i2 | CCL13 | 19.28 | 0.83 | 4.59 | 0.00 |
| TRINITY_DN92381_c0_g1_i4 | | 8.74 | 67.93 | -2.93 | 0.00 |
| TRINITY_DN92384_c6_g1_i3 | DOK1 | 4.63 | 8.20 | -0.85 | 0.00 |
| TRINITY_DN92395_c3_g1_i10 | SASH3 | 4.07 | 12.32 | -1.63 | 0.00 |
| TRINITY_DN92395_c3_g1_i4 | SASH3 | 5.91 | 17.88 | -1.65 | 0.00 |

| | | | | | |
|---|---|---|---|---|---|
| TRINITY_DN92406_c3_g1_i1 | CCR4 | 0.07 | 0.47 | -2.81 | 0.00 |
| TRINITY_DN92407_c5_g1_i4 | FA49B | 11.22 | 24.11 | -1.12 | 0.00 |
| TRINITY_DN92412_c2_g2_i1 | HA1K | 0.68 | 457.76 | -9.48 | 0.00 |
| TRINITY_DN92412_c2_g3_i4 | HMR1 | 0.11 | 137.44 | -8.21 | 0.00 |
| TRINITY_DN92412_c4_g1_i1 | HMR1 | 1.67 | 54.60 | -5.17 | 0.00 |
| TRINITY_DN92419_c7_g1_i4 | RAB10 | 0.00 | 2.60 | -6.51 | 0.00 |
| TRINITY_DN92429_c0_g1_i1 | MPDZ | 3.47 | 0.23 | 3.37 | 0.00 |
| TRINITY_DN92429_c3_g6_i1 | | 1.03 | 8.70 | -3.05 | 0.00 |
| TRINITY_DN92443_c0_g2_i2 | MCRI1 | 9.00 | 15.07 | -0.77 | 0.00 |
| TRINITY_DN92485_c7_g6_i1 | | 2.57 | 0.52 | 2.25 | 0.00 |
| TRINITY_DN92523_c4_g2_i16 | NKL | 0.80 | 5.39 | -2.80 | 0.00 |
| TRINITY_DN92527_c3_g2_i1 | RHDF1 | 0.49 | 1.35 | -1.45 | 0.00 |
| TRINITY_DN92528_c1_g2_i2 | SEPT9 | 0.52 | 2.09 | -1.95 | 0.00 |
| TRINITY_DN92528_c1_g2_i5 | SEPT9 | 0.70 | 6.59 | -3.17 | 0.00 |
| TRINITY_DN92535_c4_g2_i1 | | 2.50 | 11.26 | -2.15 | 0.00 |
| TRINITY_DN92535_c4_g2_i2 | | 0.02 | 3.42 | -6.17 | 0.00 |
| TRINITY_DN92561_c4_g2_i7 | | 0.21 | 2.66 | -3.55 | 0.00 |
| TRINITY_DN92562_c0_g4_i2 | RAB25 | 0.27 | 1.74 | -2.90 | 0.00 |
| TRINITY_DN92562_c0_g4_i4 | RAB25 | 0.00 | 16.79 | -9.29 | 0.00 |
| TRINITY_DN92565_c6_g2_i1 | | 2.10 | 4.51 | -1.13 | 0.00 |
| TRINITY_DN92583_c5_g3_i2 | | 1.04 | 9.13 | -3.22 | 0.00 |
| TRINITY_DN92585_c8_g4_i5 | | 0.00 | 2.74 | -4.51 | 0.00 |
| TRINITY_DN92586_c5_g3_i1 | IF44L | 6.20 | 32.77 | -2.40 | 0.00 |
| TRINITY_DN92587_c6_g1_i1 | | 0.17 | 1.57 | -3.11 | 0.00 |
| TRINITY_DN92605_c1_g1_i2 | | 0.18 | 6.49 | -4.38 | 0.00 |
| TRINITY_DN92605_c1_g1_i5 | | 3.86 | 11.88 | -1.57 | 0.00 |
| TRINITY_DN92605_c1_g2_i2 | CDHR1 | 5.73 | 0.39 | 3.98 | 0.00 |
| TRINITY_DN92609_c0_g1_i13 | COF2 | 185.77 | 329.04 | -0.86 | 0.00 |
| TRINITY_DN92625_c8_g3_i1 | | 8.66 | 23.99 | -1.52 | 0.00 |
| TRINITY_DN92634_c5_g3_i2 | | 12.64 | 0.55 | 4.36 | 0.00 |
| TRINITY_DN92653_c8_g2_i1 | TLR13 | 0.11 | 1.15 | -3.53 | 0.00 |
| TRINITY_DN92679_c4_g3_i1 | | 0.15 | 1.31 | -3.15 | 0.00 |
| TRINITY_DN92687_c6_g1_i4 | | 3.20 | 0.00 | 5.81 | 0.00 |
| TRINITY_DN92702_c3_g4_i2 | PL8L1 | 14.93 | 32.79 | -1.13 | 0.00 |
| TRINITY_DN92709_c4_g1_i1 | | 0.60 | 2.49 | -2.14 | 0.00 |
| TRINITY_DN92722_c1_g1_i1 | | 17.87 | 10.33 | 0.77 | 0.00 |
| TRINITY_DN92726_c0_g3_i3 | | 4.94 | 2.36 | 1.08 | 0.00 |
| TRINITY_DN92733_c2_g2_i1 | | 34.25 | 78.32 | -1.17 | 0.00 |
| TRINITY_DN92772_c0_g1_i5 | BCAR3 | 3.16 | 0.09 | 4.90 | 0.00 |
| TRINITY_DN92781_c5_g2_i2 | | 0.07 | 0.83 | -3.58 | 0.00 |
| TRINITY_DN92794_c4_g3_i1 | MCT1A | 1.78 | 8.15 | -2.25 | 0.00 |
| TRINITY_DN92805_c9_g1_i8 | GSHB | 6.44 | 2.61 | 1.33 | 0.00 |

| | | | | | |
|---|---|---|---|---|---|
| TRINITY_DN92813_c0_g2_i4 | AGO1 | 0.91 | 0.32 | 1.49 | 0.00 |
| TRINITY_DN92816_c4_g2_i2 | HEBP2 | 47.64 | 22.40 | 1.11 | 0.00 |
| TRINITY_DN92818_c6_g6_i1 | | 0.00 | 25.16 | -7.11 | 0.00 |
| TRINITY_DN92820_c1_g1_i3 | AIF1 | 36.69 | 105.60 | -1.55 | 0.00 |
| TRINITY_DN92820_c1_g3_i1 | | 0.78 | 4.35 | -2.43 | 0.00 |
| TRINITY_DN92845_c7_g2_i2 | AL5AP | 9.12 | 21.73 | -1.26 | 0.00 |
| TRINITY_DN92864_c8_g4_i2 | | 0.00 | 3.06 | -4.15 | 0.00 |
| TRINITY_DN92875_c5_g4_i1 | | 0.13 | 2.99 | -4.33 | 0.00 |
| TRINITY_DN92885_c1_g1_i11 | RRP8 | 0.00 | 0.91 | -5.27 | 0.00 |
| TRINITY_DN92887_c1_g1_i6 | CDS2 | 0.02 | 0.89 | -4.47 | 0.00 |
| TRINITY_DN92893_c3_g1_i1 | TB10C | 1.31 | 5.28 | -1.93 | 0.00 |
| TRINITY_DN92894_c3_g1_i1 | GNA14 | 0.77 | 6.68 | -2.92 | 0.00 |
| TRINITY_DN92894_c3_g2_i1 | GNA14 | 1.39 | 6.20 | -2.15 | 0.00 |
| TRINITY_DN92894_c3_g2_i2 | GNA14 | 1.81 | 13.31 | -2.86 | 0.00 |
| TRINITY_DN92938_c0_g1_i5 | CRTAM | 0.10 | 2.56 | -4.57 | 0.00 |
| TRINITY_DN92938_c0_g1_i9 | CRTAM | 0.49 | 3.09 | -2.66 | 0.00 |
| TRINITY_DN92940_c8_g1_i2 | RPA12 | 6.14 | 1.53 | 2.07 | 0.00 |
| TRINITY_DN92946_c5_g2_i2 | | 44.56 | 108.81 | -1.28 | 0.00 |
| TRINITY_DN92951_c0_g1_i1 | IKZF3 | 0.23 | 1.65 | -2.92 | 0.00 |
| TRINITY_DN92951_c0_g1_i3 | IKZF3 | 1.44 | 6.40 | -2.16 | 0.00 |
| TRINITY_DN92951_c0_g6_i2 | | 0.61 | 5.54 | -3.13 | 0.00 |
| TRINITY_DN92966_c3_g3_i1 | NETR | 0.17 | 1.35 | -3.00 | 0.00 |
| TRINITY_DN92972_c1_g2_i2 | GELS | 8.53 | 4.32 | 0.99 | 0.00 |
| TRINITY_DN92978_c9_g1_i2 | | 0.41 | 2.61 | -2.56 | 0.00 |
| TRINITY_DN92983_c6_g2_i1 | | 2.41 | 9.80 | -2.11 | 0.00 |
| TRINITY_DN92987_c5_g1_i3 | | 20.86 | 81.74 | -1.93 | 0.00 |
| TRINITY_DN92987_c5_g1_i6 | | 6.41 | 40.64 | -2.65 | 0.00 |
| TRINITY_DN92987_c5_g2_i3 | | 3.58 | 9.17 | -1.36 | 0.00 |
| TRINITY_DN92988_c10_g1_i2 | | 8.41 | 0.41 | 3.87 | 0.00 |
| TRINITY_DN92992_c5_g1_i8 | ERAP2 | 0.57 | 1.76 | -1.57 | 0.00 |
| TRINITY_DN92996_c2_g1_i1 | CD226 | 0.88 | 3.47 | -1.99 | 0.00 |
| TRINITY_DN93002_c1_g2_i1 | CCL20 | 22.05 | 96.74 | -2.08 | 0.00 |
| TRINITY_DN93002_c1_g2_i6 | CCL20 | 0.00 | 21.41 | -7.68 | 0.00 |
| TRINITY_DN93017_c9_g1_i1 | SIAT2 | 0.32 | 1.78 | -2.50 | 0.00 |
| TRINITY_DN93021_c6_g1_i1 | NLRC3 | 0.36 | 2.24 | -2.71 | 0.00 |
| TRINITY_DN93025_c4_g2_i5 | | 0.00 | 2.63 | -5.97 | 0.00 |
| TRINITY_DN93027_c8_g1_i1 | ASGR2 | 0.79 | 22.86 | -4.81 | 0.00 |
| TRINITY_DN93028_c0_g1_i3 | BROX | 3.65 | 0.37 | 3.24 | 0.00 |
| TRINITY_DN93031_c4_g1_i10 | | 1.34 | 15.00 | -3.17 | 0.00 |
| TRINITY_DN93031_c4_g1_i14 | | 0.31 | 3.70 | -3.03 | 0.00 |
| TRINITY_DN93031_c4_g2_i2 | | 2.59 | 31.26 | -3.51 | 0.00 |
| TRINITY_DN93034_c1_g1_i3 | | 0.38 | 2.60 | -2.89 | 0.00 |

| | | | | | |
|---|---|---|---|---|---|
| TRINITY_DN93040_c5_g3_i2 | ANTR1 | 2.09 | 0.02 | 5.87 | 0.00 |
| TRINITY_DN93060_c1_g1_i1 | | 0.59 | 14.54 | -3.84 | 0.00 |
| TRINITY_DN93097_c4_g2_i2 | TTY2L | 0.24 | 1.14 | -2.28 | 0.00 |
| TRINITY_DN93100_c4_g1_i1 | | 1.89 | 20.25 | -3.31 | 0.00 |
| TRINITY_DN93100_c8_g1_i2 | | 54.33 | 142.59 | -1.30 | 0.00 |
| TRINITY_DN93100_c8_g1_i3 | | 0.40 | 16.71 | -5.54 | 0.00 |
| TRINITY_DN93105_c6_g1_i1 | TC1A | 5.88 | 39.92 | -2.74 | 0.00 |
| TRINITY_DN93125_c4_g2_i2 | | 0.00 | 63.70 | -24.65 | 0.00 |
| TRINITY_DN93127_c2_g1_i9 | | 0.00 | 2.08 | -4.70 | 0.00 |
| TRINITY_DN93135_c2_g5_i2 | | 0.00 | 3.14 | -6.16 | 0.00 |
| TRINITY_DN93138_c4_g1_i3 | CCL4 | 2.73 | 8.07 | -1.57 | 0.00 |
| TRINITY_DN93138_c7_g1_i7 | HOIL1 | 2.27 | 4.82 | -1.08 | 0.00 |
| TRINITY_DN93141_c9_g1_i2 | | 0.80 | 4.84 | -2.58 | 0.00 |
| TRINITY_DN93149_c0_g2_i1 | HOX71 | 6.39 | 3.72 | 0.76 | 0.00 |
| TRINITY_DN93159_c2_g3_i1 | NECT2 | 4.39 | 17.23 | -1.95 | 0.00 |
| TRINITY_DN93173_c0_g1_i8 | | 0.30 | 2.83 | -3.33 | 0.00 |
| TRINITY_DN93176_c1_g1_i1 | | 2.49 | 8.17 | -1.72 | 0.00 |
| TRINITY_DN93176_c4_g1_i1 | | 8.06 | 1.58 | 2.24 | 0.00 |
| TRINITY_DN93179_c3_g1_i2 | | 7.40 | 4.38 | 0.72 | 0.00 |
| TRINITY_DN93180_c7_g1_i1 | | 2.66 | 83.71 | -4.97 | 0.00 |
| TRINITY_DN93182_c5_g6_i1 | | 0.87 | 2.11 | -1.34 | 0.00 |
| TRINITY_DN93205_c1_g2_i7 | USTA | 11.32 | 0.59 | 3.80 | 0.00 |
| TRINITY_DN93210_c8_g1_i3 | TC1A | 0.82 | 5.32 | -2.82 | 0.00 |
| TRINITY_DN93217_c9_g1_i1 | | 1.70 | 10.44 | -2.63 | 0.00 |
| TRINITY_DN93223_c2_g1_i1 | FLNA | 48.31 | 29.31 | 0.72 | 0.00 |
| TRINITY_DN93223_c2_g1_i8 | FLNA | 5.41 | 1.93 | 1.47 | 0.00 |
| TRINITY_DN93236_c6_g2_i1 | | 2.30 | 0.76 | 1.57 | 0.00 |
| TRINITY_DN93236_c7_g1_i5 | MOG1 | 0.03 | 0.95 | -3.74 | 0.00 |
| TRINITY_DN93258_c2_g3_i8 | | 0.17 | 3.19 | -4.29 | 0.00 |
| TRINITY_DN93258_c2_g3_i9 | PPHLN | 5.06 | 10.05 | -1.01 | 0.00 |
| TRINITY_DN93273_c0_g1_i10 | IRF1 | 23.99 | 66.53 | -1.44 | 0.00 |
| TRINITY_DN93273_c0_g1_i14 | IRF1 | 10.31 | 19.32 | -0.91 | 0.00 |
| TRINITY_DN93273_c0_g1_i15 | IRF1 | 7.45 | 19.75 | -1.43 | 0.00 |
| TRINITY_DN93273_c0_g1_i20 | | 0.54 | 5.28 | -3.45 | 0.00 |
| TRINITY_DN93273_c0_g1_i6 | IRF1 | 0.53 | 13.98 | -4.67 | 0.00 |
| TRINITY_DN93273_c0_g1_i7 | IRF1 | 5.30 | 16.13 | -1.61 | 0.00 |
| TRINITY_DN93276_c3_g1_i1 | | 7.50 | 3.38 | 1.17 | 0.00 |
| TRINITY_DN93284_c14_g1_i1 | | 0.26 | 1.93 | -2.94 | 0.00 |
| TRINITY_DN93288_c4_g2_i1 | | 0.08 | 1.83 | -3.93 | 0.00 |
| TRINITY_DN93293_c0_g2_i1 | | 85.81 | 179.59 | -1.09 | 0.00 |
| TRINITY_DN93300_c1_g1_i1 | | 48.12 | 80.64 | -0.76 | 0.00 |
| TRINITY_DN93301_c3_g1_i1 | PC11X | 0.02 | 1.49 | -4.64 | 0.00 |

| | | | | | |
|---|---|---|---|---|---|
| TRINITY_DN93311_c2_g1_i1 | TSAC | 4.70 | 18.90 | -2.02 | 0.00 |
| TRINITY_DN93311_c2_g1_i2 | TSAC | 0.19 | 3.13 | -4.18 | 0.00 |
| TRINITY_DN93311_c2_g1_i3 | TSAC | 0.00 | 0.75 | -5.37 | 0.00 |
| TRINITY_DN93328_c1_g6_i1 | CENPJ | 0.31 | 2.36 | -3.02 | 0.00 |
| TRINITY_DN93334_c0_g1_i3 | NEUA | 0.00 | 1.93 | -6.82 | 0.00 |
| TRINITY_DN93344_c4_g1_i15 | | 0.00 | 2.30 | -6.18 | 0.00 |
| TRINITY_DN93344_c4_g1_i16 | | 1.41 | 11.91 | -3.05 | 0.00 |
| TRINITY_DN93344_c4_g2_i1 | | 3.89 | 38.49 | -3.32 | 0.00 |
| TRINITY_DN93364_c1_g2_i1 | | 4.62 | 17.57 | -1.97 | 0.00 |
| TRINITY_DN93364_c1_g3_i1 | | 3.88 | 10.81 | -1.47 | 0.00 |
| TRINITY_DN93364_c1_g3_i2 | | 3.99 | 12.55 | -1.64 | 0.00 |
| TRINITY_DN93365_c3_g3_i2 | | 24.74 | 6.37 | 1.95 | 0.00 |
| TRINITY_DN93378_c2_g1_i8 | IL12B | 1.84 | 9.21 | -2.39 | 0.00 |
| TRINITY_DN93382_c4_g3_i1 | CX111 | 0.52 | 3.60 | -2.89 | 0.00 |
| TRINITY_DN93385_c3_g1_i3 | | 28.82 | 6.98 | 1.96 | 0.00 |
| TRINITY_DN93421_c4_g1_i1 | HHLA2 | 0.28 | 6.63 | -4.45 | 0.00 |
| TRINITY_DN93421_c4_g1_i4 | VTCN1 | 8.78 | 26.41 | -1.61 | 0.00 |
| TRINITY_DN93431_c1_g1_i3 | | 3.63 | 1.56 | 1.22 | 0.00 |
| TRINITY_DN93432_c11_g1_i1 | | 0.90 | 2.12 | -1.20 | 0.00 |
| TRINITY_DN93448_c6_g2_i1 | GRN3 | 16.26 | 40.17 | -1.31 | 0.00 |
| TRINITY_DN93448_c6_g3_i2 | GRN | 5.97 | 16.69 | -1.48 | 0.00 |
| TRINITY_DN93452_c0_g1_i1 | PLPP1 | 1.62 | 0.06 | 4.62 | 0.00 |
| TRINITY_DN93456_c6_g1_i6 | | 49.36 | 4.22 | 3.45 | 0.00 |
| TRINITY_DN93457_c4_g2_i4 | HDAC6 | 1.56 | 0.00 | 6.29 | 0.00 |
| TRINITY_DN93457_c4_g2_i5 | HDAC6 | 0.39 | 4.46 | -3.49 | 0.00 |
| TRINITY_DN93478_c4_g1_i3 | | 0.76 | 3.68 | -2.31 | 0.00 |
| TRINITY_DN93493_c8_g1_i6 | CTLA4 | 0.16 | 1.69 | -3.43 | 0.00 |
| TRINITY_DN93495_c0_g4_i2 | | 17.75 | 6.24 | 1.53 | 0.00 |
| TRINITY_DN93500_c1_g3_i4 | K1C18 | 6.03 | 0.40 | 3.40 | 0.00 |
| TRINITY_DN93531_c7_g3_i1 | | 17.05 | 43.65 | -1.38 | 0.00 |
| TRINITY_DN93539_c8_g1_i10 | | 0.71 | 3.44 | -2.20 | 0.00 |
| TRINITY_DN93551_c1_g3_i8 | CBX3 | 0.13 | 3.65 | -4.70 | 0.00 |
| TRINITY_DN93551_c1_g3_i9 | CBX3 | 0.10 | 10.06 | -6.08 | 0.00 |
| TRINITY_DN93555_c6_g1_i2 | | 0.23 | 8.83 | -3.56 | 0.00 |
| TRINITY_DN93569_c4_g1_i1 | | 0.02 | 1.52 | -4.35 | 0.00 |
| TRINITY_DN93570_c1_g1_i1 | | 5.48 | 2.80 | 0.94 | 0.00 |
| TRINITY_DN93576_c6_g1_i2 | GA45A | 2.95 | 6.87 | -1.23 | 0.00 |
| TRINITY_DN93579_c1_g1_i2 | FUT11 | 3.93 | 2.37 | 0.72 | 0.00 |
| TRINITY_DN93597_c0_g1_i2 | | 0.02 | 3.05 | -5.73 | 0.00 |
| TRINITY_DN93613_c8_g1_i1 | | 3.13 | 0.01 | 5.38 | 0.00 |
| TRINITY_DN93617_c0_g2_i1 | LYG | 0.10 | 1.10 | -3.56 | 0.00 |
| TRINITY_DN93634_c0_g2_i5 | CRVP | 88.35 | 15.78 | 2.41 | 0.00 |

| | | | | | |
|---|---|---|---|---|---|
| TRINITY_DN93656_c2_g1_i2 | GMDS | 23.11 | 42.60 | -0.93 | 0.00 |
| TRINITY_DN93663_c7_g1_i2 | | 4.60 | 40.25 | -3.38 | 0.00 |
| TRINITY_DN93674_c7_g3_i2 | | 3.05 | 0.30 | 3.35 | 0.00 |
| TRINITY_DN93676_c11_g1_i1 | | 0.30 | 6.29 | -3.56 | 0.00 |
| TRINITY_DN93689_c3_g2_i1 | | 5.18 | 25.77 | -2.46 | 0.00 |
| TRINITY_DN93701_c3_g2_i6 | | 0.75 | 9.73 | -3.64 | 0.00 |
| TRINITY_DN93707_c3_g2_i5 | PTN18 | 2.00 | 5.85 | -1.60 | 0.00 |
| TRINITY_DN93707_c4_g2_i6 | | 0.82 | 3.43 | -2.06 | 0.00 |
| TRINITY_DN93711_c2_g1_i1 | | 4.60 | 11.93 | -1.32 | 0.00 |
| TRINITY_DN93716_c2_g1_i3 | | 4.48 | 11.16 | -1.35 | 0.00 |
| TRINITY_DN93725_c3_g3_i1 | | 0.21 | 2.12 | -2.92 | 0.00 |
| TRINITY_DN93733_c3_g1_i1 | | 105.03 | 71.04 | 0.59 | 0.00 |
| TRINITY_DN93753_c0_g2_i10 | PLCG2 | 1.10 | 6.04 | -2.48 | 0.00 |
| TRINITY_DN93757_c8_g1_i1 | | 6.81 | 31.27 | -2.28 | 0.00 |
| TRINITY_DN93781_c5_g1_i1 | | 0.16 | 3.03 | -4.36 | 0.00 |
| TRINITY_DN93792_c2_g1_i4 | LMO7 | 0.00 | 4.81 | -7.57 | 0.00 |
| TRINITY_DN93794_c5_g2_i2 | PTGES | 0.06 | 1.79 | -4.42 | 0.00 |
| TRINITY_DN93813_c7_g2_i2 | | 3.81 | 0.00 | 6.75 | 0.00 |
| TRINITY_DN93813_c8_g2_i1 | | 5.17 | 1.20 | 2.27 | 0.00 |
| TRINITY_DN93814_c6_g4_i1 | | 3.03 | 10.65 | -1.86 | 0.00 |
| TRINITY_DN93826_c3_g2_i5 | KCD12 | 0.03 | 1.51 | -4.66 | 0.00 |
| TRINITY_DN93833_c2_g1_i3 | | 20.41 | 3.67 | 2.53 | 0.00 |
| TRINITY_DN93841_c0_g4_i1 | | 3.89 | 23.26 | -2.58 | 0.00 |
| TRINITY_DN93848_c2_g2_i4 | CLD8 | 46.75 | 70.52 | -0.60 | 0.00 |
| TRINITY_DN93862_c0_g2_i1 | | 19.93 | 61.55 | -1.62 | 0.00 |
| TRINITY_DN93870_c0_g1_i3 | | 0.12 | 1.53 | -3.56 | 0.00 |
| TRINITY_DN93870_c0_g1_i7 | | 1.12 | 3.64 | -1.73 | 0.00 |
| TRINITY_DN93883_c5_g5_i1 | | 1.27 | 8.02 | -2.67 | 0.00 |
| TRINITY_DN93884_c9_g1_i4 | UROL1 | 0.60 | 3.45 | -2.51 | 0.00 |
| TRINITY_DN93890_c3_g1_i2 | | 19.81 | 5.39 | 1.96 | 0.00 |
| TRINITY_DN93895_c4_g5_i1 | | 0.08 | 1.38 | -3.99 | 0.00 |
| TRINITY_DN93901_c3_g1_i1 | | 3.15 | 0.00 | 5.90 | 0.00 |
| TRINITY_DN93903_c2_g1_i1 | | 3.41 | 0.28 | 3.52 | 0.00 |
| TRINITY_DN93907_c1_g1_i2 | | 1.44 | 5.79 | -2.07 | 0.00 |
| TRINITY_DN93910_c4_g2_i6 | | 0.01 | 0.75 | -5.49 | 0.00 |
| TRINITY_DN93930_c1_g1_i1 | CLDY | 0.07 | 2.05 | -5.14 | 0.00 |
| TRINITY_DN93930_c1_g4_i1 | CLD4 | 2.90 | 20.23 | -2.84 | 0.00 |
| TRINITY_DN93934_c0_g1_i3 | GELS | 28.09 | 49.39 | -0.84 | 0.00 |
| TRINITY_DN93934_c0_g1_i5 | GELS | 20.80 | 38.51 | -0.93 | 0.00 |
| TRINITY_DN93941_c11_g1_i1 | | 1.33 | 0.37 | 1.78 | 0.00 |
| TRINITY_DN93951_c4_g1_i1 | | 7.25 | 2.00 | 1.85 | 0.00 |
| TRINITY_DN93960_c2_g1_i4 | | 2.63 | 8.15 | -1.67 | 0.00 |

| | | | | | |
|---|---|---|---|---|---|
| TRINITY_DN93960_c2_g2_i3 | K1C42 | 13.81 | 45.03 | -1.70 | 0.00 |
| TRINITY_DN93971_c6_g1_i1 | | 2.22 | 0.11 | 4.60 | 0.00 |
| TRINITY_DN94010_c8_g1_i5 | IKBA | 0.05 | 1.06 | -3.95 | 0.00 |
| TRINITY_DN94014_c1_g1_i8 | EIF3A | 26.79 | 0.84 | 4.58 | 0.00 |
| TRINITY_DN94015_c0_g1_i1 | | 2.67 | 6.72 | -1.34 | 0.00 |
| TRINITY_DN94024_c2_g2_i7 | | 4.53 | 8.27 | -0.91 | 0.00 |
| TRINITY_DN94026_c6_g2_i1 | | 4.73 | 40.54 | -3.12 | 0.00 |
| TRINITY_DN94029_c1_g1_i1 | STAG2 | 0.33 | 2.70 | -4.43 | 0.00 |
| TRINITY_DN94032_c0_g1_i1 | TAGAP | 1.91 | 4.67 | -1.29 | 0.00 |
| TRINITY_DN94032_c0_g1_i2 | TAGAP | 1.41 | 6.91 | -2.29 | 0.00 |
| TRINITY_DN94050_c9_g1_i1 | | 0.04 | 1.58 | -4.68 | 0.00 |
| TRINITY_DN94058_c1_g1_i16 | MARE1 | 0.00 | 1.19 | -5.36 | 0.00 |
| TRINITY_DN94062_c3_g2_i1 | | 1.27 | 0.00 | 5.72 | 0.00 |
| TRINITY_DN94065_c1_g2_i2 | CCDC3 | 5.33 | 2.87 | 0.90 | 0.00 |
| TRINITY_DN94066_c8_g1_i1 | | 5.67 | 1.21 | 2.22 | 0.00 |
| TRINITY_DN94070_c2_g1_i1 | C1S | 29.91 | 46.22 | -0.65 | 0.00 |
| TRINITY_DN94071_c3_g1_i3 | | 212.99 | 667.92 | -1.55 | 0.00 |
| TRINITY_DN94086_c0_g1_i5 | | 0.00 | 2.33 | -6.95 | 0.00 |
| TRINITY_DN94091_c0_g4_i2 | CATK | 24.04 | 48.60 | -0.96 | 0.00 |
| TRINITY_DN94094_c0_g3_i1 | CLD3 | 64.70 | 114.11 | -0.84 | 0.00 |
| TRINITY_DN94094_c0_g3_i4 | CLD3 | 3.33 | 18.24 | -2.54 | 0.00 |
| TRINITY_DN94094_c0_g4_i1 | CLD4 | 32.49 | 63.47 | -0.98 | 0.00 |
| TRINITY_DN94100_c2_g2_i1 | FRRS1 | 6.75 | 50.26 | -2.90 | 0.00 |
| TRINITY_DN94108_c1_g1_i10 | RSPRY | 1.70 | 0.00 | 5.48 | 0.00 |
| TRINITY_DN94114_c0_g1_i1 | | 6.53 | 15.66 | -1.27 | 0.00 |
| TRINITY_DN94124_c5_g5_i1 | | 0.61 | 4.29 | -2.95 | 0.00 |
| TRINITY_DN94128_c3_g1_i5 | CYTF | 1.65 | 9.19 | -2.37 | 0.00 |
| TRINITY_DN94141_c5_g2_i2 | ISM2 | 0.02 | 0.72 | -4.37 | 0.00 |
| TRINITY_DN94146_c2_g1_i8 | ERMAP | 0.58 | 4.45 | -2.87 | 0.00 |
| TRINITY_DN94154_c0_g1_i1 | ATS5 | 1.10 | 0.27 | 1.97 | 0.00 |
| TRINITY_DN94155_c0_g1_i3 | PKHF1 | 1.66 | 0.67 | 1.28 | 0.00 |
| TRINITY_DN94164_c2_g1_i1 | RNSL3 | 2.15 | 22.62 | -3.28 | 0.00 |
| TRINITY_DN94169_c1_g1_i4 | | 4.87 | 1.40 | 1.77 | 0.00 |
| TRINITY_DN94172_c1_g3_i3 | RA1L2 | 9.65 | 1.28 | 3.08 | 0.00 |
| TRINITY_DN94173_c0_g3_i2 | ITM2B | 60.50 | 99.79 | -0.75 | 0.00 |
| TRINITY_DN94181_c5_g2_i3 | | 3.00 | 7.82 | -1.38 | 0.00 |
| TRINITY_DN94188_c7_g1_i1 | | 4.84 | 0.49 | 3.10 | 0.00 |
| TRINITY_DN94194_c0_g2_i1 | | 6.72 | 0.00 | 6.94 | 0.00 |
| TRINITY_DN94196_c5_g1_i6 | S35F6 | 5.52 | 9.13 | -0.71 | 0.00 |
| TRINITY_DN94200_c4_g1_i5 | | 223.46 | 601.83 | -1.49 | 0.00 |
| TRINITY_DN94200_c4_g1_i6 | | 36.37 | 112.48 | -1.63 | 0.00 |
| TRINITY_DN94200_c4_g1_i7 | | 7.97 | 31.20 | -1.98 | 0.00 |

| | | | | | |
|---|---|---|---|---|---|
| TRINITY_DN94200_c4_g6_i1 | | 0.43 | 8.05 | -4.24 | 0.00 |
| TRINITY_DN94200_c4_g7_i1 | | 0.10 | 2.35 | -3.41 | 0.00 |
| TRINITY_DN94206_c6_g2_i2 | | 0.06 | 1.88 | -4.16 | 0.00 |
| TRINITY_DN94228_c7_g1_i1 | B2L14 | 0.62 | 3.66 | -2.58 | 0.00 |
| TRINITY_DN94228_c7_g1_i3 | | 0.21 | 3.01 | -3.70 | 0.00 |
| TRINITY_DN94234_c2_g1_i2 | | 0.50 | 2.51 | -2.27 | 0.00 |
| TRINITY_DN94244_c0_g1_i4 | WDR37 | 1.04 | 2.71 | -1.40 | 0.00 |
| TRINITY_DN94246_c3_g1_i4 | | 0.08 | 3.07 | -5.49 | 0.00 |
| TRINITY_DN94250_c5_g3_i1 | | 0.02 | 1.04 | -4.05 | 0.00 |
| TRINITY_DN94251_c11_g3_i1 | | 0.91 | 2.94 | -1.67 | 0.00 |
| TRINITY_DN94260_c2_g1_i1 | | 519.02 | 1029.68 | -0.99 | 0.00 |
| TRINITY_DN94263_c2_g2_i8 | | 2.94 | 21.52 | -2.94 | 0.00 |
| TRINITY_DN94272_c6_g3_i1 | NSRP1 | 0.09 | 4.57 | -4.47 | 0.00 |
| TRINITY_DN94274_c4_g1_i1 | | 54.39 | 17.61 | 1.60 | 0.00 |
| TRINITY_DN94286_c5_g1_i2 | TAC2N | 0.00 | 1.19 | -5.33 | 0.00 |
| TRINITY_DN94290_c2_g1_i1 | | 0.09 | 4.40 | -5.47 | 0.00 |
| TRINITY_DN94300_c3_g1_i1 | PSA6 | 9.21 | 16.07 | -0.80 | 0.00 |
| TRINITY_DN94300_c3_g1_i2 | PSA6 | 12.68 | 37.26 | -1.62 | 0.00 |
| TRINITY_DN94314_c2_g2_i1 | | 2.49 | 13.23 | -2.46 | 0.00 |
| TRINITY_DN94317_c3_g2_i1 | | 0.04 | 1.88 | -3.91 | 0.00 |
| TRINITY_DN94321_c5_g1_i1 | | 3.30 | 22.93 | -2.82 | 0.00 |
| TRINITY_DN94327_c5_g1_i1 | | 8.99 | 28.64 | -1.69 | 0.00 |
| TRINITY_DN94328_c2_g1_i1 | NPC2 | 57.17 | 97.09 | -0.76 | 0.00 |
| TRINITY_DN94332_c6_g1_i1 | | 0.42 | 2.58 | -2.69 | 0.00 |
| TRINITY_DN94333_c0_g1_i5 | CCL20 | 1.32 | 23.33 | -4.13 | 0.00 |
| TRINITY_DN94333_c0_g1_i6 | | 0.06 | 1.35 | -3.80 | 0.00 |
| TRINITY_DN94335_c0_g1_i3 | DOCK8 | 0.00 | 0.89 | -4.09 | 0.00 |
| TRINITY_DN94339_c4_g1_i5 | | 5.22 | 24.17 | -2.32 | 0.00 |
| TRINITY_DN94342_c4_g2_i1 | | 7.70 | 28.73 | -1.91 | 0.00 |
| TRINITY_DN94342_c6_g3_i2 | | 1.46 | 7.02 | -2.20 | 0.00 |
| TRINITY_DN94348_c4_g1_i7 | PDLI1 | 25.14 | 15.23 | 0.75 | 0.00 |
| TRINITY_DN94374_c2_g1_i2 | NEUL3 | 0.03 | 1.41 | -4.68 | 0.00 |
| TRINITY_DN94376_c2_g1_i7 | IGSF3 | 1.89 | 14.45 | -2.96 | 0.00 |
| TRINITY_DN94380_c7_g3_i11 | CD63 | 79.54 | 124.86 | -0.66 | 0.00 |
| TRINITY_DN94381_c4_g1_i1 | I20RB | 1.54 | 0.13 | 3.68 | 0.00 |
| TRINITY_DN94383_c0_g1_i11 | FYB | 0.07 | 0.97 | -3.75 | 0.00 |
| TRINITY_DN94383_c0_g1_i4 | FYB | 1.67 | 8.00 | -2.28 | 0.00 |
| TRINITY_DN94392_c0_g4_i1 | BC11A | 1.47 | 3.97 | -1.39 | 0.00 |
| TRINITY_DN94401_c11_g1_i3 | AGRG4 | 0.05 | 3.65 | -6.11 | 0.00 |
| TRINITY_DN94401_c11_g1_i4 | AGRG4 | 0.05 | 2.86 | -5.79 | 0.00 |
| TRINITY_DN94407_c4_g5_i1 | | 18.28 | 11.96 | 0.61 | 0.00 |
| TRINITY_DN94425_c5_g1_i5 | FLT3 | 1.15 | 6.79 | -2.51 | 0.00 |

| | | | | | |
|---|---|---|---|---|---|
| TRINITY_DN94435_c2_g1_i1 | | 34.36 | 188.73 | -2.45 | 0.00 |
| TRINITY_DN94435_c2_g1_i15 | | 11.72 | 39.92 | -1.77 | 0.00 |
| TRINITY_DN94435_c2_g1_i3 | HB2D | 65.06 | 257.45 | -2.06 | 0.00 |
| TRINITY_DN94435_c2_g1_i5 | | 203.05 | 545.41 | -1.40 | 0.00 |
| TRINITY_DN94435_c2_g2_i1 | | 1.86 | 22.42 | -3.70 | 0.00 |
| TRINITY_DN94444_c1_g1_i1 | OST48 | 28.69 | 18.35 | 0.65 | 0.00 |
| TRINITY_DN94445_c0_g1_i6 | | 5.29 | 23.20 | -2.09 | 0.00 |
| TRINITY_DN94445_c0_g1_i7 | | 0.75 | 7.47 | -3.42 | 0.00 |
| TRINITY_DN94449_c3_g1_i1 | GDIR1 | 19.66 | 33.66 | -0.80 | 0.00 |
| TRINITY_DN94463_c5_g1_i6 | | 0.07 | 1.65 | -3.96 | 0.00 |
| TRINITY_DN94472_c2_g3_i1 | | 8.80 | 2.15 | 2.12 | 0.00 |
| TRINITY_DN94483_c2_g3_i1 | | 13.62 | 4.37 | 1.64 | 0.00 |
| TRINITY_DN94496_c3_g1_i9 | SON | 0.42 | 3.39 | -2.99 | 0.00 |
| TRINITY_DN94502_c3_g1_i2 | | 4.06 | 1.91 | 1.12 | 0.00 |
| TRINITY_DN94539_c1_g1_i8 | | 0.49 | 5.00 | -3.55 | 0.00 |
| TRINITY_DN94540_c5_g1_i1 | | 0.23 | 4.21 | -3.40 | 0.00 |
| TRINITY_DN94543_c11_g1_i2 | | 0.18 | 1.57 | -3.20 | 0.00 |
| TRINITY_DN94544_c7_g1_i1 | | 8.83 | 2.59 | 1.79 | 0.00 |
| TRINITY_DN94557_c5_g2_i1 | | 20.52 | 49.98 | -1.31 | 0.00 |
| TRINITY_DN94557_c6_g2_i1 | | 0.00 | 1.37 | -5.24 | 0.00 |
| TRINITY_DN94562_c2_g1_i12 | IKZF1 | 0.19 | 2.07 | -3.48 | 0.00 |
| TRINITY_DN94562_c2_g1_i3 | IKZF1 | 0.00 | 0.57 | -5.05 | 0.00 |
| TRINITY_DN94567_c5_g4_i2 | LADD | 1.41 | 129.37 | -6.43 | 0.00 |
| TRINITY_DN94572_c3_g2_i7 | CHM4B | 0.82 | 18.00 | -4.39 | 0.00 |
| TRINITY_DN94578_c5_g1_i4 | I12R2 | 0.00 | 0.94 | -6.15 | 0.00 |
| TRINITY_DN94580_c0_g2_i1 | | 0.53 | 14.81 | -4.63 | 0.00 |
| TRINITY_DN94582_c4_g1_i6 | | 1.57 | 0.00 | 4.99 | 0.00 |
| TRINITY_DN94622_c4_g1_i4 | | 0.07 | 2.00 | -4.63 | 0.00 |
| TRINITY_DN94627_c11_g2_i5 | | 0.03 | 0.48 | -3.35 | 0.00 |
| TRINITY_DN94627_c9_g1_i1 | | 15.39 | 6.38 | 1.30 | 0.00 |
| TRINITY_DN94649_c2_g1_i1 | | 29.88 | 15.58 | 0.92 | 0.00 |
| TRINITY_DN94655_c3_g1_i1 | | 1.55 | 0.40 | 1.86 | 0.00 |
| TRINITY_DN94655_c5_g1_i1 | IL8 | 2.40 | 6.04 | -1.40 | 0.00 |
| TRINITY_DN94662_c2_g1_i11 | | 1.13 | 4.22 | -1.98 | 0.00 |
| TRINITY_DN94664_c9_g5_i1 | | 0.18 | 1.45 | -3.13 | 0.00 |
| TRINITY_DN94674_c1_g1_i13 | | 0.57 | 3.54 | -2.61 | 0.00 |
| TRINITY_DN94674_c1_g1_i2 | REG1B | 0.00 | 1.72 | -4.90 | 0.00 |
| TRINITY_DN94676_c5_g1_i2 | | 25.67 | 81.10 | -1.67 | 0.00 |
| TRINITY_DN94676_c5_g1_i3 | | 6.73 | 25.69 | -1.89 | 0.00 |
| TRINITY_DN94676_c5_g1_i4 | | 0.85 | 12.76 | -3.61 | 0.00 |
| TRINITY_DN94706_c4_g4_i2 | | 3.11 | 0.79 | 2.11 | 0.00 |
| TRINITY_DN94708_c0_g1_i2 | VP26A | 3.61 | 0.00 | 7.43 | 0.00 |

| | | | | | |
|---|---|---|---|---|---|
| TRINITY_DN94714_c12_g2_i1 | | 2.37 | 6.86 | -1.55 | 0.00 |
| TRINITY_DN94716_c0_g1_i2 | SE1L3 | 0.75 | 2.38 | -1.69 | 0.00 |
| TRINITY_DN94747_c0_g2_i10 | SLK | 0.63 | 3.18 | -2.35 | 0.00 |
| TRINITY_DN94753_c0_g2_i1 | GRAB | 0.11 | 2.50 | -4.08 | 0.00 |
| TRINITY_DN94753_c0_g2_i5 | GRZ1 | 0.77 | 3.58 | -2.27 | 0.00 |
| TRINITY_DN94766_c11_g3_i1 | | 1.36 | 3.63 | -1.37 | 0.00 |
| TRINITY_DN94774_c11_g2_i6 | | 0.09 | 1.74 | -3.44 | 0.00 |
| TRINITY_DN94777_c8_g1_i1 | | 1.51 | 9.59 | -2.61 | 0.00 |
| TRINITY_DN94787_c1_g1_i13 | JAK2 | 0.06 | 0.48 | -3.06 | 0.00 |
| TRINITY_DN94787_c1_g1_i2 | JAK2 | 0.19 | 2.66 | -3.81 | 0.00 |
| TRINITY_DN94810_c2_g1_i7 | CO9A1 | 1.37 | 0.00 | 6.04 | 0.00 |
| TRINITY_DN94817_c3_g2_i4 | | 0.19 | 3.10 | -3.86 | 0.00 |
| TRINITY_DN94825_c6_g1_i1 | | 0.69 | 2.83 | -2.07 | 0.00 |
| TRINITY_DN94831_c3_g1_i1 | RAC2 | 13.58 | 24.87 | -0.90 | 0.00 |
| TRINITY_DN94831_c3_g1_i5 | RAC2 | 13.82 | 30.11 | -1.10 | 0.00 |
| TRINITY_DN94859_c10_g1_i1 | | 14.04 | 1.18 | 3.61 | 0.00 |
| TRINITY_DN94860_c5_g2_i1 | | 18.51 | 45.00 | -1.25 | 0.00 |
| TRINITY_DN94881_c3_g7_i2 | | 11.55 | 2.47 | 2.22 | 0.00 |
| TRINITY_DN94894_c1_g1_i11 | CDN1B | 14.38 | 24.46 | -0.77 | 0.00 |
| TRINITY_DN94896_c1_g1_i1 | | 0.98 | 22.38 | -4.39 | 0.00 |
| TRINITY_DN94903_c1_g1_i10 | | 0.03 | 0.72 | -4.38 | 0.00 |
| TRINITY_DN94908_c0_g2_i2 | GRK6 | 1.09 | 6.15 | -2.50 | 0.00 |
| TRINITY_DN94909_c0_g3_i1 | EHD1 | 0.57 | 1.95 | -1.82 | 0.00 |
| TRINITY_DN94916_c7_g2_i1 | | 45.02 | 30.06 | 0.57 | 0.00 |
| TRINITY_DN94927_c0_g2_i14 | | 0.00 | 3.84 | -5.01 | 0.00 |
| TRINITY_DN94927_c0_g2_i3 | ZN185 | 1.03 | 11.78 | -3.52 | 0.00 |
| TRINITY_DN94932_c3_g1_i1 | | 1.07 | 7.63 | -2.84 | 0.00 |
| TRINITY_DN94943_c5_g2_i2 | PHTF1 | 0.00 | 2.38 | -5.81 | 0.00 |
| TRINITY_DN94953_c0_g1_i1 | GAS1 | 7.06 | 4.04 | 0.80 | 0.00 |
| TRINITY_DN94964_c5_g3_i1 | | 1.82 | 10.10 | -2.42 | 0.00 |
| TRINITY_DN94968_c0_g1_i3 | UB2D2 | 16.60 | 22.76 | -0.46 | 0.00 |
| TRINITY_DN94968_c0_g1_i7 | UB2D2 | 2.95 | 0.11 | 4.89 | 0.00 |
| TRINITY_DN94989_c3_g2_i1 | ASIC1 | 5.25 | 1.75 | 1.63 | 0.00 |
| TRINITY_DN95004_c5_g2_i7 | SAT1 | 0.94 | 2.45 | -1.40 | 0.00 |
| TRINITY_DN95004_c5_g3_i1 | | 2.57 | 15.34 | -2.46 | 0.00 |
| TRINITY_DN95014_c3_g1_i2 | | 1.47 | 8.26 | -2.64 | 0.00 |
| TRINITY_DN95017_c2_g1_i2 | DTX1 | 0.12 | 1.97 | -3.60 | 0.00 |
| TRINITY_DN95017_c4_g1_i4 | SEPT9 | 7.98 | 18.94 | -1.26 | 0.00 |
| TRINITY_DN95024_c0_g1_i1 | WNT11 | 5.99 | 2.58 | 1.19 | 0.00 |
| TRINITY_DN95043_c10_g1_i1 | | 7.00 | 0.72 | 3.31 | 0.00 |
| TRINITY_DN95043_c10_g2_i1 | | 5.40 | 0.23 | 4.48 | 0.00 |
| TRINITY_DN95044_c9_g2_i1 | | 4.68 | 29.51 | -2.72 | 0.00 |

| | | | | | |
|---|---|---|---|---|---|
| TRINITY_DN95056_c4_g1_i1 | SKAP1 | 0.27 | 3.56 | -3.82 | 0.00 |
| TRINITY_DN95056_c4_g1_i3 | SKAP1 | 0.78 | 4.70 | -2.59 | 0.00 |
| TRINITY_DN95056_c4_g1_i5 | SKAP1 | 0.85 | 4.03 | -2.21 | 0.00 |
| TRINITY_DN95076_c2_g1_i2 | | 0.75 | 6.81 | -3.21 | 0.00 |
| TRINITY_DN95076_c2_g2_i1 | FSTL1 | 33.14 | 21.52 | 0.63 | 0.00 |
| TRINITY_DN95076_c2_g3_i1 | FSTL1 | 83.77 | 36.81 | 1.21 | 0.00 |
| TRINITY_DN95079_c11_g1_i1 | | 27.09 | 9.23 | 0.94 | 0.00 |
| TRINITY_DN95088_c3_g3_i2 | | 0.06 | 0.53 | -2.99 | 0.00 |
| TRINITY_DN95088_c3_g3_i8 | | 0.40 | 3.74 | -3.17 | 0.00 |
| TRINITY_DN95101_c0_g1_i2 | OST2B | 58.15 | 30.77 | 0.92 | 0.00 |
| TRINITY_DN95101_c0_g1_i3 | OST2B | 19.04 | 9.03 | 1.07 | 0.00 |
| TRINITY_DN95103_c0_g4_i1 | | 4.68 | 0.19 | 3.93 | 0.00 |
| TRINITY_DN95109_c6_g1_i1 | BTK | 1.66 | 6.45 | -2.00 | 0.00 |
| TRINITY_DN95113_c7_g1_i4 | | 0.00 | 3.14 | -4.40 | 0.00 |
| TRINITY_DN95121_c1_g1_i3 | CTDS2 | 0.00 | 2.83 | -22.54 | 0.00 |
| TRINITY_DN95126_c6_g2_i3 | | 0.48 | 1.86 | -2.03 | 0.00 |
| TRINITY_DN95127_c10_g1_i2 | CSL2 | 10.18 | 211.69 | -4.38 | 0.00 |
| TRINITY_DN95127_c10_g1_i7 | CSL2 | 1.23 | 21.82 | -4.08 | 0.00 |
| TRINITY_DN95127_c10_g1_i9 | CSL2 | 20.69 | 273.60 | -3.67 | 0.00 |
| TRINITY_DN95136_c2_g3_i2 | K1C13 | 250.07 | 48.43 | 2.48 | 0.00 |
| TRINITY_DN95139_c0_g1_i7 | RND3 | 3.67 | 1.48 | 1.29 | 0.00 |
| TRINITY_DN95148_c8_g2_i2 | | 0.18 | 5.21 | -4.81 | 0.00 |
| TRINITY_DN95157_c0_g1_i2 | | 0.05 | 1.43 | -5.18 | 0.00 |
| TRINITY_DN95157_c0_g1_i9 | RAB1B | 0.00 | 1.36 | -6.07 | 0.00 |
| TRINITY_DN95183_c0_g4_i1 | BID | 9.15 | 17.27 | -0.94 | 0.00 |
| TRINITY_DN95184_c0_g4_i1 | | 17.37 | 8.87 | 0.96 | 0.00 |
| TRINITY_DN95190_c0_g1_i3 | CO1A2 | 152.96 | 76.18 | 1.12 | 0.00 |
| TRINITY_DN95190_c0_g1_i6 | CO1A2 | 117.56 | 61.34 | 0.99 | 0.00 |
| TRINITY_DN95190_c0_g1_i7 | CO1A2 | 75.23 | 23.92 | 1.73 | 0.00 |
| TRINITY_DN95224_c0_g1_i4 | TSN1 | 0.34 | 4.62 | -3.81 | 0.00 |
| TRINITY_DN95224_c0_g1_i6 | TSN1 | 0.22 | 3.39 | -3.55 | 0.00 |
| TRINITY_DN95229_c11_g1_i1 | | 6.75 | 12.57 | -0.91 | 0.00 |
| TRINITY_DN95243_c4_g1_i1 | CYH4 | 3.07 | 6.71 | -1.13 | 0.00 |
| TRINITY_DN95256_c6_g6_i1 | | 14.87 | 5.02 | 1.62 | 0.00 |
| TRINITY_DN95277_c3_g1_i4 | NEIL1 | 3.98 | 1.65 | 1.30 | 0.00 |
| TRINITY_DN95280_c4_g1_i13 | DHR11 | 10.75 | 20.15 | -0.94 | 0.00 |
| TRINITY_DN95288_c9_g3_i2 | GGH | 16.72 | 29.33 | -0.83 | 0.00 |
| TRINITY_DN95289_c5_g11_i3 | | 0.23 | 2.25 | -3.14 | 0.00 |
| TRINITY_DN95291_c0_g4_i2 | BT2A1 | 18.33 | 43.30 | -1.29 | 0.00 |
| TRINITY_DN95296_c8_g1_i1 | CCL14 | 20.92 | 76.59 | -1.91 | 0.00 |
| TRINITY_DN95297_c4_g1_i18 | ITB7 | 5.20 | 14.08 | -1.45 | 0.00 |
| TRINITY_DN95297_c4_g1_i2 | ITB7 | 0.20 | 1.36 | -2.72 | 0.00 |

| | | | | | |
|---|---|---|---|---|---|
| TRINITY_DN95299_c8_g1_i1 | | 1.02 | 3.63 | -1.82 | 0.00 |
| TRINITY_DN95305_c3_g8_i1 | | 0.69 | 2.86 | -2.09 | 0.00 |
| TRINITY_DN95309_c2_g1_i1 | | 0.38 | 1.00 | -1.43 | 0.00 |
| TRINITY_DN95325_c1_g1_i4 | PSB7 | 30.83 | 56.80 | -0.88 | 0.00 |
| TRINITY_DN95325_c1_g2_i4 | PB6LB | 15.21 | 28.15 | -0.91 | 0.00 |
| TRINITY_DN95327_c0_g1_i1 | | 0.11 | 1.22 | -3.42 | 0.00 |
| TRINITY_DN95327_c0_g1_i13 | | 2.57 | 10.00 | -1.98 | 0.00 |
| TRINITY_DN95327_c0_g1_i9 | | 6.76 | 12.34 | -0.87 | 0.00 |
| TRINITY_DN95347_c9_g5_i1 | RM22 | 0.62 | 2.76 | -2.13 | 0.00 |
| TRINITY_DN95359_c2_g1_i2 | | 0.16 | 1.43 | -3.14 | 0.00 |
| TRINITY_DN95368_c7_g4_i1 | | 0.70 | 5.60 | -3.23 | 0.00 |
| TRINITY_DN95394_c2_g1_i2 | | 83.08 | 302.42 | -1.83 | 0.00 |
| TRINITY_DN95405_c6_g2_i2 | | 0.08 | 29.58 | -8.01 | 0.00 |
| TRINITY_DN95412_c5_g5_i2 | | 1.01 | 11.88 | -3.58 | 0.00 |
| TRINITY_DN95422_c13_g1_i1 | | 13.75 | 5.44 | 1.33 | 0.00 |
| TRINITY_DN95425_c0_g3_i1 | | 1.54 | 3.85 | -1.32 | 0.00 |
| TRINITY_DN95429_c4_g4_i1 | | 13.97 | 56.76 | -2.08 | 0.00 |
| TRINITY_DN95445_c9_g1_i4 | STAT4 | 0.50 | 5.87 | -2.99 | 0.00 |
| TRINITY_DN95445_c9_g2_i1 | STAT4 | 0.00 | 1.04 | -4.13 | 0.00 |
| TRINITY_DN95446_c2_g1_i1 | | 3.60 | 20.06 | -2.45 | 0.00 |
| TRINITY_DN95447_c0_g1_i1 | | 4.87 | 25.46 | -2.51 | 0.00 |
| TRINITY_DN95451_c0_g1_i15 | SLAF8 | 0.03 | 0.86 | -3.68 | 0.00 |
| TRINITY_DN95460_c0_g2_i3 | ASCC2 | 0.83 | 0.00 | 5.94 | 0.00 |
| TRINITY_DN95462_c0_g2_i6 | RIOK3 | 18.38 | 10.04 | 0.90 | 0.00 |
| TRINITY_DN95469_c9_g2_i1 | LX15B | 2.53 | 8.24 | -1.70 | 0.00 |
| TRINITY_DN95469_c9_g3_i1 | | 9.22 | 21.65 | -1.24 | 0.00 |
| TRINITY_DN95471_c5_g1_i4 | | 1.02 | 6.52 | -2.73 | 0.00 |
| TRINITY_DN95491_c0_g2_i3 | ACSL6 | 0.64 | 0.00 | 5.75 | 0.00 |
| TRINITY_DN95497_c4_g2_i3 | ATS12 | 4.05 | 2.21 | 0.88 | 0.00 |
| TRINITY_DN95517_c7_g3_i1 | | 9.92 | 40.02 | -1.98 | 0.00 |
| TRINITY_DN95520_c10_g1_i2 | | 0.28 | 4.35 | -3.73 | 0.00 |
| TRINITY_DN95527_c4_g2_i1 | | 0.21 | 1.72 | -2.79 | 0.00 |
| TRINITY_DN95537_c0_g1_i11 | LPCT4 | 0.02 | 0.82 | -4.30 | 0.00 |
| TRINITY_DN95538_c1_g1_i3 | TXB1B | 6.26 | 0.00 | 7.57 | 0.00 |
| TRINITY_DN95547_c0_g1_i2 | | 0.12 | 1.19 | -3.49 | 0.00 |
| TRINITY_DN95554_c2_g2_i1 | SCOCA | 0.04 | 1.89 | -5.12 | 0.00 |
| TRINITY_DN95563_c4_g1_i1 | NAR5 | 0.03 | 8.31 | -6.97 | 0.00 |
| TRINITY_DN95569_c5_g1_i1 | M4A4A | 6.87 | 37.37 | -2.52 | 0.00 |
| TRINITY_DN95577_c1_g2_i7 | LEG3 | 5.92 | 0.41 | 3.94 | 0.00 |
| TRINITY_DN95585_c4_g1_i1 | | 26.66 | 54.20 | -1.07 | 0.00 |
| TRINITY_DN95585_c4_g7_i1 | | 12.51 | 7.59 | 0.70 | 0.00 |
| TRINITY_DN95587_c5_g2_i1 | | 1.05 | 3.13 | -1.60 | 0.00 |

| | | | | | |
|---|---|---|---|---|---|
| TRINITY_DN95592_c7_g1_i1 | | 14.91 | 1.87 | 3.08 | 0.00 |
| TRINITY_DN95609_c5_g3_i2 | | 0.00 | 2.52 | -4.37 | 0.00 |
| TRINITY_DN95623_c1_g7_i1 | | 8.32 | 25.92 | -1.67 | 0.00 |
| TRINITY_DN95629_c3_g1_i5 | TVA3 | 0.00 | 2.42 | -4.24 | 0.00 |
| TRINITY_DN95634_c5_g1_i2 | KLF4 | 32.41 | 58.82 | -0.89 | 0.00 |
| TRINITY_DN95643_c5_g3_i3 | NPTN | 1.48 | 0.32 | 2.41 | 0.00 |
| TRINITY_DN95644_c0_g1_i1 | | 21.37 | 7.80 | 1.43 | 0.00 |
| TRINITY_DN95656_c4_g1_i1 | GBP1 | 16.54 | 41.63 | -1.35 | 0.00 |
| TRINITY_DN95673_c5_g2_i2 | KCNQ2 | 1.59 | 0.00 | 5.93 | 0.00 |
| TRINITY_DN95688_c10_g1_i1 | | 0.78 | 3.49 | -2.16 | 0.00 |
| TRINITY_DN95688_c7_g1_i5 | LRRF2 | 0.05 | 0.73 | -3.39 | 0.00 |
| TRINITY_DN95696_c2_g1_i2 | IL12B | 0.03 | 3.45 | -6.11 | 0.00 |
| TRINITY_DN95696_c2_g1_i4 | | 0.21 | 6.43 | -4.82 | 0.00 |
| TRINITY_DN95696_c2_g1_i7 | IL12B | 0.54 | 6.75 | -3.61 | 0.00 |
| TRINITY_DN95699_c0_g1_i3 | HVM16 | 0.00 | 6.17 | -5.51 | 0.00 |
| TRINITY_DN95699_c0_g3_i2 | HV02 | 0.00 | 4.84 | -5.11 | 0.00 |
| TRINITY_DN95710_c3_g1_i1 | MKB | 19.12 | 8.87 | 1.11 | 0.00 |
| TRINITY_DN95719_c3_g1_i1 | CO4A2 | 6.14 | 3.47 | 0.82 | 0.00 |
| TRINITY_DN95725_c7_g2_i3 | AMPB | 6.56 | 11.09 | -0.77 | 0.00 |
| TRINITY_DN95738_c6_g3_i3 | | 0.31 | 1.54 | -2.41 | 0.00 |
| TRINITY_DN95741_c2_g6_i1 | FUCO2 | 17.77 | 23.54 | -0.42 | 0.00 |
| TRINITY_DN95750_c10_g1_i1 | | 2.42 | 4.55 | -0.95 | 0.00 |
| TRINITY_DN95753_c1_g1_i1 | CCR9 | 2.10 | 6.99 | -1.75 | 0.00 |
| TRINITY_DN95753_c1_g1_i3 | CCR9 | 3.60 | 12.95 | -1.83 | 0.00 |
| TRINITY_DN95767_c8_g2_i1 | | 6.19 | 1.77 | 1.78 | 0.00 |
| TRINITY_DN95772_c7_g1_i1 | | 1.87 | 4.65 | -1.30 | 0.00 |
| TRINITY_DN95776_c0_g1_i1 | | 1.03 | 21.64 | -4.28 | 0.00 |
| TRINITY_DN95795_c5_g2_i1 | | 2.19 | 19.67 | -3.21 | 0.00 |
| TRINITY_DN95810_c7_g3_i1 | | 4.02 | 17.94 | -2.22 | 0.00 |
| TRINITY_DN95813_c4_g2_i2 | | 0.13 | 2.58 | -4.11 | 0.00 |
| TRINITY_DN95829_c13_g2_i4 | | 55.74 | 240.26 | -2.00 | 0.00 |
| TRINITY_DN95834_c2_g1_i6 | CTNB1 | 16.49 | 8.06 | 1.01 | 0.00 |
| TRINITY_DN95838_c1_g1_i4 | HA2B | 171.10 | 938.36 | -2.53 | 0.00 |
| TRINITY_DN95838_c1_g1_i9 | | 3.20 | 18.55 | -2.58 | 0.00 |
| TRINITY_DN95845_c2_g1_i10 | RNT2 | 11.47 | 35.12 | -1.68 | 0.00 |
| TRINITY_DN95846_c2_g1_i3 | FCGR1 | 3.82 | 8.23 | -1.09 | 0.00 |
| TRINITY_DN95848_c7_g1_i1 | | 25.20 | 43.82 | -0.80 | 0.00 |
| TRINITY_DN95854_c6_g1_i1 | | 1.94 | 11.23 | -2.66 | 0.00 |
| TRINITY_DN95867_c0_g1_i4 | | 0.06 | 1.73 | -4.63 | 0.00 |
| TRINITY_DN95881_c9_g1_i1 | ADRB2 | 0.06 | 0.91 | -3.71 | 0.00 |
| TRINITY_DN95882_c5_g3_i3 | I22R2 | 0.61 | 4.66 | -2.94 | 0.00 |
| TRINITY_DN95885_c2_g1_i1 | CD3Z | 2.18 | 6.80 | -1.69 | 0.00 |

| | | | | | |
|---|---|---|---|---|---|
| TRINITY_DN95895_c3_g1_i1 | | 1.61 | 11.26 | -2.77 | 0.00 |
| TRINITY_DN95899_c5_g1_i1 | | 0.16 | 0.93 | -2.57 | 0.00 |
| TRINITY_DN95906_c4_g1_i14 | TESP1 | 0.78 | 2.67 | -1.86 | 0.00 |
| TRINITY_DN95912_c2_g1_i5 | PROX1 | 0.78 | 1.88 | -1.24 | 0.00 |
| TRINITY_DN95921_c4_g1_i5 | | 18.57 | 65.36 | -1.78 | 0.00 |
| TRINITY_DN95924_c1_g1_i8 | ODO2 | 0.00 | 1.99 | -5.78 | 0.00 |
| TRINITY_DN95929_c5_g1_i2 | | 0.07 | 0.85 | -3.63 | 0.00 |
| TRINITY_DN95929_c5_g1_i3 | | 5.31 | 15.39 | -1.60 | 0.00 |
| TRINITY_DN95938_c0_g1_i1 | KAPCB | 1.05 | 3.20 | -1.63 | 0.00 |
| TRINITY_DN95939_c6_g1_i2 | | 0.00 | 5.79 | -7.29 | 0.00 |
| TRINITY_DN95946_c3_g1_i2 | SNX1 | 2.67 | 0.57 | 3.85 | 0.00 |
| TRINITY_DN95948_c14_g1_i1 | K1C13 | 126.22 | 53.12 | 1.31 | 0.00 |
| TRINITY_DN95948_c14_g1_i3 | K1C13 | 45.52 | 16.06 | 1.55 | 0.00 |
| TRINITY_DN95948_c14_g1_i4 | K1C13 | 96.96 | 32.75 | 1.71 | 0.00 |
| TRINITY_DN95948_c14_g1_i6 | K1C13 | 225.13 | 46.18 | 2.36 | 0.00 |
| TRINITY_DN95948_c14_g2_i1 | K1C13 | 220.79 | 81.91 | 1.51 | 0.00 |
| TRINITY_DN95975_c0_g1_i3 | RB3GP | 2.17 | 8.82 | -2.17 | 0.00 |
| TRINITY_DN95976_c2_g1_i3 | HDHD5 | 0.89 | 0.17 | 2.41 | 0.00 |
| TRINITY_DN95985_c3_g1_i2 | | 1.78 | 8.31 | -2.21 | 0.00 |
| TRINITY_DN95985_c3_g4_i2 | | 23.00 | 124.20 | -2.48 | 0.00 |
| TRINITY_DN95988_c0_g4_i1 | | 0.88 | 0.00 | 5.09 | 0.00 |
| TRINITY_DN95995_c0_g1_i13 | | 0.18 | 1.76 | -3.37 | 0.00 |
| TRINITY_DN95997_c14_g1_i1 | | 0.40 | 9.92 | -4.07 | 0.00 |
| TRINITY_DN96021_c8_g1_i1 | NMI | 0.00 | 7.36 | -8.23 | 0.00 |
| TRINITY_DN96026_c4_g1_i1 | | 0.00 | 1.91 | -4.47 | 0.00 |
| TRINITY_DN96032_c4_g2_i1 | | 1.64 | 7.75 | -2.26 | 0.00 |
| TRINITY_DN96047_c2_g1_i2 | | 190.99 | 55.10 | 1.86 | 0.00 |
| TRINITY_DN96047_c2_g1_i3 | | 96.23 | 29.18 | 1.75 | 0.00 |
| TRINITY_DN96047_c2_g1_i7 | | 195.80 | 85.92 | 1.23 | 0.00 |
| TRINITY_DN96047_c2_g2_i1 | | 117.01 | 32.80 | 1.93 | 0.00 |
| TRINITY_DN96047_c2_g2_i2 | | 13.70 | 6.01 | 1.19 | 0.00 |
| TRINITY_DN96056_c1_g1_i2 | MOT7 | 0.03 | 1.53 | -4.64 | 0.00 |
| TRINITY_DN96075_c0_g4_i1 | SIPA1 | 1.80 | 5.92 | -1.72 | 0.00 |
| TRINITY_DN96092_c6_g1_i4 | ICOSL | 0.47 | 2.84 | -2.60 | 0.00 |
| TRINITY_DN96098_c0_g2_i3 | HCLS1 | 11.13 | 32.14 | -1.57 | 0.00 |
| TRINITY_DN96098_c0_g5_i1 | HCLS1 | 5.71 | 12.20 | -1.09 | 0.00 |
| TRINITY_DN96101_c0_g3_i1 | VASP | 8.50 | 17.85 | -1.09 | 0.00 |
| TRINITY_DN96106_c1_g1_i2 | UBIQ | 98.81 | 165.59 | -0.75 | 0.00 |
| TRINITY_DN96107_c0_g1_i1 | KVD28 | 0.88 | 20.66 | -4.51 | 0.00 |
| TRINITY_DN96107_c0_g2_i10 | KV401 | 0.91 | 5.98 | -2.79 | 0.00 |
| TRINITY_DN96107_c0_g2_i11 | KV15 | 3.46 | 35.35 | -3.48 | 0.00 |
| TRINITY_DN96107_c0_g2_i7 | KV401 | 0.00 | 17.16 | -5.75 | 0.00 |

| | | | | | |
|---|---|---|---|---|---|
| TRINITY_DN96149_c7_g1_i2 | RGS18 | 4.71 | 10.75 | -1.21 | 0.00 |
| TRINITY_DN96168_c0_g3_i1 | PCDG3 | 6.84 | 3.48 | 0.96 | 0.00 |
| TRINITY_DN96184_c10_g1_i1 | | 3.34 | 8.68 | -1.37 | 0.00 |
| TRINITY_DN96186_c6_g1_i4 | MCUR1 | 0.02 | 0.56 | -3.56 | 0.00 |
| TRINITY_DN96200_c4_g1_i2 | OIT3 | 0.07 | 1.12 | -4.01 | 0.00 |
| TRINITY_DN96215_c6_g2_i1 | | 0.41 | 1.44 | -1.82 | 0.00 |
| TRINITY_DN96232_c1_g2_i1 | OTC | 2.85 | 1.19 | 1.28 | 0.00 |
| TRINITY_DN96240_c5_g1_i2 | GFPT2 | 0.27 | 7.40 | -4.79 | 0.00 |
| TRINITY_DN96245_c0_g3_i1 | EPYC | 1.21 | 0.07 | 4.05 | 0.00 |
| TRINITY_DN96250_c5_g2_i1 | DLX3B | 8.59 | 4.96 | 0.78 | 0.00 |
| TRINITY_DN96251_c7_g2_i1 | KIBRA | 0.02 | 0.64 | -4.54 | 0.00 |
| TRINITY_DN96260_c3_g2_i6 | | 3.28 | 13.19 | -1.98 | 0.00 |
| TRINITY_DN96269_c0_g1_i2 | | 5.44 | 2.38 | 1.21 | 0.00 |
| TRINITY_DN96270_c1_g1_i6 | TGFB1 | 0.03 | 1.62 | -4.87 | 0.00 |
| TRINITY_DN96270_c2_g2_i1 | | 15.12 | 7.75 | 0.96 | 0.00 |
| TRINITY_DN96272_c1_g3_i1 | 1A25 | 1.13 | 2.83 | -1.37 | 0.00 |
| TRINITY_DN96292_c0_g1_i2 | | 4.51 | 377.49 | -6.27 | 0.00 |
| TRINITY_DN96292_c0_g1_i3 | | 1.01 | 65.35 | -5.47 | 0.00 |
| TRINITY_DN96292_c0_g1_i5 | | 0.37 | 61.77 | -7.26 | 0.00 |
| TRINITY_DN96292_c0_g1_i7 | | 0.17 | 25.85 | -6.61 | 0.00 |
| TRINITY_DN96302_c2_g3_i2 | | 16.21 | 5.61 | 1.56 | 0.00 |
| TRINITY_DN96305_c3_g5_i1 | | 0.07 | 0.83 | -3.27 | 0.00 |
| TRINITY_DN96306_c2_g2_i1 | | 22.24 | 12.57 | 0.83 | 0.00 |
| TRINITY_DN96314_c1_g1_i1 | TPRGL | 2.97 | 4.83 | -0.72 | 0.00 |
| TRINITY_DN96315_c7_g2_i7 | | 6.01 | 0.50 | 3.54 | 0.00 |
| TRINITY_DN96322_c4_g1_i1 | | 5.16 | 25.32 | -2.17 | 0.00 |
| TRINITY_DN96336_c1_g3_i1 | C163A | 1.75 | 5.71 | -1.78 | 0.00 |
| TRINITY_DN96337_c3_g2_i5 | | 0.57 | 2.72 | -2.27 | 0.00 |
| TRINITY_DN96341_c2_g1_i1 | | 0.68 | 3.24 | -2.21 | 0.00 |
| TRINITY_DN96343_c0_g1_i1 | SPDEF | 0.14 | 2.51 | -4.28 | 0.00 |
| TRINITY_DN96347_c7_g1_i5 | ICEF1 | 0.00 | 0.90 | -5.20 | 0.00 |
| TRINITY_DN96354_c1_g1_i5 | PEDF | 57.46 | 34.21 | 0.75 | 0.00 |
| TRINITY_DN96356_c7_g1_i1 | CD82 | 0.00 | 4.92 | -7.36 | 0.00 |
| TRINITY_DN96356_c7_g1_i2 | CD82 | 14.91 | 4.85 | 1.58 | 0.00 |
| TRINITY_DN96377_c10_g1_i1 | | 0.99 | 9.19 | -3.30 | 0.00 |
| TRINITY_DN96385_c2_g1_i1 | | 31.43 | 11.63 | 1.46 | 0.00 |
| TRINITY_DN96410_c0_g3_i2 | | 21.33 | 12.75 | 0.73 | 0.00 |
| TRINITY_DN96438_c10_g1_i2 | | 0.07 | 0.83 | -3.12 | 0.00 |
| TRINITY_DN96442_c8_g1_i1 | | 5.08 | 2.75 | 0.90 | 0.00 |
| TRINITY_DN96446_c7_g3_i1 | | 1.07 | 6.49 | -2.86 | 0.00 |
| TRINITY_DN96458_c0_g1_i2 | SLPI | 6.58 | 1.87 | 1.86 | 0.00 |
| TRINITY_DN96473_c2_g1_i1 | | 15.37 | 10.20 | 0.57 | 0.00 |

| | | | | | |
|---|---|---|---|---|---|
| TRINITY_DN96473_c2_g2_i1 | | 2.08 | 1.13 | 0.87 | 0.00 |
| TRINITY_DN96476_c8_g1_i3 | | 0.52 | 2.39 | -2.26 | 0.00 |
| TRINITY_DN96493_c1_g4_i1 | TMED2 | 0.00 | 6.42 | -8.21 | 0.00 |
| TRINITY_DN96498_c1_g1_i1 | CL17A | 0.58 | 2.42 | -2.12 | 0.00 |
| TRINITY_DN96498_c1_g1_i3 | ASGR2 | 0.08 | 1.38 | -3.80 | 0.00 |
| TRINITY_DN96511_c0_g1_i5 | 5NTD | 0.59 | 1.71 | -1.58 | 0.00 |
| TRINITY_DN96513_c0_g1_i4 | CP46A | 0.11 | 1.15 | -3.53 | 0.00 |
| TRINITY_DN96515_c0_g1_i1 | | 9.06 | 31.29 | -1.82 | 0.00 |
| TRINITY_DN96515_c1_g1_i4 | | 2.72 | 6.03 | -1.16 | 0.00 |
| TRINITY_DN96522_c0_g1_i6 | PF23B | 0.77 | 0.02 | 5.67 | 0.00 |
| TRINITY_DN96524_c13_g1_i15 | | 0.14 | 2.33 | -4.30 | 0.00 |
| TRINITY_DN96525_c4_g1_i4 | | 210.66 | 17.30 | 3.71 | 0.00 |
| TRINITY_DN96525_c4_g1_i5 | | 7.94 | 0.63 | 3.80 | 0.00 |
| TRINITY_DN96526_c1_g1_i1 | | 2.07 | 0.00 | 5.70 | 0.00 |
| TRINITY_DN96537_c4_g2_i3 | | 0.00 | 4.67 | -4.21 | 0.00 |
| TRINITY_DN96538_c1_g1_i1 | LCK | 2.40 | 8.77 | -1.84 | 0.00 |
| TRINITY_DN96540_c5_g1_i6 | | 32.11 | 1.45 | 4.46 | 0.00 |
| TRINITY_DN96547_c6_g1_i1 | | 3.58 | 9.30 | -1.41 | 0.00 |
| TRINITY_DN96550_c0_g2_i10 | ELF3 | 16.46 | 45.02 | -1.51 | 0.00 |
| TRINITY_DN96550_c0_g2_i12 | ELF3 | 0.07 | 1.73 | -4.41 | 0.00 |
| TRINITY_DN96550_c0_g3_i2 | EHF | 0.00 | 0.70 | -4.05 | 0.00 |
| TRINITY_DN96550_c0_g3_i3 | EHF | 6.52 | 22.80 | -1.81 | 0.00 |
| TRINITY_DN96561_c6_g1_i4 | | 0.00 | 14.83 | -5.79 | 0.00 |
| TRINITY_DN96591_c1_g2_i2 | DEFI6 | 1.01 | 4.25 | -2.01 | 0.00 |
| TRINITY_DN96591_c1_g3_i1 | | 0.77 | 4.54 | -2.63 | 0.00 |
| TRINITY_DN96591_c2_g1_i1 | DEFI6 | 1.36 | 4.43 | -1.70 | 0.00 |
| TRINITY_DN96593_c0_g1_i14 | ZAP70 | 0.09 | 0.89 | -3.25 | 0.00 |
| TRINITY_DN96593_c0_g1_i20 | ZAP70 | 0.42 | 2.90 | -2.73 | 0.00 |
| TRINITY_DN96600_c1_g1_i7 | DAZP1 | 0.81 | 0.08 | 3.65 | 0.00 |
| TRINITY_DN96621_c5_g1_i3 | | 27.71 | 105.85 | -1.92 | 0.00 |
| TRINITY_DN96623_c0_g1_i2 | CO1A1 | 335.14 | 168.37 | 1.05 | 0.00 |
| TRINITY_DN96644_c4_g2_i2 | | 2.36 | 10.69 | -2.20 | 0.00 |
| TRINITY_DN96645_c13_g1_i2 | | 5.83 | 25.11 | -2.17 | 0.00 |
| TRINITY_DN96650_c1_g1_i1 | | 2.54 | 14.05 | -2.51 | 0.00 |
| TRINITY_DN96671_c16_g1_i1 | | 2.03 | 6.59 | -1.77 | 0.00 |
| TRINITY_DN96671_c18_g1_i1 | | 23.50 | 10.70 | 1.11 | 0.00 |
| TRINITY_DN96679_c3_g3_i1 | | 18.62 | 81.47 | -2.07 | 0.00 |
| TRINITY_DN96684_c0_g1_i1 | | 78.80 | 37.67 | 1.09 | 0.00 |
| TRINITY_DN96688_c14_g3_i2 | TC1A | 1.42 | 7.15 | -2.38 | 0.00 |
| TRINITY_DN96689_c5_g1_i1 | | 0.18 | 5.42 | -4.22 | 0.00 |
| TRINITY_DN96698_c3_g1_i3 | | 0.21 | 2.72 | -3.72 | 0.00 |
| TRINITY_DN96698_c3_g1_i5 | | 1.68 | 11.83 | -2.85 | 0.00 |

| | | | | | |
|---|---|---|---|---|---|
| TRINITY_DN96707_c7_g1_i2 | | 7.71 | 14.44 | -0.90 | 0.00 |
| TRINITY_DN96708_c4_g1_i1 | GPR82 | 0.66 | 2.39 | -1.90 | 0.00 |
| TRINITY_DN96710_c4_g1_i2 | | 0.08 | 1.15 | -3.27 | 0.00 |
| TRINITY_DN96710_c5_g2_i3 | | 5.55 | 9.94 | -0.88 | 0.00 |
| TRINITY_DN96712_c0_g1_i1 | FZD2 | 9.32 | 4.95 | 0.94 | 0.00 |
| TRINITY_DN96717_c4_g2_i1 | | 7.45 | 4.14 | 0.82 | 0.00 |
| TRINITY_DN96730_c1_g3_i13 | RUFY3 | 0.00 | 1.18 | -5.50 | 0.00 |
| TRINITY_DN96740_c9_g2_i4 | VISTA | 4.98 | 17.04 | -1.83 | 0.00 |
| TRINITY_DN96747_c1_g1_i11 | | 0.00 | 1.12 | -5.20 | 0.00 |
| TRINITY_DN96747_c1_g1_i4 | | 3.29 | 13.12 | -1.88 | 0.00 |
| TRINITY_DN96747_c1_g2_i2 | | 2.26 | 18.58 | -2.89 | 0.00 |
| TRINITY_DN96747_c1_g2_i5 | | 4.70 | 18.28 | -1.94 | 0.00 |
| TRINITY_DN96769_c3_g1_i1 | | 0.66 | 4.25 | -2.54 | 0.00 |
| TRINITY_DN96769_c3_g1_i4 | | 0.38 | 2.13 | -2.40 | 0.00 |
| TRINITY_DN96769_c4_g1_i2 | | 9.38 | 3.96 | 1.25 | 0.00 |
| TRINITY_DN96770_c0_g2_i4 | | 0.07 | 1.98 | -4.13 | 0.00 |
| TRINITY_DN96778_c4_g3_i3 | LBP | 11.04 | 31.16 | -1.53 | 0.00 |
| TRINITY_DN96793_c3_g1_i3 | | 0.00 | 3.66 | -7.31 | 0.00 |
| TRINITY_DN96800_c3_g1_i1 | FLVC2 | 0.18 | 0.84 | -2.30 | 0.00 |
| TRINITY_DN96805_c4_g1_i2 | | 0.14 | 2.97 | -4.42 | 0.00 |
| TRINITY_DN96805_c4_g1_i3 | IFI44 | 28.82 | 265.12 | -3.21 | 0.00 |
| TRINITY_DN96805_c4_g1_i4 | IFI44 | 0.00 | 4.30 | -6.13 | 0.00 |
| TRINITY_DN96805_c4_g1_i6 | IFI44 | 5.94 | 163.71 | -4.76 | 0.00 |
| TRINITY_DN96811_c4_g1_i3 | | 25.12 | 10.90 | 1.23 | 0.00 |
| TRINITY_DN96811_c4_g2_i1 | | 11.38 | 5.81 | 0.95 | 0.00 |
| TRINITY_DN96866_c4_g1_i1 | CO1A1 | 149.61 | 68.75 | 1.16 | 0.00 |
| TRINITY_DN96883_c11_g1_i4 | ASC | 17.64 | 37.48 | -1.10 | 0.00 |
| TRINITY_DN96898_c4_g3_i5 | ODPA | 0.03 | 1.62 | -4.98 | 0.00 |
| TRINITY_DN96903_c12_g2_i2 | | 1.62 | 5.50 | -1.77 | 0.00 |
| TRINITY_DN96907_c0_g1_i6 | SEM7A | 0.83 | 4.29 | -2.40 | 0.00 |
| TRINITY_DN96914_c8_g4_i3 | I12R2 | 1.11 | 3.67 | -1.74 | 0.00 |
| TRINITY_DN96919_c15_g1_i1 | | 10.69 | 70.49 | -2.77 | 0.00 |
| TRINITY_DN96920_c0_g2_i2 | | 0.09 | 5.12 | -4.37 | 0.00 |
| TRINITY_DN96920_c0_g2_i9 | | 0.07 | 4.57 | -5.29 | 0.00 |
| TRINITY_DN96933_c3_g1_i1 | | 0.00 | 1.82 | -4.67 | 0.00 |
| TRINITY_DN96933_c4_g1_i1 | FEV | 2.34 | 4.99 | -1.09 | 0.00 |
| TRINITY_DN96938_c0_g1_i1 | PNBA | 20.47 | 11.60 | 0.82 | 0.00 |
| TRINITY_DN96941_c5_g1_i5 | | 34.35 | 102.03 | -1.57 | 0.00 |
| TRINITY_DN96941_c5_g1_i6 | | 0.86 | 4.28 | -2.38 | 0.00 |
| TRINITY_DN96941_c6_g2_i3 | SPI1 | 2.94 | 6.98 | -1.28 | 0.00 |
| TRINITY_DN96941_c6_g2_i4 | SPI1 | 0.42 | 1.72 | -2.01 | 0.00 |
| TRINITY_DN96941_c6_g2_i9 | SPI1 | 0.99 | 4.34 | -2.10 | 0.00 |

| | | | | | |
|---|---|---|---|---|---|
| TRINITY_DN96956_c10_g1_i2 | | 0.31 | 11.35 | -4.24 | 0.00 |
| TRINITY_DN96968_c5_g5_i2 | IGSF3 | 5.10 | 19.05 | -1.93 | 0.00 |
| TRINITY_DN96981_c3_g1_i5 | EX3L1 | 0.00 | 1.94 | -5.96 | 0.00 |
| TRINITY_DN96982_c7_g7_i1 | | 0.61 | 1.80 | -1.55 | 0.00 |
| TRINITY_DN96984_c2_g1_i1 | | 1.36 | 3.99 | -1.57 | 0.00 |
| TRINITY_DN96996_c4_g3_i1 | | 0.00 | 6.69 | -4.84 | 0.00 |
| TRINITY_DN97005_c0_g1_i5 | I5P2 | 3.63 | 11.13 | -1.60 | 0.00 |
| TRINITY_DN97014_c2_g1_i6 | SFRP3 | 12.94 | 7.91 | 0.69 | 0.00 |
| TRINITY_DN97017_c0_g2_i13 | CAN2 | 0.84 | 4.26 | -2.24 | 0.00 |
| TRINITY_DN97018_c10_g1_i2 | DDR2 | 0.92 | 0.05 | 4.03 | 0.00 |
| TRINITY_DN97025_c0_g1_i1 | PGAM1 | 0.11 | 7.19 | -5.84 | 0.00 |
| TRINITY_DN97025_c0_g1_i3 | PGAM1 | 43.62 | 23.34 | 0.91 | 0.00 |
| TRINITY_DN97035_c2_g1_i1 | | 0.00 | 1.31 | -5.38 | 0.00 |
| TRINITY_DN97037_c0_g1_i1 | | 2.10 | 0.65 | 1.74 | 0.00 |
| TRINITY_DN97038_c0_g1_i3 | | 361.84 | 142.90 | 1.33 | 0.00 |
| TRINITY_DN97038_c0_g1_i4 | | 233.39 | 45.86 | 2.47 | 0.00 |
| TRINITY_DN97038_c1_g1_i5 | | 0.07 | 0.52 | -3.00 | 0.00 |
| TRINITY_DN97042_c1_g1_i1 | GIMA7 | 1.28 | 17.16 | -3.73 | 0.00 |
| TRINITY_DN97083_c10_g6_i1 | | 0.85 | 3.06 | -1.84 | 0.00 |
| TRINITY_DN97095_c0_g2_i5 | SIK2 | 3.20 | 7.60 | -1.25 | 0.00 |
| TRINITY_DN97116_c3_g1_i4 | TRAF2 | 0.24 | 2.57 | -3.47 | 0.00 |
| TRINITY_DN97117_c5_g1_i2 | | 0.53 | 3.99 | -3.15 | 0.00 |
| TRINITY_DN97124_c2_g2_i1 | GPX4 | 1.68 | 12.01 | -2.84 | 0.00 |
| TRINITY_DN97124_c2_g2_i2 | GPX4 | 1.05 | 6.74 | -2.73 | 0.00 |
| TRINITY_DN97126_c0_g1_i1 | FUCO | 38.95 | 54.22 | -0.51 | 0.00 |
| TRINITY_DN97130_c2_g1_i1 | | 1.24 | 5.11 | -2.10 | 0.00 |
| TRINITY_DN97135_c0_g2_i2 | CC50A | 2.31 | 4.76 | -1.06 | 0.00 |
| TRINITY_DN97136_c8_g1_i1 | PHYD1 | 0.95 | 8.58 | -3.22 | 0.00 |
| TRINITY_DN97151_c6_g1_i5 | GP1BB | 19.05 | 5.99 | 1.78 | 0.00 |
| TRINITY_DN97176_c0_g1_i11 | TESK2 | 0.00 | 2.38 | -6.33 | 0.00 |
| TRINITY_DN97179_c6_g2_i1 | | 1.92 | 12.34 | -2.50 | 0.00 |
| TRINITY_DN97180_c11_g2_i1 | CYTB | 24.45 | 35.91 | -0.58 | 0.00 |
| TRINITY_DN97198_c0_g2_i8 | CDC45 | 0.03 | 2.14 | -4.25 | 0.00 |
| TRINITY_DN97200_c0_g1_i1 | SPRC | 75.69 | 42.23 | 0.88 | 0.00 |
| TRINITY_DN97200_c0_g1_i7 | SPRC | 212.60 | 118.60 | 0.85 | 0.00 |
| TRINITY_DN97204_c11_g1_i2 | | 0.09 | 1.14 | -3.39 | 0.00 |
| TRINITY_DN97207_c1_g1_i1 | | 5.41 | 0.80 | 2.90 | 0.00 |
| TRINITY_DN97209_c2_g1_i3 | | 0.35 | 14.20 | -3.91 | 0.00 |
| TRINITY_DN97209_c2_g1_i8 | | 3.37 | 28.72 | -3.04 | 0.00 |
| TRINITY_DN97209_c2_g2_i1 | | 0.58 | 13.66 | -4.55 | 0.00 |
| TRINITY_DN97237_c0_g1_i1 | | 0.27 | 8.00 | -4.94 | 0.00 |
| TRINITY_DN97239_c2_g2_i2 | ELF3 | 8.98 | 23.48 | -1.42 | 0.00 |

| | | | | | |
|---|---|---|---|---|---|
| TRINITY_DN97239_c2_g3_i6 | EHF | 0.20 | 1.40 | -2.82 | 0.00 |
| TRINITY_DN97239_c2_g3_i9 | EHF | 2.75 | 14.75 | -2.40 | 0.00 |
| TRINITY_DN97249_c0_g2_i2 | RN126 | 1.83 | 0.23 | 2.91 | 0.00 |
| TRINITY_DN97253_c2_g2_i1 | | 4.22 | 21.80 | -2.41 | 0.00 |
| TRINITY_DN97253_c2_g3_i1 | | 1.99 | 15.70 | -2.94 | 0.00 |
| TRINITY_DN97254_c10_g1_i4 | LIMA1 | 1.63 | 0.00 | 4.74 | 0.00 |
| TRINITY_DN97254_c9_g1_i1 | | 0.90 | 5.90 | -2.89 | 0.00 |
| TRINITY_DN97266_c3_g1_i1 | | 18.06 | 113.76 | -2.65 | 0.00 |
| TRINITY_DN97276_c4_g1_i1 | | 1.16 | 4.94 | -2.15 | 0.00 |
| TRINITY_DN97276_c4_g3_i3 | IFI44 | 0.65 | 7.54 | -3.48 | 0.00 |
| TRINITY_DN97276_c4_g6_i2 | | 0.00 | 2.00 | -5.63 | 0.00 |
| TRINITY_DN97283_c5_g1_i2 | | 0.00 | 0.50 | -3.96 | 0.00 |
| TRINITY_DN97286_c2_g1_i1 | SYCC | 3.15 | 9.34 | -1.58 | 0.00 |
| TRINITY_DN97293_c8_g3_i1 | | 0.70 | 15.90 | -4.03 | 0.00 |
| TRINITY_DN97299_c2_g1_i1 | EOMES | 0.21 | 0.95 | -2.18 | 0.00 |
| TRINITY_DN97299_c3_g2_i2 | | 0.53 | 2.15 | -2.02 | 0.00 |
| TRINITY_DN97299_c3_g4_i2 | | 3.39 | 6.88 | -1.03 | 0.00 |
| TRINITY_DN97314_c0_g2_i8 | TNR14 | 0.00 | 0.41 | -4.45 | 0.00 |
| TRINITY_DN97343_c0_g1_i4 | TECTA | 0.02 | 2.16 | -6.45 | 0.00 |
| TRINITY_DN97344_c1_g1_i9 | PKH4B | 0.08 | 0.32 | -2.44 | 0.00 |
| TRINITY_DN97349_c1_g4_i1 | CATZ | 61.04 | 98.13 | -0.68 | 0.00 |
| TRINITY_DN97368_c9_g1_i1 | | 16.09 | 126.26 | -2.97 | 0.00 |
| TRINITY_DN97380_c2_g2_i2 | CK053 | 0.37 | 1.57 | -2.06 | 0.00 |
| TRINITY_DN97380_c2_g2_i4 | CK053 | 0.20 | 1.67 | -3.20 | 0.00 |
| TRINITY_DN97389_c9_g1_i1 | | 11.80 | 3.21 | 1.91 | 0.00 |
| TRINITY_DN97394_c2_g1_i3 | MCF2L | 0.45 | 2.18 | -2.25 | 0.00 |
| TRINITY_DN97395_c0_g1_i5 | RSSA | 0.00 | 9.39 | -5.51 | 0.00 |
| TRINITY_DN97398_c2_g2_i1 | | 0.40 | 19.21 | -5.33 | 0.00 |
| TRINITY_DN97406_c4_g1_i8 | GP183 | 0.67 | 3.90 | -2.52 | 0.00 |
| TRINITY_DN97420_c3_g1_i1 | KLF11 | 13.26 | 4.97 | 1.40 | 0.00 |
| TRINITY_DN97432_c2_g2_i1 | | 3.39 | 30.96 | -3.18 | 0.00 |
| TRINITY_DN97445_c3_g1_i3 | | 1.23 | 0.11 | 3.44 | 0.00 |
| TRINITY_DN97460_c1_g1_i6 | CCNG2 | 12.22 | 25.91 | -1.09 | 0.00 |
| TRINITY_DN97473_c0_g1_i1 | CFDP1 | 0.00 | 1.39 | -6.22 | 0.00 |
| TRINITY_DN97477_c0_g1_i4 | CYTIP | 0.43 | 2.23 | -2.32 | 0.00 |
| TRINITY_DN97485_c0_g1_i10 | PSME1 | 38.19 | 72.25 | -0.88 | 0.00 |
| TRINITY_DN97490_c0_g1_i3 | MARCS | 19.79 | 37.12 | -0.94 | 0.00 |
| TRINITY_DN97501_c6_g9_i2 | | 0.40 | 3.07 | -2.89 | 0.00 |
| TRINITY_DN97515_c5_g1_i1 | RHG27 | 1.78 | 5.09 | -1.52 | 0.00 |
| TRINITY_DN97524_c0_g1_i3 | CATS | 64.02 | 147.42 | -1.23 | 0.00 |
| TRINITY_DN97524_c0_g1_i4 | CATS | 5.48 | 14.75 | -1.44 | 0.00 |
| TRINITY_DN97530_c11_g1_i1 | | 2.72 | 32.05 | -3.54 | 0.00 |

| | | | | | |
|---|---|---|---|---|---|
| TRINITY_DN97530_c9_g1_i2 | | 23.28 | 8.54 | 1.41 | 0.00 |
| TRINITY_DN97536_c4_g1_i2 | | 0.89 | 11.06 | -3.42 | 0.00 |
| TRINITY_DN97536_c4_g4_i4 | | 0.37 | 1.96 | -2.50 | 0.00 |
| TRINITY_DN97538_c0_g1_i5 | I20RA | 0.00 | 1.43 | -6.28 | 0.00 |
| TRINITY_DN97570_c3_g1_i1 | | 0.00 | 3.24 | -7.01 | 0.00 |
| TRINITY_DN97579_c3_g1_i1 | | 1.10 | 0.22 | 2.37 | 0.00 |
| TRINITY_DN97587_c3_g1_i2 | RAD51 | 6.05 | 1.05 | 2.50 | 0.00 |
| TRINITY_DN97588_c4_g3_i1 | YTX2 | 0.63 | 5.67 | -2.90 | 0.00 |
| TRINITY_DN97596_c10_g1_i1 | | 1.76 | 0.00 | 5.99 | 0.00 |
| TRINITY_DN97596_c11_g1_i1 | | 0.30 | 3.09 | -3.37 | 0.00 |
| TRINITY_DN97604_c8_g1_i1 | DHE3 | 38.77 | 17.66 | 1.21 | 0.00 |
| TRINITY_DN97636_c0_g1_i1 | KPCD3 | 0.33 | 1.38 | -2.01 | 0.00 |
| TRINITY_DN97636_c0_g3_i2 | KPCD3 | 0.13 | 1.32 | -3.16 | 0.00 |
| TRINITY_DN97643_c6_g3_i6 | AGRF3 | 0.73 | 9.55 | -3.78 | 0.00 |
| TRINITY_DN97645_c1_g2_i7 | EMAL1 | 4.67 | 9.27 | -1.00 | 0.00 |
| TRINITY_DN97645_c1_g2_i9 | EMAL1 | 6.62 | 13.45 | -1.05 | 0.00 |
| TRINITY_DN97648_c0_g2_i1 | FERM1 | 4.87 | 2.91 | 0.73 | 0.00 |
| TRINITY_DN97663_c0_g1_i21 | | 0.08 | 1.13 | -3.65 | 0.00 |
| TRINITY_DN97671_c2_g1_i1 | RN122 | 11.90 | 8.01 | 0.55 | 0.00 |
| TRINITY_DN97672_c1_g3_i4 | | 0.90 | 2.75 | -1.66 | 0.00 |
| TRINITY_DN97676_c0_g1_i6 | MYO1F | 2.08 | 7.65 | -1.90 | 0.00 |
| TRINITY_DN97681_c0_g1_i1 | PODN | 4.20 | 1.78 | 1.24 | 0.00 |
| TRINITY_DN97691_c1_g2_i1 | | 2.89 | 6.48 | -1.16 | 0.00 |
| TRINITY_DN97695_c4_g3_i2 | | 0.00 | 4.39 | -5.07 | 0.00 |
| TRINITY_DN97699_c1_g1_i2 | | 3.40 | 23.41 | -2.81 | 0.00 |
| TRINITY_DN97724_c2_g2_i4 | KPRA | 0.00 | 5.14 | -4.87 | 0.00 |
| TRINITY_DN97728_c1_g4_i2 | | 1.56 | 0.00 | 6.35 | 0.00 |
| TRINITY_DN97748_c1_g1_i5 | Y1510 | 0.02 | 0.87 | -5.02 | 0.00 |
| TRINITY_DN97762_c4_g2_i5 | | 0.26 | 2.43 | -3.02 | 0.00 |
| TRINITY_DN97766_c6_g1_i3 | | 4.11 | 1.23 | 1.73 | 0.00 |
| TRINITY_DN97774_c3_g1_i2 | NHRF1 | 1.45 | 4.84 | -1.75 | 0.00 |
| TRINITY_DN97780_c6_g1_i1 | | 1.24 | 4.50 | -1.87 | 0.00 |
| TRINITY_DN97796_c3_g2_i1 | B2MG | 1594.12 | 4386.24 | -1.50 | 0.00 |
| TRINITY_DN97796_c3_g2_i5 | | 0.11 | 1.13 | -3.26 | 0.00 |
| TRINITY_DN97797_c0_g2_i4 | YTX2 | 0.13 | 0.87 | -2.75 | 0.00 |
| TRINITY_DN97807_c6_g1_i10 | TPSNR | 1.66 | 7.41 | -2.18 | 0.00 |
| TRINITY_DN97825_c5_g2_i1 | RORA | 0.13 | 4.38 | -3.81 | 0.00 |
| TRINITY_DN97828_c4_g1_i1 | | 0.19 | 1.29 | -2.63 | 0.00 |
| TRINITY_DN97829_c5_g1_i8 | | 25.33 | 63.93 | -1.34 | 0.00 |
| TRINITY_DN97890_c0_g1_i1 | DAPLE | 0.38 | 1.53 | -2.00 | 0.00 |
| TRINITY_DN97909_c9_g3_i8 | IGKC | 1.57 | 11.24 | -2.69 | 0.00 |
| TRINITY_DN97912_c1_g4_i7 | | 17.47 | 79.53 | -2.20 | 0.00 |

| | | | | | |
|---|---|---|---|---|---|
| TRINITY_DN97913_c0_g1_i1 | | 6.60 | 21.01 | -1.69 | 0.00 |
| TRINITY_DN97923_c1_g1_i3 | SSRP1 | 0.11 | 4.74 | -5.04 | 0.00 |
| TRINITY_DN97951_c7_g1_i1 | DIAC | 4.99 | 8.38 | -0.77 | 0.00 |
| TRINITY_DN97964_c4_g1_i2 | DTX1 | 0.24 | 1.35 | -2.38 | 0.00 |
| TRINITY_DN97964_c4_g3_i1 | DTX4 | 1.46 | 5.68 | -1.97 | 0.00 |
| TRINITY_DN97966_c1_g1_i2 | PPTC7 | 1.09 | 0.02 | 6.43 | 0.00 |
| TRINITY_DN97974_c10_g1_i1 | | 4.42 | 0.40 | 3.47 | 0.00 |
| TRINITY_DN97978_c0_g2_i2 | | 0.84 | 3.31 | -1.93 | 0.00 |
| TRINITY_DN97980_c2_g1_i1 | GIT2 | 1.93 | 3.84 | -1.01 | 0.00 |
| TRINITY_DN97988_c0_g1_i1 | FAK2 | 5.03 | 17.44 | -1.80 | 0.00 |
| TRINITY_DN97989_c1_g1_i1 | BSDC1 | 0.15 | 3.45 | -4.32 | 0.00 |
| TRINITY_DN98001_c0_g2_i1 | | 2.66 | 0.57 | 2.33 | 0.00 |
| TRINITY_DN98009_c5_g1_i4 | | 1.17 | 20.19 | -3.99 | 0.00 |
| TRINITY_DN98009_c5_g1_i8 | | 2.31 | 23.16 | -3.27 | 0.00 |
| TRINITY_DN98015_c2_g1_i3 | SEM4B | 2.47 | 4.78 | -0.97 | 0.00 |
| TRINITY_DN98022_c0_g1_i3 | RPGR | 0.59 | 0.00 | 5.23 | 0.00 |
| TRINITY_DN98022_c0_g7_i4 | SRPX | 12.41 | 6.01 | 1.07 | 0.00 |
| TRINITY_DN98051_c0_g1_i7 | | 0.14 | 2.97 | -4.26 | 0.00 |
| TRINITY_DN98051_c0_g2_i13 | | 0.24 | 4.20 | -3.97 | 0.00 |
| TRINITY_DN98051_c0_g2_i2 | | 0.24 | 2.48 | -3.44 | 0.00 |
| TRINITY_DN98051_c0_g2_i5 | GIMA4 | 0.59 | 3.42 | -2.57 | 0.00 |
| TRINITY_DN98053_c0_g1_i1 | | 0.00 | 1.91 | -5.61 | 0.00 |
| TRINITY_DN98058_c0_g2_i1 | SPP2A | 0.00 | 4.12 | -7.52 | 0.00 |
| TRINITY_DN98058_c0_g2_i2 | SPP2A | 0.00 | 6.73 | -8.20 | 0.00 |
| TRINITY_DN98058_c0_g2_i6 | SPP2A | 14.99 | 20.93 | -0.52 | 0.00 |
| TRINITY_DN98060_c0_g2_i1 | | 1.75 | 13.58 | -2.86 | 0.00 |
| TRINITY_DN98070_c4_g3_i1 | | 0.50 | 3.91 | -2.91 | 0.00 |
| TRINITY_DN98078_c2_g2_i2 | | 0.61 | 8.36 | -3.69 | 0.00 |
| TRINITY_DN98081_c3_g1_i3 | N4BP3 | 0.76 | 5.48 | -2.90 | 0.00 |
| TRINITY_DN98083_c0_g1_i4 | TCPE | 0.40 | 2.79 | -2.83 | 0.00 |
| TRINITY_DN98093_c2_g1_i1 | I10R2 | 2.58 | 10.13 | -2.02 | 0.00 |
| TRINITY_DN98097_c1_g1_i5 | B3GN2 | 2.96 | 1.09 | 1.44 | 0.00 |
| TRINITY_DN98109_c7_g1_i1 | | 0.17 | 1.51 | -3.05 | 0.00 |
| TRINITY_DN98136_c1_g1_i11 | | 0.89 | 8.33 | -3.18 | 0.00 |
| TRINITY_DN98172_c4_g2_i3 | | 1.41 | 0.39 | 1.86 | 0.00 |
| TRINITY_DN98193_c5_g1_i5 | | 16.22 | 51.69 | -1.68 | 0.00 |
| TRINITY_DN98199_c0_g1_i1 | IRF2 | 9.75 | 23.88 | -1.34 | 0.00 |
| TRINITY_DN98199_c0_g1_i15 | IRF1 | 36.98 | 151.01 | -2.07 | 0.00 |
| TRINITY_DN98199_c0_g1_i4 | IRF2 | 0.74 | 1.68 | -1.20 | 0.00 |
| TRINITY_DN98199_c0_g1_i6 | IRF2 | 2.68 | 6.31 | -1.26 | 0.00 |
| TRINITY_DN98203_c9_g1_i2 | UBS3B | 0.56 | 1.97 | -1.78 | 0.00 |
| TRINITY_DN98212_c5_g1_i1 | ITB2 | 1.30 | 14.16 | -3.46 | 0.00 |

| | | | | | |
|---|---|---|---|---|---|
| TRINITY_DN98250_c0_g1_i2 | FOXA2 | 0.35 | 1.73 | -2.28 | 0.00 |
| TRINITY_DN98253_c2_g1_i1 | C1QC | 132.31 | 398.95 | -1.64 | 0.00 |
| TRINITY_DN98254_c2_g1_i3 | MEFV | 0.03 | 1.04 | -4.45 | 0.00 |
| TRINITY_DN98254_c2_g1_i4 | TRI39 | 2.07 | 18.23 | -3.16 | 0.00 |
| TRINITY_DN98278_c3_g2_i2 | | 1.60 | 5.36 | -1.79 | 0.00 |
| TRINITY_DN98279_c0_g2_i1 | CO1A1 | 218.48 | 105.74 | 1.10 | 0.00 |
| TRINITY_DN98279_c0_g2_i2 | CO1A1 | 176.23 | 93.53 | 0.97 | 0.00 |
| TRINITY_DN98306_c5_g1_i11 | | 11.46 | 0.40 | 4.61 | 0.00 |
| TRINITY_DN98310_c2_g1_i3 | TT39B | 2.14 | 9.77 | -2.12 | 0.00 |
| TRINITY_DN98318_c5_g1_i3 | UHRF2 | 0.00 | 2.36 | -4.48 | 0.00 |
| TRINITY_DN98323_c7_g1_i5 | | 1.23 | 3.12 | -1.35 | 0.00 |
| TRINITY_DN98328_c3_g1_i2 | MOES | 6.27 | 11.31 | -0.84 | 0.00 |
| TRINITY_DN98329_c2_g1_i10 | LRRC1 | 0.49 | 0.00 | 5.51 | 0.00 |
| TRINITY_DN98335_c1_g1_i5 | | 2.07 | 18.37 | -3.10 | 0.00 |
| TRINITY_DN98341_c4_g1_i2 | | 23.42 | 8.60 | 1.51 | 0.00 |
| TRINITY_DN98341_c4_g3_i5 | | 2.65 | 0.00 | 6.66 | 0.00 |
| TRINITY_DN98341_c5_g4_i1 | | 0.62 | 2.33 | -1.88 | 0.00 |
| TRINITY_DN98347_c10_g1_i1 | FZD7B | 15.01 | 8.96 | 0.72 | 0.00 |
| TRINITY_DN98359_c5_g4_i1 | | 4.52 | 13.54 | -1.64 | 0.00 |
| TRINITY_DN98361_c4_g1_i4 | | 0.00 | 5.58 | -4.22 | 0.00 |
| TRINITY_DN98367_c0_g2_i10 | S10A1 | 0.83 | 111.22 | -7.07 | 0.00 |
| TRINITY_DN98367_c0_g2_i13 | S10AA | 26.25 | 10.29 | 1.39 | 0.00 |
| TRINITY_DN98367_c0_g2_i14 | S10A1 | 83.46 | 27.77 | 1.63 | 0.00 |
| TRINITY_DN98367_c0_g2_i4 | S10A1 | 473.26 | 224.98 | 1.12 | 0.00 |
| TRINITY_DN98367_c0_g2_i5 | S10A1 | 0.00 | 7.22 | -6.24 | 0.00 |
| TRINITY_DN98367_c0_g2_i7 | S10A1 | 0.11 | 19.33 | -6.87 | 0.00 |
| TRINITY_DN98378_c0_g2_i1 | | 17.06 | 5.35 | 1.68 | 0.00 |
| TRINITY_DN98381_c3_g1_i1 | PGBD4 | 0.05 | 0.64 | -3.52 | 0.00 |
| TRINITY_DN98404_c0_g2_i8 | TIM50 | 5.08 | 2.29 | 1.18 | 0.00 |
| TRINITY_DN98405_c9_g2_i3 | CD79B | 0.67 | 2.96 | -2.20 | 0.00 |
| TRINITY_DN98414_c4_g5_i2 | | 0.63 | 1.99 | -1.66 | 0.00 |
| TRINITY_DN98434_c0_g2_i3 | NCKPL | 0.58 | 3.94 | -2.75 | 0.00 |
| TRINITY_DN98438_c0_g2_i1 | IRAK3 | 1.28 | 4.97 | -1.98 | 0.00 |
| TRINITY_DN98438_c0_g6_i1 | | 0.81 | 10.10 | -3.14 | 0.00 |
| TRINITY_DN98444_c2_g1_i10 | | 0.50 | 0.00 | 5.25 | 0.00 |
| TRINITY_DN98448_c4_g1_i1 | CLD23 | 26.70 | 40.34 | -0.62 | 0.00 |
| TRINITY_DN98460_c0_g3_i4 | RIPK3 | 6.01 | 14.05 | -1.23 | 0.00 |
| TRINITY_DN98462_c1_g1_i6 | LIMS1 | 9.78 | 4.98 | 1.00 | 0.00 |
| TRINITY_DN98483_c2_g1_i6 | CNTRL | 0.47 | 0.04 | 3.63 | 0.00 |
| TRINITY_DN98494_c2_g2_i1 | PO2F3 | 0.62 | 3.05 | -2.28 | 0.00 |
| TRINITY_DN98507_c3_g1_i4 | | 4.09 | 25.76 | -2.71 | 0.00 |
| TRINITY_DN98507_c3_g1_i5 | | 10.14 | 37.81 | -1.89 | 0.00 |

| | | | | | |
|---|---|---|---|---|---|
| TRINITY_DN98512_c0_g1_i3 | AGAP1 | 0.99 | 3.58 | -1.86 | 0.00 |
| TRINITY_DN98513_c0_g1_i6 | THMS1 | 0.07 | 1.04 | -4.02 | 0.00 |
| TRINITY_DN98513_c0_g1_i7 | THMS1 | 0.91 | 3.14 | -1.80 | 0.00 |
| TRINITY_DN98524_c9_g1_i1 | | 1.05 | 7.85 | -2.97 | 0.00 |
| TRINITY_DN98526_c0_g1_i1 | SOX8 | 3.47 | 11.43 | -1.73 | 0.00 |
| TRINITY_DN98531_c1_g4_i1 | | 0.08 | 1.07 | -3.33 | 0.00 |
| TRINITY_DN98537_c1_g1_i1 | | 0.00 | 18.68 | -4.76 | 0.00 |
| TRINITY_DN98545_c3_g1_i1 | NFKB2 | 6.54 | 15.93 | -1.31 | 0.00 |
| TRINITY_DN98547_c0_g1_i1 | PTPRH | 0.46 | 2.39 | -2.38 | 0.00 |
| TRINITY_DN98547_c0_g1_i2 | TENA | 0.58 | 4.72 | -3.04 | 0.00 |
| TRINITY_DN98547_c1_g3_i4 | IFM3 | 18.56 | 50.32 | -1.50 | 0.00 |
| TRINITY_DN98550_c3_g2_i12 | SOX13 | 1.17 | 7.66 | -2.75 | 0.00 |
| TRINITY_DN98554_c2_g1_i1 | SH21A | 2.18 | 9.20 | -2.10 | 0.00 |
| TRINITY_DN98557_c2_g1_i1 | CD276 | 2.56 | 0.00 | 7.27 | 0.00 |
| TRINITY_DN98575_c7_g1_i1 | CK096 | 4.18 | 1.71 | 1.29 | 0.00 |
| TRINITY_DN98579_c0_g1_i3 | LAG3 | 0.12 | 2.33 | -4.15 | 0.00 |
| TRINITY_DN98597_c4_g1_i1 | CD22 | 0.52 | 3.52 | -2.81 | 0.00 |
| TRINITY_DN98597_c4_g1_i14 | CD22 | 0.05 | 0.70 | -4.17 | 0.00 |
| TRINITY_DN98597_c4_g1_i16 | CD22 | 0.05 | 1.06 | -4.32 | 0.00 |
| TRINITY_DN98597_c4_g1_i2 | CD22 | 0.08 | 1.66 | -4.24 | 0.00 |
| TRINITY_DN98597_c4_g1_i5 | CD22 | 0.00 | 0.65 | -4.94 | 0.00 |
| TRINITY_DN98600_c2_g3_i1 | TNIP2 | 1.35 | 3.56 | -1.39 | 0.00 |
| TRINITY_DN98612_c1_g3_i1 | | 0.84 | 7.94 | -2.79 | 0.00 |
| TRINITY_DN98612_c2_g1_i2 | KPCT | 0.00 | 1.27 | -4.60 | 0.00 |
| TRINITY_DN98615_c0_g1_i1 | MACF1 | 1.20 | 1.09 | -1.46 | 0.00 |
| TRINITY_DN98636_c11_g1_i1 | CD22 | 0.00 | 1.18 | -5.75 | 0.00 |
| TRINITY_DN98638_c12_g4_i1 | | 0.00 | 5.39 | -4.34 | 0.00 |
| TRINITY_DN98658_c11_g1_i3 | CFAD | 2.62 | 6.82 | -1.39 | 0.00 |
| TRINITY_DN98663_c2_g4_i1 | NECT3 | 1.65 | 5.36 | -1.74 | 0.00 |
| TRINITY_DN98693_c0_g1_i7 | | 0.04 | 0.75 | -4.50 | 0.00 |
| TRINITY_DN98695_c0_g1_i4 | LRC8D | 0.03 | 0.27 | -3.59 | 0.00 |
| TRINITY_DN98698_c1_g2_i4 | KHDR1 | 0.32 | 1.28 | -2.02 | 0.00 |
| TRINITY_DN98716_c1_g1_i2 | GIMA7 | 1.51 | 5.51 | -1.89 | 0.00 |
| TRINITY_DN98727_c1_g1_i2 | | 2.15 | 5.20 | -1.23 | 0.00 |
| TRINITY_DN98735_c4_g1_i1 | | 12.81 | 0.76 | 4.01 | 0.00 |
| TRINITY_DN98739_c4_g1_i1 | SPI1 | 1.04 | 2.56 | -1.31 | 0.00 |
| TRINITY_DN98739_c4_g1_i3 | SPI1 | 3.37 | 11.96 | -1.86 | 0.00 |
| TRINITY_DN98762_c3_g3_i3 | | 1.11 | 6.43 | -2.42 | 0.00 |
| TRINITY_DN98767_c6_g2_i1 | CENPW | 0.24 | 4.73 | -4.43 | 0.00 |
| TRINITY_DN98784_c7_g1_i1 | LPXN | 6.29 | 12.78 | -1.04 | 0.00 |
| TRINITY_DN98805_c1_g6_i1 | | 1.97 | 7.92 | -1.99 | 0.00 |
| TRINITY_DN98814_c1_g1_i11 | | 0.00 | 4.01 | -6.09 | 0.00 |

| | | | | | |
|---|---|---|---|---|---|
| TRINITY_DN98814_c1_g1_i2 | SOCS1 | 0.00 | 2.32 | -5.51 | 0.00 |
| TRINITY_DN98814_c1_g1_i6 | SOCS1 | 0.00 | 1.99 | -4.79 | 0.00 |
| TRINITY_DN98816_c1_g2_i3 | ABR | 0.34 | 1.71 | -2.32 | 0.00 |
| TRINITY_DN98829_c4_g2_i2 | | 3.09 | 13.52 | -2.24 | 0.00 |
| TRINITY_DN98834_c4_g2_i1 | | 52.43 | 21.75 | 1.21 | 0.00 |
| TRINITY_DN98834_c4_g2_i2 | | 63.41 | 28.95 | 1.11 | 0.00 |
| TRINITY_DN98836_c3_g1_i1 | | 543.10 | 1472.43 | -1.49 | 0.00 |
| TRINITY_DN98836_c3_g2_i1 | | 17.42 | 55.67 | -1.73 | 0.00 |
| TRINITY_DN98842_c0_g2_i3 | CD2 | 1.16 | 5.45 | -2.22 | 0.00 |
| TRINITY_DN98844_c0_g1_i9 | AZI2 | 0.56 | 4.74 | -3.19 | 0.00 |
| TRINITY_DN98865_c3_g1_i2 | VEGFD | 1.15 | 0.02 | 4.78 | 0.00 |
| TRINITY_DN98868_c5_g1_i2 | | 12.87 | 0.51 | 4.72 | 0.00 |
| TRINITY_DN98877_c3_g1_i1 | DYLT3 | 1.59 | 0.00 | 5.85 | 0.00 |
| TRINITY_DN98884_c4_g1_i6 | AN32B | 11.56 | 1.77 | 2.67 | 0.00 |
| TRINITY_DN98904_c2_g1_i1 | | 0.00 | 1.72 | -5.39 | 0.00 |
| TRINITY_DN98906_c6_g1_i3 | | 0.06 | 2.11 | -4.89 | 0.00 |
| TRINITY_DN98906_c6_g1_i9 | | 3.35 | 17.79 | -2.32 | 0.00 |
| TRINITY_DN98907_c0_g1_i2 | | 0.00 | 11.41 | -4.96 | 0.00 |
| TRINITY_DN98913_c1_g1_i7 | | 0.00 | 1.42 | -5.37 | 0.00 |
| TRINITY_DN98913_c1_g1_i9 | | 0.32 | 2.27 | -2.91 | 0.00 |
| TRINITY_DN98924_c3_g1_i1 | | 0.12 | 1.65 | -3.55 | 0.00 |
| TRINITY_DN98939_c0_g1_i11 | PAMR1 | 0.65 | 0.02 | 4.81 | 0.00 |
| TRINITY_DN98960_c2_g1_i11 | PPIP1 | 2.39 | 6.53 | -1.48 | 0.00 |
| TRINITY_DN98960_c2_g1_i9 | PPIP1 | 0.31 | 1.62 | -2.38 | 0.00 |
| TRINITY_DN98960_c2_g2_i1 | PPIP1 | 3.17 | 11.16 | -1.95 | 0.00 |
| TRINITY_DN98967_c2_g2_i2 | ACSL1 | 0.98 | 2.86 | -1.61 | 0.00 |
| TRINITY_DN98973_c16_g1_i1 | | 7.67 | 25.04 | -1.70 | 0.00 |
| TRINITY_DN99000_c3_g2_i5 | | 0.26 | 2.33 | -3.07 | 0.00 |
| TRINITY_DN99010_c3_g1_i1 | | 0.04 | 1.99 | -4.22 | 0.00 |
| TRINITY_DN99019_c5_g1_i2 | VASP | 4.24 | 14.80 | -1.88 | 0.00 |
| TRINITY_DN99021_c3_g1_i1 | MYADM | 0.22 | 1.35 | -2.70 | 0.00 |
| TRINITY_DN99035_c6_g1_i4 | CD53 | 1.56 | 7.64 | -2.33 | 0.00 |
| TRINITY_DN99035_c6_g2_i4 | CD53 | 9.52 | 25.22 | -1.43 | 0.00 |
| TRINITY_DN99037_c1_g1_i3 | RAB35 | 0.00 | 0.88 | -4.80 | 0.00 |
| TRINITY_DN99061_c4_g1_i1 | | 5.94 | 52.93 | -3.20 | 0.00 |
| TRINITY_DN99064_c0_g1_i4 | MRC1 | 0.28 | 2.96 | -3.44 | 0.00 |
| TRINITY_DN99073_c3_g3_i2 | | 2.77 | 66.09 | -4.57 | 0.00 |
| TRINITY_DN99073_c3_g4_i1 | | 0.18 | 6.90 | -5.41 | 0.00 |
| TRINITY_DN99073_c3_g4_i3 | | 0.55 | 10.14 | -4.26 | 0.00 |
| TRINITY_DN99073_c3_g4_i4 | | 0.86 | 20.56 | -4.65 | 0.00 |
| TRINITY_DN99080_c3_g1_i2 | | 2.45 | 0.99 | 1.29 | 0.00 |
| TRINITY_DN99083_c0_g1_i4 | | 0.33 | 5.59 | -3.98 | 0.00 |

| | | | | | |
|---|---|---|---|---|---|
| TRINITY_DN99084_c0_g1_i1 | | 1.49 | 6.42 | -2.10 | 0.00 |
| TRINITY_DN99096_c0_g2_i12 | ZC12A | 0.82 | 3.83 | -2.24 | 0.00 |
| TRINITY_DN99096_c0_g2_i2 | ZC12A | 0.39 | 2.91 | -2.95 | 0.00 |
| TRINITY_DN99098_c0_g1_i17 | | 1.35 | 9.39 | -2.74 | 0.00 |
| TRINITY_DN99099_c0_g1_i1 | TACC3 | 1.62 | 0.00 | 7.05 | 0.00 |
| TRINITY_DN99102_c8_g8_i1 | | 1.17 | 5.66 | -2.29 | 0.00 |
| TRINITY_DN99122_c8_g3_i1 | PPCE | 0.31 | 2.86 | -2.98 | 0.00 |
| TRINITY_DN99129_c0_g1_i1 | | 0.00 | 1.02 | -5.25 | 0.00 |
| TRINITY_DN99129_c0_g1_i2 | | 7.71 | 3.54 | 1.15 | 0.00 |
| TRINITY_DN99129_c0_g1_i3 | | 5.81 | 1.84 | 1.70 | 0.00 |
| TRINITY_DN99130_c0_g2_i3 | FSTL1 | 12.12 | 4.03 | 1.59 | 0.00 |
| TRINITY_DN99136_c12_g1_i1 | | 15.57 | 3.32 | 2.18 | 0.00 |
| TRINITY_DN99166_c3_g1_i1 | | 0.28 | 4.95 | -3.92 | 0.00 |
| TRINITY_DN99178_c0_g1_i13 | BAZ2B | 0.00 | 0.86 | -6.09 | 0.00 |
| TRINITY_DN99185_c4_g3_i1 | | 1.18 | 21.69 | -4.02 | 0.00 |
| TRINITY_DN99206_c2_g1_i3 | GIMA4 | 0.10 | 1.72 | -3.75 | 0.00 |
| TRINITY_DN99217_c0_g1_i4 | I20L2 | 0.00 | 2.73 | -6.50 | 0.00 |
| TRINITY_DN99224_c0_g1_i15 | PLD4 | 2.48 | 6.31 | -1.36 | 0.00 |
| TRINITY_DN99238_c2_g1_i1 | | 0.21 | 4.55 | -4.55 | 0.00 |
| TRINITY_DN99259_c3_g2_i1 | | 3.56 | 7.86 | -1.13 | 0.00 |
| TRINITY_DN99260_c0_g3_i1 | IBP5 | 1.37 | 0.00 | 6.03 | 0.00 |
| TRINITY_DN99266_c3_g1_i3 | | 2.47 | 109.16 | -5.32 | 0.00 |
| TRINITY_DN99266_c3_g1_i6 | | 0.14 | 5.17 | -4.77 | 0.00 |
| TRINITY_DN99268_c1_g1_i2 | SDHA | 4.38 | 0.68 | 2.76 | 0.00 |
| TRINITY_DN99283_c4_g1_i5 | STXB | 18.25 | 39.20 | -1.09 | 0.00 |
| TRINITY_DN99291_c0_g1_i4 | LS12A | 11.92 | 8.21 | 0.53 | 0.00 |
| TRINITY_DN99298_c0_g1_i1 | SH3L1 | 19.97 | 32.73 | -0.72 | 0.00 |
| TRINITY_DN99308_c1_g1_i4 | ZMAT5 | 3.13 | 0.16 | 4.55 | 0.00 |
| TRINITY_DN99308_c1_g1_i5 | ZMAT5 | 1.87 | 0.09 | 4.47 | 0.00 |
| TRINITY_DN99308_c1_g3_i1 | | 2.88 | 0.44 | 2.67 | 0.00 |
| TRINITY_DN99312_c0_g1_i2 | TRAF3 | 0.11 | 0.96 | -2.98 | 0.00 |
| TRINITY_DN99313_c0_g1_i2 | | 0.18 | 11.59 | -3.72 | 0.00 |
| TRINITY_DN99315_c5_g1_i1 | CCD50 | 2.34 | 0.08 | 4.80 | 0.00 |
| TRINITY_DN99316_c6_g1_i1 | ASPP2 | 0.37 | 1.77 | -2.09 | 0.00 |
| TRINITY_DN99346_c3_g2_i2 | LAMC2 | 0.83 | 0.20 | 2.01 | 0.00 |
| TRINITY_DN99352_c11_g1_i1 | | 1.70 | 3.55 | -1.06 | 0.00 |
| TRINITY_DN99360_c1_g1_i1 | CO1A2 | 140.78 | 62.55 | 1.27 | 0.00 |
| TRINITY_DN99360_c1_g1_i2 | CO1A2 | 99.06 | 51.44 | 1.03 | 0.00 |
| TRINITY_DN99362_c2_g1_i7 | CYH1 | 0.70 | 6.14 | -3.07 | 0.00 |
| TRINITY_DN99363_c2_g1_i4 | EPCAM | 15.41 | 37.73 | -1.36 | 0.00 |
| TRINITY_DN99393_c6_g4_i3 | PAR14 | 4.44 | 9.42 | -1.07 | 0.00 |
| TRINITY_DN99409_c12_g6_i1 | | 18.65 | 8.63 | 1.17 | 0.00 |

| | | | | | |
|---|---|---|---|---|---|
| TRINITY_DN99413_c2_g3_i1 | SN | 0.08 | 1.23 | -3.64 | 0.00 |
| TRINITY_DN99446_c2_g2_i1 | | 12.49 | 31.25 | -1.41 | 0.00 |
| TRINITY_DN99455_c3_g1_i1 | | 17.74 | 6.37 | 1.60 | 0.00 |
| TRINITY_DN99460_c0_g2_i3 | PCOC1 | 31.03 | 17.07 | 0.85 | 0.00 |
| TRINITY_DN99470_c5_g2_i1 | | 3.12 | 10.08 | -1.72 | 0.00 |
| TRINITY_DN99470_c5_g2_i2 | VASP | 46.21 | 182.97 | -1.98 | 0.00 |
| TRINITY_DN99473_c0_g1_i9 | QCR1 | 5.42 | 0.04 | 6.78 | 0.00 |
| TRINITY_DN99474_c6_g2_i2 | PK3CD | 0.51 | 2.33 | -2.18 | 0.00 |
| TRINITY_DN99481_c6_g1_i3 | | 0.00 | 6.55 | -5.44 | 0.00 |
| TRINITY_DN99510_c0_g1_i2 | S40A1 | 0.80 | 2.41 | -1.58 | 0.00 |
| TRINITY_DN99515_c5_g1_i3 | | 7.28 | 12.90 | -0.82 | 0.00 |
| TRINITY_DN99530_c4_g2_i8 | | 2.56 | 6.09 | -1.25 | 0.00 |
| TRINITY_DN99532_c2_g1_i5 | | 28.25 | 77.34 | -1.46 | 0.00 |
| TRINITY_DN99548_c3_g1_i3 | PRS27 | 284.16 | 45.61 | 2.56 | 0.00 |
| TRINITY_DN99556_c2_g1_i1 | | 29.41 | 13.00 | 1.24 | 0.00 |
| TRINITY_DN99556_c9_g1_i1 | | 0.00 | 1.63 | -4.73 | 0.00 |
| TRINITY_DN99568_c4_g1_i1 | CO2A1 | 7.17 | 0.53 | 3.74 | 0.00 |
| TRINITY_DN99568_c5_g5_i6 | FA49A | 1.06 | 4.82 | -2.17 | 0.00 |
| TRINITY_DN99568_c6_g1_i1 | S10A1 | 0.36 | 3.51 | -3.30 | 0.00 |
| TRINITY_DN99578_c0_g1_i2 | | 3.08 | 10.06 | -1.72 | 0.00 |
| TRINITY_DN99578_c1_g1_i2 | ACTP1 | 123.19 | 67.77 | 0.85 | 0.00 |
| TRINITY_DN99586_c2_g1_i1 | | 77.95 | 41.67 | 0.92 | 0.00 |
| TRINITY_DN99588_c1_g3_i1 | | 2.18 | 3.90 | -0.86 | 0.00 |
| TRINITY_DN99608_c2_g1_i1 | | 0.11 | 1.22 | -3.14 | 0.00 |
| TRINITY_DN99623_c3_g1_i3 | | 2.90 | 0.00 | 5.94 | 0.00 |
| TRINITY_DN99629_c3_g4_i2 | | 0.00 | 8.36 | -6.44 | 0.00 |
| TRINITY_DN99644_c2_g1_i1 | | 0.32 | 4.65 | -3.19 | 0.00 |
| TRINITY_DN99651_c1_g2_i1 | ADH1 | 19.58 | 9.16 | 1.11 | 0.00 |
| TRINITY_DN99655_c1_g1_i1 | | 2.33 | 4.34 | -0.92 | 0.00 |
| TRINITY_DN99655_c1_g3_i1 | | 3.36 | 6.08 | -0.88 | 0.00 |
| TRINITY_DN99666_c1_g1_i2 | FGFR4 | 5.20 | 2.58 | 1.03 | 0.00 |
| TRINITY_DN99668_c0_g1_i1 | MEFV | 0.85 | 10.43 | -3.57 | 0.00 |
| TRINITY_DN99668_c0_g3_i2 | ERMAP | 0.37 | 6.08 | -3.91 | 0.00 |
| TRINITY_DN99676_c3_g2_i1 | | 0.14 | 1.66 | -3.85 | 0.00 |
| TRINITY_DN99676_c3_g2_i3 | | 0.67 | 4.33 | -2.63 | 0.00 |
| TRINITY_DN99677_c0_g1_i1 | GPC6 | 3.59 | 1.94 | 0.89 | 0.00 |
| TRINITY_DN99678_c1_g2_i3 | PPIF | 0.18 | 5.11 | -4.42 | 0.00 |
| TRINITY_DN99686_c2_g1_i1 | | 2.87 | 0.25 | 3.46 | 0.00 |
| TRINITY_DN99688_c6_g1_i1 | TNR1A | 0.00 | 0.53 | -4.05 | 0.00 |
| TRINITY_DN99688_c7_g2_i2 | | 0.54 | 3.36 | -2.61 | 0.00 |
| TRINITY_DN99693_c1_g2_i4 | SAP | 2.76 | 12.11 | -2.13 | 0.00 |
| TRINITY_DN99693_c2_g2_i3 | MYO1F | 0.26 | 1.25 | -2.23 | 0.00 |

| | | | | | |
|---|---|---|---|---|---|
| TRINITY_DN99693_c2_g2_i4 | MYO1F | 3.67 | 8.76 | -1.26 | 0.00 |
| TRINITY_DN99714_c4_g1_i1 | LTC4S | 0.70 | 7.08 | -3.37 | 0.00 |
| TRINITY_DN99714_c4_g1_i5 | LTC4S | 0.23 | 1.89 | -3.02 | 0.00 |
| TRINITY_DN99715_c4_g1_i5 | DEFI6 | 1.97 | 6.48 | -1.74 | 0.00 |
| TRINITY_DN99715_c4_g1_i9 | DEFI6 | 0.15 | 1.94 | -3.57 | 0.00 |
| TRINITY_DN99715_c4_g2_i14 | | 0.09 | 2.05 | -3.39 | 0.00 |
| TRINITY_DN99724_c6_g1_i2 | | 0.30 | 2.95 | -3.19 | 0.00 |
| TRINITY_DN99733_c0_g1_i2 | K1C42 | 20.17 | 67.31 | -1.79 | 0.00 |
| TRINITY_DN99748_c2_g1_i9 | | 1.13 | 0.00 | 5.76 | 0.00 |
| TRINITY_DN99774_c9_g2_i3 | CX6B1 | 1.50 | 6.92 | -2.14 | 0.00 |
| TRINITY_DN99788_c1_g2_i1 | RHG15 | 0.33 | 2.55 | -2.95 | 0.00 |
| TRINITY_DN99793_c1_g1_i1 | | 1.47 | 18.41 | -3.41 | 0.00 |
| TRINITY_DN99793_c3_g3_i1 | | 0.37 | 2.15 | -2.30 | 0.00 |
| TRINITY_DN99813_c2_g1_i3 | B4GN3 | 0.00 | 1.03 | -6.40 | 0.00 |
| TRINITY_DN99819_c11_g2_i2 | | 0.10 | 2.14 | -4.19 | 0.00 |
| TRINITY_DN99823_c3_g1_i1 | | 0.27 | 6.89 | -4.30 | 0.00 |
| TRINITY_DN99830_c0_g1_i1 | MDM4 | 1.09 | 0.00 | 7.15 | 0.00 |
| TRINITY_DN99837_c2_g1_i4 | | 0.00 | 2.21 | -5.26 | 0.00 |
| TRINITY_DN99838_c12_g1_i5 | KLDC1 | 0.00 | 1.69 | -5.23 | 0.00 |
| TRINITY_DN99842_c1_g1_i8 | PLCB3 | 0.07 | 1.22 | -3.97 | 0.00 |
| TRINITY_DN99851_c8_g1_i3 | | 0.00 | 2.87 | -5.56 | 0.00 |
| TRINITY_DN99853_c5_g1_i4 | LOX5 | 0.12 | 4.35 | -5.20 | 0.00 |
| TRINITY_DN99853_c5_g1_i8 | | 0.23 | 2.74 | -3.17 | 0.00 |
| TRINITY_DN99895_c2_g4_i1 | TRFE | 29.78 | 3.71 | 3.06 | 0.00 |
| TRINITY_DN99898_c0_g3_i16 | TVA3 | 0.07 | 1.40 | -3.47 | 0.00 |
| TRINITY_DN99902_c4_g2_i3 | ZG16 | 1566.94 | 469.47 | 1.64 | 0.00 |
| TRINITY_DN99908_c0_g1_i1 | | 4.31 | 8.92 | -1.06 | 0.00 |
| TRINITY_DN99908_c0_g3_i1 | | 1.98 | 10.79 | -2.48 | 0.00 |
| TRINITY_DN99908_c0_g4_i2 | | 1.07 | 4.13 | -1.92 | 0.00 |
| TRINITY_DN99910_c8_g8_i1 | IL16 | 3.85 | 8.78 | -1.19 | 0.00 |
| TRINITY_DN99917_c0_g1_i9 | HMR1 | 0.89 | 4.37 | -2.27 | 0.00 |
| TRINITY_DN99921_c1_g1_i5 | SCAR3 | 0.85 | 0.02 | 5.28 | 0.00 |
| TRINITY_DN99933_c3_g4_i2 | | 0.36 | 4.60 | -3.46 | 0.00 |
| TRINITY_DN99948_c3_g1_i16 | TNR5 | 0.24 | 1.75 | -2.85 | 0.00 |
| TRINITY_DN99948_c3_g1_i8 | | 0.28 | 1.70 | -2.67 | 0.00 |
| TRINITY_DN99988_c2_g1_i4 | IP6K2 | 8.68 | 13.72 | -0.67 | 0.00 |
| TRINITY_DN99993_c0_g2_i1 | SOX9 | 16.17 | 26.12 | -0.70 | 0.00 |
| TRINITY_DN99993_c0_g3_i4 | SOX8 | 0.24 | 2.61 | -3.38 | 0.00 |
| TRINITY_DN99997_c1_g2_i2 | | 0.00 | 2.81 | -5.11 | 0.00 |
| TRINITY_DN99999_c0_g2_i2 | FLNC | 4.32 | 1.89 | 1.22 | 0.00 |

**Table S2.** List of the differentially expressed genes of the fin in yamame

| Gene_id | Annotation | HT_TPM | NT_TPM | log2(FoldChange) | *P* - value |
|---|---|---|---|---|---|
| TRINITY_DN100010_c5_g1_i4 | SAP | 0.06 | 3.27 | -6.51 | 0.00 |
| TRINITY_DN100020_c2_g1_i5 | CO3 | 4.83 | 0.04 | 3.11 | 0.00 |
| TRINITY_DN100136_c3_g1_i1 | | 0.49 | 7.06 | -3.90 | 0.00 |
| TRINITY_DN100181_c1_g1_i1 | URGCP | 0.00 | 1.42 | -6.21 | 0.00 |
| TRINITY_DN100207_c2_g4_i6 | FREM1 | 1.05 | 7.17 | -2.77 | 0.00 |
| TRINITY_DN100244_c5_g4_i2 | SEPT2 | 8.11 | 0.00 | 7.16 | 0.00 |
| TRINITY_DN100245_c1_g1_i5 | SF3B1 | 9.00 | 15.12 | -0.78 | 0.00 |
| TRINITY_DN100251_c1_g1_i14 | DDX17 | 1.29 | 3.76 | -1.57 | 0.00 |
| TRINITY_DN100257_c2_g1_i1 | VIME | 5.15 | 11.93 | -1.25 | 0.00 |
| TRINITY_DN100260_c3_g3_i1 | AGRG4 | 1.08 | 7.20 | -2.79 | 0.00 |
| TRINITY_DN100264_c1_g2_i4 | | 0.00 | 1.78 | -6.12 | 0.00 |
| TRINITY_DN100293_c2_g1_i4 | CNFNB | 61.37 | 26.65 | 1.17 | 0.00 |
| TRINITY_DN100304_c4_g2_i20 | GIMA4 | 2.41 | 9.66 | -2.01 | 0.00 |
| TRINITY_DN100329_c2_g1_i1 | | 0.00 | 5.07 | -22.65 | 0.00 |
| TRINITY_DN100358_c1_g2_i13 | MUC2L | 0.92 | 10.54 | -3.59 | 0.00 |
| TRINITY_DN100358_c1_g2_i16 | MUC5B | 0.83 | 0.00 | 5.14 | 0.00 |
| TRINITY_DN100358_c1_g2_i6 | MUC2L | 57.10 | 26.80 | 1.04 | 0.00 |
| TRINITY_DN100392_c2_g1_i5 | SYVC | 0.00 | 0.83 | -5.83 | 0.00 |
| TRINITY_DN100422_c0_g2_i3 | TBB | 10.05 | 18.22 | -0.91 | 0.00 |
| TRINITY_DN100423_c1_g1_i1 | KIF4 | 1.57 | 0.20 | 2.92 | 0.00 |
| TRINITY_DN100423_c1_g1_i5 | KIF4 | 1.39 | 0.32 | 2.05 | 0.00 |
| TRINITY_DN100481_c2_g1_i7 | M3K5 | 1.21 | 0.00 | 7.10 | 0.00 |
| TRINITY_DN100483_c2_g1_i12 | RGL2 | 2.67 | 0.05 | 5.51 | 0.00 |
| TRINITY_DN100513_c1_g4_i2 | | 0.22 | 1.54 | -2.85 | 0.00 |
| TRINITY_DN100533_c1_g1_i9 | COBA2 | 0.02 | 2.97 | -5.02 | 0.00 |
| TRINITY_DN100572_c1_g2_i3 | GVIN1 | 0.01 | 5.15 | -7.23 | 0.00 |
| TRINITY_DN100589_c6_g3_i1 | | 0.00 | 2.10 | -6.47 | 0.00 |
| TRINITY_DN100642_c5_g1_i14 | SESN1 | 14.32 | 32.05 | -1.18 | 0.00 |
| TRINITY_DN100645_c2_g2_i1 | GCN1 | 3.52 | 2.04 | 0.74 | 0.00 |
| TRINITY_DN100647_c0_g1_i1 | | 0.54 | 1.31 | -1.29 | 0.00 |
| TRINITY_DN100662_c1_g2_i4 | | 0.03 | 2.36 | -5.85 | 0.00 |
| TRINITY_DN100667_c3_g2_i1 | | 5.11 | 14.84 | -1.58 | 0.00 |
| TRINITY_DN100677_c8_g4_i4 | | 0.00 | 11.83 | -6.90 | 0.00 |
| TRINITY_DN100705_c5_g6_i1 | ETS2 | 35.42 | 57.06 | -0.71 | 0.00 |
| TRINITY_DN100727_c2_g1_i7 | RL7 | 23.12 | 77.70 | -1.80 | 0.00 |
| TRINITY_DN100748_c6_g2_i1 | FYCO1 | 2.73 | 0.09 | 4.94 | 0.00 |
| TRINITY_DN100748_c6_g2_i2 | FYCO1 | 0.00 | 1.03 | -5.13 | 0.00 |
| TRINITY_DN100789_c4_g1_i1 | LRBA | 4.63 | 3.35 | 0.44 | 0.00 |
| TRINITY_DN100824_c3_g2_i3 | CADH1 | 15.44 | 5.76 | 1.37 | 0.00 |

| | | | | | |
|---|---|---|---|---|---|
| TRINITY_DN100858_c5_g3_i3 | | 6.51 | 2.43 | 1.34 | 0.00 |
| TRINITY_DN100867_c1_g2_i5 | | 1.73 | 7.01 | -2.04 | 0.00 |
| TRINITY_DN100914_c0_g1_i3 | | 0.01 | 2.10 | -7.52 | 0.00 |
| TRINITY_DN100914_c0_g1_i6 | | 1.72 | 0.01 | 6.56 | 0.00 |
| TRINITY_DN100917_c9_g1_i1 | | 1.33 | 9.18 | -2.89 | 0.00 |
| TRINITY_DN100926_c2_g1_i1 | SPTB2 | 0.01 | 1.38 | -6.13 | 0.00 |
| TRINITY_DN100939_c26_g1_i1 | LMO7 | 0.00 | 1.78 | -7.10 | 0.00 |
| TRINITY_DN100968_c2_g1_i1 | TF29 | 5.04 | 9.08 | -0.90 | 0.00 |
| TRINITY_DN100975_c9_g1_i1 | | 1.04 | 6.33 | -2.68 | 0.00 |
| TRINITY_DN100982_c0_g1_i1 | | 5.18 | 16.24 | -1.68 | 0.00 |
| TRINITY_DN104860_c0_g1_i1 | RM02 | 9.93 | 5.53 | 0.81 | 0.00 |
| TRINITY_DN40439_c0_g1_i1 | HDAC6 | 9.42 | 1.63 | 2.47 | 0.00 |
| TRINITY_DN47095_c0_g1_i1 | UB4AA | 8.59 | 0.45 | 4.06 | 0.00 |
| TRINITY_DN61670_c0_g1_i1 | | 4.94 | 40.96 | -3.07 | 0.00 |
| TRINITY_DN69452_c0_g1_i1 | | 14.98 | 40.32 | -1.47 | 0.00 |
| TRINITY_DN69646_c0_g1_i1 | | 0.49 | 1.89 | -1.94 | 0.00 |
| TRINITY_DN71263_c0_g1_i1 | PSMD8 | 34.06 | 21.42 | 0.63 | 0.00 |
| TRINITY_DN71883_c0_g1_i1 | | 28.67 | 65.00 | -1.21 | 0.00 |
| TRINITY_DN74129_c0_g1_i3 | TRFE | 47.69 | 0.92 | 4.02 | 0.00 |
| TRINITY_DN75674_c0_g1_i2 | PFD6 | 25.97 | 14.53 | 0.80 | 0.00 |
| TRINITY_DN76007_c0_g1_i1 | PSB7 | 45.37 | 25.04 | 0.82 | 0.00 |
| TRINITY_DN76786_c0_g1_i1 | | 0.19 | 1.63 | -3.33 | 0.00 |
| TRINITY_DN78369_c0_g1_i1 | | 0.00 | 2.19 | -4.79 | 0.00 |
| TRINITY_DN78571_c0_g1_i1 | | 7.13 | 37.19 | -2.42 | 0.00 |
| TRINITY_DN79151_c0_g1_i1 | SELK | 11.70 | 1.05 | 3.47 | 0.00 |
| TRINITY_DN79306_c0_g1_i2 | | 31.92 | 86.52 | -1.48 | 0.00 |
| TRINITY_DN79748_c0_g1_i1 | CREM | 0.14 | 1.61 | -3.60 | 0.00 |
| TRINITY_DN80077_c0_g1_i2 | | 0.75 | 5.13 | -2.84 | 0.00 |
| TRINITY_DN80443_c0_g2_i2 | | 0.09 | 1.10 | -3.75 | 0.00 |
| TRINITY_DN80730_c0_g1_i1 | | 14.13 | 0.57 | 3.97 | 0.00 |
| TRINITY_DN80955_c0_g1_i1 | | 1.15 | 22.04 | -4.26 | 0.00 |
| TRINITY_DN81005_c0_g1_i1 | ANR22 | 5.92 | 10.02 | -0.81 | 0.00 |
| TRINITY_DN81011_c0_g1_i1 | SGO2 | 0.97 | 0.26 | 1.92 | 0.00 |
| TRINITY_DN81400_c0_g1_i4 | ECHM | 12.21 | 7.09 | 0.76 | 0.00 |
| TRINITY_DN81585_c0_g1_i1 | | 4.23 | 1.20 | 1.80 | 0.00 |
| TRINITY_DN81687_c0_g2_i2 | IF43B | 2.19 | 0.37 | 2.32 | 0.00 |
| TRINITY_DN81765_c0_g1_i3 | | 2.02 | 0.29 | 2.72 | 0.00 |
| TRINITY_DN81792_c0_g1_i1 | | 3.86 | 1.37 | 1.52 | 0.00 |
| TRINITY_DN81968_c0_g1_i1 | TOP2A | 7.20 | 2.03 | 1.80 | 0.00 |
| TRINITY_DN81970_c0_g1_i1 | | 3.91 | 0.99 | 1.91 | 0.00 |
| TRINITY_DN82062_c0_g1_i1 | SELT2 | 26.85 | 20.19 | 0.38 | 0.00 |
| TRINITY_DN82358_c5_g1_i1 | SOCS3 | 11.53 | 28.87 | -1.37 | 0.00 |

| | | | | | |
|---|---|---|---|---|---|
| TRINITY_DN82358_c5_g1_i2 | SOCS3 | 17.82 | 49.81 | -1.52 | 0.00 |
| TRINITY_DN82408_c0_g1_i3 | MUC5B | 4.06 | 0.35 | 3.42 | 0.00 |
| TRINITY_DN82426_c0_g1_i1 | | 0.55 | 15.86 | -4.39 | 0.00 |
| TRINITY_DN82433_c0_g1_i2 | SNX27 | 2.32 | 8.74 | -1.95 | 0.00 |
| TRINITY_DN82488_c0_g1_i1 | HG2A | 50.35 | 86.88 | -0.83 | 0.00 |
| TRINITY_DN82864_c0_g3_i1 | EVL | 0.34 | 1.49 | -2.08 | 0.00 |
| TRINITY_DN82946_c0_g1_i2 | | 0.37 | 3.50 | -3.35 | 0.00 |
| TRINITY_DN82967_c0_g1_i1 | SYAC | 8.95 | 4.22 | 1.03 | 0.00 |
| TRINITY_DN83096_c0_g1_i1 | | 3.50 | 19.03 | -2.49 | 0.00 |
| TRINITY_DN83194_c0_g1_i1 | TPSN | 6.22 | 11.72 | -0.94 | 0.00 |
| TRINITY_DN83200_c0_g1_i1 | IEX1 | 9.89 | 30.24 | -1.65 | 0.00 |
| TRINITY_DN83214_c0_g2_i1 | | 0.31 | 14.23 | -4.30 | 0.00 |
| TRINITY_DN83214_c0_g2_i2 | | 0.79 | 19.70 | -4.63 | 0.00 |
| TRINITY_DN83259_c8_g3_i2 | | 1.30 | 8.73 | -2.93 | 0.00 |
| TRINITY_DN83284_c0_g1_i3 | | 10.23 | 0.00 | 6.79 | 0.00 |
| TRINITY_DN83380_c2_g2_i1 | | 0.78 | 4.93 | -2.67 | 0.00 |
| TRINITY_DN83411_c0_g2_i1 | RLP24 | 113.95 | 179.06 | -0.69 | 0.00 |
| TRINITY_DN83666_c1_g1_i1 | ID1 | 46.70 | 67.96 | -0.58 | 0.00 |
| TRINITY_DN83738_c0_g1_i3 | | 3.91 | 23.41 | -2.75 | 0.00 |
| TRINITY_DN83824_c0_g1_i1 | HIG2A | 16.92 | 9.32 | 0.80 | 0.00 |
| TRINITY_DN83832_c0_g2_i1 | JUN | 10.02 | 18.19 | -0.89 | 0.00 |
| TRINITY_DN83840_c5_g2_i1 | | 0.67 | 7.69 | -3.42 | 0.00 |
| TRINITY_DN83910_c0_g1_i1 | MPP6 | 0.83 | 0.11 | 3.19 | 0.00 |
| TRINITY_DN83919_c0_g1_i2 | | 6.48 | 0.00 | 7.07 | 0.00 |
| TRINITY_DN83925_c1_g1_i1 | | 0.00 | 0.78 | -4.71 | 0.00 |
| TRINITY_DN84026_c8_g1_i1 | | 0.39 | 3.04 | -2.98 | 0.00 |
| TRINITY_DN84054_c1_g3_i9 | KI67 | 3.22 | 0.00 | 7.73 | 0.00 |
| TRINITY_DN84061_c4_g9_i1 | CALM | 70.40 | 40.88 | 0.74 | 0.00 |
| TRINITY_DN84101_c1_g1_i10 | TBB | 1.06 | 4.71 | -2.17 | 0.00 |
| TRINITY_DN84180_c4_g3_i1 | | 9.40 | 0.20 | 4.31 | 0.00 |
| TRINITY_DN84180_c5_g2_i1 | EGR1 | 17.58 | 65.81 | -1.97 | 0.00 |
| TRINITY_DN84180_c5_g3_i1 | EGR1 | 3.40 | 17.11 | -2.36 | 0.00 |
| TRINITY_DN84265_c9_g2_i9 | K1C42 | 21.79 | 58.88 | -1.51 | 0.00 |
| TRINITY_DN84393_c6_g1_i11 | | 0.37 | 6.61 | -4.08 | 0.00 |
| TRINITY_DN84397_c0_g1_i3 | | 52.56 | 18.39 | 1.45 | 0.00 |
| TRINITY_DN84400_c8_g1_i3 | | 22.81 | 2.85 | 2.84 | 0.00 |
| TRINITY_DN84404_c4_g1_i1 | SIA4B | 2.28 | 0.36 | 2.64 | 0.00 |
| TRINITY_DN84482_c0_g1_i1 | ANO8 | 0.41 | 2.18 | -2.43 | 0.00 |
| TRINITY_DN84498_c0_g1_i3 | IGSF6 | 1.81 | 0.14 | 3.45 | 0.00 |
| TRINITY_DN84521_c7_g2_i2 | | 0.00 | 2.22 | -5.84 | 0.00 |
| TRINITY_DN84538_c3_g2_i2 | | 3.51 | 7.40 | -1.10 | 0.00 |
| TRINITY_DN84538_c3_g8_i1 | | 2.01 | 13.32 | -2.54 | 0.00 |

| | | | | | |
|---|---|---|---|---|---|
| TRINITY_DN84621_c4_g2_i3 | CCL28 | 236.06 | 372.04 | -0.70 | 0.00 |
| TRINITY_DN84655_c3_g8_i3 | | 0.49 | 3.25 | -2.80 | 0.00 |
| TRINITY_DN84673_c4_g1_i7 | RAB25 | 0.00 | 9.73 | -8.82 | 0.00 |
| TRINITY_DN84694_c1_g1_i1 | PRS6B | 11.81 | 7.03 | 0.70 | 0.00 |
| TRINITY_DN84716_c5_g1_i1 | BSPRY | 0.01 | 0.48 | -4.51 | 0.00 |
| TRINITY_DN84751_c3_g1_i5 | | 0.00 | 1.23 | -5.14 | 0.00 |
| TRINITY_DN84758_c0_g1_i2 | CY1 | 0.00 | 10.06 | -8.36 | 0.00 |
| TRINITY_DN84823_c3_g1_i2 | | 2.23 | 9.91 | -2.24 | 0.00 |
| TRINITY_DN84833_c0_g1_i1 | | 0.00 | 1.90 | -6.05 | 0.00 |
| TRINITY_DN84842_c0_g1_i3 | ACBD6 | 3.99 | 1.75 | 1.16 | 0.00 |
| TRINITY_DN84871_c1_g1_i2 | SOX3 | 7.98 | 14.40 | -0.90 | 0.00 |
| TRINITY_DN84877_c4_g1_i2 | | 0.03 | 2.17 | -4.97 | 0.00 |
| TRINITY_DN84879_c1_g2_i1 | | 20.58 | 1.12 | 4.45 | 0.00 |
| TRINITY_DN84879_c1_g3_i1 | | 202.52 | 16.55 | 3.70 | 0.00 |
| TRINITY_DN84937_c0_g1_i5 | HMR1 | 0.05 | 41.75 | -9.97 | 0.00 |
| TRINITY_DN84940_c1_g1_i1 | | 19.53 | 33.06 | -0.79 | 0.00 |
| TRINITY_DN84947_c4_g2_i1 | | 1.26 | 3.61 | -1.55 | 0.00 |
| TRINITY_DN84956_c4_g1_i1 | | 0.35 | 1.99 | -2.80 | 0.00 |
| TRINITY_DN84957_c0_g3_i3 | RECQ4 | 1.24 | 0.23 | 2.32 | 0.00 |
| TRINITY_DN84977_c0_g1_i1 | JUN | 5.87 | 13.14 | -1.20 | 0.00 |
| TRINITY_DN85027_c3_g1_i3 | PHLA2 | 10.35 | 22.93 | -1.20 | 0.00 |
| TRINITY_DN85073_c1_g1_i1 | | 491.22 | 191.53 | 1.30 | 0.00 |
| TRINITY_DN85095_c1_g2_i1 | | 1.05 | 5.37 | -2.51 | 0.00 |
| TRINITY_DN85112_c3_g4_i1 | | 0.31 | 2.14 | -2.81 | 0.00 |
| TRINITY_DN85115_c6_g2_i2 | RS12 | 516.07 | 245.14 | 1.05 | 0.00 |
| TRINITY_DN85122_c4_g7_i1 | | 112.12 | 567.44 | -2.38 | 0.00 |
| TRINITY_DN85123_c6_g1_i3 | SELH | 17.16 | 7.75 | 1.09 | 0.00 |
| TRINITY_DN85131_c0_g1_i3 | HB2D | 18.98 | 43.44 | -1.21 | 0.00 |
| TRINITY_DN85138_c5_g4_i2 | | 0.09 | 6.77 | -5.91 | 0.00 |
| TRINITY_DN85168_c0_g1_i1 | | 0.49 | 7.65 | -4.00 | 0.00 |
| TRINITY_DN85178_c3_g1_i13 | | 9.17 | 1.00 | 4.47 | 0.00 |
| TRINITY_DN85208_c0_g1_i2 | CCL20 | 0.34 | 3.31 | -3.39 | 0.00 |
| TRINITY_DN85239_c7_g1_i1 | HIG1A | 14.62 | 5.71 | 1.31 | 0.00 |
| TRINITY_DN85279_c0_g1_i2 | | 14.44 | 64.86 | -2.16 | 0.00 |
| TRINITY_DN85303_c0_g1_i1 | ODO1 | 9.91 | 6.25 | 0.64 | 0.00 |
| TRINITY_DN85377_c7_g1_i6 | SYFB | 13.50 | 7.60 | 0.79 | 0.00 |
| TRINITY_DN85418_c4_g2_i6 | ZBT26 | 0.01 | 0.71 | -6.25 | 0.00 |
| TRINITY_DN85460_c0_g1_i2 | SAA5 | 82.56 | 7.07 | 3.63 | 0.00 |
| TRINITY_DN85461_c3_g1_i1 | | 2.62 | 16.10 | -2.67 | 0.00 |
| TRINITY_DN85519_c5_g2_i4 | PROF2 | 0.55 | 5.74 | -3.36 | 0.00 |
| TRINITY_DN85557_c6_g1_i4 | P73 | 1.34 | 0.00 | 22.72 | 0.00 |
| TRINITY_DN85579_c0_g2_i2 | IF44L | 0.00 | 1.00 | -5.46 | 0.00 |

| | | | | | |
|---|---|---|---|---|---|
| TRINITY_DN85579_c0_g2_i5 | IF44L | 0.00 | 1.54 | -6.06 | 0.00 |
| TRINITY_DN85579_c0_g2_i6 | IF44L | 0.07 | 2.86 | -5.25 | 0.00 |
| TRINITY_DN85579_c0_g2_i8 | IF44L | 0.04 | 5.55 | -6.79 | 0.00 |
| TRINITY_DN85591_c4_g1_i2 | CND2 | 5.33 | 1.66 | 1.63 | 0.00 |
| TRINITY_DN85606_c1_g1_i4 | | 1.26 | 2.40 | -0.96 | 0.00 |
| TRINITY_DN85619_c1_g2_i1 | GNA14 | 1.15 | 4.03 | -1.83 | 0.00 |
| TRINITY_DN85652_c6_g1_i2 | PPIC | 1.27 | 2.90 | -1.24 | 0.00 |
| TRINITY_DN85696_c0_g1_i2 | TAGAP | 0.95 | 2.01 | -1.11 | 0.00 |
| TRINITY_DN85741_c4_g1_i1 | | 3.43 | 1.44 | 1.22 | 0.00 |
| TRINITY_DN85741_c5_g3_i1 | | 48.28 | 9.69 | 2.30 | 0.00 |
| TRINITY_DN85749_c0_g1_i9 | REG1B | 0.06 | 2.04 | -4.66 | 0.00 |
| TRINITY_DN85775_c1_g1_i2 | | 2.06 | 14.44 | -2.78 | 0.00 |
| TRINITY_DN85807_c4_g1_i2 | GATA2 | 1.21 | 0.00 | 4.28 | 0.00 |
| TRINITY_DN85815_c3_g4_i3 | | 0.20 | 2.03 | -3.34 | 0.00 |
| TRINITY_DN85837_c2_g1_i2 | MD2L1 | 5.67 | 2.07 | 1.39 | 0.00 |
| TRINITY_DN85882_c0_g2_i2 | UROK | 22.00 | 37.29 | -0.79 | 0.00 |
| TRINITY_DN85929_c2_g1_i1 | | 0.00 | 3.13 | -5.03 | 0.00 |
| TRINITY_DN85929_c2_g4_i2 | HG2A | 3.98 | 85.46 | -4.46 | 0.00 |
| TRINITY_DN85965_c8_g1_i1 | | 6.41 | 1.25 | 2.33 | 0.00 |
| TRINITY_DN85991_c1_g1_i1 | | 26.22 | 86.84 | -1.79 | 0.00 |
| TRINITY_DN85999_c4_g2_i7 | SRS11 | 12.12 | 5.75 | 1.06 | 0.00 |
| TRINITY_DN86069_c6_g1_i4 | UBL5 | 3.83 | 0.43 | 3.36 | 0.00 |
| TRINITY_DN86079_c2_g1_i7 | PLPP3 | 5.81 | 0.28 | 4.51 | 0.00 |
| TRINITY_DN86088_c1_g1_i2 | EMIL2 | 0.00 | 5.93 | -8.86 | 0.00 |
| TRINITY_DN86088_c1_g1_i5 | EMIL2 | 0.00 | 1.01 | -6.28 | 0.00 |
| TRINITY_DN86135_c4_g4_i2 | | 0.00 | 9.91 | -5.76 | 0.00 |
| TRINITY_DN86162_c5_g1_i2 | | 1.24 | 12.54 | -3.54 | 0.00 |
| TRINITY_DN86176_c10_g1_i2 | | 1.05 | 6.26 | -2.65 | 0.00 |
| TRINITY_DN86229_c5_g1_i1 | TM14C | 21.14 | 8.98 | 1.25 | 0.00 |
| TRINITY_DN86283_c1_g1_i3 | | 0.00 | 2.68 | -5.18 | 0.00 |
| TRINITY_DN86299_c14_g1_i1 | | 8.30 | 1.06 | 2.86 | 0.00 |
| TRINITY_DN86325_c7_g1_i1 | ALKMO | 1.41 | 4.62 | -1.73 | 0.00 |
| TRINITY_DN86332_c2_g1_i2 | | 0.68 | 6.53 | -3.37 | 0.00 |
| TRINITY_DN86332_c2_g7_i1 | | 1.52 | 7.40 | -2.31 | 0.00 |
| TRINITY_DN86357_c2_g1_i2 | CLD3 | 5.37 | 15.48 | -1.56 | 0.00 |
| TRINITY_DN86357_c2_g2_i4 | CLD4 | 13.60 | 26.00 | -0.97 | 0.00 |
| TRINITY_DN86370_c0_g1_i4 | TRAK2 | 2.67 | 0.25 | 3.28 | 0.00 |
| TRINITY_DN86405_c0_g1_i8 | IRF1 | 0.81 | 6.03 | -2.95 | 0.00 |
| TRINITY_DN86447_c8_g2_i1 | | 0.13 | 2.36 | -4.80 | 0.00 |
| TRINITY_DN86451_c0_g1_i8 | YBX2B | 0.00 | 0.72 | -4.86 | 0.00 |
| TRINITY_DN86458_c2_g1_i5 | BIN3 | 0.00 | 2.32 | -7.13 | 0.00 |
| TRINITY_DN86486_c0_g1_i7 | | 0.33 | 3.66 | -3.43 | 0.00 |

| | | | | | |
|---|---|---|---|---|---|
| TRINITY_DN86491_c3_g3_i4 | | 0.03 | 1.45 | -4.44 | 0.00 |
| TRINITY_DN86499_c4_g3_i1 | PLD3B | 10.47 | 6.30 | 0.71 | 0.00 |
| TRINITY_DN86520_c2_g2_i1 | | 0.30 | 2.08 | -2.83 | 0.00 |
| TRINITY_DN86530_c1_g2_i17 | | 2.58 | 0.07 | 4.40 | 0.00 |
| TRINITY_DN86541_c2_g2_i2 | | 3.66 | 8.57 | -1.29 | 0.00 |
| TRINITY_DN86545_c2_g2_i4 | | 17.82 | 8.12 | 1.08 | 0.00 |
| TRINITY_DN86554_c7_g1_i7 | CDO1 | 0.00 | 0.64 | -4.61 | 0.00 |
| TRINITY_DN86570_c9_g4_i1 | APOC1 | 302.55 | 28.52 | 1.50 | 0.00 |
| TRINITY_DN86608_c0_g1_i2 | TRAP1 | 2.14 | 0.10 | 4.24 | 0.00 |
| TRINITY_DN86688_c6_g1_i7 | CD82 | 0.00 | 11.12 | -8.18 | 0.00 |
| TRINITY_DN86724_c8_g1_i1 | | 0.94 | 3.12 | -1.80 | 0.00 |
| TRINITY_DN86741_c2_g1_i4 | GFPT1 | 6.11 | 0.00 | 6.24 | 0.00 |
| TRINITY_DN86743_c6_g3_i1 | ARCH | 11.04 | 5.80 | 0.90 | 0.00 |
| TRINITY_DN86745_c2_g2_i1 | S27A4 | 0.96 | 0.10 | 3.12 | 0.00 |
| TRINITY_DN86763_c1_g3_i1 | H2AZ | 30.56 | 11.07 | 1.41 | 0.00 |
| TRINITY_DN86795_c0_g1_i1 | | 0.09 | 7.83 | -5.86 | 0.00 |
| TRINITY_DN86798_c0_g2_i3 | | 0.47 | 3.42 | -3.18 | 0.00 |
| TRINITY_DN86880_c4_g1_i2 | | 0.44 | 4.38 | -3.41 | 0.00 |
| TRINITY_DN86905_c6_g2_i1 | I20RB | 1.36 | 0.07 | 3.97 | 0.00 |
| TRINITY_DN86909_c11_g1_i2 | SNX3 | 21.76 | 14.49 | 0.54 | 0.00 |
| TRINITY_DN86920_c9_g1_i1 | GSTA | 45.89 | 28.72 | 0.65 | 0.00 |
| TRINITY_DN86957_c1_g2_i3 | | 1.55 | 0.00 | 4.38 | 0.00 |
| TRINITY_DN86967_c0_g3_i1 | DMXL2 | 0.74 | 0.00 | 5.66 | 0.00 |
| TRINITY_DN87061_c0_g1_i1 | CEBPD | 47.74 | 84.38 | -0.85 | 0.00 |
| TRINITY_DN87061_c0_g1_i2 | CEBPD | 62.90 | 113.90 | -0.88 | 0.00 |
| TRINITY_DN87071_c4_g1_i3 | | 1.80 | 7.26 | -2.03 | 0.00 |
| TRINITY_DN87135_c1_g1_i2 | | 0.00 | 26.26 | -10.06 | 0.00 |
| TRINITY_DN87135_c1_g1_i3 | | 0.00 | 26.80 | -10.42 | 0.00 |
| TRINITY_DN87143_c8_g2_i4 | RAB10 | 0.00 | 4.34 | -7.71 | 0.00 |
| TRINITY_DN87259_c3_g2_i2 | MYOC | 0.01 | 11.37 | -8.77 | 0.00 |
| TRINITY_DN87267_c0_g1_i1 | PGRP2 | 4.80 | 8.62 | -0.88 | 0.00 |
| TRINITY_DN87275_c0_g1_i1 | | 0.25 | 1.52 | -2.63 | 0.00 |
| TRINITY_DN87278_c10_g2_i2 | | 3.35 | 13.69 | -2.09 | 0.00 |
| TRINITY_DN87380_c0_g1_i5 | | 2.08 | 0.00 | 4.73 | 0.00 |
| TRINITY_DN87457_c3_g3_i3 | | 14.90 | 31.17 | -1.09 | 0.00 |
| TRINITY_DN87460_c2_g3_i1 | | 4.38 | 0.21 | 4.18 | 0.00 |
| TRINITY_DN87464_c10_g2_i1 | | 6.37 | 2.33 | 1.40 | 0.00 |
| TRINITY_DN87514_c4_g2_i2 | FAP24 | 4.71 | 2.64 | 0.79 | 0.00 |
| TRINITY_DN87527_c4_g1_i8 | | 0.14 | 2.34 | -4.01 | 0.00 |
| TRINITY_DN87531_c3_g1_i3 | TNNI1 | 4.31 | 15.66 | -1.93 | 0.00 |
| TRINITY_DN87581_c3_g1_i2 | | 2.36 | 0.16 | 3.30 | 0.00 |
| TRINITY_DN87614_c5_g2_i1 | | 3.76 | 10.80 | -1.56 | 0.00 |

| | | | | | |
|---|---|---|---|---|---|
| TRINITY_DN87617_c6_g1_i2 | RAB32 | 1.02 | 0.04 | 4.09 | 0.00 |
| TRINITY_DN87640_c1_g1_i9 | RAB9A | 1.31 | 0.17 | 3.13 | 0.00 |
| TRINITY_DN87687_c5_g1_i12 | IKBA | 12.98 | 26.31 | -1.05 | 0.00 |
| TRINITY_DN87687_c5_g1_i13 | IKBA | 2.97 | 7.20 | -1.30 | 0.00 |
| TRINITY_DN87687_c5_g1_i18 | IKBA | 10.34 | 23.16 | -1.18 | 0.00 |
| TRINITY_DN87687_c5_g1_i2 | IKBA | 24.34 | 56.84 | -1.27 | 0.00 |
| TRINITY_DN87697_c3_g2_i3 | A9A1A | 2.84 | 0.00 | 7.08 | 0.00 |
| TRINITY_DN87697_c3_g2_i4 | A9A1A | 2.73 | 0.00 | 6.77 | 0.00 |
| TRINITY_DN87700_c3_g1_i5 | | 0.00 | 3.44 | -5.49 | 0.00 |
| TRINITY_DN87720_c0_g1_i6 | COPT1 | 0.77 | 0.02 | 4.62 | 0.00 |
| TRINITY_DN87762_c0_g1_i6 | BRPF3 | 0.94 | 0.13 | 2.91 | 0.00 |
| TRINITY_DN87764_c2_g1_i1 | CCL25 | 120.14 | 199.44 | -0.75 | 0.00 |
| TRINITY_DN87830_c1_g1_i8 | CD9 | 0.04 | 2.54 | -5.58 | 0.00 |
| TRINITY_DN87883_c3_g3_i1 | | 64.78 | 40.69 | 0.63 | 0.00 |
| TRINITY_DN87934_c5_g2_i4 | | 764.74 | 1506.09 | -1.03 | 0.00 |
| TRINITY_DN87934_c5_g2_i5 | | 406.38 | 1142.80 | -1.55 | 0.00 |
| TRINITY_DN87951_c7_g5_i2 | | 0.11 | 4.39 | -4.31 | 0.00 |
| TRINITY_DN87952_c0_g1_i1 | KV315 | 0.54 | 3.16 | -2.65 | 0.00 |
| TRINITY_DN87952_c0_g1_i2 | | 0.45 | 2.03 | -2.26 | 0.00 |
| TRINITY_DN87956_c4_g1_i2 | | 1.50 | 3.78 | -1.34 | 0.00 |
| TRINITY_DN88074_c3_g1_i1 | HG2A | 24.64 | 66.67 | -1.49 | 0.00 |
| TRINITY_DN88074_c3_g2_i1 | HG2A | 168.88 | 476.71 | -1.55 | 0.00 |
| TRINITY_DN88076_c0_g1_i15 | | 14.61 | 0.00 | 5.60 | 0.00 |
| TRINITY_DN88076_c0_g2_i2 | | 13.16 | 0.76 | 4.06 | 0.00 |
| TRINITY_DN88115_c4_g2_i1 | | 6.73 | 11.11 | -0.75 | 0.00 |
| TRINITY_DN88140_c5_g1_i1 | | 73.30 | 5.01 | 2.61 | 0.00 |
| TRINITY_DN88160_c4_g1_i1 | | 5.48 | 1.10 | 2.22 | 0.00 |
| TRINITY_DN88167_c1_g1_i1 | CD3E | 2.43 | 5.06 | -1.11 | 0.00 |
| TRINITY_DN88170_c5_g3_i1 | CX023 | 0.10 | 3.39 | -4.64 | 0.00 |
| TRINITY_DN88183_c5_g4_i1 | CH25H | 6.34 | 14.57 | -1.24 | 0.00 |
| TRINITY_DN88246_c3_g2_i1 | | 2.10 | 18.93 | -3.21 | 0.00 |
| TRINITY_DN88257_c1_g4_i1 | AMOT | 0.00 | 0.54 | -5.91 | 0.00 |
| TRINITY_DN88261_c1_g1_i7 | | 0.03 | 0.81 | -4.09 | 0.00 |
| TRINITY_DN88269_c1_g1_i1 | PGRP2 | 3.00 | 0.63 | 2.22 | 0.00 |
| TRINITY_DN88270_c1_g1_i1 | | 3.77 | 0.53 | 2.69 | 0.00 |
| TRINITY_DN88335_c0_g1_i1 | TNF10 | 20.05 | 28.94 | -0.56 | 0.00 |
| TRINITY_DN88337_c1_g1_i1 | RAP1B | 0.39 | 1.25 | -5.53 | 0.00 |
| TRINITY_DN88337_c2_g1_i2 | PGAM1 | 12.11 | 5.53 | 1.11 | 0.00 |
| TRINITY_DN88337_c2_g1_i4 | PGAM1 | 0.00 | 2.68 | -6.49 | 0.00 |
| TRINITY_DN88431_c11_g2_i2 | | 0.35 | 4.01 | -3.33 | 0.00 |
| TRINITY_DN88466_c7_g1_i2 | | 7.29 | 3.87 | 0.87 | 0.00 |
| TRINITY_DN88482_c0_g1_i1 | CCD80 | 7.93 | 17.15 | -1.17 | 0.00 |

| | | | | | |
|---|---|---|---|---|---|
| TRINITY_DN88554_c3_g1_i9 | PR15B | 1.56 | 5.89 | -1.94 | 0.00 |
| TRINITY_DN88602_c4_g4_i3 | BORG1 | 5.67 | 1.01 | 2.46 | 0.00 |
| TRINITY_DN88602_c4_g4_i6 | BORG1 | 0.13 | 2.79 | -4.71 | 0.00 |
| TRINITY_DN88675_c2_g1_i7 | AKIR2 | 3.27 | 0.02 | 6.45 | 0.00 |
| TRINITY_DN88684_c1_g1_i1 | | 4.81 | 11.13 | -1.24 | 0.00 |
| TRINITY_DN88706_c0_g2_i4 | ODPX | 2.32 | 0.00 | 6.74 | 0.00 |
| TRINITY_DN88712_c0_g1_i7 | TM127 | 4.14 | 7.65 | -0.91 | 0.00 |
| TRINITY_DN88764_c1_g2_i3 | | 1.14 | 0.12 | 2.96 | 0.00 |
| TRINITY_DN88769_c4_g4_i2 | | 0.63 | 13.73 | -4.27 | 0.00 |
| TRINITY_DN88816_c3_g3_i1 | | 4.75 | 0.00 | 7.59 | 0.00 |
| TRINITY_DN88858_c3_g1_i2 | FLOT2 | 0.00 | 2.80 | -5.34 | 0.00 |
| TRINITY_DN88858_c3_g1_i4 | FLOT2 | 0.07 | 2.65 | -5.13 | 0.00 |
| TRINITY_DN88875_c4_g1_i4 | CCDB1 | 2.75 | 0.13 | 4.42 | 0.00 |
| TRINITY_DN88927_c2_g1_i3 | NDUAA | 18.77 | 9.57 | 0.95 | 0.00 |
| TRINITY_DN88952_c11_g2_i1 | | 0.16 | 4.46 | -4.23 | 0.00 |
| TRINITY_DN89014_c1_g1_i2 | SEPT7 | 8.84 | 4.00 | 1.11 | 0.00 |
| TRINITY_DN89024_c0_g1_i4 | XBP1 | 7.32 | 11.15 | -0.65 | 0.00 |
| TRINITY_DN89066_c3_g1_i2 | | 5.14 | 12.37 | -1.30 | 0.00 |
| TRINITY_DN89080_c6_g1_i7 | EXO1 | 0.76 | 0.02 | 4.36 | 0.00 |
| TRINITY_DN89143_c2_g1_i9 | CDC20 | 4.27 | 0.92 | 2.17 | 0.00 |
| TRINITY_DN89159_c7_g2_i1 | | 0.37 | 4.10 | -3.78 | 0.00 |
| TRINITY_DN89175_c4_g1_i1 | FRRS1 | 3.82 | 9.98 | -1.42 | 0.00 |
| TRINITY_DN89233_c3_g1_i2 | | 21.45 | 96.37 | -2.19 | 0.00 |
| TRINITY_DN89266_c1_g1_i4 | EMAL3 | 0.00 | 3.84 | -7.48 | 0.00 |
| TRINITY_DN89268_c1_g4_i1 | IER2 | 3.50 | 9.57 | -1.47 | 0.00 |
| TRINITY_DN89272_c4_g1_i8 | MIC19 | 1.59 | 0.02 | 5.15 | 0.00 |
| TRINITY_DN89276_c7_g1_i1 | GVIN1 | 0.00 | 6.86 | -7.79 | 0.00 |
| TRINITY_DN89395_c3_g1_i7 | IQGA1 | 6.01 | 0.81 | 2.86 | 0.00 |
| TRINITY_DN89430_c2_g1_i3 | DEPD7 | 1.26 | 0.11 | 3.51 | 0.00 |
| TRINITY_DN89435_c7_g1_i11 | | 4.68 | 0.12 | 4.62 | 0.00 |
| TRINITY_DN89435_c7_g1_i2 | | 0.00 | 3.96 | -5.37 | 0.00 |
| TRINITY_DN89449_c3_g2_i1 | INK1B | 4.33 | 7.69 | -0.85 | 0.00 |
| TRINITY_DN89464_c0_g1_i6 | CAN9 | 22.13 | 7.78 | 1.45 | 0.00 |
| TRINITY_DN89469_c2_g1_i1 | TNMD | 14.58 | 58.34 | -2.03 | 0.00 |
| TRINITY_DN89506_c5_g2_i4 | | 0.04 | 0.54 | -3.68 | 0.00 |
| TRINITY_DN89647_c7_g1_i2 | | 0.00 | 9.33 | -7.68 | 0.00 |
| TRINITY_DN89665_c0_g2_i1 | SPDEF | 0.47 | 1.59 | -1.76 | 0.00 |
| TRINITY_DN89668_c0_g2_i1 | | 0.54 | 2.51 | -2.23 | 0.00 |
| TRINITY_DN89712_c0_g2_i6 | DUS1 | 3.16 | 12.19 | -1.98 | 0.00 |
| TRINITY_DN89712_c0_g2_i8 | | 3.52 | 12.85 | -1.87 | 0.00 |
| TRINITY_DN89720_c4_g1_i2 | | 0.46 | 10.52 | -4.49 | 0.00 |
| TRINITY_DN89723_c4_g3_i1 | | 5.50 | 11.60 | -1.11 | 0.00 |

| | | | | | |
|---|---|---|---|---|---|
| TRINITY_DN89726_c6_g1_i4 | FCGBP | 8.24 | 2.20 | 1.91 | 0.00 |
| TRINITY_DN89733_c3_g1_i2 | | 0.12 | 1.65 | -4.06 | 0.00 |
| TRINITY_DN89840_c3_g1_i6 | | 0.00 | 0.50 | -5.10 | 0.00 |
| TRINITY_DN89883_c0_g1_i5 | K1C13 | 17.12 | 6.48 | 1.35 | 0.00 |
| TRINITY_DN89891_c1_g1_i3 | SAMD9 | 0.12 | 1.65 | -3.92 | 0.00 |
| TRINITY_DN89891_c1_g2_i1 | SAM9L | 0.15 | 1.51 | -3.33 | 0.00 |
| TRINITY_DN89896_c9_g1_i7 | EDF1 | 23.59 | 11.01 | 1.06 | 0.00 |
| TRINITY_DN89912_c9_g3_i6 | FER | 0.00 | 0.95 | -6.01 | 0.00 |
| TRINITY_DN90007_c3_g2_i3 | K1C18 | 51.47 | 115.01 | -1.22 | 0.00 |
| TRINITY_DN90014_c2_g1_i1 | AVIL | 3.75 | 8.30 | -1.18 | 0.00 |
| TRINITY_DN90014_c2_g1_i2 | AVIL | 0.47 | 1.34 | -1.60 | 0.00 |
| TRINITY_DN90029_c0_g1_i1 | CNST | 1.39 | 0.00 | 5.41 | 0.00 |
| TRINITY_DN90067_c2_g1_i7 | TSN11 | 0.10 | 1.48 | -3.98 | 0.00 |
| TRINITY_DN90094_c0_g1_i1 | | 8.50 | 81.93 | -3.32 | 0.00 |
| TRINITY_DN90094_c0_g1_i2 | | 1.90 | 24.07 | -3.74 | 0.00 |
| TRINITY_DN90094_c0_g1_i6 | | 0.40 | 10.46 | -4.55 | 0.00 |
| TRINITY_DN90094_c0_g1_i8 | | 9.27 | 59.52 | -2.71 | 0.00 |
| TRINITY_DN90094_c0_g1_i9 | | 2.34 | 19.73 | -3.12 | 0.00 |
| TRINITY_DN90110_c1_g2_i2 | GILT | 16.66 | 30.41 | -0.90 | 0.00 |
| TRINITY_DN9017_c0_g1_i1 | HGD | 1.49 | 20.98 | -3.89 | 0.00 |
| TRINITY_DN90191_c5_g2_i1 | LOX5 | 0.31 | 2.02 | -2.80 | 0.00 |
| TRINITY_DN90251_c14_g1_i1 | | 0.20 | 5.39 | -4.83 | 0.00 |
| TRINITY_DN90265_c2_g1_i1 | PLA2R | 0.20 | 2.34 | -3.39 | 0.00 |
| TRINITY_DN90265_c2_g1_i9 | PLA2R | 0.63 | 5.79 | -3.21 | 0.00 |
| TRINITY_DN90302_c2_g1_i3 | POF1B | 0.90 | 0.02 | 4.80 | 0.00 |
| TRINITY_DN90316_c2_g1_i3 | | 5.67 | 14.20 | -1.35 | 0.00 |
| TRINITY_DN90321_c1_g2_i10 | RGS1 | 7.47 | 23.55 | -1.68 | 0.00 |
| TRINITY_DN90425_c6_g2_i1 | | 0.51 | 2.25 | -2.16 | 0.00 |
| TRINITY_DN90436_c5_g1_i1 | | 90.68 | 132.60 | -0.59 | 0.00 |
| TRINITY_DN90544_c6_g1_i1 | | 2.15 | 15.84 | -2.94 | 0.00 |
| TRINITY_DN90544_c6_g2_i2 | | 0.05 | 4.90 | -5.55 | 0.00 |
| TRINITY_DN90544_c6_g2_i3 | | 2.24 | 19.33 | -3.14 | 0.00 |
| TRINITY_DN90635_c1_g3_i2 | | 0.00 | 0.78 | -5.33 | 0.00 |
| TRINITY_DN90654_c0_g1_i1 | GFPT1 | 5.32 | 0.34 | 4.10 | 0.00 |
| TRINITY_DN90702_c5_g2_i1 | IL12B | 0.18 | 4.22 | -4.53 | 0.00 |
| TRINITY_DN90719_c0_g1_i1 | M1I1B | 65.32 | 177.03 | -1.48 | 0.00 |
| TRINITY_DN90746_c4_g1_i1 | RHBL4 | 0.04 | 1.12 | -4.47 | 0.00 |
| TRINITY_DN90770_c1_g1_i1 | RN146 | 0.00 | 1.15 | -6.00 | 0.00 |
| TRINITY_DN90779_c3_g2_i2 | | 10.79 | 4.86 | 1.09 | 0.00 |
| TRINITY_DN90840_c0_g2_i1 | | 0.02 | 0.62 | -4.22 | 0.00 |
| TRINITY_DN90915_c0_g5_i1 | NDC80 | 2.74 | 0.83 | 1.67 | 0.00 |
| TRINITY_DN90937_c2_g2_i2 | ANGL4 | 21.79 | 38.27 | -0.84 | 0.00 |

| | | | | | |
|---|---|---|---|---|---|
| TRINITY_DN90955_c3_g6_i1 | | 5.26 | 9.93 | -0.95 | 0.00 |
| TRINITY_DN90975_c6_g1_i5 | IFI44 | 0.18 | 3.21 | -4.66 | 0.00 |
| TRINITY_DN90991_c3_g1_i13 | MYO1C | 0.35 | 1.64 | -2.22 | 0.00 |
| TRINITY_DN90992_c0_g1_i9 | RIPK3 | 0.51 | 3.50 | -2.73 | 0.00 |
| TRINITY_DN90994_c0_g1_i5 | MDHC | 9.33 | 4.96 | 0.88 | 0.00 |
| TRINITY_DN91000_c0_g1_i6 | LYAM1 | 0.69 | 10.88 | -3.88 | 0.00 |
| TRINITY_DN91003_c7_g1_i1 | | 0.00 | 1.58 | -5.50 | 0.00 |
| TRINITY_DN91017_c9_g2_i1 | QCR2 | 5.98 | 3.31 | 0.81 | 0.00 |
| TRINITY_DN91036_c1_g1_i6 | TENS | 0.93 | 3.77 | -2.01 | 0.00 |
| TRINITY_DN91132_c9_g1_i1 | MIC60 | 8.05 | 4.70 | 0.75 | 0.00 |
| TRINITY_DN91137_c2_g1_i1 | K1C18 | 0.00 | 10.50 | -23.47 | 0.00 |
| TRINITY_DN91140_c0_g1_i4 | NECP2 | 3.45 | 0.00 | 7.04 | 0.00 |
| TRINITY_DN91186_c9_g1_i6 | | 2.98 | 0.23 | 3.41 | 0.00 |
| TRINITY_DN91223_c1_g1_i1 | | 0.67 | 5.66 | -3.52 | 0.00 |
| TRINITY_DN91254_c0_g1_i1 | NACA | 0.00 | 2.95 | -6.59 | 0.00 |
| TRINITY_DN91254_c0_g1_i5 | NACA | 0.00 | 15.66 | -9.87 | 0.00 |
| TRINITY_DN91254_c0_g1_i7 | NACA | 0.00 | 98.16 | -12.00 | 0.00 |
| TRINITY_DN91274_c6_g1_i1 | | 0.04 | 1.38 | -4.75 | 0.00 |
| TRINITY_DN91324_c1_g1_i1 | | 3.14 | 21.59 | -2.85 | 0.00 |
| TRINITY_DN91358_c0_g1_i2 | | 470.89 | 163.31 | 1.49 | 0.00 |
| TRINITY_DN91362_c6_g5_i1 | | 4.97 | 0.74 | 2.60 | 0.00 |
| TRINITY_DN91368_c4_g1_i5 | | 0.00 | 4.19 | -5.72 | 0.00 |
| TRINITY_DN91372_c2_g1_i1 | | 2.18 | 5.62 | -1.42 | 0.00 |
| TRINITY_DN91379_c3_g1_i1 | | 2.41 | 13.12 | -2.46 | 0.00 |
| TRINITY_DN91379_c7_g1_i1 | | 0.68 | 3.20 | -2.31 | 0.00 |
| TRINITY_DN91389_c2_g1_i2 | DOCK5 | 0.52 | 2.75 | -2.52 | 0.00 |
| TRINITY_DN91391_c2_g1_i2 | AEP1 | 0.18 | 1.42 | -2.99 | 0.00 |
| TRINITY_DN91391_c2_g2_i1 | | 4.70 | 26.30 | -2.51 | 0.00 |
| TRINITY_DN91391_c2_g3_i1 | | 2.36 | 8.42 | -1.89 | 0.00 |
| TRINITY_DN91393_c2_g1_i3 | | 0.00 | 1.12 | -6.48 | 0.00 |
| TRINITY_DN91404_c0_g1_i8 | UBAC1 | 2.20 | 13.50 | -2.66 | 0.00 |
| TRINITY_DN91433_c7_g3_i3 | CTF8 | 2.15 | 0.52 | 1.91 | 0.00 |
| TRINITY_DN91439_c8_g2_i16 | ACTY | 0.12 | 1.61 | -3.89 | 0.00 |
| TRINITY_DN91462_c5_g1_i13 | | 2.69 | 33.97 | -3.70 | 0.00 |
| TRINITY_DN91462_c5_g1_i15 | | 0.48 | 12.79 | -3.99 | 0.00 |
| TRINITY_DN91462_c5_g1_i5 | | 0.34 | 15.49 | -4.58 | 0.00 |
| TRINITY_DN91462_c5_g1_i6 | | 2.01 | 43.49 | -4.47 | 0.00 |
| TRINITY_DN91462_c5_g2_i2 | | 0.85 | 24.82 | -5.02 | 0.00 |
| TRINITY_DN91462_c5_g2_i6 | | 0.78 | 35.47 | -5.33 | 0.00 |
| TRINITY_DN91481_c4_g3_i1 | | 3.60 | 8.67 | -1.29 | 0.00 |
| TRINITY_DN91481_c4_g3_i2 | | 8.00 | 16.84 | -1.10 | 0.00 |
| TRINITY_DN91503_c5_g2_i2 | | 4.22 | 0.79 | 2.38 | 0.00 |

| | | | | | |
|---|---|---|---|---|---|
| TRINITY_DN91524_c4_g1_i5 | RTN3 | 17.73 | 9.03 | 0.92 | 0.00 |
| TRINITY_DN91528_c1_g1_i3 | GLT14 | 0.69 | 1.85 | -1.51 | 0.00 |
| TRINITY_DN91559_c1_g4_i1 | MAG | 0.00 | 0.76 | -5.13 | 0.00 |
| TRINITY_DN91628_c0_g1_i4 | I20RA | 0.55 | 1.97 | -1.84 | 0.00 |
| TRINITY_DN91637_c5_g1_i1 | | 2.19 | 0.02 | 6.95 | 0.00 |
| TRINITY_DN91680_c5_g2_i1 | | 0.29 | 1.66 | -2.53 | 0.00 |
| TRINITY_DN91750_c3_g2_i4 | PDIA3 | 0.00 | 1.23 | -6.43 | 0.00 |
| TRINITY_DN91751_c0_g2_i2 | CAZA1 | 21.15 | 12.80 | 0.69 | 0.00 |
| TRINITY_DN91751_c0_g3_i2 | CAZA1 | 34.86 | 23.40 | 0.54 | 0.00 |
| TRINITY_DN91777_c0_g2_i2 | CATK | 11.52 | 28.10 | -1.31 | 0.00 |
| TRINITY_DN91799_c0_g3_i2 | | 39.28 | 63.20 | -0.72 | 0.00 |
| TRINITY_DN91816_c0_g4_i1 | MICA1 | 1.32 | 0.00 | 5.70 | 0.00 |
| TRINITY_DN91827_c0_g2_i3 | | 0.23 | 1.82 | -3.01 | 0.00 |
| TRINITY_DN91838_c6_g2_i1 | | 26.03 | 2.85 | 3.15 | 0.00 |
| TRINITY_DN91865_c2_g1_i11 | GPSM1 | 0.27 | 1.52 | -2.47 | 0.00 |
| TRINITY_DN91874_c7_g3_i1 | | 0.39 | 3.03 | -3.04 | 0.00 |
| TRINITY_DN91889_c3_g1_i1 | | 0.52 | 3.30 | -2.73 | 0.00 |
| TRINITY_DN91914_c1_g1_i2 | ARHG8 | 2.21 | 0.63 | 1.75 | 0.00 |
| TRINITY_DN91947_c2_g1_i2 | NFIL3 | 3.94 | 9.70 | -1.33 | 0.00 |
| TRINITY_DN92000_c1_g1_i4 | ACO11 | 0.00 | 1.32 | -6.43 | 0.00 |
| TRINITY_DN92053_c2_g1_i2 | | 1.19 | 0.37 | 1.61 | 0.00 |
| TRINITY_DN92056_c10_g1_i6 | SVOP | 9.02 | 22.54 | -1.36 | 0.00 |
| TRINITY_DN92061_c5_g1_i5 | ID2 | 46.55 | 70.80 | -0.64 | 0.00 |
| TRINITY_DN92073_c4_g3_i1 | | 0.63 | 2.37 | -1.96 | 0.00 |
| TRINITY_DN92077_c1_g2_i9 | | 4.06 | 0.42 | 3.27 | 0.00 |
| TRINITY_DN92095_c0_g1_i4 | KIF23 | 1.33 | 0.04 | 4.68 | 0.00 |
| TRINITY_DN92106_c1_g1_i1 | BFAR | 0.09 | 1.56 | -3.98 | 0.00 |
| TRINITY_DN92178_c1_g1_i2 | DFP3 | 8.14 | 24.25 | -1.62 | 0.00 |
| TRINITY_DN92211_c0_g1_i5 | LIN7C | 4.59 | 8.33 | -0.90 | 0.00 |
| TRINITY_DN92211_c0_g7_i1 | | 0.44 | 4.78 | -3.58 | 0.00 |
| TRINITY_DN92211_c0_g7_i2 | | 3.35 | 0.12 | 4.15 | 0.00 |
| TRINITY_DN92225_c1_g1_i1 | | 0.69 | 4.56 | -2.75 | 0.00 |
| TRINITY_DN92234_c1_g1_i6 | GPM6B | 0.00 | 3.57 | -6.87 | 0.00 |
| TRINITY_DN92248_c4_g1_i7 | IGSF3 | 0.49 | 2.82 | -2.55 | 0.00 |
| TRINITY_DN92275_c5_g1_i6 | | 0.00 | 1.73 | -6.66 | 0.00 |
| TRINITY_DN92302_c2_g1_i1 | | 22.92 | 72.32 | -1.72 | 0.00 |
| TRINITY_DN92356_c0_g1_i4 | GL8D1 | 0.00 | 1.67 | -6.62 | 0.00 |
| TRINITY_DN92395_c4_g2_i1 | | 0.26 | 1.29 | -2.31 | 0.00 |
| TRINITY_DN92420_c1_g1_i1 | LADD | 1.39 | 14.55 | -3.41 | 0.00 |
| TRINITY_DN92427_c5_g3_i2 | PLA2R | 0.47 | 2.46 | -2.45 | 0.00 |
| TRINITY_DN92428_c1_g2_i1 | | 4.78 | 0.26 | 3.92 | 0.00 |
| TRINITY_DN92442_c2_g5_i1 | | 9.01 | 1.42 | 2.59 | 0.00 |

| | | | | | |
|---|---|---|---|---|---|
| TRINITY_DN92443_c6_g1_i2 | LTC4S | 0.40 | 2.78 | -2.79 | 0.00 |
| TRINITY_DN92474_c4_g1_i2 | PON2 | 3.70 | 7.43 | -1.04 | 0.00 |
| TRINITY_DN92481_c4_g1_i4 | PAR3 | 0.00 | 1.11 | -5.57 | 0.00 |
| TRINITY_DN92491_c0_g1_i1 | BZW1A | 2.60 | 6.47 | -1.36 | 0.00 |
| TRINITY_DN92506_c0_g1_i2 | UHRF1 | 4.20 | 2.00 | 1.02 | 0.00 |
| TRINITY_DN92539_c0_g1_i1 | SYWC | 6.96 | 2.87 | 1.25 | 0.00 |
| TRINITY_DN92588_c0_g3_i1 | PACS1 | 0.01 | 0.84 | -4.90 | 0.00 |
| TRINITY_DN92599_c1_g2_i2 | JUNB | 9.69 | 20.07 | -1.10 | 0.00 |
| TRINITY_DN92626_c2_g4_i1 | UBE2K | 0.00 | 1.56 | -5.45 | 0.00 |
| TRINITY_DN92638_c0_g1_i2 | NUB1 | 0.66 | 5.20 | -3.03 | 0.00 |
| TRINITY_DN92643_c1_g1_i3 | BSDC1 | 0.08 | 4.65 | -5.80 | 0.00 |
| TRINITY_DN92679_c4_g1_i10 | STS | 0.07 | 1.33 | -4.45 | 0.00 |
| TRINITY_DN92698_c6_g2_i6 | MEL2A | 1.38 | 0.21 | 2.88 | 0.00 |
| TRINITY_DN92710_c0_g2_i2 | MUC5B | 0.04 | 2.34 | -5.65 | 0.00 |
| TRINITY_DN92710_c1_g3_i1 | | 0.00 | 7.53 | -7.00 | 0.00 |
| TRINITY_DN92710_c1_g3_i12 | | 1.62 | 27.79 | -4.08 | 0.00 |
| TRINITY_DN92710_c1_g3_i13 | | 0.40 | 11.15 | -4.78 | 0.00 |
| TRINITY_DN92710_c1_g3_i15 | MUC5B | 0.21 | 7.66 | -5.30 | 0.00 |
| TRINITY_DN92710_c1_g3_i19 | | 0.43 | 11.57 | -4.56 | 0.00 |
| TRINITY_DN92710_c1_g3_i20 | MUC5B | 0.50 | 6.67 | -3.78 | 0.00 |
| TRINITY_DN92710_c1_g3_i7 | MUC5A | 0.05 | 2.73 | -4.69 | 0.00 |
| TRINITY_DN92710_c1_g3_i9 | | 0.64 | 32.27 | -5.71 | 0.00 |
| TRINITY_DN92766_c0_g1_i4 | MSLNL | 1.65 | 0.42 | 1.97 | 0.00 |
| TRINITY_DN92769_c8_g2_i10 | AGRG4 | 0.00 | 1.59 | -6.88 | 0.00 |
| TRINITY_DN92779_c5_g1_i4 | PKHG3 | 0.79 | 0.00 | 5.96 | 0.00 |
| TRINITY_DN92781_c1_g5_i1 | TMED2 | 0.00 | 5.45 | -7.79 | 0.00 |
| TRINITY_DN92793_c9_g1_i2 | | 7.51 | 2.16 | 1.74 | 0.00 |
| TRINITY_DN92825_c5_g1_i3 | RC3H1 | 0.02 | 0.35 | -3.78 | 0.00 |
| TRINITY_DN92832_c0_g1_i11 | | 18.59 | 0.80 | 4.24 | 0.00 |
| TRINITY_DN92832_c0_g1_i2 | | 29.31 | 3.32 | 3.08 | 0.00 |
| TRINITY_DN92832_c0_g1_i3 | | 124.87 | 16.86 | 2.84 | 0.00 |
| TRINITY_DN92832_c0_g1_i4 | | 14.12 | 0.92 | 3.82 | 0.00 |
| TRINITY_DN92832_c0_g1_i5 | | 4.38 | 0.24 | 4.20 | 0.00 |
| TRINITY_DN92832_c0_g1_i6 | | 14.27 | 1.37 | 3.31 | 0.00 |
| TRINITY_DN92832_c0_g1_i8 | | 31.35 | 2.92 | 3.36 | 0.00 |
| TRINITY_DN92833_c2_g2_i6 | PTMAA | 139.74 | 234.26 | -0.77 | 0.00 |
| TRINITY_DN92839_c0_g2_i1 | DUS6 | 0.00 | 1.35 | -6.35 | 0.00 |
| TRINITY_DN92839_c0_g2_i2 | DUS6 | 10.72 | 19.80 | -0.91 | 0.00 |
| TRINITY_DN92901_c1_g1_i3 | HMCN2 | 0.01 | 0.63 | -5.73 | 0.00 |
| TRINITY_DN92921_c1_g2_i2 | OLM2B | 2.24 | 0.00 | 7.35 | 0.00 |
| TRINITY_DN92967_c4_g2_i3 | LIPO | 9.54 | 78.05 | -3.05 | 0.00 |
| TRINITY_DN93085_c5_g1_i1 | | 16.46 | 7.80 | 1.01 | 0.00 |

| | | | | | |
|---|---|---|---|---|---|
| TRINITY_DN93110_c0_g1_i1 | STX5 | 1.48 | 0.03 | 5.23 | 0.00 |
| TRINITY_DN93125_c1_g1_i6 | LYPA2 | 10.55 | 6.36 | 0.68 | 0.00 |
| TRINITY_DN93143_c8_g1_i4 | | 0.22 | 3.77 | -4.20 | 0.00 |
| TRINITY_DN93172_c3_g1_i4 | AQP3 | 35.75 | 36.03 | 23.19 | 0.00 |
| TRINITY_DN93239_c1_g1_i13 | AGRP | 0.11 | 2.51 | -4.54 | 0.00 |
| TRINITY_DN93267_c3_g1_i1 | TES | 4.47 | 0.00 | 7.39 | 0.00 |
| TRINITY_DN93289_c5_g3_i1 | SLAP2 | 0.13 | 0.95 | -2.85 | 0.00 |
| TRINITY_DN93321_c2_g1_i8 | | 0.00 | 0.60 | -4.78 | 0.00 |
| TRINITY_DN93384_c2_g2_i10 | | 18.86 | 5.12 | 1.83 | 0.00 |
| TRINITY_DN93417_c7_g3_i4 | | 28.04 | 4.47 | 2.86 | 0.00 |
| TRINITY_DN93433_c3_g1_i1 | | 0.16 | 1.30 | -2.89 | 0.00 |
| TRINITY_DN93447_c5_g3_i2 | | 3.30 | 0.49 | 4.32 | 0.00 |
| TRINITY_DN93447_c5_g3_i4 | | 0.18 | 5.64 | -4.97 | 0.00 |
| TRINITY_DN93468_c0_g1_i4 | RL4B | 0.00 | 7.92 | -7.98 | 0.00 |
| TRINITY_DN93468_c0_g1_i9 | RL4B | 28.35 | 50.30 | -0.86 | 0.00 |
| TRINITY_DN93500_c8_g3_i2 | NHS | 0.00 | 1.13 | -4.95 | 0.00 |
| TRINITY_DN93572_c2_g1_i1 | | 0.17 | 2.27 | -3.58 | 0.00 |
| TRINITY_DN93616_c4_g2_i2 | | 5.37 | 0.37 | 3.62 | 0.00 |
| TRINITY_DN93632_c0_g1_i5 | CNTN1 | 47.58 | 87.01 | -0.89 | 0.00 |
| TRINITY_DN93644_c2_g1_i9 | YIF1A | 1.72 | 0.50 | 1.81 | 0.00 |
| TRINITY_DN93668_c3_g1_i4 | | 18.96 | 3.18 | 2.56 | 0.00 |
| TRINITY_DN93669_c0_g1_i13 | AKA12 | 0.00 | 2.53 | -8.93 | 0.00 |
| TRINITY_DN93669_c0_g1_i5 | AKA12 | 0.75 | 0.00 | 6.10 | 0.00 |
| TRINITY_DN93669_c0_g1_i6 | AKA12 | 0.00 | 1.33 | -7.48 | 0.00 |
| TRINITY_DN93678_c6_g1_i9 | MBTP2 | 0.61 | 0.01 | 4.77 | 0.00 |
| TRINITY_DN93690_c6_g2_i1 | | 0.26 | 1.69 | -2.73 | 0.00 |
| TRINITY_DN93774_c5_g1_i16 | PHLB1 | 0.00 | 0.88 | -6.02 | 0.00 |
| TRINITY_DN93775_c0_g2_i14 | | 8.04 | 25.12 | -1.68 | 0.00 |
| TRINITY_DN93814_c1_g3_i3 | | 0.57 | 9.23 | -4.06 | 0.00 |
| TRINITY_DN93822_c0_g1_i1 | KLF4 | 41.62 | 110.19 | -1.44 | 0.00 |
| TRINITY_DN93822_c0_g1_i5 | KLF2 | 11.50 | 35.34 | -1.65 | 0.00 |
| TRINITY_DN93851_c7_g1_i7 | | 0.00 | 1.31 | -5.82 | 0.00 |
| TRINITY_DN93854_c1_g2_i1 | | 0.17 | 11.56 | -5.13 | 0.00 |
| TRINITY_DN93860_c1_g4_i6 | VGFR2 | 0.02 | 1.49 | -6.02 | 0.00 |
| TRINITY_DN93865_c7_g1_i2 | ADAP1 | 0.25 | 1.82 | -2.98 | 0.00 |
| TRINITY_DN93880_c1_g1_i4 | ARF4 | 5.97 | 0.00 | 7.37 | 0.00 |
| TRINITY_DN93882_c1_g2_i2 | K1C18 | 1.41 | 6.34 | -2.19 | 0.00 |
| TRINITY_DN93887_c1_g1_i6 | TFPI2 | 0.92 | 7.51 | -3.08 | 0.00 |
| TRINITY_DN93894_c0_g3_i1 | CCL20 | 8.39 | 30.53 | -1.90 | 0.00 |
| TRINITY_DN93905_c0_g2_i1 | | 0.03 | 4.45 | -5.77 | 0.00 |
| TRINITY_DN93944_c6_g1_i1 | GYS1 | 3.73 | 1.17 | 1.68 | 0.00 |
| TRINITY_DN93961_c0_g2_i4 | BTG2 | 17.44 | 36.46 | -1.11 | 0.00 |

| | | | | | |
|---|---|---|---|---|---|
| TRINITY_DN93971_c3_g1_i1 | SMAD4 | 0.00 | 4.01 | -5.06 | 0.00 |
| TRINITY_DN93973_c5_g1_i4 | | 0.12 | 2.18 | -4.17 | 0.00 |
| TRINITY_DN93973_c5_g2_i1 | | 2.11 | 7.57 | -1.90 | 0.00 |
| TRINITY_DN93990_c2_g1_i2 | EGR1 | 9.83 | 38.47 | -2.03 | 0.00 |
| TRINITY_DN93990_c2_g1_i4 | EGR1 | 2.39 | 14.39 | -2.62 | 0.00 |
| TRINITY_DN93990_c2_g3_i1 | EGR1 | 9.14 | 30.18 | -1.78 | 0.00 |
| TRINITY_DN94018_c2_g1_i6 | ARMT1 | 0.00 | 0.96 | -6.03 | 0.00 |
| TRINITY_DN94065_c0_g2_i4 | PHB | 12.84 | 7.24 | 0.81 | 0.00 |
| TRINITY_DN94075_c3_g1_i1 | TOP2A | 6.82 | 2.70 | 1.28 | 0.00 |
| TRINITY_DN94122_c1_g1_i4 | | 0.00 | 2.01 | -4.72 | 0.00 |
| TRINITY_DN94194_c4_g1_i4 | OSTC | 37.83 | 23.82 | 0.63 | 0.00 |
| TRINITY_DN94201_c0_g2_i1 | | 0.00 | 25.94 | -5.86 | 0.00 |
| TRINITY_DN94202_c1_g2_i1 | PPID | 13.55 | 6.54 | 1.00 | 0.00 |
| TRINITY_DN94223_c5_g1_i3 | | 0.00 | 10.08 | -7.85 | 0.00 |
| TRINITY_DN94223_c5_g3_i1 | | 246.75 | 69.21 | 1.76 | 0.00 |
| TRINITY_DN94230_c1_g1_i1 | | 108.34 | 72.09 | 0.54 | 0.00 |
| TRINITY_DN94234_c2_g5_i1 | IRF1 | 27.85 | 69.77 | -1.34 | 0.00 |
| TRINITY_DN94273_c0_g3_i5 | PGK | 25.96 | 10.79 | 1.32 | 0.00 |
| TRINITY_DN94324_c7_g2_i6 | AGRG3 | 0.26 | 2.67 | -3.36 | 0.00 |
| TRINITY_DN94340_c0_g3_i1 | RSPRY | 3.01 | 0.07 | 5.43 | 0.00 |
| TRINITY_DN94344_c3_g3_i1 | FGL2 | 18.02 | 39.00 | -1.14 | 0.00 |
| TRINITY_DN94351_c1_g4_i1 | | 2.47 | 27.62 | -3.45 | 0.00 |
| TRINITY_DN94351_c3_g3_i1 | | 0.00 | 4.69 | -22.16 | 0.00 |
| TRINITY_DN94396_c0_g1_i3 | PCKGC | 0.00 | 0.79 | -5.67 | 0.00 |
| TRINITY_DN94450_c2_g2_i5 | IFI44 | 1.79 | 9.90 | -2.47 | 0.00 |
| TRINITY_DN94456_c9_g1_i1 | MRC2 | 1.12 | 7.24 | -2.74 | 0.00 |
| TRINITY_DN94516_c5_g3_i6 | DERM | 2.43 | 0.30 | 2.90 | 0.00 |
| TRINITY_DN94529_c6_g3_i2 | | 0.10 | 17.40 | -5.41 | 0.00 |
| TRINITY_DN94536_c0_g5_i4 | JUNB | 64.91 | 147.85 | -1.25 | 0.00 |
| TRINITY_DN94541_c2_g1_i4 | | 887.08 | 362.46 | 1.24 | 0.00 |
| TRINITY_DN94558_c1_g1_i8 | NFIL3 | 0.02 | 0.55 | -4.40 | 0.00 |
| TRINITY_DN94563_c1_g3_i1 | BARD1 | 0.72 | 0.23 | 1.64 | 0.00 |
| TRINITY_DN94568_c0_g1_i4 | BHE40 | 21.71 | 37.16 | -0.82 | 0.00 |
| TRINITY_DN94602_c3_g1_i7 | | 4.25 | 15.87 | -1.93 | 0.00 |
| TRINITY_DN94611_c6_g1_i1 | | 1.10 | 5.44 | -2.35 | 0.00 |
| TRINITY_DN94628_c4_g2_i1 | | 0.95 | 5.05 | -2.47 | 0.00 |
| TRINITY_DN94629_c9_g5_i1 | | 0.55 | 4.44 | -2.91 | 0.00 |
| TRINITY_DN94661_c0_g2_i5 | | 0.24 | 19.36 | -5.35 | 0.00 |
| TRINITY_DN94675_c0_g1_i4 | IMA1 | 7.46 | 2.63 | 1.45 | 0.00 |
| TRINITY_DN94679_c3_g1_i1 | | 0.11 | 58.90 | -7.78 | 0.00 |
| TRINITY_DN94679_c3_g2_i2 | | 0.04 | 4.81 | -6.08 | 0.00 |
| TRINITY_DN94698_c2_g2_i1 | | 4.60 | 7.65 | -0.76 | 0.00 |

| | | | | | |
|---|---|---|---|---|---|
| TRINITY_DN94726_c3_g1_i1 | | 112.60 | 15.71 | 2.92 | 0.00 |
| TRINITY_DN94726_c9_g1_i6 | PRS27 | 4.80 | 0.15 | 5.12 | 0.00 |
| TRINITY_DN94726_c9_g1_i9 | PRS27 | 64.56 | 4.16 | 4.04 | 0.00 |
| TRINITY_DN94744_c0_g1_i5 | ITM2B | 93.70 | 138.52 | -0.61 | 0.00 |
| TRINITY_DN94744_c0_g5_i5 | ITM2B | 43.36 | 59.47 | -0.48 | 0.00 |
| TRINITY_DN94783_c2_g2_i2 | | 2.86 | 1.50 | 0.89 | 0.00 |
| TRINITY_DN94788_c0_g2_i4 | RNF13 | 3.39 | 0.00 | 7.46 | 0.00 |
| TRINITY_DN94802_c4_g2_i4 | | 2.37 | 21.56 | -3.21 | 0.00 |
| TRINITY_DN94840_c0_g2_i3 | RSPRY | 1.23 | 0.00 | 5.32 | 0.00 |
| TRINITY_DN94883_c4_g4_i2 | K1C13 | 3.00 | 19.88 | -2.69 | 0.00 |
| TRINITY_DN94891_c5_g4_i1 | ZN706 | 108.17 | 73.08 | 0.52 | 0.00 |
| TRINITY_DN94919_c1_g2_i15 | F122A | 1.17 | 0.07 | 4.33 | 0.00 |
| TRINITY_DN94925_c5_g1_i1 | | 0.49 | 5.55 | -3.29 | 0.00 |
| TRINITY_DN94957_c3_g7_i1 | | 59.06 | 89.54 | -0.65 | 0.00 |
| TRINITY_DN94966_c1_g1_i2 | TISB | 26.25 | 59.10 | -1.21 | 0.00 |
| TRINITY_DN94966_c1_g1_i4 | TISB | 33.65 | 67.95 | -1.06 | 0.00 |
| TRINITY_DN94966_c1_g1_i5 | TISB | 36.13 | 73.26 | -1.08 | 0.00 |
| TRINITY_DN94966_c1_g1_i7 | TISB | 22.88 | 48.71 | -1.14 | 0.00 |
| TRINITY_DN94979_c2_g4_i1 | M1I1B | 20.99 | 49.31 | -1.28 | 0.00 |
| TRINITY_DN94979_c2_g4_i2 | M1I1B | 18.55 | 44.27 | -1.29 | 0.00 |
| TRINITY_DN94991_c2_g1_i5 | | 0.00 | 1.89 | -6.69 | 0.00 |
| TRINITY_DN94992_c2_g1_i4 | | 6.52 | 0.52 | 3.59 | 0.00 |
| TRINITY_DN95055_c5_g8_i1 | | 17.47 | 0.67 | 4.10 | 0.00 |
| TRINITY_DN95109_c5_g1_i2 | B2MG | 70.77 | 121.35 | -0.80 | 0.00 |
| TRINITY_DN95127_c1_g1_i6 | | 0.15 | 1.67 | -3.76 | 0.00 |
| TRINITY_DN95129_c0_g1_i1 | RHGBA | 1.64 | 0.66 | 1.24 | 0.00 |
| TRINITY_DN95146_c0_g1_i19 | UAP1 | 1.08 | 0.05 | 4.26 | 0.00 |
| TRINITY_DN95177_c1_g1_i7 | IGM | 0.05 | 2.92 | -6.27 | 0.00 |
| TRINITY_DN95218_c0_g3_i11 | TP53B | 0.87 | 0.00 | 5.53 | 0.00 |
| TRINITY_DN95220_c3_g1_i1 | ENOF1 | 1.02 | 6.21 | -2.65 | 0.00 |
| TRINITY_DN95238_c0_g1_i4 | MUC2 | 1.53 | 60.55 | -5.35 | 0.00 |
| TRINITY_DN95253_c3_g3_i1 | LDB1 | 0.12 | 8.46 | -4.58 | 0.00 |
| TRINITY_DN95255_c1_g1_i3 | CIRBB | 10.66 | 2.50 | 2.02 | 0.00 |
| TRINITY_DN95256_c2_g1_i11 | AGRG7 | 0.15 | 2.01 | -3.85 | 0.00 |
| TRINITY_DN95261_c2_g1_i3 | | 4.78 | 0.00 | 7.47 | 0.00 |
| TRINITY_DN95263_c4_g1_i6 | SKAP2 | 0.34 | 2.05 | -2.52 | 0.00 |
| TRINITY_DN95265_c1_g1_i4 | TPL2B | 0.00 | 6.00 | -8.10 | 0.00 |
| TRINITY_DN95309_c8_g1_i1 | | 145.66 | 39.94 | 1.82 | 0.00 |
| TRINITY_DN95309_c8_g1_i5 | | 2.69 | 0.23 | 3.58 | 0.00 |
| TRINITY_DN95309_c8_g1_i8 | | 85.82 | 23.44 | 1.85 | 0.00 |
| TRINITY_DN95309_c8_g1_i9 | | 24.08 | 8.39 | 1.46 | 0.00 |
| TRINITY_DN95313_c0_g1_i6 | COG8 | 1.44 | 0.04 | 5.00 | 0.00 |

| | | | | | |
|---|---|---|---|---|---|
| TRINITY_DN95328_c1_g1_i4 | PRC1 | 0.00 | 1.02 | -5.99 | 0.00 |
| TRINITY_DN95357_c1_g2_i3 | AT1A2 | 12.82 | 0.95 | 3.46 | 0.00 |
| TRINITY_DN95376_c2_g1_i1 | | 0.00 | 2.50 | -6.06 | 0.00 |
| TRINITY_DN95430_c0_g1_i3 | KIN17 | 1.89 | 0.10 | 3.92 | 0.00 |
| TRINITY_DN95454_c0_g2_i9 | | 0.69 | 12.70 | -4.19 | 0.00 |
| TRINITY_DN95468_c0_g1_i3 | PLK2 | 8.07 | 14.63 | -0.90 | 0.00 |
| TRINITY_DN95488_c1_g1_i2 | | 0.00 | 6.15 | -6.42 | 0.00 |
| TRINITY_DN95515_c0_g1_i13 | RSSA | 0.42 | 7.85 | -4.13 | 0.00 |
| TRINITY_DN95519_c3_g1_i3 | ASNA | 0.00 | 1.55 | -6.06 | 0.00 |
| TRINITY_DN95539_c4_g2_i1 | CCR6 | 0.42 | 2.45 | -2.67 | 0.00 |
| TRINITY_DN95545_c0_g1_i1 | | 0.00 | 10.96 | -6.76 | 0.00 |
| TRINITY_DN95545_c0_g1_i2 | | 0.22 | 11.10 | -5.85 | 0.00 |
| TRINITY_DN95572_c12_g1_i1 | | 1.07 | 3.05 | -1.57 | 0.00 |
| TRINITY_DN95610_c1_g1_i2 | PTBP2 | 7.43 | 0.32 | 4.31 | 0.00 |
| TRINITY_DN95632_c8_g1_i4 | | 0.06 | 3.79 | -4.55 | 0.00 |
| TRINITY_DN95634_c1_g1_i8 | RPN1 | 16.54 | 9.98 | 0.68 | 0.00 |
| TRINITY_DN95635_c0_g4_i3 | ZN106 | 0.46 | 0.00 | 5.42 | 0.00 |
| TRINITY_DN95672_c1_g2_i14 | HGS | 1.79 | 0.04 | 5.28 | 0.00 |
| TRINITY_DN95677_c0_g1_i1 | | 8.13 | 11.90 | -0.60 | 0.00 |
| TRINITY_DN95699_c0_g1_i5 | SYCC | 2.40 | 0.05 | 5.29 | 0.00 |
| TRINITY_DN95724_c14_g1_i2 | | 2.13 | 0.00 | 5.33 | 0.00 |
| TRINITY_DN95731_c3_g1_i5 | | 0.00 | 2.06 | -7.83 | 0.00 |
| TRINITY_DN95741_c1_g1_i22 | NUCL | 8.38 | 0.00 | 8.54 | 0.00 |
| TRINITY_DN95784_c1_g1_i2 | EGR1 | 14.95 | 48.90 | -1.78 | 0.00 |
| TRINITY_DN95784_c2_g1_i1 | | 2.86 | 12.58 | -2.17 | 0.00 |
| TRINITY_DN95784_c2_g2_i1 | | 17.20 | 61.67 | -1.91 | 0.00 |
| TRINITY_DN95856_c1_g1_i19 | PFKAP | 1.04 | 0.00 | 4.98 | 0.00 |
| TRINITY_DN95869_c1_g1_i2 | ARRD2 | 6.88 | 13.62 | -1.02 | 0.00 |
| TRINITY_DN95886_c5_g1_i1 | | 3.63 | 10.58 | -1.54 | 0.00 |
| TRINITY_DN95891_c1_g1_i1 | PAXI | 0.89 | 0.00 | 4.70 | 0.00 |
| TRINITY_DN95924_c5_g1_i3 | PLDX1 | 0.91 | 0.00 | 4.46 | 0.00 |
| TRINITY_DN95934_c0_g1_i1 | IL1B | 1.91 | 6.22 | -1.75 | 0.00 |
| TRINITY_DN95934_c0_g1_i2 | IL1B | 4.85 | 18.48 | -1.98 | 0.00 |
| TRINITY_DN95961_c0_g2_i1 | CR3L3 | 0.00 | 16.94 | -6.31 | 0.00 |
| TRINITY_DN95976_c3_g5_i1 | | 0.41 | 3.51 | -3.12 | 0.00 |
| TRINITY_DN95988_c1_g3_i1 | | 0.40 | 13.04 | -4.91 | 0.00 |
| TRINITY_DN95989_c0_g1_i14 | | 14.49 | 2.55 | 2.51 | 0.00 |
| TRINITY_DN95993_c1_g1_i16 | KI20A | 0.73 | 0.00 | 5.71 | 0.00 |
| TRINITY_DN95993_c1_g2_i4 | MCM7 | 13.46 | 6.80 | 0.93 | 0.00 |
| TRINITY_DN95996_c3_g2_i2 | PTX3 | 0.59 | 9.52 | -4.15 | 0.00 |
| TRINITY_DN96003_c0_g1_i5 | EPN3 | 0.80 | 0.00 | 5.64 | 0.00 |
| TRINITY_DN96005_c1_g2_i7 | SC31A | 2.78 | 1.16 | 1.21 | 0.00 |

| | | | | | |
|---|---|---|---|---|---|
| TRINITY_DN96013_c5_g1_i1 | | 3.20 | 0.08 | 4.98 | 0.00 |
| TRINITY_DN96022_c0_g1_i1 | CEBPA | 15.80 | 26.94 | -0.80 | 0.00 |
| TRINITY_DN96077_c4_g1_i10 | ICOSL | 4.33 | 0.01 | 8.00 | 0.00 |
| TRINITY_DN96077_c4_g1_i4 | ICOSL | 0.87 | 0.01 | 5.87 | 0.00 |
| TRINITY_DN96128_c2_g4_i6 | ZDHC2 | 0.04 | 1.05 | -4.87 | 0.00 |
| TRINITY_DN96191_c0_g1_i2 | TOB1 | 20.79 | 40.37 | -1.00 | 0.00 |
| TRINITY_DN96204_c3_g1_i3 | | 0.00 | 1.36 | -6.03 | 0.00 |
| TRINITY_DN96225_c9_g2_i4 | | 0.10 | 3.20 | -4.57 | 0.00 |
| TRINITY_DN96228_c3_g1_i4 | SC5A3 | 0.55 | 0.00 | 5.39 | 0.00 |
| TRINITY_DN96255_c5_g1_i5 | ACTS | 38.33 | 137.23 | -1.89 | 0.00 |
| TRINITY_DN96327_c0_g3_i1 | MCM5 | 7.29 | 2.94 | 1.25 | 0.00 |
| TRINITY_DN96364_c2_g1_i1 | | 0.21 | 1.50 | -2.86 | 0.00 |
| TRINITY_DN96413_c1_g6_i2 | | 12.62 | 19.82 | -0.69 | 0.00 |
| TRINITY_DN96449_c3_g1_i3 | RFLA | 1.02 | 0.00 | 6.26 | 0.00 |
| TRINITY_DN96457_c8_g2_i19 | | 1.59 | 0.00 | 6.14 | 0.00 |
| TRINITY_DN96477_c1_g1_i4 | TOM70 | 9.06 | 4.45 | 0.97 | 0.00 |
| TRINITY_DN96485_c6_g1_i1 | | 9.75 | 1.94 | 2.24 | 0.00 |
| TRINITY_DN96534_c2_g1_i2 | | 2.38 | 8.03 | -1.78 | 0.00 |
| TRINITY_DN96636_c0_g3_i1 | | 11.41 | 27.49 | -1.31 | 0.00 |
| TRINITY_DN96670_c5_g3_i7 | STA13 | 0.00 | 0.99 | -6.83 | 0.00 |
| TRINITY_DN96679_c4_g1_i1 | | 2.50 | 0.65 | 1.87 | 0.00 |
| TRINITY_DN96736_c0_g3_i1 | FUCL4 | 1.90 | 9.98 | -2.41 | 0.00 |
| TRINITY_DN96737_c1_g1_i8 | APOEB | 517.37 | 787.03 | -0.64 | 0.00 |
| TRINITY_DN96747_c10_g3_i1 | SHSA4 | 0.00 | 3.61 | -6.58 | 0.00 |
| TRINITY_DN96755_c6_g1_i2 | GIT2 | 0.08 | 0.78 | -3.37 | 0.00 |
| TRINITY_DN96758_c4_g1_i5 | GT2D2 | 1.68 | 0.00 | 6.80 | 0.00 |
| TRINITY_DN96779_c2_g3_i2 | ANX2A | 0.00 | 12.98 | -8.58 | 0.00 |
| TRINITY_DN96820_c2_g1_i4 | K0907 | 2.19 | 0.12 | 4.16 | 0.00 |
| TRINITY_DN96847_c10_g3_i1 | MAFK | 4.85 | 10.04 | -1.07 | 0.00 |
| TRINITY_DN96867_c2_g3_i2 | | 1.53 | 0.16 | 3.11 | 0.00 |
| TRINITY_DN96868_c0_g1_i1 | GRP75 | 12.08 | 6.51 | 0.85 | 0.00 |
| TRINITY_DN96881_c1_g1_i1 | ARPC2 | 40.58 | 21.34 | 0.89 | 0.00 |
| TRINITY_DN96894_c5_g3_i2 | | 0.05 | 2.01 | -5.17 | 0.00 |
| TRINITY_DN96908_c1_g2_i1 | METK1 | 8.50 | 4.74 | 0.81 | 0.00 |
| TRINITY_DN96926_c1_g1_i8 | DDX18 | 0.10 | 3.43 | -5.19 | 0.00 |
| TRINITY_DN96950_c5_g1_i1 | | 0.52 | 5.21 | -3.34 | 0.00 |
| TRINITY_DN96984_c0_g1_i5 | T106B | 0.78 | 0.69 | -5.65 | 0.00 |
| TRINITY_DN97018_c3_g1_i4 | HNRPK | 2.62 | 0.30 | 3.56 | 0.00 |
| TRINITY_DN97019_c10_g3_i1 | | 0.81 | 2.92 | -1.96 | 0.00 |
| TRINITY_DN97036_c0_g1_i4 | CD68 | 0.00 | 12.74 | -9.06 | 0.00 |
| TRINITY_DN97036_c0_g1_i6 | CD68 | 0.16 | 6.78 | -5.37 | 0.00 |
| TRINITY_DN97087_c3_g2_i2 | KLH24 | 0.00 | 0.90 | -6.04 | 0.00 |

| | | | | | |
|---|---|---|---|---|---|
| TRINITY_DN97133_c2_g1_i1 | | 2.34 | 0.78 | 1.49 | 0.00 |
| TRINITY_DN97143_c3_g1_i5 | PCAT1 | 0.51 | 0.02 | 3.65 | 0.00 |
| TRINITY_DN97143_c3_g1_i8 | PCAT1 | 2.28 | 0.29 | 3.09 | 0.00 |
| TRINITY_DN97143_c3_g3_i1 | | 0.59 | 5.03 | -3.17 | 0.00 |
| TRINITY_DN97145_c5_g1_i2 | APOD | 22.98 | 0.93 | 4.81 | 0.00 |
| TRINITY_DN97186_c1_g1_i13 | PIM1 | 13.48 | 33.42 | -1.37 | 0.00 |
| TRINITY_DN97186_c1_g1_i7 | PIM1 | 16.77 | 42.07 | -1.38 | 0.00 |
| TRINITY_DN97187_c0_g1_i10 | KCTD3 | 0.00 | 0.88 | -7.11 | 0.00 |
| TRINITY_DN97191_c0_g1_i6 | TSP1 | 0.11 | 1.28 | -3.51 | 0.00 |
| TRINITY_DN97207_c1_g1_i4 | A33 | 0.00 | 0.66 | -5.03 | 0.00 |
| TRINITY_DN97230_c8_g1_i1 | | 0.23 | 3.13 | -3.65 | 0.00 |
| TRINITY_DN97240_c4_g1_i9 | CATC | 15.70 | 8.94 | 0.77 | 0.00 |
| TRINITY_DN97261_c0_g2_i1 | FUBP1 | 30.72 | 10.38 | 1.51 | 0.00 |
| TRINITY_DN97292_c0_g1_i10 | ATF3 | 4.18 | 19.68 | -2.22 | 0.00 |
| TRINITY_DN97292_c0_g3_i1 | ATF3 | 3.13 | 17.07 | -2.45 | 0.00 |
| TRINITY_DN97339_c1_g1_i6 | MTRR | 1.08 | 0.00 | 5.62 | 0.00 |
| TRINITY_DN97347_c3_g1_i1 | | 0.03 | 0.92 | -4.70 | 0.00 |
| TRINITY_DN97392_c4_g5_i1 | | 0.00 | 7.72 | -6.59 | 0.00 |
| TRINITY_DN97455_c5_g1_i1 | | 19.41 | 4.10 | 2.14 | 0.00 |
| TRINITY_DN97491_c1_g1_i8 | KDM1A | 0.17 | 3.38 | -4.44 | 0.00 |
| TRINITY_DN97531_c1_g1_i12 | FOSB | 0.19 | 3.11 | -4.07 | 0.00 |
| TRINITY_DN97541_c2_g1_i2 | | 46.88 | 110.64 | -1.30 | 0.00 |
| TRINITY_DN97541_c2_g2_i1 | | 50.10 | 135.19 | -1.48 | 0.00 |
| TRINITY_DN97541_c2_g3_i1 | | 10.18 | 54.62 | -2.46 | 0.00 |
| TRINITY_DN97550_c8_g1_i1 | | 0.86 | 5.21 | -2.63 | 0.00 |
| TRINITY_DN97557_c4_g6_i1 | TCB1 | 0.31 | 4.52 | -3.56 | 0.00 |
| TRINITY_DN97574_c7_g1_i1 | | 25.81 | 9.02 | 1.50 | 0.00 |
| TRINITY_DN97580_c0_g1_i6 | KPCD | 3.01 | 0.14 | 4.36 | 0.00 |
| TRINITY_DN97613_c4_g1_i1 | IER2 | 79.66 | 188.00 | -1.30 | 0.00 |
| TRINITY_DN97625_c0_g2_i3 | PRRX1 | 0.02 | 3.95 | -7.86 | 0.00 |
| TRINITY_DN97672_c0_g1_i9 | MBP | 2.14 | 0.02 | 5.74 | 0.00 |
| TRINITY_DN97698_c4_g2_i1 | | 0.38 | 4.57 | -3.68 | 0.00 |
| TRINITY_DN97722_c3_g1_i1 | OAT | 9.30 | 3.53 | 1.42 | 0.00 |
| TRINITY_DN97800_c5_g1_i3 | AGRF3 | 0.50 | 3.37 | -2.79 | 0.00 |
| TRINITY_DN97823_c1_g2_i6 | CNDH2 | 1.65 | 0.31 | 2.28 | 0.00 |
| TRINITY_DN97825_c1_g3_i1 | GIPC1 | 0.21 | 4.06 | -4.19 | 0.00 |
| TRINITY_DN97879_c2_g1_i1 | E2F8 | 2.52 | 0.46 | 2.46 | 0.00 |
| TRINITY_DN97883_c1_g1_i23 | | 4.10 | 0.67 | 2.68 | 0.00 |
| TRINITY_DN97910_c1_g1_i5 | B4GT4 | 2.31 | 4.26 | -0.91 | 0.00 |
| TRINITY_DN97922_c5_g1_i1 | CENPW | 3.60 | 1.31 | 1.42 | 0.00 |
| TRINITY_DN97945_c1_g2_i2 | KTAP2 | 9.00 | 4.48 | 0.97 | 0.00 |
| TRINITY_DN97952_c1_g1_i1 | CBPZ | 0.00 | 1.11 | -6.68 | 0.00 |

| | | | | | |
|---|---|---|---|---|---|
| TRINITY_DN97952_c1_g1_i10 | CBPZ | 0.00 | 1.65 | -6.83 | 0.00 |
| TRINITY_DN97955_c5_g2_i1 | | 3.33 | 0.26 | 3.74 | 0.00 |
| TRINITY_DN97958_c0_g1_i1 | | 5.12 | 11.82 | -1.23 | 0.00 |
| TRINITY_DN98010_c2_g1_i2 | | 0.00 | 14.11 | -8.37 | 0.00 |
| TRINITY_DN98014_c1_g1_i5 | CH60 | 9.15 | 4.04 | 1.15 | 0.00 |
| TRINITY_DN98032_c4_g2_i3 | EIF3B | 21.83 | 12.24 | 0.81 | 0.00 |
| TRINITY_DN98041_c2_g1_i2 | | 0.06 | 1.45 | -4.22 | 0.00 |
| TRINITY_DN98054_c3_g1_i3 | MK03 | 0.00 | 1.51 | -22.24 | 0.00 |
| TRINITY_DN98081_c7_g2_i1 | | 47.75 | 135.15 | -1.55 | 0.00 |
| TRINITY_DN98163_c2_g2_i1 | | 22.33 | 1.93 | 3.53 | 0.00 |
| TRINITY_DN98163_c3_g1_i2 | ANXA4 | 0.00 | 6.53 | -8.63 | 0.00 |
| TRINITY_DN98172_c3_g1_i9 | ENPP1 | 1.45 | 0.09 | 3.75 | 0.00 |
| TRINITY_DN98209_c5_g1_i1 | BCAT1 | 9.12 | 4.64 | 0.93 | 0.00 |
| TRINITY_DN98221_c2_g1_i2 | GRHL1 | 0.03 | 2.97 | -5.68 | 0.00 |
| TRINITY_DN98231_c1_g2_i7 | LIMD1 | 0.00 | 0.38 | -5.24 | 0.00 |
| TRINITY_DN98236_c0_g1_i10 | EF1A2 | 53.45 | 71.82 | -1.56 | 0.00 |
| TRINITY_DN98306_c0_g2_i10 | | 0.70 | 0.00 | 5.17 | 0.00 |
| TRINITY_DN98312_c1_g2_i1 | CCNG1 | 13.53 | 16.27 | -1.35 | 0.00 |
| TRINITY_DN98347_c7_g1_i10 | LIPO | 2.02 | 0.00 | 6.52 | 0.00 |
| TRINITY_DN98347_c7_g1_i17 | LIPO | 18.44 | 156.21 | -3.10 | 0.00 |
| TRINITY_DN98407_c2_g1_i9 | PGH2 | 3.84 | 17.45 | -2.24 | 0.00 |
| TRINITY_DN98453_c1_g2_i3 | | 3.36 | 0.11 | 4.83 | 0.00 |
| TRINITY_DN98453_c1_g2_i4 | | 2.49 | 0.00 | 5.74 | 0.00 |
| TRINITY_DN98584_c0_g2_i1 | CK5P2 | 0.45 | 0.00 | 6.63 | 0.00 |
| TRINITY_DN98608_c12_g2_i2 | | 2.00 | 7.88 | -2.03 | 0.00 |
| TRINITY_DN98613_c4_g1_i4 | MARE1 | 0.00 | 0.87 | -5.48 | 0.00 |
| TRINITY_DN98616_c1_g2_i1 | | 22.07 | 10.71 | 1.01 | 0.00 |
| TRINITY_DN98627_c5_g1_i2 | | 4.48 | 0.10 | 5.64 | 0.00 |
| TRINITY_DN98654_c1_g1_i2 | ZN395 | 3.54 | 5.93 | -0.77 | 0.00 |
| TRINITY_DN98662_c1_g1_i4 | GABP2 | 162.06 | 347.57 | -1.15 | 0.00 |
| TRINITY_DN98684_c1_g1_i2 | TNPO3 | 1.92 | 0.75 | 1.28 | 0.00 |
| TRINITY_DN98752_c2_g1_i5 | CTCF | 0.00 | 2.63 | -6.99 | 0.00 |
| TRINITY_DN98785_c0_g4_i2 | | 0.20 | 2.37 | -3.50 | 0.00 |
| TRINITY_DN98796_c0_g1_i17 | PRP39 | 0.00 | 3.01 | -8.10 | 0.00 |
| TRINITY_DN98796_c0_g1_i9 | PRP39 | 0.00 | 3.18 | -8.18 | 0.00 |
| TRINITY_DN98814_c0_g1_i2 | | 3.11 | 5.55 | -0.87 | 0.00 |
| TRINITY_DN98828_c1_g1_i2 | AGRG1 | 0.47 | 4.18 | -3.18 | 0.00 |
| TRINITY_DN98876_c8_g4_i2 | | 0.00 | 1.97 | -5.82 | 0.00 |
| TRINITY_DN98880_c1_g1_i3 | CNNM3 | 0.00 | 1.04 | -6.75 | 0.00 |
| TRINITY_DN98896_c7_g1_i4 | | 6.22 | 4.47 | -2.15 | 0.00 |
| TRINITY_DN98900_c0_g4_i16 | SKT | 0.03 | 0.37 | -3.95 | 0.00 |
| TRINITY_DN98903_c1_g1_i6 | | 26.05 | 66.12 | -1.40 | 0.00 |

| | | | | | |
|---|---|---|---|---|---|
| TRINITY_DN98914_c2_g1_i3 | RIR2 | 29.02 | 10.39 | 1.42 | 0.00 |
| TRINITY_DN98914_c3_g1_i5 | | 0.00 | 1.39 | -5.79 | 0.00 |
| TRINITY_DN98997_c0_g1_i5 | CO6A1 | 3.52 | 39.13 | -3.54 | 0.00 |
| TRINITY_DN99029_c2_g7_i1 | | 3.36 | 6.77 | -1.08 | 0.00 |
| TRINITY_DN99032_c0_g1_i1 | ITA4 | 0.03 | 1.93 | -6.42 | 0.00 |
| TRINITY_DN99057_c5_g2_i2 | MEF2C | 0.95 | 0.02 | 5.12 | 0.00 |
| TRINITY_DN99063_c10_g1_i4 | HDAC6 | 0.00 | 2.92 | -6.58 | 0.00 |
| TRINITY_DN99064_c0_g1_i4 | SORT1 | 2.20 | 4.95 | -1.20 | 0.00 |
| TRINITY_DN99075_c3_g1_i6 | KPCD3 | 3.02 | 4.81 | -0.70 | 0.00 |
| TRINITY_DN99087_c0_g1_i2 | | 1.15 | 4.29 | -1.95 | 0.00 |
| TRINITY_DN99092_c3_g2_i6 | CATH | 0.13 | 4.45 | -4.59 | 0.00 |
| TRINITY_DN99135_c2_g1_i4 | TBC17 | 0.90 | 5.92 | -3.02 | 0.00 |
| TRINITY_DN99156_c0_g1_i6 | PDCD4 | 5.63 | 7.66 | -0.48 | 0.00 |
| TRINITY_DN99187_c1_g1_i4 | SPON1 | 0.24 | 6.05 | -4.70 | 0.00 |
| TRINITY_DN99242_c6_g2_i3 | | 8.87 | 19.06 | -1.14 | 0.00 |
| TRINITY_DN99265_c0_g3_i1 | CBS | 6.69 | 3.94 | 0.73 | 0.00 |
| TRINITY_DN99275_c4_g1_i4 | S61A2 | 8.13 | 4.36 | 0.86 | 0.00 |
| TRINITY_DN99385_c6_g2_i4 | | 0.57 | 3.35 | -2.64 | 0.00 |
| TRINITY_DN99440_c1_g1_i15 | K1468 | 0.45 | 2.43 | -2.41 | 0.00 |
| TRINITY_DN99441_c0_g1_i1 | MSLNL | 2.84 | 0.50 | 2.50 | 0.00 |
| TRINITY_DN99476_c3_g2_i1 | KLF6 | 22.36 | 36.01 | -0.73 | 0.00 |
| TRINITY_DN99575_c4_g1_i3 | RIR1 | 10.53 | 4.56 | 1.17 | 0.00 |
| TRINITY_DN99598_c4_g1_i4 | IF2B3 | 0.71 | 0.04 | 3.73 | 0.00 |
| TRINITY_DN99634_c0_g4_i2 | STT3A | 12.88 | 6.41 | 0.95 | 0.00 |
| TRINITY_DN99642_c3_g2_i7 | MMP2 | 0.26 | 52.23 | -7.62 | 0.00 |
| TRINITY_DN99669_c0_g2_i10 | S6A13 | 0.58 | 3.35 | -2.58 | 0.00 |
| TRINITY_DN99781_c4_g1_i16 | CKLF3 | 2.76 | 5.38 | -0.99 | 0.00 |
| TRINITY_DN99817_c2_g2_i1 | ABCAC | 0.70 | 0.16 | 2.07 | 0.00 |
| TRINITY_DN99817_c2_g2_i2 | ABCAC | 3.14 | 1.22 | 1.37 | 0.00 |
| TRINITY_DN99823_c2_g5_i3 | TBAT | 32.52 | 19.08 | 0.72 | 0.00 |
| TRINITY_DN99825_c0_g3_i1 | CCER1 | 42.93 | 66.39 | -0.67 | 0.00 |
| TRINITY_DN99892_c0_g1_i4 | TNR6B | 0.02 | 0.75 | -5.57 | 0.00 |
| TRINITY_DN99929_c1_g1_i1 | | 0.14 | 3.20 | -4.07 | 0.00 |
| TRINITY_DN99930_c2_g1_i7 | CDN1B | 16.28 | 26.51 | -0.73 | 0.00 |
| TRINITY_DN99937_c2_g3_i1 | | 6.16 | 1.99 | 1.56 | 0.00 |
| TRINITY_DN99955_c1_g1_i13 | CO6A2 | 8.65 | 25.13 | -1.58 | 0.00 |
| TRINITY_DN99955_c1_g1_i8 | CO6A2 | 0.40 | 14.47 | -5.22 | 0.00 |
| TRINITY_DN99955_c1_g2_i1 | CO6A2 | 6.20 | 21.60 | -1.87 | 0.00 |
| TRINITY_DN99958_c1_g2_i1 | SYNE2 | 0.00 | 1.14 | -8.44 | 0.00 |
| TRINITY_DN99980_c4_g1_i10 | SRC8 | 0.00 | 11.73 | -9.65 | 0.00 |
| TRINITY_DN99987_c3_g1_i4 | | 0.71 | 12.63 | -3.94 | 0.00 |

**Table S3.** The significantly enriched pathways involving differentially expressed genes of the **gill** of HT vs NT ($P < 0.01$)

| KEGG pathway | Gene number | Background number | Rich factor | *P*-value | Gene enriched |
|---|---|---|---|---|---|
| Cytokine-cytokine receptor interaction | 32 | 165 | 0.19 | 1E-20 | IL7RA, IL21R, TNF14, CCR9, CXCR4, CCL20, TNR9, TNR5, IFNG, VEGFD, CXL14, CCR5, IL8, IL2RB, I13R2, XCR1, IL6ST, FLT3, CCR6, IL6RA, CCL19, I12R2, TNFA, IL2RG, GHR, I20RA, I20RB, TGFB1, CCL4, TR11B, I22R2, CCR7 |
| Cell adhesion molecules (CAMs) | 20 | 137 | 0.15 | 2E-11 | CLDY, CLD4, L1CAM, TNR5, HA1K, NECT3, HA2B, CLD6, CLD3, ICOSL, HMR1, CD276, CD2, PTPRC, HB2D, ITB2, H2-Ea, CTLA4, CD226, OCLN |
| Focal adhesion | 25 | 245 | 0.10 | 7E-11 | LAMC3, CO2A1, CO1A1, CO9A3, FLNA, FYN, FLNC, PAXI, CO1A2, LAMB1, TSP4B, FYNB, BIR, RAC2, CO4A2, PK3CG, DOCK2, PK3CD, ITB5, TGFI1, CTNB1, VASP, VEGFD, ACTN1, LAMB4, VAV |
| ECM-receptor interaction | 12 | 80 | 0.15 | 1E-09 | CO2A1, CO9A3, CO1A1, CO1A2, LAMB1, TSP4B, LAMC3, GP1BB, ITB5, LAMB4, CO4A2, SETVS |
| Phagosome | 20 | 156 | 0.13 | 2E-08 | NCF1, ITB2, ITB5, HA1K, CLC4M, HA2B, TSP4B, H2-Ea, HMR1, RAB5A, COR1A, HB2D, CO3, TBA3, COL12, ABCB9, CY24B, TAP2, CATS, NCF4, |
| Arachidonic acid metabolism | 11 | 51 | 0.22 | 3E-08 | PTGIS, LOX5, LIPO, LCNL1, LKHA4, PA24F, LTC4S, PGH2, PA24A, PTGES, LOX12 |
| AGE-RAGE signaling pathway in diabetic complications | 14 | 124 | 0.11 | 1E-07 | CO1A1, TGFB1, CO1A2, CDN1B, PK3CG, MMP2, PK3CD, PLCG2, CY24B, PIM1, PLCB3, VEGFD, IL8, CO4A2 |
| Herpes simplex infection | 21 | 183 | 0.11 | 1E-06 | TNF14, IRF3, HA1K, NECT3, IFNG, HCFC1, HB2D, HG2A, TRAF3, IKBA, HMR1, CO3, H2-Ea, HA2B, ABCB9, TAP2, TRAF2, TNFA, JAK1, FADD, CSK22 |
| Endocytosis | 36 | 340 | 0.11 | 5E-06 | PSD4, CHM4B, CBL, DYN2, SH3K1, HA1K, WASP, EHD1, VP26A, CAZA2, CCR5, IL2RB, HMR1, RAB5A, CYH4, NED4L, MPRI, FGFR4, SNX1, ARAP1, DAB2, ADRB2, CXCR4, ACAP2, PARD3, IL2RG, GIT2, AGAP1, TGFB1, ARF2, GRK6, CYH1, HSP70, ARC1B, RAB10, RAB35 |
| Intestinal immune network for IgA production | 8 | 40 | 0.20 | 8E-06 | ICOSL, CCR9, TNR5, TGFB1, CXCR4, HA2B, H2-Ea, HB2D, |
| Tight junction | 15 | 179 | 0.08 | 2E-05 | CLDY, E41L2, CLD4, MYH9, CLD6, CLD3, SRC8, MPDZ, HCLS1, KPCT, CTNB1, PARD3, ACTN1, CSK22, OCLN |
| NOD-like receptor signaling pathway | 7 | 49 | 0.14 | 2E-04 | CASPC, IKBA, ASC, PPIP1, BIR, IL8, HS90B |

**Table S3.** *Cont.*

| KEGG pathway | Gene number | Background number | Rich factor | *P*-value | Gene enriched |
|---|---|---|---|---|---|
| Toll-like receptor signaling pathway | 11 | 98 | 0.11 | 6E-04 | PK3CD, M3K8, FADD, IKBA, TNR5, IL8, IRF3, CATK, PK3CG, ICOSL, TRAF3 |
| Lysosome | 15 | 144 | 0.10 | 6E-04 | LAP4A, DNS2A, SAP, AP1S3, NPC2, SORT1, CATS, CATH, CATZ, PPGB, CATK, MPRI, PPT2A, CD63, CATB |
| Adherens junction | 12 | 100 | 0.12 | 7E-04 | PARD3, FYN, LMO7, FGR1A, FYNB, PTN6, ACTN1, RAC2, NECT3, CTNB1, CSK22, WASP |
| Regulation of actin cytoskeleton | 21 | 269 | 0.08 | 1E-03 | RAC2, NCKPL, WASP, PAXI, SUPT5H, ITB2, ITAE, COF2, FGFR4, PK3CG, GELS, DOCK2, PK3CD, FGR1A, ITB5, TGFI1, MOES, ARC1B, ACTN1, ARHGC, VAV, |
| Apoptosis | 16 | 173 | 0.09 | 2E-03 | PK3CD, TRAF2, GA45A, GRAB, BIR, CATS, IKBA, CATZ, CATH, TBA3, CATK, GRZ1, PK3CG, BID, FADD, CATB |
| ABC transporters | 5 | 39 | 0.13 | 2E-03 | MRP2, MRP1, ABCB9, TAP2, MRP3 |
| Proteasome | 6 | 55 | 0.11 | 5E-03 | PSB7, PSA2, PSA6, PB6LB, IFNG, PSME1 |
| Metabolic pathways | 71 | 1275 | 0.06 | 6E-03 | ODPX, OTC, ACSL1, CDS2, SIAT2, LPCT4, LIPO, I5P2, SDHL, SAT1, 5NTD, PNPH, AOXC, BHS, ODO2, PCKGC, CY1, PGH2, QCR1, DYR, HPSE, GSHB, PTGIS, NDUS2, GLT18, PLD4, LOX5, GCNT3, UAP1, CX6C1, TKT, PGAM1, LCNL1, PLCG2, GALT8, CDO1, 5NT3, GMDS, RPA12, KGUA, LOX12, MGT4A, SDHA, GPAT4, LTC4S, AATM, G6PD, PA24F, ALN, B4GT1, C1GLT, ALOX5, LKHA4, ADK, DGKD, GMPPB, DHE3, NRK2, NFS1, NEUA, PTGES, PPT2A, OST48, GLCNE, PA24A, ODPA, DGKZ, PLPP1, CX6B1, PLCB3, GFPT2 |
| VEGF signaling pathway | 9 | 81 | 0.11 | 7E-03 | PK3CD, PLCG2, TGFI1, PA24F, PGH2, RAC2, PA24A, PK3CG, PAXI |
| Nicotinate and nicotinamide metabolism | 5 | 29 | 0.17 | 8E-03 | NRK2, 5NT3, 5NTD, PNPH, AOXC |
| Cytosolic DNA-sensing pathway | 6 | 39 | 0.15 | 8E-03 | CASPC, IKBA, ASC, RIPK4, IRF3, RIPK3 |

**Table S4.** The significantly enriched pathways involving differentially expressed genes of the **fin** of HT vs NT (*P < 0.01)*

| **KEGG pathway** | **Gene number** | **Background number** | **Rich factor** | ***P*-value** | **Gene enriched** |
|---|---|---|---|---|---|
| AGE-RAGE signaling pathway in diabetic complications | 10 | 124 | 0.08 | 0.000 | SMAD4, KPCD, CDN1B, MK03, MMP2, EGR1, PIM1, IL1B, JUN, EGR1 |
| Herpes simplex infection | 10 | 183 | 0.05 | 0.000 | SOCS3, IKBA, HG2A, IL1B, JUN, CO3, HB2D, HNRPK, IKBA, EGR1 |
| Metabolic pathways | 35 | 1275 | 0.03 | 0.000 | ODO1, PLPP3, PGH2, PGK, LIPO, PGAM1, UAP1, CBS, METK1, A9A1A, CDO1, MDHC, PCAT1, B4GT4, HGD, ENPP1, ODPX, RPN1, SIA4B, STT3A, LOX5, QCR2, GFPT1, PFKAP, ECHM, IL1B, GLT14, RIR1, PCKGC, PON2, RIR2, CY1, NDUAA, OAT, BCAT1 |
| Glycolysis / Gluconeogenesis | 6 | 73 | 0.08 | 0.000 | PFKAP, PCKGC, ODPX, PGK, PGAM1, A9A1A |
| Apoptosis | 9 | 173 | 0.05 | 0.000 | CATH, CATK, MK03, ACTS, M3K5, IKBA, JUN, CATC, TNF10 |
| Toll-like receptor signaling pathway | 5 | 98 | 0.05 | 0.000 | CATK, MK03, IL1B, IKBA, JUN |
| p53 signaling pathway | 7 | 74 | 0.09 | 0.000 | TSP1, SHSA4, CCNG1, P73, CD82, RIR2, SESN1 |
| Biosynthesis of amino acids | 6 | 86 | 0.07 | 0.001 | PGK, PGAM1, BCAT1, CBS, METK1, PFKAP |
| Focal adhesion | 10 | 245 | 0.04 | 0.001 | CO6A2, TSP1, MK03, ACTS, JUN, VGFR2, CO6A1, PAXI, RAP1B, ITA4 |
| Carbon metabolism | 7 | 125 | 0.06 | 0.002 | ODO1, ODPX, PGK, PGAM1, ECHM, MDHC, PFKAP |
| Cysteine and methionine metabolism | 5 | 50 | 0.10 | 0.002 | CBS, METK1, BCAT1, CDO1, MDHC |
| Cytokine-cytokine receptor interaction | 9 | 165 | 0.05 | 0.002 | CCL28, CCL25, VGFR2, IL1B, I20RB, CCL20, TNF10, CCR6, I20RA |
| Insulin signaling pathway | 6 | 173 | 0.03 | 0.003 | SOCS3, MK03, GYS1, FLOT2, CALM, SOCS3 |
| Aminoacyl-tRNA biosynthesis | 5 | 62 | 0.08 | 0.005 | SYCC, SYAC, SYWC, SYVC, SYFB |
| Phagosome | 7 | 156 | 0.04 | 0.005 | TBB, TSP1, ACTS, CO3, HGS, HB2D, S61A2 |
| Ribosome | 6 | 134 | 0.04 | 0.008 | RL7, RL4B, RM02, RS12, RSSA, RLP24 |
| FoxO signaling pathway | 8 | 171 | 0.05 | 0.009 | MAD4, MK03, CDN1B, PLK2, KLF2, KLF4, PCKGC, TNF10 |

**Table S5.** List of DEGs associated with ECM in gill

| Gene | Gene symbol | log2FC |
|---|---|---|
| Collagen alpha-1(I) chain | CO1A1 | 1.07 |
| Collagen alpha-2(I) chain | CO1A2 | 1.23 |
| Collagen alpha-1(II) chain | CO2A1 | 3.05 |
| Collagen alpha-2(IV) chain | CO4A2 | 0.82 |
| Collagen alpha-3(IX) chain | CO9A3 | 2.36 |
| Platelet glycoprotein Ib beta chain | GP1BB | 1.78 |
| Integrin beta-5 | ITB5 | 1.04 |
| Laminin subunit beta-1 | LAMB1 | 1.02 |
| Laminin subunit beta-4 | LAMB4 | 1.33 |
| Laminin subunit gamma-3 | LAMC3 | 1.19 |
| Variant-silencing SET domain-containing protein | SETVS | -1.37 |
| Thrombospondin-4-B | TSP4B | 0.97 |

**Table S6**. List of DEGs associated with CAMs in gill

| Gene | Gene symbol | log2FC |
|---|---|---|
| Claudin-like protein ZF-A89 | CLDY | -2.41 |
| Claudin-4 | CLD4 | -2.56 |
| Neural cell adhesion molecule L1 | L1CAM | -2.20 |
| Tumor necrosis factor receptor superfamily member 5 | TNR5 | -3.64 |
| H-2 class I histocompatibility antigen, K-K alpha chain | HA1K | -9.48 |
| Nectin-3 | NECT3 | -2.17 |
| H-2 class II histocompatibility antigen, A-B alpha chain | HA2B | -2.53 |
| Claudin-6 | CLD6 | -5.11 |
| Claudin-3 | CLD3 | -2.07 |
| ICOS ligand | ICOSL | -2.26 |
| Major histocompatibility complex class I-related gene protein | HMR1 | -8.55 |
| CD276 antigen homolog | CD276 | -2.93 |
| T-cell surface antigen CD2 | CD2 | -2.40 |
| Receptor-type tyrosine-protein phosphatase C | PTPRC | -1.30 |
| DLA class II histocompatibility antigen, DR-1 beta chain | HB2D | -2.06 |
| Integrin beta-2 | ITB2 | -2.97 |
| H-2 class II histocompatibility antigen, A-U alpha chain-like | H2-Ea | -2.58 |
| Cytotoxic T-lymphocyte protein 4 | CTLA4 | -3.43 |
| CD226 antigen | CD226 | -1.99 |
| Occludin | OCLN | -1.18 |

**Table S7**. List of DEGs associated with cell junction and adherens in gill

| Gene | Gene symbol | log2FC |
|---|---|---|
| Alpha-actinin-1 | ACTN1 | 0.81 |
| Multiple PDZ domain protein | ARC1B | 3.37 |
| Rho guanine nucleotide exchange factor 12 | ARHGC | -2.62 |
| Inhibitor of apoptosis protein | BIR | -1.78 |
| Claudin-3 | CLD3 | -2.07 |
| Claudin-4 | CLD4 | -2.56 |
| Claudin-6 | CLD6 | -5.11 |
| Claudin-like protein ZF-A89 | CLDY | -2.41 |
| Collagen alpha-1(I) chain | CO1A1 | 1.07 |
| Collagen alpha-2(I) chain | CO1A2 | 1.23 |
| Collagen alpha-1(II) chain | CO2A1 | 3.05 |
| Collagen alpha-2(IV) chain | CO4A2 | 0.82 |
| Collagen alpha-3(IX) chain | CO9A3 | 2.36 |
| Cofilin-2 | COF2 | -0.86 |
| Casein kinase II subunit alpha | CSK22 | 4.53 |
| Catenin beta-1 | CTNB1 | 1.01 |
| Dedicator of cytokinesis protein 2 | DOCK2 | -3.29 |
| Band 4.1-like protein 2 | E41L2 | -1.19 |
| Fibroblast growth factor receptor 4 | FGFR4 | 1.03 |
| Fibroblast growth factor receptor 1-A | FGR1A | -4.42 |
| Filamin-A | FLNA | 1.91 |
| Filamin-C | FLNC | 1.22 |
| Tyrosine-protein kinase Fyn | FYN | -1.65 |
| Tyrosine-protein kinase fynb | FYNB | -1.55 |
| Gelsolin | GELS | -0.88 |
| Hematopoietic lineage cell-specific protein | HCLS1 | -1.49 |
| Integrin alpha-E | ITAE | -1.74 |
| Integrin beta-2 | ITB2 | -2.97 |
| Integrin beta-5 | ITB5 | 1.04 |
| Protein kinase C theta type | KPCT | -4.60 |
| Laminin subunit beta-1 | LAMB1 | 1.02 |
| Laminin subunit beta-4 | LAMB4 | 1.33 |
| Laminin subunit gamma-3 | LAMC3 | 1.19 |
| LIM domain only protein 7 | LMO7 | -7.57 |
| Moesin | MOES | -0.84 |
| Multiple PDZ domain protein | MPDZ | 3.37 |
| Myosin-9 | MYH9 | -5.22 |
| Nck-associated protein 1-like | NCKPL | -2.75 |
| Nectin-3 | NECT3 | -2.17 |

**Table S7**. *Cont.*

| Gene | Gene symbol | log2FC |
|---|---|---|
| Occludin | OCLN | -1.18 |
| Partitioning defective 3 homolog | PARD3 | 3.52 |
| Paxillin | PAXI | 3.73 |
| Phosphatidylinositol 4,5-bisphosphate 3-kinase catalytic subunit delta isoform | PK3CD | -2.18 |
| Phosphatidylinositol 4,5-bisphosphate 3-kinase catalytic subunit gamma isoform | PK3CG | -1.98 |
| Tyrosine-protein phosphatase non-receptor type 6 | PTN6 | -1.43 |
| Ras-related C3 botulinum toxin substrate 2 | RAC2 | -1.00 |
| Src substrate protein p85 | SRC8 | -6.81 |
| Transcription elongation factor SPT5 | SUPT5H | -2.44 |
| Transforming growth factor beta-1-induced transcript 1 protein | TGFI1 | 1.01 |
| Thrombospondin-4-B | TSP4B | 0.97 |
| Vasodilator-stimulated phosphoprotein | VASP | -1.65 |
| Proto-oncogene vav | VAV | -1.61 |
| Vascular endothelial growth factor D | VEGFD | 4.78 |
| Wiskott-Aldrich syndrome protein homolog | WASP | -3.68 |

**Table S8**. List of DEGs associated with glycolysis in fin

| Gene | Gene symbol | log2FC |
|---|---|---|
| Aldehyde dehydrogenase family 9 member A1-A | A9A1A | 6.93 |
| Pyruvate dehydrogenase protein X component | ODPX | 6.74 |
| Phosphoenolpyruvate carboxykinase, cytosolic | PCKGC | -5.67 |
| ATP-dependent 6-phosphofructokinase, platelet type | PFKAP | 4.98 |
| Phosphoglycerate mutase 1 | PGAM1 | 1.11 |
| Phosphoglycerate kinase | PGK | 1.32 |

**Table S9**. List of DEGs associated with p53 signaling pathway in fin

| Gene | Gene symbol | log2FC |
|---|---|---|
| Thrombospondin-1 | TSP1 | -3.51 |
| Protein shisa-4 | SHSA4 | -6.58 |
| Cyclin-G1 | CCNG1 | -1.35 |
| Tumor protein p73 | P73 | 22.72 |
| CD82 antigen | CD82 | -8.18 |
| Ribonucleoside-diphosphate reductase subunit M2 | RIR2 | 1.42 |
| Sestrin-1 | SESN1 | -1.18 |
